# Breaking the blue limit of non-conjugated hydrocarbons via multiple through-space interactions

**DOI:** 10.1093/nsr/nwag257

**Published:** 2026-05-07

**Authors:** Shuxin Jiang, Ziwei Deng, Xiaoming Wang, Zheng Zhao, Ben Zhong Tang

**Affiliations:** State Key Laboratory of Organometallic Chemistry and Shanghai-Hong Kong Joint Laboratory in Chemical Synthesis, Shanghai Institute of Organic Chemistry, University of Chinese Academy of Sciences, Chinese Academy of Sciences, Shanghai 200032, China; Guangdong Basic Research Center of Excellence for Aggregate Science, School of Science and Engineering, Shenzhen Institute of Aggregate Science and Technology, The Chinese University of Hong Kong (Shenzhen), Shenzhen 518172, China; State Key Laboratory of Organometallic Chemistry and Shanghai-Hong Kong Joint Laboratory in Chemical Synthesis, Shanghai Institute of Organic Chemistry, University of Chinese Academy of Sciences, Chinese Academy of Sciences, Shanghai 200032, China; School of Chemistry and Materials Science, Hangzhou Institute for Advanced Study, University of Chinese Academy of Sciences, Hangzhou 310024, China; School of Chemistry and Chemical Engineering, Henan Normal University, Xinxiang 453007, China; Guangdong Basic Research Center of Excellence for Aggregate Science, School of Science and Engineering, Shenzhen Institute of Aggregate Science and Technology, The Chinese University of Hong Kong (Shenzhen), Shenzhen 518172, China; Guangdong Basic Research Center of Excellence for Aggregate Science, School of Science and Engineering, Shenzhen Institute of Aggregate Science and Technology, The Chinese University of Hong Kong (Shenzhen), Shenzhen 518172, China

**Keywords:** through-space interaction, non-conventional luminescent materials, aggregation-induced emission, hydroarylation, allenes

## Abstract

The development of non-conjugated luminescent systems has innovated the traditional design strategy of through-bond conjugation (TBC) and inspired a deeper understanding on molecular photophysics. However, a challenge encountered by these non-conjugated structures is that the fluorescence of pure hydrocarbon systems is largely confined to the blue region (∼470 nm), raising the question of whether this is a fundamental limitation. Herein, we break this barrier by constructing a library of over 50 non-conjugated arylated alkenes via binuclear nickel-catalyzed hydroarylation of allenes. It is disclosed that synergistic multiple through-space interactions (TSIs) including molecular twisting-tuned intramolecular TSIs combined with intermolecular TSIs mediated by the double bonds of styrene units are critical for achieving the ultralong-wavelength aggregation-induced emission of these non-conjugated structures. This enables tunable light emission from blue to yellow, orange and, remarkably, even to single-compound-based warm white light. Furthermore, strategic substitution extends the fluorescence into the deep-red region, with a tail reaching 900 nm. This work demonstrates that non-conjugated pure hydrocarbons can achieve a broad spectrum of colors, fundamentally advancing the understanding of the luminescence phenomenon of non-conjugated systems and providing a novel design strategy for luminescent materials beyond the traditional TBC paradigm.

## Teaser

Breaking the blue limit of non-conjugated hydrocarbons: via through-space interaction, scientists have pushed pure hydrocarbon fluorescence from blue to deep-red, unlocking warm white light from a single molecule.

## INTRODUCTION

**Figure 1.**
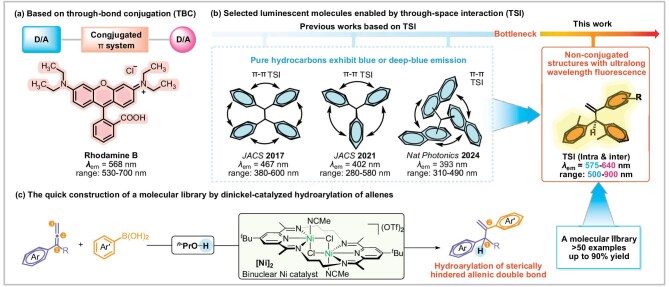
Construction of a library of TSI-based luminescent molecules with long-wavelength emission. (a) Luminogens relying on TBC. (b) Selected luminescent molecules enabled by TSI. (c) The quick construction of a molecular library by dinickel-catalyzed hydroarylation of allenes.

Luminescent materials hold immense potential in a variety of applications, including optoelectronic devices [1–4], disease diagnosis [5,6], bioimaging [7–10] and information encryption [11,12]. A central objective in the field is the precise regulation of emission wavelengths to be adaptable to different scenarios [13,14]. Conventional energy splitting theory dictates that electron delocalization via π-conjugated covalent bonds is necessary to modulate the energy gap and thereby control absorption and emission [15,16]. This principle underpins the traditional design of luminogens, which involves connecting π-conjugated building blocks through covalent bonds in a strategy known as through-bond conjugation (TBC, Fig. 1a) [17–19]. This has also led to non-conjugated molecules, and the macroscopic materials made from them, being largely overlooked due to their seemingly non-emissive nature. On the other hand, the non-conventional luminescent materials recently developed by researchers have demonstrated that through-space interaction (TSI)—a much weaker interaction than chemical bonding—can induce excellent solid-state visible fluorescence, even when through-bond π-electron delocalization is blocked [20–22]. For example, Zhu *et al*. discovered that the five-membered-ring succinimide-derived cyclic imides can emit room temperature phosphorescence (RTP) in the red and near-infrared (NIR) spectral range, in spite of their very limited conjugation length. Further mechanism investigation indicated that the clustering of imide, C=C and halogen moieties help to extend the electron delocalization and boost the spin orbital coupling (SOC) and intersystem crossing (ISC), enabling the RTP emission in red and NIR regions [20]. Zhang *et al*. studied the interesting luminescence behavior of a group of non-conjugated cyclohexanedione isomers. They demonstrated that the TBC effect reduced the highest occupied molecular orbital (HOMO)–lowest unoccupied molecular orbital (LUMO) energy gap of a single molecule, and it facilitated the formation of stronger through-space conjugation (TSC) in the aggregate state. The cooperative effect of TBC and TSC leads to more significantly red-shifted emissions [21]. By employing non-conjugated tetraphenylethane as a model compound, Zhang *et al*. demonstrated that intramolecular TSC between the ‘isolated’ phenyl rings played an important role for this abnormal visible fluorescence emission of these compounds in the solid state [22]. In comparison with the conjugated structures, these non-conjugated luminogens not only challenge the fundamental design principles of current fluorescent materials but also offer advantages such as high solubility, low toxicity and potential biological activity. This makes them highly promising for advanced optoelectronics and bioimaging [23].

Driven by this new strategy, non-conjugated luminescent systems with the property of aggregation-induced emission (AIE) have been developed that emit across the visible-to-red spectrum [22,24–26]. However, the reported long-wavelength fluorescence in non-conjugated systems has generally required the introduction of heteroatoms or heteroaromatics. Most pure hydrocarbons exhibit blue or deep-blue emission, typically not exceeding ∼470 nm (Fig. 1b) [22,24–27]. This raises a critical question: is blue emission the intrinsic ceiling for non-conjugated hydrocarbons? Therefore, constructing novel non-conjugated hydrocarbons, exploring the upper limit of their emission wavelength, and unveiling the structure–property relationship are not only significant to enrich the current fluorescent molecular systems but also essential to deepen the understanding of modern photophysics.

Herein, we successfully prepared a series of arylated alkenes through a bis(pyridyldiimine) (PDI) dinickel complex-catalyzed 1,2-hydroarylation of 1,1-diarylallenes in the presence of arylboronic acids and *n*-PrOH, achieving the hydroarylation of the inner double bond with high regioselectivities (Fig. 1c). Crucially, we discovered that the presence of a double bond and molecular twisting played a significant role in shifting the emission wavelength of non-conjugated hydrocarbons into the yellow and orange fluorescence regions. Remarkably, one derivative even exhibits single-compound-based warm white light emission.

Utilizing this methodology, a molecular library comprising over 50 homologues of prop-2-ene-1,1,2-triyltribenzene (TBE) with TSI characteristics was constructed efficiently. By introducing diverse substituents, the emission wavelength could be further extended into the deep-red region, with an emission tail reaching 900 nm. This far surpasses the emission wavelengths achieved by many small molecules based on TBC with comparable molecular size. This work not only deepens the understanding of emission behaviors in non-conjugated hydrocarbons but also provides an innovative alternative to the conventional TBC design strategy.

## RESULTS

### Molecular library construction

**Figure 2.**
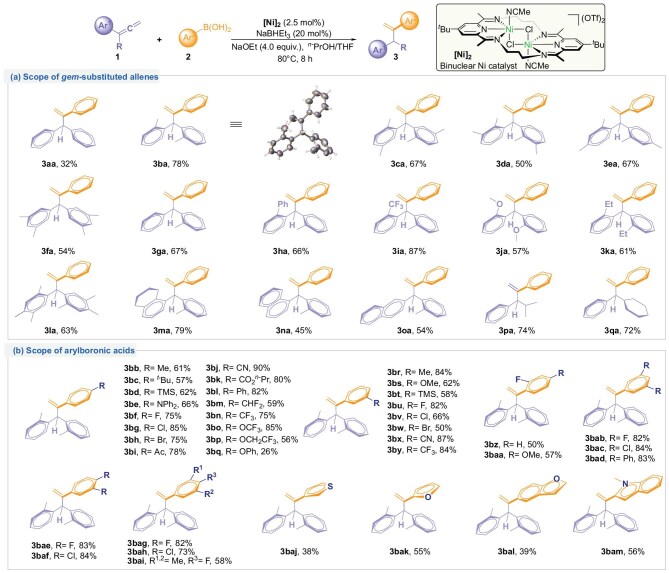
A molecular library of prop-2-ene-1,1,2-triyltriarenes constructed by dinickel-catalyzed hydroarylation. Percentages represent isolated yields. (a) Scope of gem-substituted allenes. (b) Scope of arylboronic acids.

Ni-catalyzed hydroarylation of olefins with arylboronic acids and alcohols has evolved into a rapidly developing strategy for accessing arylated products [28,29]. However, to the best of our knowledge, this elegant strategy has never been explored in the hydroarylation of allenes, probably due to the problematic control of regio- and stereoselectivity [30–32]. As part of our research efforts aiming at the development of binuclear catalysis [33–39], we commenced our studies by using 1,1-diphenylallene **1a** and phenylboronic acid **2a** as the model substrates and a PDI–dinickel complex as the catalyst, and the reaction was carried out in the presence of NaBHEt_3_ and NaOEt in a solvent mixture of *n*-PrOH and tetrahydrofuran (THF) at 80°C for 8.0 h [38,39]. In this case, the 1,2-hydroarylation product **3aa** was obtained in 32% yield with high regioselectivity (>20:1). Further analysis indicated that 1,1-diphenylallene is unstable and can spontaneously polymerize, even at room temperature. To our delight, using sterically bulkier 1,1-di-*o*-tolylallene as the allene substrate, the reaction gave the 1,2-hydroarylation product **3ba** in 78% yield. Control experiments suggested that the unique binuclear structure of the Ni complex might play a key role in directing the reactivity, since other common mono-Ni catalysts only gave very poor yields of the desired product **3ba** (Table S7). Based on this novel methodology, we succeeded in creating a library of TBEs with diverse electronic/steric features, as shown in Fig. 2. The reactions of allene substrates bearing *ortho-* or *meta-* substituents on the aromatic rings, such as methyl, ethyl, phenyl, methoxy and trifluoromethyl, all afforded the corresponding hydroarylation products **3ca**–**3oa** in 45%–87% yields. Moreover, the reaction is also regioselective for 1-alkyl-1-arylallenes, giving the corresponding 1,2-hydroarylation products **3pa** and **3qa** in good yields. However, the reactions of 1-methyl-1-phenylallene or 1-phenylallene only led to intractable mixtures of regioisomeric products. Presumably, the steric hindrance of the allene might play an important role in dictating the reaction outcomes. In addition, a wide range of arylboronic acids with different steric and electronic natures reacted smoothly with allene **1b**, affording the desired TBEs (**3bb**–**3bam**) in moderate to high yields, revealing good functional group compatibility of this protocol. Finally, a TBE molecular library (>50) was successfully established by way of this unique dinickel-catalyzed 1,2-hydroarylation, providing a good platform for further photophysical studies by exploiting the potential TSIs.

### Photophysical properties

**Figure 3.**
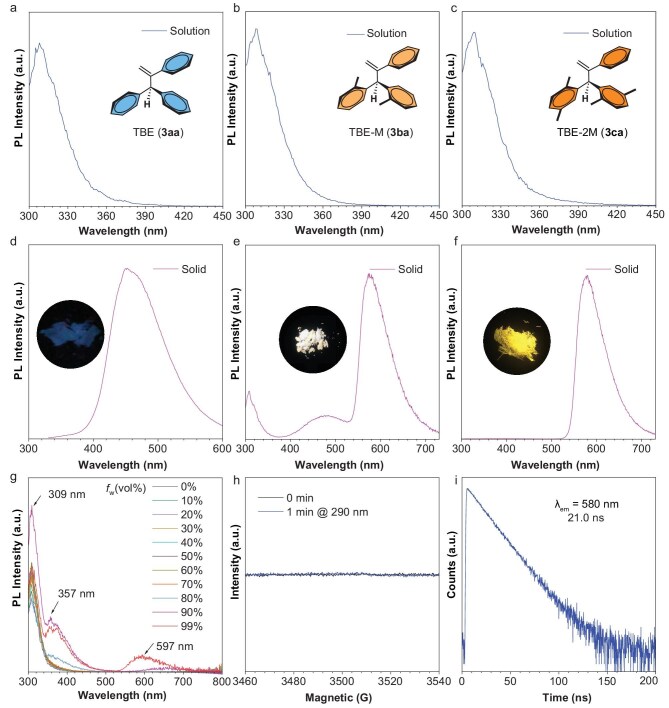
(a–c) PL spectra of TBE, TBE-M and TBE-2M in THF solution. *c* = 10^−4^ M, *λ*_ex_ = 260 nm. (d–f) PL spectra of TBE, TBE-M and TBE-2M in the solid state. *λ*_ex_ = 290 nm. The inset images of (d–f) are photos of TBE, TBE-M and TBE-2M solid under irradiation of 365, 290 and 290 nm, respectively. (g) PL spectra of TBE-2M in THF/water mixtures with different water fractions (*f*_w_). *c* = 10^−4^ M, *λ*_ex_ = 290 nm. (h) Solid-state ESR spectrum of TBE-2M under dark or 290 nm UV light. (i) Time-resolved PL decay curves of TBE-2M measured at maximum emissions of 580 nm.

The basic photophysical properties of a TBE molecular library are summarized in Table S8. In THF solution, these compounds exhibited maximum absorption wavelengths in the range of approximately 220–280 nm and maximum emission wavelengths in the range of approximately 300–400 nm. In the solid state, the powders of these compounds displayed maximum emission wavelengths spanning approximately 350–640 nm. Considering their non-conjugated systems, it was interesting to note that some compounds emitted blue light in solution but orange or red light in the solid state. Moreover, these non-conjugated systems with modification of the molecular backbone including introducing heteroatoms (**3bu, 3bab, 3ia**) and changing numbers of methyl groups (**3aa, 3ba, 3ca, 3la, 3bb**) generally obtain long-wavelength emission. To investigate this unexpected long-wavelength emission behavior in non-conjugated systems, we chose the products TBE (**3aa**), TBE-M (**3ba**) and TBE-2M (**3ca**) as representatives for further detailed studies of the photophysical properties, with their purities confirmed by high-performance liquid chromatography (HPLC) analysis (Fig. S1). TBE, TBE-M and TBE-2M in THF solution exhibited absorption maxima at wavelengths of approximately 246, 244 and 242 nm, respectively (Fig. S2). These values were found to closely resemble the absorption maximum (250 nm) of styrene, as depicted in Fig. S3, which is consistent with their non-conjugated structures. Furthermore, upon excitation with 260 nm light (Fig. S4), the photoluminescence (PL) spectra showed that TBE, TBE-M and TBE-2M each exhibited a single emission peak at approximately 309 nm in THF solution, with quantum yields (QYs) of 2.5%, 4.5% and 2.9%, respectively (Fig. 3a–c). This emission can be ascribed to the styrene unit, which was further evidenced by the similar peaks in the emission spectrum of styrene (Fig. S3). In the solid state, under daylight, all compounds appeared as white powdered solids with similar macroscopic appearances. In contrast, under 290 nm ultraviolet (UV) irradiation, they exhibited distinct fluorescence emissions, with colors spanning from approximately 450 nm (blue) to 580 nm (orange). When excited at 290 nm, TBE powder emitting blue light exhibited a single emission peak at approximately 450 nm with a QY of 1.5% (Fig. 3d and Fig. S5), similar to the maximum emission (445 nm) of the crystals of TBE (Fig. S6a). Under the same excitation conditions, the TBE-M powder emitting white light displayed three distinct emission peaks at 309, 480 and 575 nm (Fig. 3e), respectively, with a higher QY of 19.8%. To evaluate the quality of generated white light emitted by the TBE-M, its International Commission on Illumination (CIE) coordinates and correlated color temperature (CCT) were determined. The TBE-M emission displayed CIE coordinates of (0.48, 0.43) and a CCT of 2604 K (as shown in Fig. S7), suggesting it corresponds to warm white light [40]. To further understand the origin of the three emission peaks of TBE-M solid, the excitation-dependent emission behavior has been investigated (Fig. S8). The results indicated that when the excitation wavelength increased from 250 to 300 nm, TBE-M exhibited three emission peaks including the single molecular emission at 309 nm and two additional emission peaks at 480 and 575 nm. Further increasing the excitation wavelength from 310 to 360 nm, the peaks at 309 and 575 nm disappeared and the peak at 480 nm was enhanced. The excitation-dependent emission behavior suggests the multi-peak emission of TBE-M in the solid state could be ascribed to the different emission species, including the monomer and multimer existing within the aggregates of TBE-M. In contrast to TBE-M powder, the TBE-2M powder emitting orange light showed a single emission peak at 580 nm with a QY of 14.8% (Fig. 3f and Fig. S5). Likewise, the crystals of TBE-M also showed three peaks at 309, 482 and 577 nm, while the crystals of TBE-2M displayed an emission peak at 575 nm (Fig. S6b and S6c).

Notably, these compounds exhibited similar emission behaviors in solution but displayed obviously different emission performance in the solid state. Such different emission behavior associated with the phase change prompted us to study their PL upon aggregation in water. Aggregates of these tested compounds were generated by increasing the water fraction in the THF/water mixture, resulting in discernible changes in emission intensity (Figs S9–S10 and Fig. 3g). It is noteworthy that in these cases, the solutions of TBE and TBE-2M exhibited new peaks at 375 and 357 nm, respectively, when the fraction of water (*f*_w_) increased to 80%, suggesting that intramolecular TSIs may have formed [24]. Additionally, similar red-region luminescence peaks appeared at approximately 610 and 597 nm for TBE-M and TBE-2M, respectively, at a *f*_w_ value of 99% (Fig. 3g and Fig. S10). For each of these three compounds, the corresponding solid-state excitation spectra related to the emission peak at long wavelengths were markedly different from those of styrene units (Fig. S11). Since the red emission of these compounds cannot align with the fluorescence of their non-conjugated structures, it raises the possibility that the solid emission may originate from radical emission or phosphorescence [41,42]. To investigate these possibilities, electron spin resonance (ESR) spectra were further measured on the solid samples of these compounds. The spectra showed no significant radical signals, regardless of whether the samples were irradiated with 290 nm light or not (Fig. 3h and Fig. S12) [43]. Additionally, the lifetimes of TBE, TBE-M and TBE-2M for each emission peak were measured to be no more than 30 ns, which were typical fluorescence lifetimes rather than those characteristic of phosphorescence (Fig. 3i and Fig. S13). Furthermore, the photochemical stability of TBE-2M solid upon UV irradiation was evaluated by HPLC and field ionization high-resolution mass spectrometry (FI-HRMS) (Fig. S14), demonstrating that no photochemical byproduct was generated after UV irradiation (290 nm) for 15 min. The photophysical properties of all molecules are summarized in Table S9. These studies revealed that the incorporation of the methyl groups in TBE compounds resulted in a new emission band in the range of 575–610 nm in the solid state. It is indeed intriguing that these non-conjugated molecules, despite having a styryl moiety as the largest chromophore, could exhibit unimaginable long-wavelength yellow emission. This observation challenges the conventional impression of the relationship between conjugation length and emission wavelength, and suggests that aggregation may generate non-typical chromophores that go beyond the TBC design principle.

**Figure 4.**
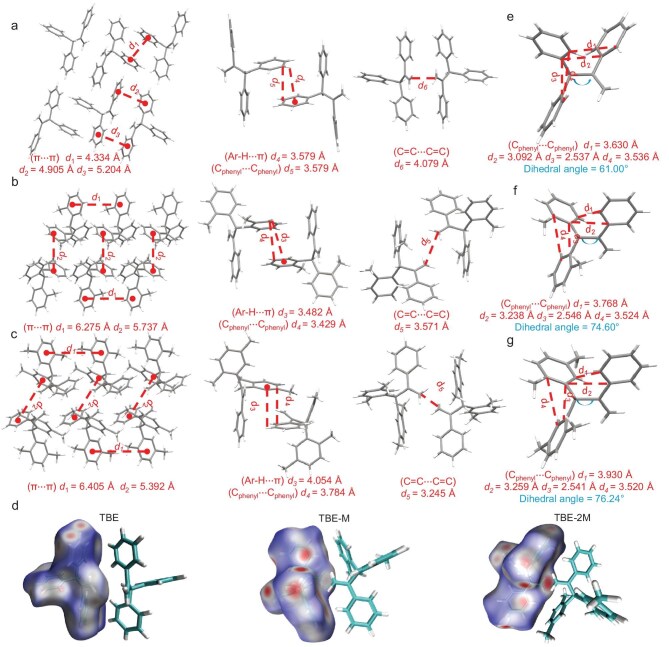
(a–c) Crystal packing diagrams of (a) TBE, (b) TBE-M and (c) TBE-2M. (d) Hirshfeld surfaces of TBE, TBE-M and TBE-2M, where the intermolecular interactions are highlighted by red spots. (e–g) Single-molecule conformations illustrating intramolecular distances and dihedral angles of (e) TBE, (f) TBE-M and (g) TBE-2M.

Single crystals can offer important information to explore the structure–property relationship in the aggregate state, which would be valuable in elucidating the origin of the unique emission behavior of these TBE derivatives. Therefore, the crystal structures of TBE derivatives were obtained and studied (Fig. 4, Tables S13–S15). In general, none of the three molecules exhibited dense packing, with the intermolecular centroid–centroid distances around 4.334–6.405 Å, suggesting no strong π–π stacking. However, some non-covalent Ar–H**⋅⋅⋅**π and C_phenyl_**⋅⋅⋅**C_phenyl_ weak interactions have been observed within the crystals of TBE, TBE-M and TBE-2M, respectively (Fig. 4). Among the crystals of the three compounds, the weak interactions in TBE-M (Ar–H**⋅⋅⋅**π, 3.482 Å; C_phenyl_**⋅⋅⋅**C_phenyl_, 3.429 Å) are stronger than those in TBE (Ar–H**⋅⋅⋅**π, 3.579 Å; C_phenyl_**⋅⋅⋅**C_phenyl_, 3.579 Å) and TBE-2M (Ar–H**⋅⋅⋅**π, 4.054 Å; C_phenyl_**⋅⋅⋅**C_phenyl_, 3.784 Å). Additionally, in comparison to TBE, the introduction of methyl groups at the *ortho-* positions of the benzene rings led to the observation of some CH_3_**⋅⋅⋅**π interactions in the crystals of TBE-M and TBE-2M, with distances of 3.042 and 2.999 Å, respectively (Fig. S15). These intermolecular interactions help rigidify the molecular conformation and boost the intramolecular electron communication, leading to the relatively higher PLQY of TBE-M and TBE-2M (19.8% and 14.8%) than of TBE (1.5%) in the solid state. Through comprehensively considering the Ar–H**⋅⋅⋅**π interactions, CH_3_**⋅⋅⋅**π interactions and C_phenyl_**⋅⋅⋅**C_phenyl_ interactions, TBE-M exhibited stronger intermolecular non-covalent interactions than TBE and TBE-2M, which well explained its highest solid PLQY among the three molecules.

Interestingly, although TBE-M exhibited the highest solid PLQY among the three molecules, TBE-2M showed a longer emission wavelength (580 nm) than that of both TBE-M (575 nm) and TBE (450 nm). It is noteworthy that the emission wavelengths of the three molecules corresponded well with their intermolecular C=C**⋅⋅⋅**C=C interactions, which had been reflected by the C=C**⋅⋅⋅**C=C distances of 4.079, 3.571 and 3.245 Å for TBE, TBE-M and TBE-2M, respectively. Quantitative analysis of intermolecular interactions using the Hirshfeld surface method was further performed to better evaluate the intermolecular interactions. As illustrated in Fig. 4d, the red regions represented an effective electron overlap, TBE mainly exhibited C–H**⋅⋅⋅**π interactions, while distinct intermolecular interactions of double bonds in styrene units were observed in TBE-M and TBE-2M. These results suggested that the intermolecular mutually interacted double bonds may play a significant role in boosting the intermolecular electron delocalization between the styrene moieties and thus contribute to the redshift of the emission wavelength, with stronger C=C**⋅⋅⋅**C=C interaction affording longer emission wavelength.

However, regarding the UV absorption and emission of the styrene moiety, the electron delocalization between the intermolecular styrene moieties may not be sufficient to produce yellow emission approaching 580 nm; we thus further considered other factors that may contribute to the long emission of TBE-M and TBE-2M. As shown in Fig. 4e–g, the C_phenyl_**⋅⋅⋅**C_phenyl_ distance of the isolated phenyl rings within one molecule was measured as 2.537, 2.546 and 2.541 Å for TBE, TBE-M and TBE-2M, respectively. These distances suggest the presence of intramolecular TSIs among the isolated phenyl rings, which may serve as an additional approach to boosting the electron delocalization to contribute to the formation of the chromophore. Consequently, the introduction of methyl groups in TBE-M and TBE-2M enhanced the molecular conformation frustration, causing the intramolecular and intermolecular p-orbitals overlap and enhanced C_phenyl_**⋅⋅⋅**C_phenyl_ intramolecular electron delocalization. In addition, the introduction of methyl groups also reduced the intermolecular C=C**⋅⋅⋅**C=C distance between sp^2^ carbon atoms, facilitating a C=C**⋅⋅⋅**C=C intermolecular electron delocalization between the adjacent styrene units. This distinct interaction pattern caused the different fluorescence behaviors of these molecules in their aggregated states.

**Figure 5.**
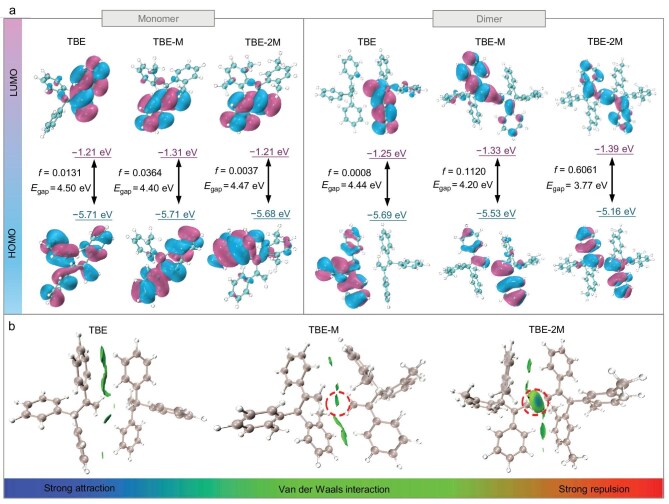
Theoretical calculations. (a) Frontier molecular orbitals of optimized excited-state geometries of monomer and dimer of TBE derivatives calculated by the ONIOM method. The quantum mechanical (QM) layer was calculated by the TD-DFT method at the B3LYP-D3/6-31G(d,p) level and the molecular mechanics (MM) layer was calculated by the UFF method in the Gaussian 16 program. (b) IGMH analysis of optimized excited-state dimer geometries of TBE derivatives [47–49].

To further investigate whether these intermolecular or intramolecular interactions would influence their electronic structures, theoretical calculations were conducted using the ONIOM method on single molecules and dimers of TBE derivatives to visualize the HOMO and the LUMO at the excited states (Figs S16–S18, Tables S10–S12). As shown in Fig. 5a, the HOMO of TBE was delocalized across the entire non-conjugated molecular backbone, whereas the HOMO of TBE-M was distributed on two adjacent phenyl rings and the styryl unit. In contrast, the HOMO of TBE-2M was mainly situated on one of the isolated benzene rings and partially extended to the adjacent phenyl ring. Meanwhile, the LUMOs of TBE, TBE-M and TBE-2M exhibit a consistent distribution pattern, being mainly confined to the styryl unit in each molecule. These results suggest that upon excitation, TBE and TBE-M exhibit both local excited (LE) state transition and through-space charge transfer (TSCT) characteristics [44,45], whereas TBE-2M predominantly displays TSCT behavior, indicating that methyl substitution significantly influences the electronic structure of the TBE derivatives. Furthermore, the oscillator strengths of TBE (0.0131) and TBE-M (0.0364) were more than 10 times higher than that of TBE-2M (0.0037), suggesting that monomer emission is more favorable for TBE and TBE-M than for TBE-2M. However, the dimers of TBE, TBE-M and TBE-2M exhibited markedly different electronic structures compared to their monomeric forms. For instance, in the TBE dimer, the HOMO is localized on one molecule, while the LUMO resides on the other, resulting in an intermolecular charge-transfer (CT) state with a low oscillator strength of 0.0008. This explains why TBE displays only deep-blue monomer emission without any longer-wavelength dimer emission. In contrast, the HOMO of the TBE-M dimer shows clear through-space electron delocalization across both molecules, combining LE and CT characteristics. The increased oscillator strength reflects a favorable HOMO–LUMO transition with a hybrid LE and CT nature. The high oscillator strengths of both the monomer and dimer of TBE-M are consistent with its dual emission in the aggregate state. Similarly, TBE-2M exhibits a hybrid LE and CT electronic structure akin to that of TBE-M, in agreement with its longer-wavelength emission. However, due to the significantly lower oscillator strength of its monomer, TBE-2M displays only a single bright long-wavelength emission compared to TBE-M. Moreover, the calculated electron-hole distributions of TBE derivatives in their monomer and dimer states, as shown in Fig. S19, further demonstrated the through-space electron delocalization characteristic of the TBE-M and TBE-2M dimers, as well as their lower energy levels compared to those of the corresponding monomers. Overall, the theoretical calculations for the monomers and dimers of TBE, TBE-M and TBE-2M are in good agreement with the experimentally measured PL spectra. It is worth noting that the calculated energy gaps appeared to be slightly larger than the actual emission peaks, which commonly happens and may be ascribed to the tendency of hybrid density functionals to overestimate transition energies [46].

To exclude the interference of different packing modes, we also calculated the other type of dimer that lacks C=C**⋅⋅⋅**C=C interactions in the crystal. As shown in Fig. S20, only some CT characteristics were observed, which have been reflected by the separated HOMO and LUMO, and no obvious TSI has been observed, corresponding with their electron-hole distribution characteristics (Fig. S21). Moreover, the weak oscillator strength of TBE-M and TBE-2M suggests an unfavorable transition. Therefore, this packing mode should not be reasonable for the long-wavelength emission of TBE-M and TBE-2M. To further probe the intermolecular interactions within these dimers, we conducted independent gradient model based on Hirshfeld (IGMH) analysis (Fig. 5b). The results revealed distinct green regions—indicative of van der Waals interactions—localized around the double bonds in the styrene units of TBE-M and TBE-2M dimers, corroborating the presence of C=C⋯C=C interactions. Intriguingly, the addition of methyl groups enhances the C=C⋯C=C interactions of the styrene units in the dimer, despite the steric hindrance typically imposed by such substituents. This counterintuitive molecular frustration effect likely contributes to the observed emission red shift, underscoring how targeted structural modifications can induce conformational changes in the solid or aggregated state, thereby modulating electronic interactions and photophysical properties.

Moreover, to further elucidate the role of through-space C=C⋯C=C interaction in affording the chromophore and long-wavelength emission in the aggregate state, the olefin-hydrogenated product of TBE-M was designed and synthesized, named TBE-M-H. The absorption and emission maxima of TBE-M-H were located at 263 and 288 nm in THF solution (Fig. S22). Since TBE-M-H occurs as a liquid at room temperature, only its aggregate state was tested. The AIE curves showed that the emission intensity of TBE-M-H at 288 nm initially increased and subsequently decreased (Fig. S23). With the formation of aggregate at an *f*_w_ value over 70%, the TSI at 395 nm was drastically enhanced. However, no longer emission peak within the approximate range of 500 to 600 nm emerged in the aggregate state, revealing the significance of the styryl unit in affording long emission.

**Figure 6.**
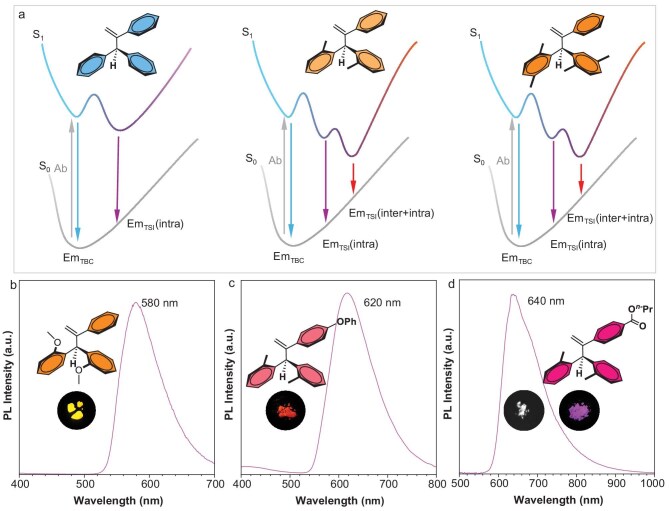
(a) Proposed luminescence mechanism and the potential energy surfaces of TBE derivatives in aggregate states. Ab = absorption, Em = emission. PL intensity spectra of (b) TBE-O, (c) TBE-OPh and (d) TBE-E in the solid state. *λ*_ex_ = 290, 300 and 305 nm, respectively. Insets: fluorescence photographs of (b) TBE-O, (c) TBE-OPh and (d) TBE-E under 290 nm (right) irradiation, and TBE-E under 254 nm (left) irradiation using 900 nm LP filter and 20 ms exposure time.

Through experimental and theoretical analysis, the possible photophysical process of TBE derivatives in aggregates was proposed and is shown in Fig. 6a. Upon photoexcitation to S_1_, the excitons decay via different radiative channels to produce emission. For TBE, the emission is attributed to radiative transitions from the styryl units, as well as intramolecular TSIs. In contrast, TBE-M and TBE-2M exhibit radiative channels containing the emission of styrene units, intramolecular TSIs and intermolecular TSIs within the dimer, contributing to their redshifted emission and enhanced emission intensity. To further demonstrate the plausible existence of intermolecular or intramolecular TSIs in these TBE derivatives, the single molecular photophysical behavior in the solid state was also investigated. TBE, TBE-M and TBE-2M were doped into a rigid matrix of poly (methyl methacrylate) (PMMA) at a doping concentration of 1 wt%. The emission spectra of TBE@PMMA showed two emissive peaks of approximately 309 and 384 nm (Fig. S24a). The shorter-wavelength peak is ascribed to the emission of the styryl unit, and the longer-wavelength peak at 384 nm suggested the weak intramolecular TSCT property. Similarly, TBE-M@PMMA and TBE-2M@PMMA also showed two emissive peaks, as shown in Fig. S24b and S24c; the through bond emission (styrene emission) for both TBE-M and TBE-2M were observed at approximately 309 nm, while the intramolecular TSI emissions were observed at around 387 nm for TBE-M and 371 nm for TBE-2M, respectively. Further increasing the doping concentration to 60%, the TBE-M@PMMA and TBE-2M@PMMA exhibited an orange emission peak compared to TBE@PMMA, which was consistent with that observed in their crystalline states (Figs S6 and S25). These results suggested that intermolecular TSIs among TBE-M and TBE-2M molecules contribute to their respective long-wavelength emissions. The concentration-dependent PL spectra at 313 and 77 K were also recorded for the three compounds TBE, TBE-M and TBE-2M. As shown in Fig. S26a–c, at 313 K, they exhibited a single emission peak at a wavelength of 309 nm. However, when molecular motion was constrained at 77 K in solution, new peaks emerged at approximately 425 nm for TBE, 418 nm for TBE-M, and 380 nm for TBE-2M, indicating the presence of intramolecular TSIs (Fig. S26d–f).

Since the double bond of the styrene unit plays a significant role in bridging the intermolecular TSI, three TBE derivatives with a phenyl ring and double bond modifications were also investigated (Fig. 6b–d), named TBE-O (**3ja**), TBE-OPh (**3bq**) and TBE-E (**3bk**), with their purity confirmed by HPLC (Fig. S27). Their absorption spectra are shown in Fig. S28. The photographs of their powders under daylight and 290 nm UV are shown in Fig. S29; they exhibited white to pale yellow powdered solids in the solid state while distinct fluorescence emissions, with colors spanning from approximately 580 nm (orange) to 640 nm (red). Interestingly, TBE-O, with modifications on its non-styrene fragment, showed no changes in its maximum emission. While modifying the styrene unit, the emission spectra of TBE-OPh and TBE-E showed longer-wavelength peaks at 620 and 640 nm in the solid state, respectively, surpassing the emission wavelength observed for TBE-M and TBE-2M. The crystal structures of TBE-OPh and TBE-E were successfully obtained, which enabled the further investigation of the structure–property relationship (Fig. S30). In general, neither of the two molecules exhibited dense packing, with intermolecular centroid-to-centroid distances ranging from 5.032 to 6.165 Å, indicating the absence of strong π–π interactions. Notably, the intermolecular C=C⋯C=C distances in TBE-OPh (3.64 Å) and TBE-E (3.663 Å) were comparatively short, enabling potential intermolecular electron delocalization through the double bond of the styrene unit. Furthermore, ONIOM calculations were performed to explore their TSI in the excited states. Indeed, the HOMO and LUMO distributions of the TBE-OPh and TBE-E dimers were found to be delocalized across adjacent styryl units (Fig. S31), emphasizing the important role of the styrene unit. The calculated energy gaps of the dimers were smaller than those of the corresponding monomers, consistent with their longer-wavelength emission. These results support the conclusion that the styrene double bond plays a significant role in facilitating intermolecular TSIs. Also, the picture captured by the IVIS imaging system at an emission wavelength beyond 900 nm for TBE-E under 254 nm irradiation exhibited a strong fluorescence signal (Fig. 6d). This observation indicated that these non-conjugated systems with ultra-large Stokes shifts are promising candidates for advanced detection or imaging applications [50–53].

## DISCUSSION

In summary, a library of non-conjugated hydrocarbons with tunable fluorescence from blue to yellow, orange and, remarkably, even to single-compound-based warm white light has been efficiently constructed by dinickel-catalyzed 1,2-hydroarylation of 1,1-diarylallenes with arylboronic acids. This work demonstrates that non-conjugated pure hydrocarbons can achieve a broad spectrum of colors, not only fundamentally breaking the blue limit of reported hydrocarbons but also advancing the understanding of the non-typical luminescence phenomenon of non-conjugated systems. By the systematic investigation of the photophysical properties of the non-conjugated systems, it has been disclosed that the molecular twisting-tuned intramolecular TSI combined with a double bond-bridged intermolecular TSI is responsible for achieving the ultralong-wavelength fluorescence of these non-conjugated hydrocarbons. Further strategic substitution of the bridged styrene unit with simple functional groups generally extends the fluorescence into the longer deep-red region, even with a tail reaching 900 nm, which could be detected by an NIR-II imaging system. The facile synthesis, simple structures and long visible light emission of these non-typical luminescent systems enable their potential applications for advanced optoelectronics and bioimaging. Moreover, this work provides a new design strategy for luminescent materials beyond the traditional TBC paradigm.

## MATERIALS AND METHODS

### Procedure for 1,2-hydroarylation of allenes

Under an argon atmosphere, arylboronic acid (0.80 mmol, 4.0 equiv.), dinuclear nickel complex (5.4 mg, 2.5 mol%) and NaOEt (54.4 mg, 4.0 equiv.) were added to an oven-dried 10 mL Schlenk tube equipped with a magnetic stir bar. The Schlenk tube was then moved to a glovebox, and allene (0.20 mmol, 1.0 equiv.), *n-*PrOH (1.0 mL), THF (2.5 mL) and NaBHEt_3_ (40 μL, 20.0 mol%, 1.0 M in THF) were added sequentially. After that, the tube was sealed and moved out of the glovebox. The reaction mixture was stirred at room temperature for 1.0 h, then heated to 80°C and stirred for 8.0 h. After cooling to room temperature, saturated brine was added and the reaction mixture was extracted with ethyl acetate. The organic layer was concentrated in vacuum and the residue was purified by chromatography on silica gel, eluting with the mixture of ethyl acetate/hexane to give the 1,2-hydroarylation product.

## Supplementary Material

nwag257_Supplemental_File

## Data Availability

The data supporting the findings of this study are available within the article and Supplementary data files, and are also available from the corresponding author upon request. Crystallographic data coordinates for structures reported in this article have been deposited at the Cambridge Crystallographic Data Center (CCDC), under the deposition numbers CCDC 2405806 (product **3aa**), CCDC 2405805 (product **3ba**), CCDC 2405804 (product **3ca**), CCDC 2473694 (product **3ja**), CCDC 2473696 (product **3bk**) and CCDC 2473695 (product **3bq**). These data can be obtained free of charge from the CCDC via https://www.ccdc.cam.ac.uk/structures/. Source data are provided with this paper.

## References

[1] Xie Z, Liu D, Gao C et al. High mobility emissive organic semiconductors for optoelectronic devices. J Am Chem Soc 2025; 147: 2239–56. 10.1021/jacs.4c11208 39792593

[2] Ostroverkhova O . Organic optoelectronic materials: mechanisms and applications. Chem Rev 2016; 116: 13279–412. 10.1021/acs.chemrev.6b00127 27723323

[3] Lee KW, Wan Y, Huang Z et al. Organic optoelectronic materials: a rising star of bioimaging and phototherapy. Adv Mater 2024; 36: 2306492. 10.1002/adma.202306492 37595570

[4] Hwang J, Nagaraju P, Cho MJ et al. Aggregation-induced emission luminogens for organic light-emitting diodes with a single-component emitting layer. Aggregate 2023; 4: e199. 10.1002/agt2.199

[5] Sharma A, Verwilst P, Li M et al. Theranostic fluorescent probes. Chem Rev 2024; 124: 2699–804. 10.1021/acs.chemrev.3c00778 38422393 PMC11132561

[6] Cheng P, Pu K. Molecular imaging and disease theranostics with renal-clearable optical agents. Nat Rev Mater 2021; 6: 1095–113. 10.1038/s41578-021-00328-6

[7] Hong G, Antaris AL, Dai H. Near-infrared fluorophores for biomedical imaging. Nat Biomed Eng 2017; 1: 0010. 10.1038/s41551-016-0010

[8] Gonçalves MST . Fluorescent labeling of biomolecules with organic probes. Chem Rev 2009; 109: 190–212. 10.1021/cr0783840 19105748

[9] Kobayashi H, Ogawa M, Alford R et al. New strategies for fluorescent probe design in medical diagnostic imaging. Chem Rev 2010; 110: 2620–40. 10.1021/cr900263j 20000749 PMC3241938

[10] Yang Z, Sharma A, Qi J et al. Super-resolution fluorescent materials: an insight into design and bioimaging applications. Chem Soc Rev 2016; 45: 4651–67. 10.1039/C5CS00875A 27296269

[11] Zhang J, He B, Hu Y et al. Stimuli-responsive AIEgens. Adv Mater 2021; 33: 2008071. 10.1002/adma.202008071 34137087

[12] Lou K, Hu Z, Zhang H et al. Information storage based on stimuli-responsive fluorescent 3D code materials. Adv Funct Mater 2022; 32: 2113274. 10.1002/adfm.202113274

[13] Li J, Duan H, Pu K. Nanotransducers for near-infrared photoregulation in biomedicine. Adv Mater 2019; 31: 1901607. 10.1002/adma.201901607 31199021

[14] Lei Z, Sun C, Pei P et al. Stable, wavelength-tunable fluorescent dyes in the NIR-II region for in vivo high-contrast bioimaging and multiplexed biosensing. Angew Chem Int Ed 2019; 58: 8166–71. 10.1002/anie.201904182 31008552

[15] Hou J, Park M-H, Zhang S et al. Bandgap and molecular energy level control of conjugated polymer photovoltaic materials based on benzo[1,2-*b*:4,5-*b*']dithiophene. Macromolecules 2008; 41: 6012–8. 10.1021/ma800820r

[16] Wiberg KB, Frisch MJ. Effect of conjugation on electron distributions. Separation of σ and π terms. J Chem Theory Comput 2016; 12: 1220–7. 10.1021/acs.jctc.5b01149 26845247

[17] Yuan J, Yang H, Huang W et al. Design strategies and applications of cyanine dyes in phototherapy. Chem Soc Rev 2025; 54: 341–66. 10.1039/D3CS00585B 39576179

[18] Hoffmann R . Interaction of orbitals through space and through bonds. Acc Chem Res 1971; 4: 1–9. 10.1021/ar50037a001

[19] Yamaguchi Y, Matsubara Y, Ochi T et al. How the π conjugation length affects the fluorescence emission efficiency. J Am Chem Soc 2008; 130: 13867–9. 10.1021/ja8040493 18816053

[20] Zhu T, Yang T, Zhang Q et al. Clustering and halogen effects enabled red/near-infrared room temperature phosphorescence from aliphatic cyclic imides. Nat Commun 2022; 13: 2658. 10.1038/s41467-022-30368-7 35551197 PMC9098632

[21] Zhang X, Bai Y, Deng J et al. Effects of nonaromatic through-bond conjugation and through-space conjugation on the photoluminescence of nontraditional luminogens. Aggregate 2024; 5: e517. 10.1002/agt2.517

[22] Zhang H, Zheng X, Xie N et al. Why do simple molecules with “isolated” phenyl rings emit visible light? J Am Chem Soc 2017; 139: 16264–72. 10.1021/jacs.7b08592 29064249

[23] Zhang J, Xiong Z, Zhang H et al. Emergent clusteroluminescence from nonemissive molecules. Nat Commun 2025; 16: 3910. 10.1038/s41467-025-59212-4 40280920 PMC12032425

[24] Zhang J, Hu L, Zhang K et al. How to manipulate through-space conjugation and clusteroluminescence of simple AIEgens with isolated phenyl rings. J Am Chem Soc 2021; 143: 9565–74. 10.1021/jacs.1c03882 34115474

[25] Wang L, Xiong Z, Sun JZ et al. How the length of through-space conjugation influences the clusteroluminescence of oligo(phenylene methylene)s. Angew Chem Int Ed 2024; 63: e202318245. 10.1002/anie.202318245 38165147

[26] Xiong Z, Zhang J, Sun JZ et al. Excited-state odd–even effect in through-space interactions. J Am Chem Soc 2023; 145: 21104–13. 10.1021/jacs.3c08164 37715315

[27] Zhang Z, Xiong Z, Chu B et al. Manipulation of clusteroluminescence in carbonyl-based aliphatic polymers. Aggregate 2022; 3: e278. 10.1002/agt2.278

[28] Xiao L-J, Cheng L, Feng W-M et al. Nickel(0)-catalyzed hydroarylation of styrenes and 1,3-dienes with organoboronic compounds. Angew Chem Int Ed 2018; 57: 461–4. 10.1002/anie.201710735 29159912

[29] Bhakta S, Ghosh T. Nickel-catalyzed hydroarylation reaction: a useful tool in organic synthesis. Org Chem Front 2022; 9: 5074–103. 10.1039/D2QO00826B

[30] Pagès L, Abed Ali Abdine R, Monnier F et al. Transition metal-catalyzed intermolecular hydroarylation of allenes. Eur J Org Chem 2022; 41: e202200724. 10.1002/ejoc.202200724

[31] Ma S, Jiao N, Ye L. Palladium-catalyzed highly regio- and stereoselective addition of organoboronic acids to allenes in the presence of AcOH. Chem Eur J 2003; 9: 6049–56. 10.1002/chem.200305301 14679517

[32] Oh CH, Ahn TW, Reddy VR. Palladium-catalyzed addition of organoboronic acids to allenes. Chem Commun 2003: 2622–3. 10.1039/b309258e 14594311

[33] Farley CM, Uyeda C. Organic reactions enabled by catalytically active metal-metal bonds. Trends Chem 2019; 1: 497–509. 10.1016/j.trechm.2019.04.002

[34] Uyeda C, Farley CM. Dinickel active sites supported by redox-active ligands. Acc Chem Res 2021; 54: 3710–9. 10.1021/acs.accounts.1c00424 34565142 PMC9667495

[35] Nath BD, Takaishi K, Ema T. Macrocyclic multinuclear metal complexes acting as catalysts for organic synthesis. Catal Sci Technol 2020; 10: 12–34. 10.1039/C9CY01894H

[36] Chen K, Zhu H, Li Y et al. Dinuclear cobalt complex-catalyzed stereodivergent semireduction of alkynes: switchable selectivities controlled by H_2_O. ACS Catal 2021; 11: 13696–705. 10.1021/acscatal.1c04141

[37] Cai Q, Rao H, Li S-J et al. Well-defined chiral dinuclear copper complexes in enantioselective propargylic substitution: for a long-standing supposition on binuclear mechanism. Chem 2024; 10: 265–82. 10.1016/j.chempr.2023.09.006

[38] Chen K, Zhu H, Liu S et al. Switch in selectivities by dinuclear nickel catalysis: 1,4-hydroarylation of 1,3-dienes to *Z*-olefins. J Am Chem Soc 2023; 145: 24877–88. 10.1021/jacs.3c09283 37903244

[39] Chen K, Zhu H, Jiang S et al. Alkyne dimerization-hydroarylation to form pentasubstituted 1,3-dienes via binuclear nickel catalysis. Nat Commun 2025; 16: 3077. 10.1038/s41467-025-58398-x 40159503 PMC11955554

[40] Berns RS . Designing white-light LED lighting for the display of art: a feasibility study. Color Res Appl 2011; 36: 324–34. 10.1002/col.20633

[41] Li J, Shen P, Zhao Z et al. Through-space conjugation: a thriving alternative for optoelectronic materials. CCS Chem 2019; 1: 181–96. 10.31635/ccschem.019.20180020

[42] Liu D, Wang W-J, Alam P et al. Highly efficient circularly polarized near-infrared phosphorescence in both solution and aggregate. Nat Photonics 2024; 18: 1276–84. 10.1038/s41566-024-01538-4

[43] Ai X, Evans EW, Dong S et al. Efficient radical-based light-emitting diodes with doublet emission. Nature 2018; 563: 536–40. 10.1038/s41586-018-0695-9 30464267

[44] Shao S, Wang L. Through-space charge transfer polymers for solution-processed organic light-emitting diodes. Aggregate 2020; 1: 45–56. 10.1002/agt2.4

[45] Tang X, Cui L-S, Li H-C et al. Highly efficient luminescence from space-confined charge-transfer emitters. Nat Mater 2020; 19: 1332–8. 10.1038/s41563-020-0710-z 32541938

[46] Laurent AD, Jacquemin D. TD-DFT benchmarks: a review. Int J Quantum Chem 2013; 113: 2019–39. 10.1002/qua.24438

[47] Adamo C, Jacquemin D. The calculations of excited-state properties with time-dependent density functional theory. Chem Soc Rev 2013; 42: 845–56. 10.1039/C2CS35394F 23117144

[48] Tirado-Rives J, Jorgensen WL. Performance of B3LYP density functional methods for a large set of organic molecules. J Chem Theory Comput 2008; 4: 297–306. 10.1021/ct700248k 26620661

[49] Goerigk L . A comprehensive overview of the DFT-D3 London-dispersion correction. In: Non-Covalent Interactions in Quantum Chemistry and Physics: Theory and Applications. Amsterdam: Elsevier, 2017, 195–219.

[50] Tian R, Li K, Lin Y et al. Characterization techniques of polymer aging: from beginning to end. Chem Rev 2023; 123: 3007–88. 10.1021/acs.chemrev.2c00750 36802560

[51] Shao H, Li Y, Zhuang J et al. Retinomorphic photonic synapses for mimicking ultraviolet radiation sensing and damage imaging. Adv Funct Mater 2024; 34: 2316381. 10.1002/adfm.202316381

[52] Jiang N, Zhu C, Li K et al. Recent progress in nonconventional luminescent macromolecules and their applications. Macromolecules 2024; 57: 5561–77. 10.1021/acs.macromol.4c00186 38948183 PMC11210344

[53] Guo L, Yan L, He Y et al. Hyperbranched polyborate: a non-conjugated fluorescent polymer with unanticipated high quantum yield and multicolor emission. Angew Chem Int Ed 2022; 61: e202204383. 10.1002/anie.202204383 35499909

