# Supplementary material for: Breaking the blue limit of non-conjugated hydrocarbons via multiple through-space interactions
Source: Natl Sci Rev. 2026 May 7;13(11):nwag257. doi: 10.1093/nsr/nwag257 (PMC13278502; doi:10.1093/nsr/nwag257)
Supplement: nwag257_Supplemental_File [file nwag257_supplemental_file.pdf]

Supplementary Information

Breaking the Blue Limit of Non-Conjugated Hydrocarbons via Multiple Through-Space Interactions

Shuxin Jiang,[†a] Ziwei Deng,[†b] Xiaoming Wang,*[a,c,d] Zheng Zhao,*[b] Ben Zhong Tang[b]

[a] State Key Laboratory of Organometallic Chemistry and Shanghai Hongkong Joint Laboratory in Chemical Synthesis, Shanghai Institute of Organic Chemistry, University of Chinese Academy of Sciences, Chinese Academy of Sciences, 345 Lingling Road, Shanghai 200032, China.

[b] Guangdong Basic Research Center of Excellence for Aggregate Science, School of Science and Engineering, Shenzhen Institute of Aggregate Science and Technology, The Chinese University of Hong Kong (Shenzhen), Longgang, Shenzhen, Guangdong 518172, China.

[c] School of Chemistry and Materials Science, Hangzhou Institute for Advanced Study, University of Chinese Academy of Sciences, 1 Sub-lane Xiangshan, Hangzhou 310024, China.

[d] School of Chemistry and Chemical Engineering, Henan Normal University, Xinxiang, 453007, China.

*Correspondence author. Email: xiaoming@sioc.ac.cn; zhaozheng@cuhk.edu.cn

†S. J. and Z. D. contributed equally to this work.

Table of contents

1. General Information S2

2.General procedure for allene synthesis S2

3. Characterization of the allenes S3

4. Procedures for the preparation of dinuclear nickel complex A S7

5. Optimization of the reaction conditions S8

6. General procedure for 1,2-hydroarylation of allenes S11

7. Characterization of the 1,2-hydroarylation products S12

8. Characterization of photophysical properties S31

9. Crystal data and structure refinement for **3aa** S65

10. Crystal data and structure refinement for **3ba** S66

11. Crystal data and structure refinement for **3ca** S67

12. Crystal data and structure refinement for **3ja** S68

13. Crystal data and structure refinement for **3bq** S69

14. Crystal data and structure refinement for **3bk** S70

15. References S71

16. NMR spectra of the allenes S72

17. NMR spectra of the 1,2-hydroarylation products S85

1. General Information

Unless otherwise noted, all reactions and manipulations were carried out using standard Schlenk, high-vacuum and glovebox techniques. All reagents and solvents used in this study were purchased from commercial sources and used as received. The analytical thin layer chromatography (TLC) was performed on HSGF/UV254 plates. The flash chromatography was performed on Huanghai silica gel (200-300 mesh) by standard techniques eluting with solvents as indicated. NMR spectra were recorded on Bruker 400 MHz, Agilent 400 MHz and Varian 400 MHz at ambient temperature. The residual peak of deuterated solvent was used as a reference for ^{1}H and ^{13}C chemical shifts. The following abbreviations (or combinations thereof) were used to explain the multiplicities: s = singlet, d = doublet, t = triplet, q = quartet, p = quintet, h = sextet, hept = septet, m = multiplet, The IR spectra were measured on a BRUKER TENSOR 27 FT-IR spectrometer. GC analysis was performed on Shimadzu GC-2014 gas chromatograph. HRMS (EI) spectra were obtained on a Waters Micromass GCT Premier spectrometer. HRMS (FI) spectra were obtained on JEOL-AccuTOF-GCv4G-GCT MS. HRMS (ESI) was determined on Bruker APEXIII 7.0 TESLA FTMS spectrometer. HRMS (DART) was determined on Thermo Fisher Scientific LTQ FTICR-MS. Melting points were measured on an electrothermal digital melting point apparatus. The UV-Vis absorption spectra were acquired by a Perkin Elmer Lambda 365. Photoluminescence spectra were obtained by Edinburgh FLS1000 luminescence spectrometer. In vivo fluorescence imaging system (IVIS) was obtained by Suzhou NIR-Optics Technology Co., Ltd, China.

2. General procedure for allene synthesis.[1]

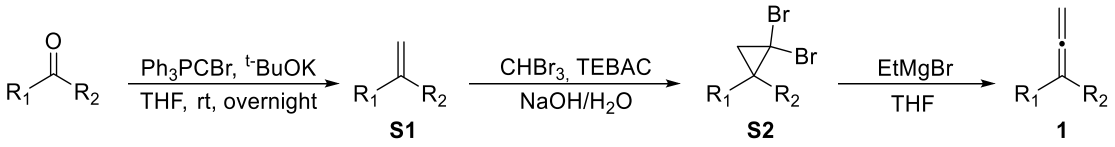

Step 1: In an oven dried flask, was added methyl triphenylphosphonium bromide (1.2 equiv) followed by THF (2.5 mmol /mL). Then ^{t}BuOK (1.2 equiv) was added and the resulting yellow suspension was stirred at room temperature for 60 min. To this suspension, a solution of ketone (1.0 equiv) was added in one portion and the resulting mixture was further stirred at room temperature overnight. Water and DCM were added to the reaction mixture, and the aqueous phase was extracted with DCM (3 × 50 mL). The combined organic phases were washed with saturated NaCl solution, dried over Na_2SO_4 and the solvent was removed under reduced pressure. The reaction mixture was purified by column chromatography over silica gel (200-300 mesh) using hexanes as eluent to afford **S1**.

Step 2: To a solution of alkene **S1** (1.0 equiv), bromoform (1.5 equiv) and $BnNEt_3Cl$ (1 mol%) was added dropwise a solution of 50% NaOH (aq.), and the mixture was stirred at room temperature for 60 min, then heated to 60 °C and further stirred until conversion was complete as observed by TLC analysis. Water and DCM were added and the aqueous phase was extracted with DCM (3 x 50 mL). The combined organic phases were washed with saturated NaCl solution, dried over Na_2SO_4 and the solvent was removed under reduced pressure. The reaction mixture was purified by column chromatography to afford **S2**.

Step 3: EtMgBr (3.0 M in ether, 1.5 equiv) was added dropwise to a pre-cooled (ice-bath) solution

of **S2** (1.0 equiv) in dry THF (1.0 mL/mmol) under nitrogen atmosphere. After EtMgBr was added the mixture was then slowly warmed to room temperature, and stirred at room temperature for an additional 2 hours. Then the reaction was quenched by HCl (0.5 N, 10 ml) solution, water was added, and the mixture was extracted with ether (3 x 50 mL). The combined organic layers were washed with brine, dried with anhydrous Na_2SO_4 and filtered. After removing the solvent under reduced pressure, the crude product was purified by column chromatography on silica gel to afford allenes.

3. Characterization of the allenes

2,2'-(propa-1,2-diene-1,1-diyl)bis(methylbenzene) (1b)

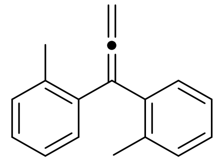

White solid, m. p. 68.2-70.3 °C.

^{1}H NMR (400 MHz, $CDCl_3$) δ 7.23 – 7.07 (m, 8H), 5.04 (s, 2H), 2.28 (s, 6H) ppm; ^{13}C NMR (100 MHz, $CDCl_3$) δ 209.1, 136.9, 136.4, 130.7, 129.4, 127.2, 125.9, 106.3, 75.9, 20.6 ppm; IR(neat) ν 3057, 3015, 2957, 2924, 1929, 1484, 1455, 1042, 913, 859, 756, 723, 629, 449 cm^{-1}; HRMS-EI (m/z) M$^+$ calcd. for $C_{17}H_{16}$ 220.1247; found 220.1241.

2,2'-(propa-1,2-diene-1,1-diyl)bis(1,4-dimethylbenzene) (1c)

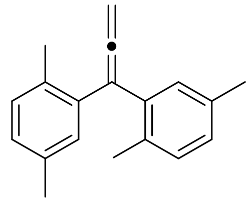

White solid, m. p. 39.6-42.4 °C.

^{1}H NMR (400 MHz, $CDCl_3$) δ 7.08 (d, J = 7.7 Hz, 2H), 6.99 (d, J = 7.8 Hz, 2H), 6.90 (s, 2H), 4.99 (s, 2H), 2.27 (s, 6H), 2.22 (s, 6H) ppm; ^{13}C NMR (100 MHz, $CDCl_3$) δ 209.0, 136.7, 135.3, 133.3, 130.6, 129.9, 127.9, 106.3, 75.7, 20.9, 20.1 ppm; IR(neat) ν 3016, 2920, 1928, 1495, 1451, 883, 854, 804, 745, 723, 658, 525, 485, 453 cm^{-1}; HRMS-EI (m/z) M$^+$ calcd. for $C_{19}H_{20}$ 248.1560; found 248.1563.

3,3'-(propa-1,2-diene-1,1-diyl)bis(1,2-dimethylbenzene) (1d)

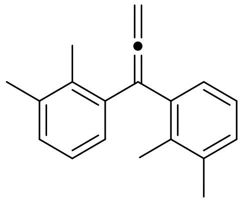

Green oil.

^{1}H NMR (400 MHz, $CDCl_3$) δ 7.09 (d, J = 5.9 Hz, 2H), 7.05 (t, J = 7.5 Hz, 2H), 6.97 (d, J = 7.4 Hz, 2H), 4.98 (s, 2H), 2.32 (s, 6H), 2.24 (s, 6H) ppm; ^{13}C NMR (100 MHz, $CDCl_3$) δ 209.0, 137.7, 137.3, 134.8, 128.8, 127.3, 125.3, 106.7, 75.3, 20.8, 16.7 ppm; IR(neat) ν 2914, 1937, 1455, 1288, 841, 783, 737, 722, 613, 439 cm^{-1}; HRMS-EI (m/z) M$^+$ calcd. for $C_{19}H_{20}$ 248.1560; found 248.1562.

4,4'-(propa-1,2-diene-1,1-diyl)bis(1,3-dimethylbenzene) (1e)

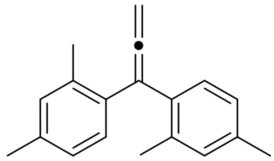

White solid, m. p. 36.5-38.1 °C.

^{1}H NMR (400 MHz, $CDCl_3$) δ 7.19 – 7.05 (m, 6H), 5.11 (s, 2H), 2.44 (s, 6H), 2.39 (s, 6H) ppm; ^{13}C NMR (100 MHz, $CDCl_3$) δ 209.1, 136.7, 136.2, 134.1, 131.4, 129.3, 126.6, 105.9, 75.6, 21.0, 20.5 ppm; IR(neat) ν 3010, 2918, 1937, 1497, 1447, 1376, 870, 846, 815, 730, 589, 568, 442 cm^{-1}; HRMS-EI (m/z) M^+ calcd. for $C_{19}H_{20}$ 248.1560; found 248.1562.

5,5'-(propa-1,2-diene-1,1-diyl)bis(1,3-dimethylbenzene) (1f)

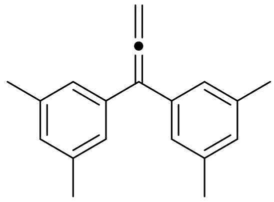

Dark blue oil.

^{1}H NMR (400 MHz, $CDCl_3$) δ 7.01 (s, 4H), 6.95 (s, 2H), 5.24 (s, 2H), 2.33 (s, 12H) ppm; ^{13}C NMR (100 MHz, $CDCl_3$) δ 209.7, 137.8, 136.3, 128.9, 126.2, 109.2, 77.5, 21.3 ppm; IR(neat) ν 2914, 1935, 1597, 1446, 847, 730, 699, 446 cm^{-1}; HRMS-EI (m/z) M^+ calcd. for $C_{19}H_{20}$ 248.1560; found 248.1562.

2-(1-(*o*-tolyl)propa-1,2-dien-1-yl)-1,1'-biphenyl (1h)

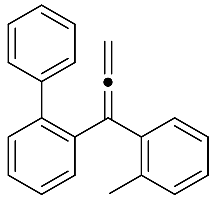

Yellow oil.

^{1}H NMR (400 MHz, $CDCl_3$) δ 7.31 – 7.17 (m, 9H), 7.08 – 6.97 (m, 4H), 4.53 (s, 2H), 2.20 (s, 3H) ppm; ^{13}C NMR (100 MHz, Deuterium Oxide) δ 207.9, 139.5, 139.1, 134.1, 134.0, 133.9, 128.1, 127.9, 127.5, 126.9, 126.5, 125.2, 124.8, 124.7, 124.4, 123.8, 123.1, 103.3, 74.8, 74.5, 74.2, 73.2, 18.2 ppm; IR(neat) ν 3018, 2923, 1937, 1474, 1435, 913, 842, 759, 742, 723, 698, 645, 614, 527, 464 cm^{-1}; HRMS-EI (m/z) M^+ calcd. for $C_{22}H_{18}$ 282.1403; found 282.1406.

1-methyl-2-(1-(2-(trifluoromethyl)phenyl)propa-1,2-dien-1-yl)benzene (1i)

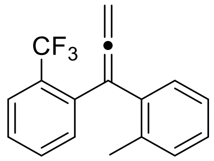

Colorless oil.

^{1}H NMR (400 MHz, $CDCl_3$) δ 7.78 (d, J = 6.5 Hz, 1H), 7.53 (t, J = 7.6 Hz, 1H), 7.43 (t, J = 7.6 Hz, 1H), 7.37 (d, J = 7.7 Hz, 1H), 7.28 (d, J = 7.5 Hz, 1H), 7.23 (t, J = 7.3 Hz, 2H), 7.17 (t, J = 6.5 Hz,

1H), 7.04 (d, J = 7.6 Hz, 1H), 5.14 (s, 2H), 2.46 (s, 3H) ppm; ^{13}C NMR (100 MHz, $CDCl_3$) δ 209.4, 137.3, 136.6, 136.0, 132.0, 131.6, 131.0, 129.0, 128.4 (q, J = 30.4 Hz), 127.3, 127.3, 126.7 (q, J = 5.5 Hz), 125.8, 124.2 (q, J = 274.2 Hz), 103.9, 77.0, 20.9 ppm; ^{19}F NMR (376 MHz, $CDCl_3$) δ -57.2 (s, 3F) ppm; IR(neat) ν 3019, 2927, 1941, 1602 1578, 1489, 1448, 1311, 1164, 1128, 1109, 1055, 1034, 915, 848, 759, 723, 632, 698, 543, 455 cm^{-1}; HRMS-EI (m/z) M$^+$ calcd. for $C_{17}H_{13}F_3$ 274.0964; found 274.0966.

2,2'-(propa-1,2-diene-1,1-diyl)bis(methoxybenzene) (1j)

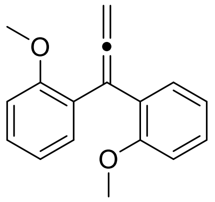

Yellow oil.

^{1}H NMR (400 MHz, $CDCl_3$) δ 7.22 (t, J = 8.6 Hz, 2H), 7.11 (d, J = 7.6 Hz, 2H), 6.92 – 6.83 (m, 4H), 5.01 (s, 2H), 3.70 (s, 6H) ppm; ^{13}C NMR (100 MHz, $CDCl_3$) δ 210.9, 157.0, 130.1, 128.2, 126.6, 120.5, 111.5, 101.0, 74.5, 55.6 ppm; IR(neat) ν 2946, 1936, 1593, 1579, 1488, 1457, 1431, 1255, 1235, 1024, 913, 852, 752, 624, 580 cm^{-1}; HRMS-EI (m/z) M$^+$ calcd. for $C_{17}H_{16}O_2$ 248.1145; found 248.1142.

5,5'-(propa-1,2-diene-1,1-diyl)bis(1,2,4-trimethylbenzene) (1l)

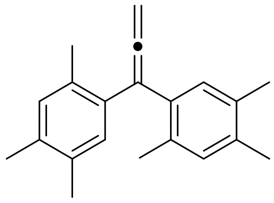

White solid, m. p. 100.1-102.2 °C.

^{1}H NMR (400 MHz, $CDCl_3$) δ 6.97 (s, 2H), 6.86 (s, 2H), 4.95 (s, 2H), 2.24 (s, 6H), 2.22 (s, 6H), 2.18 (s, 6H) ppm; ^{13}C NMR (100 MHz, $CDCl_3$) δ 209.0, 135.3, 134.4, 133.8, 133.6, 132.0, 130.4, 105.9, 75.4, 20.0, 19.3, 19.2 ppm; IR(neat) ν 2918, 1932, 1496, 1455, 1021, 1000, 873, 848, 760, 727, 606, 558, 455, 444 cm^{-1}; HRMS-EI (m/z) M$^+$ calcd. for $C_{21}H_{24}$ 276.1873; found 276.1870.

5-(1-(*o*-tolyl)propa-1,2-dien-1-yl)-1,2,3,4-tetrahydronaphthalene (1m)

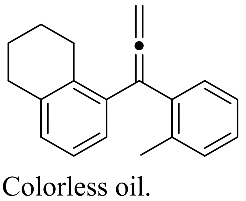

Colorless oil.

^{1}H NMR (400 MHz, $CDCl_3$) δ 7.24 – 6.97 (m, 7H), 5.01 (s, 2H), 2.87 – 2.78 (m, 2H), 2.66 – 2.56 (m, 2H), 2.34 (s, 3H), 1.80 – 1.70 (m, 4H) ppm; ^{13}C NMR (100 MHz, $CDCl_3$) δ 208.9, 137.7, 137.2, 136.8, 136.4, 135.6, 130.7, 129.1, 128.5, 127.2, 127.0, 125.9, 125.3, 106.0, 75.7, 30.0, 27.5, 23.4, 22.8, 20.8ppm; IR(neat) ν 3016, 2925, 1929, 1485, 1451, 843, 779, 759, 722, 666, 634, 436 cm^{-1}; HRMS-EI (m/z) M$^+$ calcd. for $C_{20}H_{20}$ 260.1560; found 260.1564.

1-(1-(*o*-tolyl)propa-1,2-dien-1-yl)naphthalene (1n)

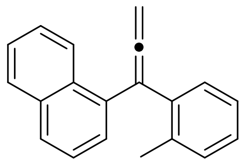

Colorless oil.

^{1}H NMR (400 MHz, $CDCl_3$) δ 8.17 (d, J = 9.1 Hz, 1H), 7.88 (d, J = 7.4 Hz, 1H), 7.81 (d, J = 7.7 Hz, 1H), 7.52 – 7.40 (m, 3H), 7.34 (d, J = 7.2 Hz, 1H), 7.25 – 7.10 (m, 4H), 5.13 (s, 2H), 2.34 (s, 3H) ppm; ^{13}C NMR (100 MHz, $CDCl_3$) δ 210.0, 137.2, 136.4, 135.3, 134.1, 131.3, 130.8, 129.6, 128.5, 127.9, 127.3, 127.1, 126.0, 126.0, 125.7, 125.5, 125.4, 105.4, 77.3, 77.0, 76.7, 76.1, 20.9 ppm; IR(neat) ν 3012, 2922, 1935, 1592, 1505, 1392, 844, 800, 776, 723, 619, 429 cm^{-1}; HRMS-EI (m/z) M^+ calcd. for $C_{20}H_{16}$ 256.1247; found 256.1240.

2-(1-(*o*-tolyl)propa-1,2-dien-1-yl)naphthalene (1o)

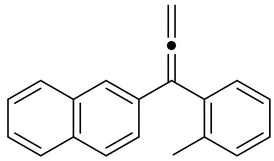

Yellow solid, m. p. 91.7-93.3 °C.

^{1}H NMR (400 MHz, $CDCl_3$) δ 7.86 – 7.76 (m, 2H), 7.70 (d, J = 8.9 Hz, 1H), 7.61 (d, J = 8.7 Hz, 1H), 7.49 – 7.39 (m, 3H), 7.34 (d, J = 5.4 Hz, 4H), 5.28 (s, 2H), 2.26 (s, 3H) ppm; ^{13}C NMR (100 MHz, $CDCl_3$) δ 208.86, 137.1, 135.4, 133.7, 133.5, 132.4, 130.4, 130.3, 128.0, 127.8, 127.5, 126.1, 126.0, 125.7, 125.2, 125.1, 107.5, 77.9, 20.0 ppm; IR(neat) ν 3011, 2917, 1929, 1502, 1456, 894, 862, 819, 751, 724, 673, 584, 468 cm^{-1}; HRMS-EI (m/z) M^+ calcd. for $C_{20}H_{16}$ 256.1247; found 256.1248.

4. Procedures for the preparation of dinuclear nickel complex A

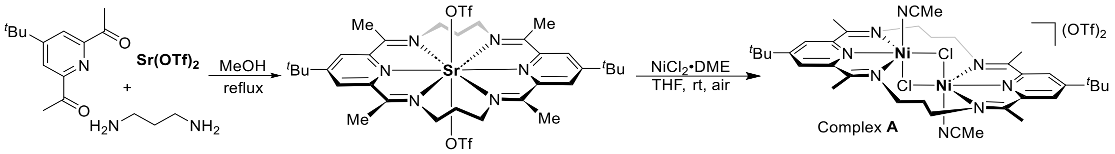

Synthesis of (^{3}PDI$_2$)Sr(OTf)$_2$: A solution of 1,3-diaminopropane (338 mg, 4.56 mmol) in 10 mL of methanol was added to a methanol (30.0 mL) solution of Sr(OTf)$_2$ (880 mg, 2.28 mmol) and 4-*tert*-butyl-2,6-diacetylpyridine (1.0 g, 4.56 mmol) at room temperature. The resulting yellow solution was refluxed for 24.0 h. After cooling the reaction mixture to room temperature, volatile materials were removed under reduced pressure. The residue was dissolved in *ca.* 3.0 mL of DCM and layered with 15.0 mL of hexane, then stored at -20 °C to afford (^{3}PDI$_2$)Sr(OTf)$_2$ as an off-white crystalline solid. Yield: 1.68 g (82%). ^{1}H NMR (400 MHz, $CDCl_3$) δ 7.81 (s, 4H), 3.95 (t, *J* = 6.4 Hz, 8H), 2.45 (s, 12H), 1.88 (p, *J* = 6.5 Hz, 4H), 1.42 (s, 18H) ppm; ^{13}C NMR (100 MHz, $CDCl_3$) δ 167.0, 163.9, 155.4, 121.3, 46.5, 35.4, 30.5, 28.7, 15.1 ppm; ^{19}F NMR (376 MHz, $CDCl_3$) δ -78.7 ppm. The NMR spectra were consistent with the spectra reported in the literature[2].

Synthesis of dinuclear nickel complex A:[3] $NiCl_2$·DME (440 mg, 2.0 mmol) and (^{3}PDI$_2$)Sr(OTf)$_2$ (900 mg, 1.0 mmol) were mixed in 30.0 mL of THF at room temperature under air. After stirring for 2.0 h, the green slurry was formed, which was filtered directly to get a light green solid containing Sr salt. Then these green solid was dissolved in hot CH_3CN and the Sr salt can be removed by filtration. After that, the resulting green solution was stored at -20 °C to afford the dinuclear nickel complex **A** as a green solid. Yield: 737 mg (67%).

Dinuclear nickel complexes **B** and **C** were synthesized according to the literature.[4]

5. Optimization of the reaction conditions

Table S1. Screening of reductants[a]

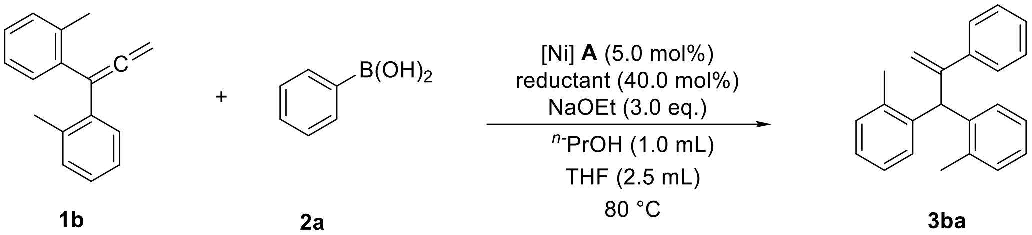

entry	reductant	conv. (%)[b]	**3ba** (%)[b]
1	$NaBHEt_3$	>99	78
2	$NaBH(OAc)_3$	26	15
3	$NaBH_3CN$	>99	35
4	LiBH(*s*-Bu)$_3$	37	8
5	Synhydrid®	80	62
6	DIBAL-H	85	67

[a]Reaction conditions: **1b** (0.20 mmol), **2a** (0.60 mmol), dinickel complex **A** (5.0 mol%), reductant (40.0 mol%) and NaOEt (0.60 mmol) in ^{n}-PrOH (1.0 mL) / THF (2.5 mL) at 80 °C for 8.0 h. [b]Conversion and yields were determined by GC using mesitylene as the internal standard.

Table S2. Screening of ether-solvents[a]

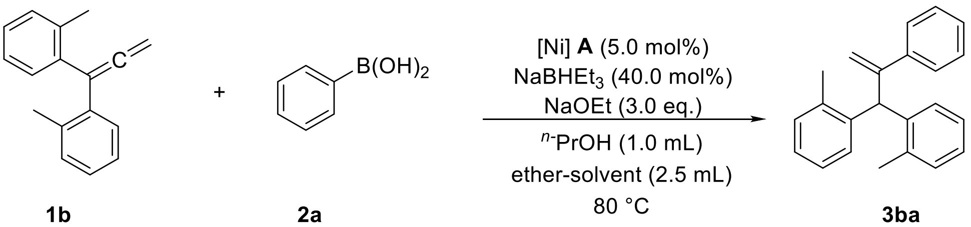

entry	solvent	conv. (%)[b]	**3ba** (%)[b]
1	Diglyme	12	trace
2	DME	5	3
3	^{n}-Bu_2O	70	48
4	^{i}-Pr_2O	77	43
5	^{t}-BuOMe	84	50
6	THF	>99	78
7	2-Methyltetrahydrofuran	89	71

[a]Reaction conditions: **1b** (0.20 mmol), **2a** (0.60 mmol), dinickel complex **A** (5.0 mol%), $NaBHEt_3$ (40.0 mol%) and NaOEt (0.60 mmol) in ^{n}-PrOH (1.0 mL)/ether-solvent (2.5 mL) at 80 °C for 8.0 h. [b]Conversion and yields were determined by GC using mesitylene as the internal standard.

Table S3. Screening of alcohols[a]

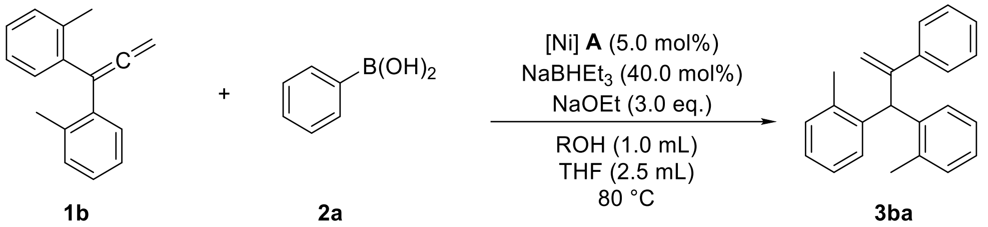

entry	alcohol	conv. (%)[b]	**3ba** (%)[b]
1	MeOH	18	7
2	EtOH	>99	36
3	^{i}-PrOH	87	58
4	^{n}-PrOH	>99	78
5	^{t}-BuOH	>99	21
6	^{t}-PentylOH	48	3

[a]Reaction conditions: **1b** (0.20 mmol), **2a** (0.60 mmol), dinickel complex **A** (5.0 mol%), $NaBHEt_3$ (40.0 mol%) and NaOEt (0.60 mmol) in ROH (1.0 mL) / THF (2.5 mL) at 80 °C for 8.0 h. [b] Conversion and yields were determined by GC using mesitylene as the internal standard.

Table S4. Screening of $PhB(OH)_2$ loading[a]

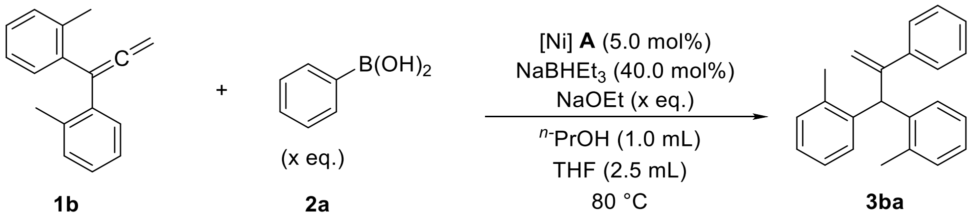

entry	$PhB(OH)_2$ (x eq.)	conv. (%)[b]	**3ba** (%)[b]
1	2	87	60
2	2.5	>99	73
3	3	>99	78
4	3.5	>99	85
5	4	>99	87
6	4.5	>99	87
7	5	>99	86

[a]Reaction conditions: **1b** (0.20 mmol), **2a** (x eq.), dinickel complex **A** (5.0 mol%), $NaBHEt_3$ (40.0 mol%) and NaOEt (x eq.) in ^{n}-PrOH (1.0 mL) / THF (2.5 mL) at 80 °C for 8.0 h. [b] Conversion and yields were determined by GC using mesitylene as the internal standard.

Table S5. Screening of temperature[a]

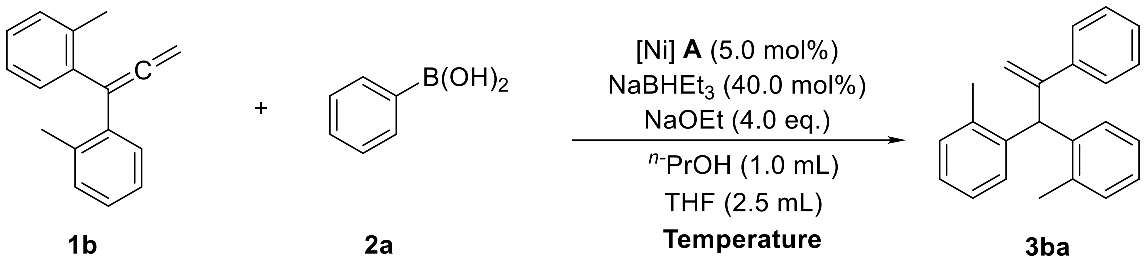

entry	Temperature / °C	conv. (%)[b]	**3ba** (%)[b]
1	60	83	71
2	70	>99	78
3	80	>99	87
4	90	>99	83

[a]Reaction conditions: **1b** (0.20 mmol), **2a** (0.80 mmol.), dinickel complex **A** (5.0 mol%), $NaBHEt_3$ (40.0 mol%) and NaOEt (0.80 mmol.) in ^{n}-PrOH (1.0 mL) /THF (2.5 mL) at **Temperature** for 8.0 h. [b] Conversion and yields were determined by GC using mesitylene as the internal standard.

Table S6. Screening of [Ni] **A** loading[a]

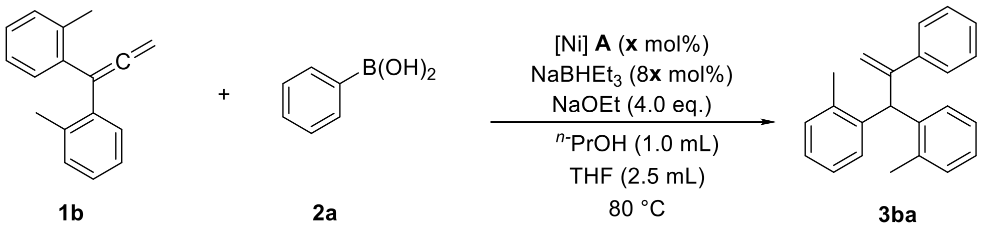

entry	[Ni] **A** (x mol%)	conv. (%)[b]	**3ba** (%)[b]
1	0	0	0
2	2.5	>99	87
3	5	>99	87

[a]Reaction conditions: **1b** (0.20 mmol), **2a** (0.80 mmol.), dinickel complex **A** (**x** mol%), $NaBHEt_3$ (8**x** mol%) and NaOEt (0.80 mmol.) in ^{n}-PrOH (1.0 mL) /THF (2.5 mL) at 80 °C for 8.0 h. [b] Conversion and yields were determined by GC using mesitylene as the internal standard.

Table S7. Screening of nickel catalysts[a]

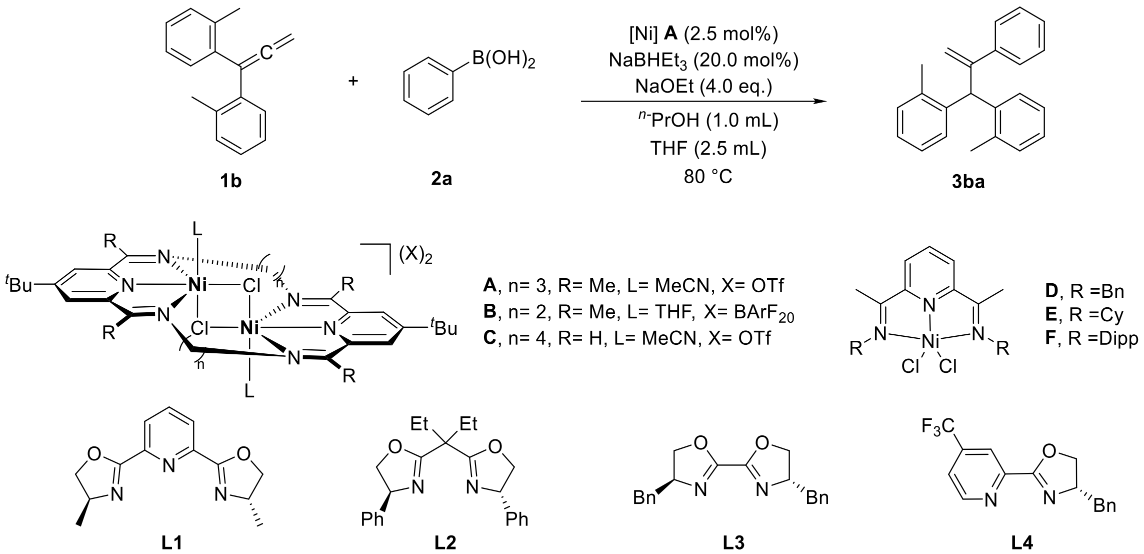

entry	deviation from standard conditions	conv. (%)[b]	**3ba** (%)[b]
1	none	>99	87
2	no $NaBHEt_3$	>99	45
3	no NaOEt	70	24
4	no THF	54	19
5	no ^{n}-PrOH	>99	26
6	**B** instead of **A**	79	31
7	**C** instead of **A**	>99	31
8	**D** instead of **A**	74	22
9	**E** instead of **A**	>99	9
10	**F** instead of **A**	30	3
11	**L1** + $NiCl_2$·DME instead of **A**	43	trace
12	**L2** + $NiCl_2$·DME instead of **A**	54	3
13	**L3** + $NiCl_2$·DME instead of **A**	82	6
14	**L4** + $NiCl_2$·DME instead of **A**	47	trace

[a]Reaction conditions: **1b** (0.20 mmol), **2a** (0.80 mmol.), dinickel complex **A** (2.5 mol%), $NaBHEt_3$ (20 mol%) and NaOEt (0.80 mmol.) in ^{n}-PrOH (1.0 mL) /THF (2.5 mL) at 80 °C for 8.0 h. [b] Conversion and yields were determined by GC using mesitylene as the internal standard.

6. General procedure for 1,2-hydroarylation of allenes

Under argon atmosphere, arylboronic acid (0.80 mmol, 4.0 equiv), dinuclear nickel complex **A** (5.4 mg, 2.5 mol%) and NaOEt (54.4 mg, 4.0 equiv) were added to an oven-dried 10 mL Schlenk tube equipped with a magnetic stir bar. Then the Schlenk tube was moved to glovebox, allene (0.20 mmol, 1.0 equiv), ^{n}-PrOH (1.0 mL), THF (2.5 mL) and $NaBHEt_3$ (40 µL, 20.0 mol%, 1.0 M in THF) were added sequentially. After that, the tube was sealed and moved out of the glovebox. The reaction mixture was stirred at room temperature for 1.0 hour, then heated to 80 °C and stirred for 8.0 hours. After cooling to room temperature, saturated brine was added and the reaction mixture was extracted with ethyl acetate. The organic layer was concentrated in vacuum and the residue was purified by chromatography on silica gel, eluting with the mixture of ethyl acetate/hexane to give the 1,2-hydroarylation product.

7. Characterization of the 1,2-hydroarylation products

prop-2-ene-1,1,2-triyltribenzene (3aa)

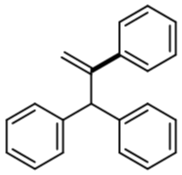

White solid, 17.3 mg, 32 % yield, m. p. 49.6-52.5 °C.

^{1}H NMR (400 MHz, $CDCl_3$) δ 7.47 (dd, *J* = 7.9, 2.6 Hz, 2H), 7.36 – 7.18 (m, 13H), 5.74 (s, 1H), 5.46 (s, 1H), 4.81 (s, 1H) ppm; ^{13}C NMR (100 MHz, $CDCl_3$) δ 150.62, 142.57, 141.76, 129.43, 128.29, 128.20, 127.34, 126.40, 126.34, 117.45, 56.04 ppm.

2,2'-(2-phenylprop-2-ene-1,1-diyl)bis(methylbenzene) (3ba)

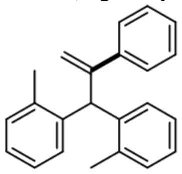

Colorless crystal, 46.5 mg, 78 % yield, m. p. 59.1-61.3 °C.

^{1}H NMR (400 MHz, $CDCl_3$) δ 7.40 (d, *J* = 7.3 Hz, 2H), 7.26 – 7.06 (m, 9H), 7.02 (d, *J* = 7.3 Hz, 2H), 5.67 (s, 1H), 5.50 (s, 1H), 4.66 (s, 1H), 2.24 (s, 6H) ppm; ^{13}C NMR (100 MHz, $CDCl_3$) δ 149.2, 141.9, 140.5, 136.5, 130.3, 128.9, 128.3, 127.4, 126.4, 126.0, 125.8, 117.2, 49.2, 19.5 ppm; IR(neat) ν 3013, 2919, 1599, 1486, 1459, 1031, 906, 779, 744, 698, 600, 448 cm^{-1}; HRMS-EI (m/z) M^+ calcd. for $C_{23}H_{22}$ 298.1716; found 298.1714.

2,2'-(2-phenylprop-2-ene-1,1-diyl)bis(1,4-dimethylbenzene) (3ca)

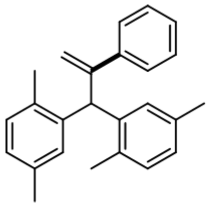

Colorless crystal, 44.0 mg, 67 % yield, m. p. 118.4-119.6 °C.

^{1}H NMR (400 MHz, $CDCl_3$) δ 7.42 (d, *J* = 6.6 Hz, 2H), 7.31 – 7.21 (m, 3H), 7.08 (d, *J* = 7.6 Hz, 2H), 6.97 (d, *J* = 7.6 Hz, 2H), 6.88 (s, 2H), 5.71 (s, 1H), 5.46 (s, 1H), 4.72 (s, 1H), 2.26 (s, 6H), 2.22 (s, 6H) ppm; ^{13}C NMR (100 MHz, $CDCl_3$) δ 149.5, 142.3, 140.3, 135.0, 133.4, 130.2, 129.7, 128.3, 127.3, 127.0, 126.1, 117.1, 49.3, 21.3, 19.2 ppm; IR(neat) ν 3012, 2920, 1620, 1572, 1494, 1442, 1154, 1031, 904, 889, 805, 776, 699, 539, 457, 440 cm^{-1}; HRMS-EI (m/z) M^+ calcd. for $C_{25}H_{26}$ 326.2039; found 326.2031.

3,3'-(2-phenylprop-2-ene-1,1-diyl)bis(1,2-dimethylbenzene) (3da)

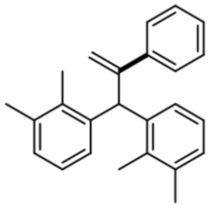

White solid, 32.7 mg, 50 % yield, m. p. 85.6-88.2 °C.

^{1}H NMR (400 MHz, $CDCl_3$) δ 7.49 – 7.43 (m, 2H), 7.33 – 7.28 (m, 2H), 7.28 – 7.23 (m, 1H), 7.09 (d, J = 7.5 Hz, 2H), 7.03 (t, J = 7.5 Hz, 2H), 6.94 (d, J = 7.6 Hz, 2H), 5.73 (d, J = 1.3 Hz, 1H), 5.64 (s, 1H), 4.72 (s, 1H), 2.35 (s, 6H), 2.18 (s, 6H) ppm; ^{13}C NMR (100 MHz, $CDCl_3$) δ 149.8, 142.0, 140.8, 136.6, 134.9, 128.3, 128.2, 127.3, 127.0, 126.0, 125.1, 117.3, 49.9, 20.9, 15.0 ppm; IR(neat) ν 3021,.2945, 1496, 915, 822, 781, 695, 478 cm^{-1}; HRMS-EI (m/z) M$^+$ calcd. for $C_{25}H_{26}$ 326.2029; found 326.2039.

4,4'-(2-phenylprop-2-ene-1,1-diyl)bis(1,3-dimethylbenzene) (3ea)

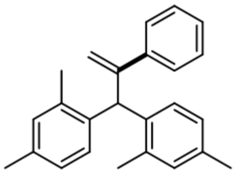

White solid, 43.6 mg, 67 % yield, m. p. 99.7-102.2 °C.

^{1}H NMR (400 MHz, $CDCl_3$) δ 7.51 – 7.44 (m, 2H), 7.34 – 7.24 (m, 3H), 7.05 (s, 2H), 7.01 – 6.93 (m, 4H), 5.72 (d, J = 1.2 Hz, 1H), 5.49 (s, 1H), 4.75 (t, J = 1.3 Hz, 1H), 2.35 (s, 6H), 2.28 (s, 6H) ppm; ^{13}C NMR (100 MHz, $CDCl_3$) δ 149.6, 142.1, 137.6, 136.2, 135.7, 131.2, 128.8, 128.3, 127.3, 126.4, 126.0, 116.9, 48.5, 21.0, 19.5 ppm; IR(neat) ν 2918, 1621, 1572, 1494, 1442, 984, 876, 774, 644, 609, 581, 444 cm^{-1}; HRMS-EI (m/z) M$^+$ calcd. for $C_{25}H_{26}$ 326.2029; found 326.2039.

5,5'-(2-phenylprop-2-ene-1,1-diyl)bis(1,3-dimethylbenzene) (3fa)

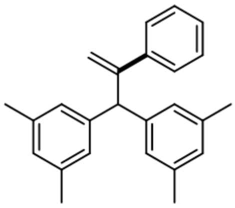

Colorless oil, 36.8 mg, 56 % yield.

^{1}H NMR (400 MHz, $CDCl_3$) δ 7.40 (d, J = 7.6 Hz, 2H), 7.27 – 7.16 (m, 3H), 6.84 – 6.79 (m, 6H), 5.66 (s, 1H), 5.23 (s, 1H), 4.79 (s, 1H), 2.25 (s, 12H) ppm; ^{13}C NMR (100 MHz, $CDCl_3$) δ 150.6, 142.6, 142.1, 137.5, 128.2, 128.0, 127.2, 127.2, 126.4, 117.2, 55.9, 21.4 ppm; IR(neat) ν 3014, 2861, 1599, 1492, 1463, 1444, 1031, 905, 848, 696, 530 cm^{-1}; HRMS-EI (m/z) M$^+$ calcd. for $C_{25}H_{26}$ 326.2029; found 326.2032.

(1-(*o*-tolyl)prop-2-ene-1,2-diyl)dibenzene (3ga)

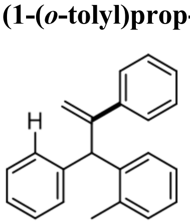

Colorless oil, 37.6 mg, 67 % yield.

^{1}H NMR (400 MHz, $CDCl_3$) δ 7.45 – 7.40 (m, 2H), 7.32 – 7.20 (m, 8H), 7.18 – 7.06 (m, 3H), 7.05 – 7.01 (m, 1H), 5.70 (s, 1H), 5.49 (s, 1H), 4.71 (s, 1H), 2.30 (s, 3H) ppm; ^{13}C NMR (100 MHz,

$CDCl_3$) δ 150.0, 141.9, 141.8, 141.1, 136.4, 130.4, 129.7, 129.0, 128.3, 128.3, 127.4, 126.4, 126.4, 126.2, 125.8, 117.2, 52.6, 19.8 ppm; IR(neat) ν 3023, 2920, 1599, 1490, 1448, 1029, 907, 776, 742, 697, 596, 447 cm^{-1}; HRMS-EI (m/z) M^+ calcd. for $C_{22}H_{20}$ 284.1560; found 284.1557.

2-(2-phenyl-1-(*o*-tolyl)allyl)-1,1'-biphenyl (3ha)

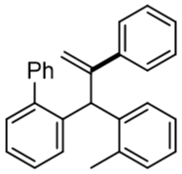

White solid, 47.6 mg, 66 % yield, m. p. 122.7-124.6 °C.
^1H NMR (400 MHz, $CDCl_3$) δ 7.30 – 7.02 (m, 19H), 5.61 (s, 1H), 5.33 (s, 1H), 4.77 (s, 1H), 1.86 (s, 3H) ppm; ^{13}C NMR (100 MHz, $CDCl_3$) δ 150.7, 142.5, 141.8, 141.6, 141.1, 139.1, 136.5, 130.2, 130.0, 129.8, 129.3, 129.0, 128.1, 127.9, 127.3, 127.2, 127.0, 126.0, 126.2, 125.6, 117.4, 49.4, 19.4 ppm; IR(neat) ν 3019, 2921, 1487, 1473, 1445, 1435, 907, 774, 756, 742, 703, 691, 599, 511, 447 cm^{-1}; HRMS-EI (m/z) M^+ calcd. for $C_{28}H_{24}$ 360.1873; found 360.1867.

1-methyl-2-(2-phenyl-1-(2-(trifluoromethyl)phenyl)allyl)benzene (3ia)

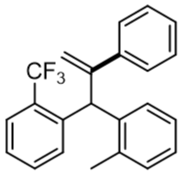

White solid, 61.1 mg, 87 % yield, m. p. 88.6-91.2 °C.
^1H NMR (400 MHz, $CDCl_3$) δ 7.69 (d, J = 7.8 Hz, 1H), 7.47 – 7.41 (m, 3H), 7.37 – 7.29 (m, 2H), 7.27 – 7.10 (m, 6H), 7.06 (d, J = 7.3 Hz, 1H), 5.95 (s, 1H), 5.67 (s, 1H), 4.63 (s, 1H), 2.31 (s, 3H) ppm; ^{13}C NMR (100 MHz, $CDCl_3$) δ 150.0, 141.5, 140.2, 140.1, 136.6, 131.6, 130.7, 128.9, 128.7 (q, J = 29.3 Hz), 128.4, 127.6, 126.7, 126.6 (q, J = 5.9 Hz), 126.4, 125.8, 124.5 (q, J = 273.8 Hz), 117.6, 48.3, 19.7 ppm; ^{19}F NMR (376 MHz, $CDCl_3$) δ -59.9. (s, 3F) ppm; IR(neat) ν 3024, 2924, 1602, 1488, 1448, 1308, 1148, 1104, 1059, 1036, 912, 771, 746, 714, 583, 446 cm^{-1}; HRMS-EI (m/z) M^+ calcd. for $C_{23}H_{19}F$ 352.1433; found 352.1430.

2,2'-(2-phenylprop-2-ene-1,1-diyl)bis(methoxybenzene) (3ja)

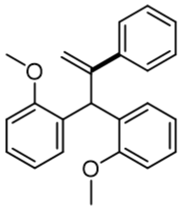

Colorless crystal, 37.6 mg, 57 % yield, m. p. 124.8-126.9 °C.
^1H NMR (400 MHz, $CDCl_3$) δ 7.50 (d, J = 8.2 Hz, 2H), 7.25 (t, J = 7.4 Hz, 2H), 7.22 – 7.16 (m, 2H), 7.05 (d, J = 7.6 Hz, 2H), 6.91 – 6.80 (m, 4H), 6.12 (s, 1H), 5.51 (s, 1H), 4.67 (s, 1H), 3.76 (s, 6H) ppm;

^{13}C NMR (100 MHz, $CDCl_3$) δ 157.2, 150.6, 142.4, 131.0, 129.7, 128.0, 127.3, 127.1, 126.4, 120.0, 115.3, 110.6, 55.7, 41.9 ppm; IR(neat) ν 2923, 2852, 1597, 1584, 1487, 1460, 1438, 1291, 1241, 1185, 1160, 1105, 1051, 1024, 903, 753, 730, 715, 534 cm^{-1}; HRMS-FI (m/z) M$^+$ calcd. for $C_{23}H_{22}O_2$ 330.1614; found 330.1621.

2,2'-(2-phenylprop-2-ene-1,1-diyl)bis(ethylbenzene) (3ka)

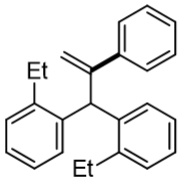

Colorless oil, 43.7 mg, 67 % yield.
^{1}H NMR (400 MHz, $CDCl_3$) δ 7.43 (d, J = 8.0 Hz, 2H), 7.29 – 7.18 (m, 7H), 7.13 – 7.07 (m, 4H), 5.69 (m, 2H), 4.70 (s, 1H), 2.73 – 2.56 (m, J = 7.4 Hz, 4H), 1.21 (t, J = 7.6 Hz, 6H) ppm; ^{13}C NMR (100 MHz, $CDCl_3$) δ 150.3, 142.1, 142.0, 140.1, 129.3, 128.4, 128.3, 127.4, 126.6, 126.2, 125.6, 117.6, 48.1, 25.4, 14.6 ppm; IR(neat) ν 3022, 2964, 2931, 2873, 1624, 1599, 1573, 1484, 1447, 1056, 1029, 883, 776, 753, 702, 600 cm^{-1}; HRMS-EI (m/z) M$^+$ calcd. for $C_{25}H_{26}$ 326.2029; found 326.2029.

5,5'-(2-phenylprop-2-ene-1,1-diyl)bis(1,2,4-trimethylbenzene) (3la)

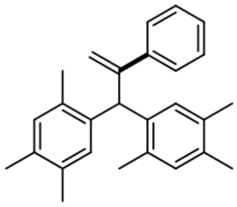

White solid, 44.6 mg, 63 % yield, m. p. 136.3-138.7 °C.
^{1}H NMR (400 MHz, $CDCl_3$) δ 7.46 (d, J = 6.8 Hz, 2H), 7.37 – 7.23 (m, 3H), 6.99 (s, 2H), 6.85 (s, 2H), 5.71 (s, 1H), 5.43 (s, 1H), 4.76 (s, 1H), 2.26 (s, 6H), 2.23 (s, 6H), 2.20 (s, 6H) ppm; ^{13}C NMR (100 MHz, $CDCl_3$) δ 149.9, 142.4, 137.9, 134.0, 133.5, 133.4, 131.7, 131.7, 130.2, 128.3, 127.2, 126.1, 116.9, 48.6, 19.5, 19.2, 19.0 ppm; IR(neat) ν 2916, 1618, 1497, 1450, 1021, 909, 870, 778, 689, 631, 451 cm^{-1}; HRMS-EI (m/z) M$^+$ calcd. for $C_{27}H_{30}$ 354.2342; found 354.2346.

5-(2-phenyl-1-(*o*-tolyl)allyl)-1,2,3,4-tetrahydronaphthalene (3ma)

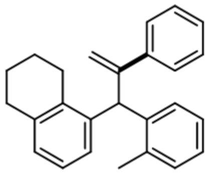

Colorless oil, 52.5 mg, 77 % yield.
^{1}H NMR (400 MHz, $CDCl_3$) δ 7.52 – 7.42 (m, 2H), 7.32 – 7.04 (m, 9H), 6.97 (d, J = 7.3 Hz, 1H), 5.73 (s, 1H), 5.56 (s, 1H), 4.75 (s, 1H), 2.88 – 2.77 (m, 3H), 2.63 – 2.54 (m, 1H), 2.33 (s, 3H), 1.87 – 1.77 (m, 4H) ppm; ^{13}C NMR (100 MHz, $CDCl_3$) δ 149.5, 142.0, 140.8, 140.3, 137.4, 136.5, 135.2, 130.2, 128.9, 128.3, 127.7, 127.4, 126.6, 126.3, 126.0, 125.8, 124.9, 117.0, 48.5, 30.3, 25.8, 23.4,

22.7, 19.6 ppm; IR(neat) ν 3020, 2857, 1624, 1599, 1578, 1490, 1452, 906, 777, 762, 734, 699, 450 cm^{-1}; HRMS-EI (m/z) M^+ calcd. for $C_{26}H_{26}$ 338.2029; found 338.2035.

1-(2-phenyl-1-(*o*-tolyl)allyl)naphthalene (3na)

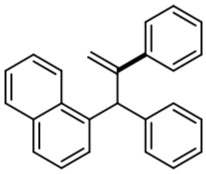

Colorless oil, 30.1 mg, 45 % yield.

^1H NMR (400 MHz, $CDCl_3$) δ 7.98 – 7.89 (m, 2H), 7.80 (d, J = 8.2 Hz, 1H), 7.56 – 7.47 (m, 4H), 7.41 (t, J = 7.7 Hz, 1H), 7.33 – 7.26 (m, 5H), 7.24 – 7.19 (m, 1H), 7.15 – 7.10 (m, 2H), 6.18 (s, 1H), 5.82 (s, 1H), 4.78 (s, 1H), 2.36 (s, 3H) ppm; ^{13}C NMR (100 MHz, $CDCl_3$) δ 149.1, 141.7, 140.8, 138.2, 136.3, 134.0, 132.0, 130.5, 129.1, 128.8, 128.4, 127.5, 127.4, 127.0, 126.6, 126.1, 126.0, 125.9, 125.4, 125.3, 123.9, 117.8, 48.7, 19.7 ppm; IR(neat) ν 3018, 2920, 1624, 1598, 1573, 1508, 885, 779, 742, 702, 597, 523, 448 cm^{-1}; HRMS-EI (m/z) M^+ calcd. for $C_{26}H_{22}$ 334.1716; found 334.1716.

2-(2-phenyl-1-(*o*-tolyl)allyl)naphthalene (3oa)

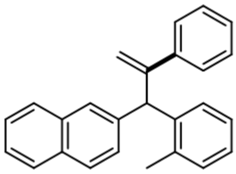

White solid, 36.1 mg, 54 % yield, m. p. 128.1-130.3 °C.

^1H NMR (400 MHz, $CDCl_3$) δ 7.90 – 7.82 (m, 2H), 7.79 (dd, J = 6.2, 3.4 Hz, 1H), 7.67 (s, 1H), 7.55 – 7.47 (m, 4H), 7.44 (dd, J = 8.5, 1.8 Hz, 1H), 7.32 – 7.23 (m, 4H), 7.19 (t, J = 8.9 Hz, 1H), 7.16 (t, J = 7.4 Hz, 1H), 7.10 (d, J = 7.5 Hz, 1H), 5.81 (s, 1H), 5.70 (s, 1H), 4.82 (s, 1H), 2.38 (s, 3H) ppm; ^{13}C NMR (100 MHz, $CDCl_3$) δ 149.7, 141.8, 141.0, 139.6, 136.5, 133.5, 132.3, 130.4, 129.2, 128.4, 128.3, 128.0, 127.9, 127.9, 127.5, 127.4, 126.5, 126.2, 125.9, 125.8, 125.5, 117.5, 52.7, 19.8 ppm; IR(neat) ν 3055, 2920, 1625, 1598, 1489, 907, 863, 822, 802, 782, 749, 705, 528, 476, 448 cm^{-1}; HRMS-EI (m/z) M^+ calcd. for $C_{26}H_{22}$ 334.1716; found 334.1721.

(4-methylpent-1-ene-2,3-diyl)dibenzene (3pa)

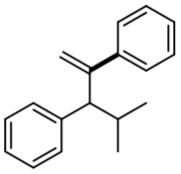

Colorless oil, 35.2 mg, 74 % yield.

^1H NMR (400 MHz, $CDCl_3$) δ 7.30 – 7.12 (m, 10H), 5.35 (d, J = 7.7 Hz, 2H), 3.33 (d, J = 10.3 Hz, 1H), 2.27 (dhept, J = 10.5 Hz, 6.4 Hz, 1H), 1.13 (d, J = 6.5 Hz, 3H), 0.76 (d, J = 6.6 Hz, 3H) ppm; ^{13}C NMR (100 MHz, $CDCl_3$) δ 151.7, 143.8, 143.1, 128.6, 128.1, 128.0, 127.0, 126.8, 126.0, 113.0, 59.5, 31.8, 22.1, 21.5 ppm; IR(neat) ν 3025, 2955, 1624, 1599, 1574, 1492, 1452, 1384, 1072, 1029,

898, 774, 748, 697, 599, 574, 512 cm^{-1}; HRMS-EI (m/z) M^+ calcd. for $C_{18}H_{20}$ 236.1560; found 236.1561.

(1-cyclohexylprop-2-ene-1,2-diyl)dibenzene (3qa)

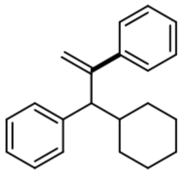

Colorless oil, 40.5 mg, 72 % yield.

^{1}H NMR (400 MHz, $CDCl_3$) δ 7.32 – 7.12 (m, 10H), 5.37 (s, 1H), 5.34 (s, 1H), 3.42 (d, J = 10.4 Hz, 1H), 2.24 (d, J = 13.0 Hz, 1H), 1.92 (q, J = 10.9 Hz, 1H), 1.81 – 1.65 (m, 3H), 1.40 – 1.16 (m, 4H), 1.00 (q, J = 12.5 Hz, 1H), 0.83 (q, J = 10.7, 10.2 Hz, 1H) ppm; ^{13}C NMR (100 MHz, $CDCl_3$) δ 151.2, 143.9, 142.8, 128.6, 128.0, 127.9, 126.9, 126.8, 126.0, 112.8, 58.2, 41.4, 32.4, 31.8, 26.6, 26.5, 26.4 ppm; IR(neat) ν 3024, 2919, 2849, 1624, 1599, 1492, 1447, 1073, 1029, 899, 774, 746, 697, 585, 500 cm^{-1}; HRMS-EI (m/z) M^+ calcd. for $C_{21}H_{24}$ 276.1873; found 276.1876.

2,2'-(2-(p-tolyl)prop-2-ene-1,1-diyl)bis(methylbenzene) (3bb)

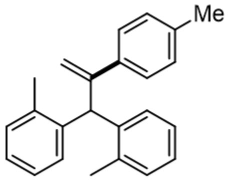

White solid, 38.1 mg, 61 % yield, m. p. 70.2-71.5 °C.

^{1}H NMR (400 MHz, $CDCl_3$) δ 7.34 (d, J = 8.0 Hz, 2H), 7.23 – 7.03 (m, 10H), 5.69 (s, 1H), 5.53 (s, 1H), 4.66 (s, 1H), 2.32 (s, 3H), 2.28 (s, 6H) ppm; ^{13}C NMR (100 MHz, $CDCl_3$) δ 149.0, 140.6, 139.0, 137.1, 136.5, 130.3, 129.0, 128.9, 126.4, 125.9, 125.8, 116.3, 49.2, 21.0, 19.5 ppm; IR(neat) ν 3019, 2920, 2851, 1622, 1566, 1511, 1483, 1459, 1399, 904, 819, 765, 733, 583, 565, 478, 448 cm^{-1}; HRMS-EI (m/z) M^+ calcd. for $C_{24}H_{24}$ 312.1873; found 312.1880.

2,2'-(2-(4-(tert-butyl)phenyl)prop-2-ene-1,1-diyl)bis(methylbenzene) (3bc)

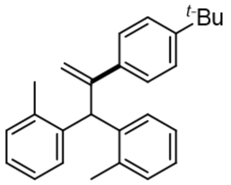

White solid, 40.7 mg, 57 % yield, m. p. 110.7-112.8 °C.

^{1}H NMR (400 MHz, $CDCl_3$) δ 7.41 – 7.36 (m, 2H), 7.33 – 7.29 (m, 2H), 7.21 (d, J = 7.4, 2H), 7.20 – 7.16 (m, 2H), 7.15 – 7.11 (m, 2H), 7.07 (d, J = 7.4 Hz, 2H), 5.73 (s, 1H), 5.54 (s, 1H), 4.68 (s, 1H), 2.29 (s, 6H), 1.32 (s, 9H) ppm; ^{13}C NMR (100 MHz, $CDCl_3$) δ 150.3, 148.8, 140.7, 138.8, 136.5, 130.3, 128.9, 126.3, 125.8, 125.6, 125.2, 116.5, 49.1, 34.4, 31.3, 19.6 ppm; IR(neat) ν 2965, 2865, 1624, 1601, 1510, 1480, 1459, 1119, 1029, 905, 844, 746, 726, 568, 548, 451 cm^{-1}; HRMS-EI (m/z) M^+ calcd. for $C_{27}H_{30}$ 354.2342; found 354.2351.

(4-(3,3-di-*o*-tolylprop-1-en-2-yl)phenyl)trimethylsilane (3bd)

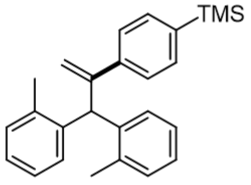

White solid, 41.2 mg, 62 % yield, m. p. 132.3-133.5 °C.

^{1}H NMR (400 MHz, $CDCl_3$) δ 7.48 – 7.41 (m, 4H), 7.22 – 7.11 (m, 6H), 7.06 (d, *J* = 6.9 Hz, 2H), 5.75 (s, 1H), 5.55 (s, 1H), 4.71 (s, 1H), 2.28 (s, 6H), 0.26 (s, 9H) ppm; ^{13}C NMR (100 MHz, $CDCl_3$) δ 149.1, 142.1, 140.5, 139.5, 136.5, 133.4, 130.3, 128.9, 126.4, 125.8, 125.2, 117.3, 49.1, 19.6, -1.2 ppm; ^{29}Si NMR (80 MHz, $CDCl_3$) δ -4.3 ppm; IR(neat) ν 3013, 2948, 1595, 1485, 1460, 1245, 1114, 1075, 920, 837, 825, 747, 729, 628, 533, 453 cm^{-1}; HRMS-EI (m/z) M^+ calcd. for $C_{26}H_{30}Si$ 370.2111; found 370.2110.

4-(3,3-di-*o*-tolylprop-1-en-2-yl)-N,N-diphenylaniline (3be)

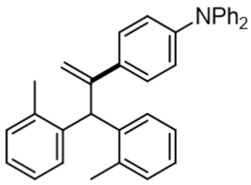

Colorless oil, 61.1 mg, 66 % yield.

^{1}H NMR (400 MHz, $CDCl_3$) δ 7.24 (m, 3H), 7.23 – 7.20 (m, 3H), 7.17 – 7.12 (m, 4H), 7.11 (s, 1H), 7.09 (m, 1H), 7.08 – 7.05 (m, 4H), 7.03 – 6.96 (m, 4H), 6.91 (d, *J* = 8.7 Hz, 2H), 5.65 (s, 1H), 5.45 (s, 1H), 4.59 (s, 1H), 2.23 (s, 6H) ppm; ^{13}C NMR (100 MHz, $CDCl_3$) δ 148.3, 147.5, 147.0, 140.7, 136.5, 135.4, 130.3, 129.2, 128.9, 126.6, 126.3, 125.8, 124.6, 122.9, 122.7, 115.6, 49.0, 19.6 ppm; IR(neat) ν 3032, 2920, 1588, 1506, 1487, 1460, 1314, 1275, 1178, 1156, 906, 842, 827, 745, 728, 695, 645, 618, 535, 507, 448, 408 cm^{-1}; HRMS-ESI (m/z) $[M+H]^+$ calcd. for $C_{35}H_{32}N$ 466.2529; found 466.2535.

2,2'-(2-(4-fluorophenyl)prop-2-ene-1,1-diyl)bis(methylbenzene) (3bf)

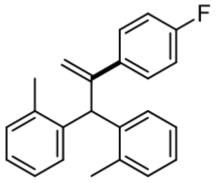

Colorless crystal, 47.5 mg, 75 % yield, m. p. 83.8-86.5 °C.

^{1}H NMR (400 MHz, $CDCl_3$) δ 7.44 – 7.36 (m, 2H), 7.23 – 7.12 (m, 6H), 7.05 (d, *J* = 9.1 Hz, 2H), 7.01 – 6.93 (m, 2H), 5.66 (s, 1H), 5.49 (s, 1H), 4.70 (s, 1H), 2.28 (s, 6H) ppm; ^{13}C NMR (100 MHz, $CDCl_3$) δ 162.2 (d, *J* = 246.5 Hz), 148.3, 140.2, 138.0 (d, *J* = 3.4 Hz), 136.5, 130.4, 128.9, 127.6 (d, *J* = 7.9 Hz), 126.5, 125.9, 117.0 (d, *J* = 1.3 Hz), 115.2 (d, *J* = 21.3 Hz), 49.3, 19.5 ppm; ^{19}F NMR (377 MHz, $CDCl_3$) δ -115.0 (tt, *J* = 8.6, 5.4 Hz) ppm; IR(neat) ν 3061, 2919, 1621, 1600, 1508, 1483, 1459, 1231, 1165, 904, 843, 833, 768, 744, 736, 582, 561, 511, 484, 451 cm^{-1}; HRMS-EI (m/z) M^+ calcd. for $C_{23}H_{21}F$ 316.1622; found 316.1626.

2,2'-(2-(4-chlorophenyl)prop-2-ene-1,1-diyl)bis(methylbenzene) (3bg)

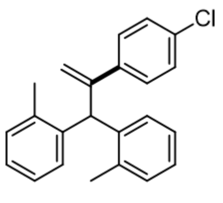

White solid, 56.7 mg, 85 % yield, m. p. 135.9-138.2 °C.

^{1}H NMR (400 MHz, $CDCl_3$) δ 7.34 – 7.29 (m, 2H), 7.22 – 7.18 (m, 2H), 7.16 (m, 2H), 7.13 (td, J = 7.3, 2.0 Hz, 2H), 7.08 (td, J = 7.3, 2.0 Hz, 2H), 6.99 (d, J = 7.4 Hz, 2H), 5.65 (d, J = 0.9 Hz, 1H), 5.43 (s, 1H), 4.67 (d, J = 1.2 Hz, 1H), 2.23 (s, 6H) ppm; ^{13}C NMR (100 MHz, $CDCl_3$) δ 3015, 2920, 1487, 1459, 1392, 1378, 1097, 1007, 869, 826, 808, 748, 727, 448 ppm; IR(neat) ν 3013, 2919, 1599, 1486, 1459, 1031, 906, 779, 744, 698, 600, 448 cm^{-1}; HRMS-EI (m/z) M^{+} calcd. for $C_{23}H_{21}Cl$ 332.1326; found 332.1331.

2,2'-(2-(4-bromophenyl)prop-2-ene-1,1-diyl)bis(methylbenzene) (3bh)

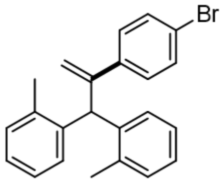

Colorless crystal, 52.3 mg, 75 % yield, m. p. 135.9-138.2 °C.

^{1}H NMR (400 MHz, $CDCl_3$) δ 7.36 (d, J = 8.5 Hz, 2H), 7.25 (d, J = 8.6 Hz, 2H), 7.19 – 7.05 (m, 6H), 6.98 (d, J = 7.4 Hz, 2H), 5.66 (s, 1H), 5.43 (s, 1H), 4.68 (s, 1H), 2.23 (s, 6H) ppm; ^{13}C NMR (100 MHz, $CDCl_3$) δ 148.3, 140.8, 140.0, 136.5, 131.4, 130.4, 128.9, 127.7, 126.6, 125.9, 121.4, 117.6, 49.1, 19.5 ppm; IR(neat) ν 2920, 2852, 1620, 1602, 1485, 1459, 1388, 1378, 1090, 1003, 908, 837, 824, 748, 734, 453, 444 cm^{-1}; HRMS-EI (m/z) M^{+} calcd. for $C_{23}H_{21}Br$ 376.0821; found 376.0828.

1-(4-(3,3-di-*o*-tolylprop-1-en-2-yl)phenyl)ethan-1-one (3bi)

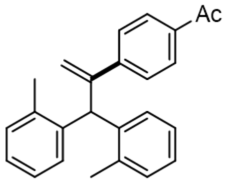

White solid, 53.1 mg, 78 % yield, m. p. 143.9-145.4 °C.

^{1}H NMR (400 MHz, $CDCl_3$) δ 7.87 (d, J = 7.0 Hz, 2H), 7.51 (d, J = 7.0 Hz, 2H), 7.23 – 7.14 (m, 4H), 7.11 (t, J = 7.3 Hz, 2H), 7.02 (d, J = 7.3 Hz, 2H), 5.79 (s, 1H), 5.54 (s, 1H), 4.80 (s, 1H), 2.56 (s, 3H), 2.27 (s, 6H) ppm; ^{13}C NMR (100 MHz, $CDCl_3$) δ 197.4, 148.5, 146.5, 139.9, 136.4, 136.0, 130.4, 128.8, 128.5, 126.6, 126.1, 125.9, 118.9, 49.0, 26.5, 19.4 ppm; IR(neat) ν 2916, 2852, 1680, 1600, 1483, 1461, 1429, 1402, 1357, 1267, 960, 911, 847, 828, 748, 730, 692, 681, 631, 587, 450 cm^{-1}; HRMS-EI (m/z) M^{+} calcd. for $C_{25}H_{24}O$ 340.1822; found 340.1821.

4-(3,3-di-*o*-tolylprop-1-en-2-yl)benzonitrile (3bj)

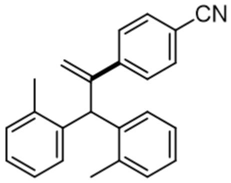

White solid, 58.4 mg, 90 % yield, m. p. 142.5-144.2 °C.
^{1}H NMR (400 MHz, $CDCl_3$) δ 7.59 – 7.46 (m, 4H), 7.24 – 7.15 (m, 4H), 7.12 (t, J = 7.3 Hz, 2H), 7.00 (d, J = 7.6 Hz, 2H), 5.78 (s, 1H), 5.49 (s, 1H), 4.85 (s, 1H), 2.26 (s, 6H) ppm; ^{13}C NMR (100 MHz, $CDCl_3$) δ 148.0, 146.3, 139.5, 136.4, 132.2, 130.5, 128.8, 126.7, 126.6, 126.0, 119.7, 118.7, 110.9, 48.9, 19.4 ppm; IR(neat) ν 3059, 2225, 1624, 1602, 1482, 1461, 1401, 1381, 920, 845, 756, 731, 625, 580, 559, 542, 454 cm^{-1}; HRMS-FI (m/z) M^{+} calcd. for $C_{24}H_{21}N$ 323.1669; found 323.1673.

propyl 4-(3,3-di-*o*-tolylprop-1-en-2-yl)benzoate (3bk)

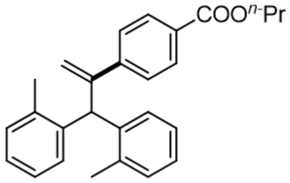

Colorless crystal, 61.4 mg, 80 % yield, m. p. 70.2-71.5 °C.
^{1}H NMR (400 MHz, $CDCl_3$) δ 7.96 (d, J = 8.5 Hz, 2H), 7.49 (d, J = 8.5 Hz, 2H), 7.20 (d, J = 7.4 Hz, 2H), 7.16 (td, J = 7.2, 1.6 Hz, 2H), 7.11 (td, J = 7.4, 1.9 Hz, 2H), 7.03 (d, J = 7.4 Hz, 2H), 5.78 (s, 1H), 5.53 (s, 1H), 4.79 (s, 1H), 4.27 (t, J = 6.6 Hz, 2H), 2.27 (s, 6H), 1.78 (h, J = 7.1 Hz, 2H), 1.03 (t, J = 7.4 Hz, 3H) ppm; ^{13}C NMR (100 MHz, $CDCl_3$) δ 166.3, 148.6, 146.3, 140.0, 136.4, 130.4, 129.7, 129.3, 128.9, 126.6, 125.94, 125.86, 118.7, 66.4, 49.0, 22.1, 19.5, 10.5 ppm; IR(neat) ν 2965, 1713, 1605, 1481, 1459, 1406, 1389, 1376, 1270, 1183, 1159, 1108, 989, 938, 916, 866, 781, 752, 719, 551, 449 cm^{-1}; HRMS-ESI (m/z) [M+Na]$^{+}$ calcd. for $C_{27}H_{28}O_2Na$ 407.1982; found 407.1978.

4-(3,3-di-*o*-tolylprop-1-en-2-yl)-1,1'-biphenyl (3bl)

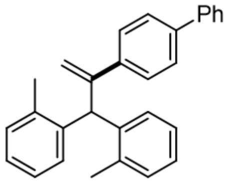

Colorless crystal, 61.2 mg, 82 % yield, m. p. 167.3-169.5 °C.
^{1}H NMR (400 MHz, $CDCl_3$) δ 7.63 (d, J = 7.0 Hz, 2H), 7.58 (m, 4H), 7.49 (t, J = 7.6 Hz, 2H), 7.40 (t, J = 7.3 Hz, 1H), 7.28 (d, J = 7.3 Hz, 2H), 7.25 (td, J = 7.1, 1.9 Hz, 2H), 7.19 (td, J = 7.1, 1.9 Hz, 2H), 7.15 (dd, J = 7.4, 1.8 Hz, 2H), 5.85 (s, 1H), 5.65 (s, 1H), 4.81 (s, 1H), 2.37 (s, 6H) ppm; ^{13}C NMR (100 MHz, $CDCl_3$) δ 148.7, 140.7, 140.6, 140.4, 140.2, 136.5, 130.4, 129.0, 128.7, 127.2, 127.0, 126.9, 126.5, 126.4, 125.9, 117.1, 49.1, 19.6 ppm; IR(neat) ν 3023, 1600, 1577, 1483, 1459, 1401, 1377, 909, 850, 829, 772, 752, 737, 694, 637, 448 cm^{-1}; HRMS-EI (m/z) M^{+} calcd. for $C_{29}H_{26}$ 374.2029; found 374.2026.

2,2'-(2-(4-(difluoromethyl)phenyl)prop-2-ene-1,1-diyl)bis(methylbenzene) (3bm)

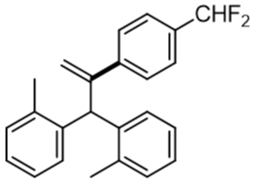

White solid, 41.1 mg, 59 % yield, m. p. 85.0-87.1 °C.

^{1}H NMR (400 MHz, $CDCl_3$) δ 7.48 – 7.42 (m, 2H), 7.25 – 7.22 (m, 2H), 7.19 (dd, J = 7.3, 1.7Hz, 2H), 7.16 (td, J = 7.3, 1.7 Hz, 2H), 7.10 – 7.03 (m, 4H), 6.50 (t, J_{FH} = 74.0 Hz, 1H), 5.74 – 5.68 (m, 1H), 5.51 (s, 1H), 4.74 (s, 1H), 2.30 (s, 6H) ppm; ^{13}C NMR (100 MHz, $CDCl_3$) δ 150.5, 148.1, 140.1, 139.2, 136.5, 130.4, 128.8, 127.4, 126.5, 125.9, 119.2, 117.4, 115.8 (d, J = 259.5 Hz), 49.2, 19.5 ppm; ^{19}F NMR (376 MHz, $CDCl_3$) δ -80.7 (d, J = 74.1 Hz) ppm; IR(neat) ν 2973, 1630, 1601, 1505, 1484, 1461, 1387, 1219, 1178, 1118, 1089, 904, 745, 726, 454, 443 cm^{-1}; HRMS-EI (m/z) M^{+} calcd. for $C_{24}H_{22}F_2$ 364.1633; found 364.1629.

2,2'-(2-(4-(trifluoromethyl)phenyl)prop-2-ene-1,1-diyl)bis(methylbenzene) (3bn)

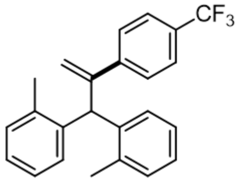

White solid, 54.9 mg, 75 % yield, m. p. 127.2-129.3 °C.

^{1}H NMR (400 MHz, $CDCl_3$) δ 7.58 – 7.49 (m, 4H), 7.25 – 7.10 (m, 6H), 7.04 (d, J = 7.4 Hz, 2H), 5.77 (s, 1H), 5.52 (s, 1H), 4.81 (s, 1H), 2.28 (s, 6H) ppm; ^{13}C NMR (100 MHz, $CDCl_3$) δ 148.3, 145.4, 139.9, 136.5, 130.5, 129.4 (q, J = 32.4 Hz), 128.9, 126.7, 126.3, 126.0, 125.4 (q, J = 3.8 Hz), 124.1 (q, J = 272 Hz), 119.0, 49.1, 19.5 ppm; ^{19}F NMR (376 MHz, $CDCl_3$) δ -62.5(s, 3F) ppm; IR(neat) ν 2975, 1614, 1483, 1461, 1404, 1382, 1319, 1156, 1114, 1089, 1064, 1012, 916, 847, 830, 758, 739, 423 cm^{-1}; HRMS-EI (m/z) M^{+} calcd. for $C_{24}H_{21}F_3$ 366.1590; found 366.1589.

2,2'-(2-(4-(trifluoromethoxy)phenyl)prop-2-ene-1,1-diyl)bis(methylbenzene) (3bo)

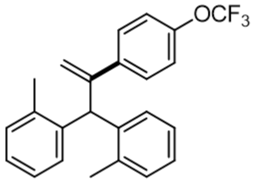

White solid, 65.2 mg, 85 % yield, m. p. 81.3-83.2 °C.

^{1}H NMR (400 MHz, $CDCl_3$) δ 7.47 – 7.41 (m, 2H), 7.24 – 7.11 (m, 8H), 7.05 (d, J = 7.5 Hz, 2H), 5.71 (s, 1H), 5.49 (s, 1H), 4.75 (s, 1H), 2.28 (s, 6H) ppm; ^{13}C NMR (100 MHz, $CDCl_3$) δ 148.5 (q, J = 1.7 Hz), 148.0, 140.6, 140.1, 136.5, 130.5, 128.8, 127.4, 126.6, 125.9, 120.7, 120.4(q, J = 258 Hz), 117.9, 49.2, 19.5 ppm; ^{19}F NMR (377 MHz, $CDCl_3$) δ -57.7 (s, 3F) ppm; IR(neat) ν 2925, 2857, 1629, 1601, 1506, 1483, 1461, 1253, 1219, 1152, 914, 741, 688, 450 cm^{-1}; HRMS-EI (m/z) M^{+} calcd. for $C_{24}H_{21}F_3O$ 382.1539; found 382.1541.

2,2'-(2-(4-(2,2,2-trifluoroethoxy)phenyl)prop-2-ene-1,1-diyl)bis(methylbenzene) (3bp)

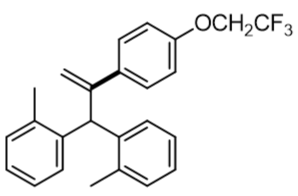

White solid, 44.6 mg, 56 % yield, m. p. 76.7-78.2 °C.
^{1}H NMR (400 MHz, $CDCl_3$) δ 7.42 – 7.35 (m, 2H), 7.20 (d, J = 7.4 Hz, 2H), 7.16 (td, J = 7.2, 1.6 Hz, 2H), 7.11 (td, J = 7.2, 1.9 Hz, 2H), 7.03 (d, J = 7.5 Hz, 2H), 6.88 – 6.80 (m, 2H), 5.65 (s, 1H), 5.48 (s, 1H), 4.65 (s, 1H), 4.30 (q, J_{FH} = 8.2 Hz, 2H), 2.27 (s, 6H) ppm; ^{13}C NMR (100 MHz, $CDCl_3$) δ 156.7, 148.2, 140.3, 136.5, 136.3, 130.4, 128.9, 127.4, 126.5, 125.8, 123.3(q, J = 279Hz) 116.4, 114.7, 65.7 (q, J = 35.7 Hz), 49.2, 19.5 ppm; ^{19}F NMR (376 MHz, $CDCl_3$) δ -74.0 (t, J = 8.0 Hz, 3F) ppm; IR(neat) ν 2973, 2946, 1604, 1510, 1483, 1458, 1291, 1235, 1190, 1162, 1096, 1074, 976, 900, 839, 827, 776, 748, 671, 453 cm^{-1}; HRMS-EI (m/z) M$^+$ calcd. for $C_{25}H_{23}F_3O$ 396.1696; found 396.1701.

2,2'-(2-(4-phenoxyphenyl)prop-2-ene-1,1-diyl)bis(methylbenzene) (3bq)

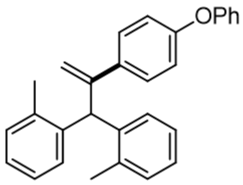

White solid, 20.2 mg, 26 % yield, m. p. 104.3-106.5 °C.
^{1}H NMR (400 MHz, $CDCl_3$) δ 7.40 – 7.36 (m, 2H), 7.35 – 7.31 (m, 2H), 7.20 – 7.14 (m, 4H), 7.13 – 7.08 (m, 3H), 7.05 – 6.99 (m, 4H), 6.91 – 6.87 (m, 2H), 5.66 (s, 1H), 5.48 (s, 1H), 4.64 (s, 1H), 2.26 (s, 6H) ppm; ^{13}C NMR (100 MHz, $CDCl_3$) δ 156.8, 156.8, 148.3, 140.4, 136.7, 136.5, 130.3, 129.7, 128.9, 127.3, 126.4, 125.8, 123.4, 119.2, 118.2, 116.4, 49.2, 19.5 ppm; IR(neat) ν 3062, 2968, 1587, 1460, 1239, 1166, 912, 872, 857, 748, 688, 464 cm^{-1}; HRMS-EI (m/z) M$^+$ calcd. for $C_{29}H_{26}O$ 390.1978; found 390.1974.

2,2'-(2-(*m*-tolyl)prop-2-ene-1,1-diyl)bis(methylbenzene) (3br)

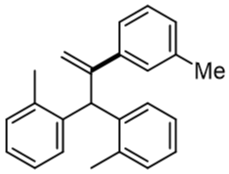

White solid, 52.5 mg, 84 % yield, m. p. 72.2-74.3 °C.
^{1}H NMR (400 MHz, $CDCl_3$) δ 7.30 (s, 1H), 7.24 – 7.19 (m, 4H), 7.18 (s, 2H), 7.14 (m, 2H), 7.07 (d, J = 7.3 Hz, 3H), 5.71 (s, 1H), 5.56 (s, 1H), 4.70 (s, 1H), 2.35 (s, 3H), 2.30 (s, 6H) ppm; ^{13}C NMR (100 MHz, $CDCl_3$) δ 149., 142.0, 140.5, 137.7, 136.5, 130.3, 128.9, 128.2, 128.2, 126.8, 126.4, 125.8, 123.1, 117.0, 49.2, 21.6, 19.5 ppm; IR(neat) ν 3014, 2918, 1600, 1483, 1459, 910, 794, 761, 733, 674, 604, 580, 449 cm^{-1}; HRMS-EI (m/z) M$^+$ calcd. for $C_{24}H_{24}$ 312.1873; found 312.1873.

2,2'-(2-(3-methoxyphenyl)prop-2-ene-1,1-diyl)bis(methylbenzene) (3bs)

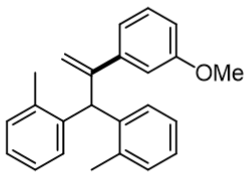

Colorless oil, 40.8 mg, 62 % yield.

^{1}H NMR (400 MHz, $CDCl_3$) δ 7.24 – 7.10 (m, 7H), 7.05 (m, 3H), 6.99 (s, 1H), 6.79 (dd, *J* = 8.1, 2.6 Hz, 1H), 5.72 (s, 1H), 5.52 (s, 1H), 4.71 (s, 1H), 3.7 (s, 3H), 2.29 (s, 6H) ppm; ^{13}C NMR (100 MHz, $CDCl_3$) δ 159.4, 149.1, 143.5, 140.4, 136.5, 130.3, 129.2, 128.9, 126.4, 125.8, 118.6, 117.3, 112.5, 112.2, 55.0, 49.2, 19.5 ppm; IR(neat) ν 3017, 2921, 1599, 1574, 1485, 1460, 1427, 1285, 1256, 1215, 1171, 1048, 908, 880, 781, 763, 732, 448 cm^{-1}; HRMS-EI (m/z) M$^+$ calcd. for $C_{24}H_{24}O$ 328.1822; found 328.1816.

(3-(3,3-di-*o*-tolylprop-1-en-2-yl)phenyl)trimethylsilane (3bt)

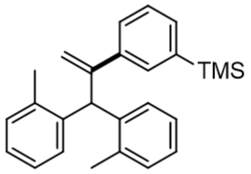

White solid, 43.6 mg, 58 % yield, m. p. 68.5-71.5 °C.

^{1}H NMR (400 MHz, $CDCl_3$) δ 7.55 (s, 1H), 7.43 (t, *J* = 8.4 Hz, 2H), 7.29 (t, *J* = 7.6 Hz, 1H), 7.23 – 7.09 (m, 8H), 5.74 (s, 1H), 5.54 (s, 1H), 4.73 (s, 1H), 2.30 (s, 6H), 0.23 (s, 9H) ppm; ^{13}C NMR (100 MHz, $CDCl_3$) δ 149.4, 141.0, 140.5, 140.3, 136.4, 132.4, 131.0, 130.3, 129.0, 127.7, 126.5, 126.4, 125.8, 116.8, 49.3, 19.5, -1.3 ppm; ^{29}Si NMR (80 MHz, $CDCl_3$) δ -3.9 ppm; IR(neat) ν 3014, 2949, 1482, 1459, 1378, 1247, 1131, 917, 862, 838, 798, 747, 725, 685, 619, 607, 451 cm^{-1}; HRMS-EI (m/z) M$^+$ calcd. for $C_{26}H_{30}Si$ 298.1716; found 298.1714.

2,2'-(2-(3-fluorophenyl)prop-2-ene-1,1-diyl)bis(methylbenzene) (3bu)

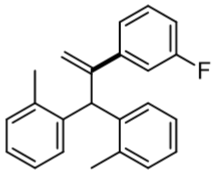

White solid, 52.1 mg, 82 % yield, m. p. 73.9-76.6 °C.

^{1}H NMR (400 MHz, $CDCl_3$) δ 7.26 – 7.07 (m, 9H), 7.01 (d, *J* = 6.8 Hz, 2H), 6.94 – 6.88 (m, 1H), 5.70 (s, 1H), 5.46 (s, 1H), 4.72 (s, 1H), 2.25 (s, 6H) ppm; ^{13}C NMR (100 MHz, $CDCl_3$) δ 162.8 (d, *J* = 244.9 Hz), 148.3, 144.3 (d, *J* = 7.3 Hz), 140.0, 136.5, 130.4, 129.8 (d, *J* = 8.3 Hz), 128.8, 126.6, 125.9, 121.7 (d, *J* = 2.7 Hz), 118.1, 114.2 (d, *J* = 21.3 Hz), 113.0 (d, *J* = 21.9 Hz), 49.1, 19.5 ppm; ^{19}F NMR (376 MHz, $CDCl_3$) δ -113.3 (ddd, *J* = 10.8, 8.5, 5.6 Hz, 1F) ppm; IR(neat) ν 2923, 2853, 1605, 1577, 1484, 1460, 1436, 1255, 1188, 1161, 906, 873, 785, 765, 734, 447 cm^{-1}; HRMS-EI (m/z) M$^+$ calcd. for $C_{23}H_{21}F$ 316.1622; found 316.1630.

2,2'-(2-(3-chlorophenyl)prop-2-ene-1,1-diyl)bis(methylbenzene) (3bv)

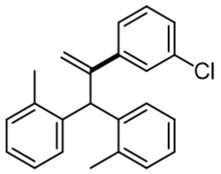

White solid, 44.0 mg, 66 % yield, m. p. 66.7-69.0 °C.

^{1}H NMR (400 MHz, $CDCl_3$) δ 7.48 (m, 1H), 7.30 (dt, J = 6.8, 2.1 Hz, 1H), 7.25 – 7.14 (m, 8H), 7.06 (d, J = 7.4 Hz, 2H), 5.73 (s, 1H), 5.52 (s, 1H), 4.77 (s, 1H), 2.31 (s, 6H) ppm; ^{13}C NMR (100 MHz, $CDCl_3$) δ 148.2, 143.8, 139.9, 136.5, 134.2, 130.4, 129.6, 128.8, 127.5, 126.6, 126.2, 125.9, 124.1, 118.2, 49.0, 19.5 ppm; IR(neat) ν 3016, 2925, 1590, 1557, 1482, 1461, 911, 873, 834, 790, 762, 744, 726, 689, 576, 457 cm^{-1}; HRMS-EI (m/z) M^{+} calcd. for $C_{23}H_{21}Cl$ 332.1326; found 332.1332.

2,2'-(2-(3-bromophenyl)prop-2-ene-1,1-diyl)bis(methylbenzene) (3bw)

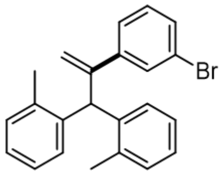

Colorless oil, 37.7 mg, 50 % yield.

^{1}H NMR (400 MHz, $CDCl_3$) δ 7.64 (t, J = 1.9 Hz, 1H), 7.38 (d, J = 7.8 Hz, 1H), 7.32 (d, J = 8.1 Hz, 1H), 7.24 – 7.13 (m, 7H), 7.05 (d, J = 7.5 Hz, 2H), 5.71 (s, 1H), 5.49 (s, 1H), 4.75 (s, 1H), 2.30 (s, 6H) ppm; ^{13}C NMR (100 MHz, $CDCl_3$) δ 148.1, 144.1, 139.9, 136.4, 130.4, 130.4, 129.9, 129.2, 128.8, 126.6, 125.9, 124.6, 122.5, 118.3, 49.0, 19.5 ppm; IR(neat) ν 3060, 2923, 1558, 1485, 1459, 907, 781, 751, 729, 713, 688, 675, 452 cm^{-1}; HRMS-EI (m/z) M^{+} calcd. for $C_{23}H_{21}Br$ 376.0821; found 376.0825.

3-(3,3-di-*o*-tolylprop-1-en-2-yl)benzonitrile (3bx)

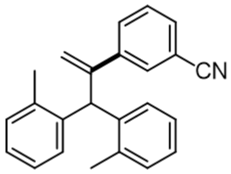

White solid, 56.4 mg, 87 % yield, m. p. 154.2-156.6 °C.

^{1}H NMR (400 MHz, $CDCl_3$) δ 7.70 (s, 1H), 7.61 (d, J = 8.0 Hz, 1H), 7.50 (d, J = 7.7 Hz, 1H), 7.36 (t, J = 7.8 Hz, 1H), 7.23 – 7.14 (m, 4H), 7.14 – 7.08 (m, 2H), 6.99 (d, J = 7.1 Hz, 2H), 5.71 (s, 1H), 5.46 (s, 1H), 4.79 (s, 1H), 2.26 (s, 6H) ppm; ^{13}C NMR (100 MHz, $CDCl_3$) δ 147.6, 143.2, 139.5, 136.4, 130.9, 130.6, 130.4, 129.7, 129.2, 128.8, 126.7, 126.0, 119.1, 118.8, 112.6, 49.0, 19.4 ppm; IR(neat) ν 3018, 2855, 2226, 1486, 1462, 915, 890, 804, 765, 745, 732, 665, 611, 458 cm^{-1}; HRMS-EI (m/z) M^{+} calcd. for $C_{24}H_{21}N$ 323.1669; found 323.1675.

2,2'-(2-(3-(trifluoromethyl)phenyl)prop-2-ene-1,1-diyl)bis(methylbenzene) (3by)

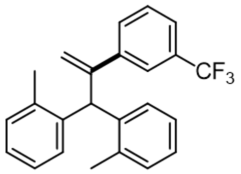

White solid, 61.4 mg, 84 % yield, m. p. 61.5-63.8 °C.
^{1}H NMR (400 MHz, CDCl$_3$) δ 7.77 (s, 1H), 7.59 (d, *J* = 7.8 Hz, 1H), 7.52 (d, *J* = 7.8 Hz, 1H), 7.41 (t, *J* = 7.8 Hz, 1H), 7.27 – 7.16 (m, 6H), 7.09 (d, *J* = 7.4 Hz, 2H), 5.80 (s, 1H), 5.57 (s, 1H), 4.84 (s, 1H), 2.33 (s, 6H) ppm; ^{13}C NMR (100 MHz, CDCl$_3$) δ 148.3, 142.7, 139.9, 136.5, 130.8 (q, *J* = 32.1 Hz), 130.6, 129.3, 129.2, 129.0, 126.7, 126.0, 124.20 (q, *J* = 3.8 Hz), 124.17 (q, *J* = 273 Hz), 122.9 (q, *J* = 3.9 Hz), 118.5, 49.1, 19.6 ppm; ^{19}F NMR (376 MHz, CDCl$_3$) δ -62.6 (s, 3F) ppm; IR(neat) ν 2923, 1485, 1460, 1432, 1335, 1267, 1238, 1149, 1125, 1071, 921, 900, 808, 765, 742, 726, 700, 453 cm^{-1}; HRMS-EI (m/z) M$^+$ calcd. for $C_{24}H_{21}F_3$ 366.1590; found 366.1592.

2,2'-(2-(2-fluorophenyl)prop-2-ene-1,1-diyl)bis(methylbenzene) (3bz)

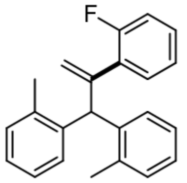

White solid, 31.9 mg, 50 % yield, m. p. 69.2-71.7 °C.
^{1}H NMR (400 MHz, CDCl$_3$) δ 7.36 (t, *J* = 7.8 Hz, 1H), 7.21 – 7.08 (m, 9H), 7.06 – 6.97 (m, 2H), 5.69 (s, 1H), 5.53 (s, 1H), 4.83 (s, 1H), 2.33 (s, 6H) ppm; ^{13}C NMR (100 MHz, CDCl$_3$) δ 159.9 (d, *J* = 247.3 Hz), 145.2 (d, *J* = 2.1 Hz), 139.9, 136.9, 130.4, 130.3, 130.1 (d, *J* = 4.2 Hz), 129.2, 128.7 (d, *J* = 8.6 Hz), 126.4, 125.7, 124.0 (d, *J* = 3.4 Hz), 120.6 (d, *J* = 2.9 Hz), 115.9 (d, *J* = 23.6 Hz), 49.40 (d, *J* = 3.6 Hz), 19.49 ppm; ^{19}F NMR (376 MHz, CDCl$_3$) δ -115.0 (dt, *J* = 11.9, 6.7 Hz, 1F) ppm; IR(neat) ν 3063, 2922, 1484, 1448, 1203, 922, 789, 772, 757, 602, 505, 452 cm^{-1}; HRMS-EI (m/z) M$^+$ calcd. for $C_{23}H_{21}F$ 316.1622; found 316.1618.

2,2'-(2-(2-fluoro-4-methoxyphenyl)prop-2-ene-1,1-diyl)bis(methylbenzene) (3baa)

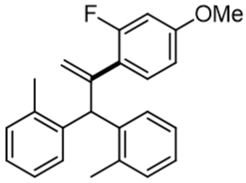

Colorless oil, 39.2 mg, 57 % yield.
^{1}H NMR (400 MHz, CDCl$_3$) δ 7.23 – 7.07 (m, 8H), 7.01 (d, *J* = 7.5 Hz, 2H), 6.84 (t, *J* = 9.2 Hz, 1H), 5.63 (s, 1H), 5.44 (s, 1H), 4.67 – 4.61 (m, 1H), 3.85 (s, 3H), 2.26 (s, 6H) ppm; ^{13}C NMR (100 MHz, CDCl$_3$) δ 152.1 (d, *J* = 244.9 Hz), 147.7, 146.9 (d, *J* = 11.2 Hz), 140.2, 136.5, 135.1 (d, *J* = 5.9 Hz), 130.4, 128.8, 126.5, 125.8, 121.7 (d, *J* = 3.4 Hz), 116.5, 113.8 (d, *J* = 18.9 Hz), 113.1, 56.2, 49.1, 19.5 ppm; ^{19}F NMR (376 MHz, CDCl$_3$) δ -135.4 (dd, *J* = 13.0, 8.9 Hz, 1F) ppm; IR(neat) ν 3017, 2934, 1619, 1575, 1515, 1485, 1460, 1441, 1427, 1269, 1175, 1133, 1026, 908, 879, 788, 732, 614, 449 cm^{-1}; HRMS-EI (m/z) M$^+$ calcd. for $C_{24}H_{23}FO$ 346.1727; found 346.1731.

2,2'-(2-(3,5-difluorophenyl)prop-2-ene-1,1-diyl)bis(methylbenzene) (3bab)

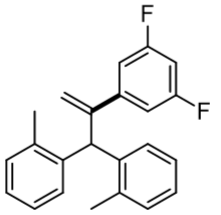

White solid, 54.6 mg, 82 % yield, m. p. 111.6-113.0 °C.
^{1}H NMR (400 MHz, $CDCl_3$) δ 7.26 – 7.10 (m, 6H), 7.01 (d, *J* = 7.5 Hz, 2H), 6.94 (d, *J* = 7.0 Hz, 2H), 6.68 (t, *J* = 8.8 Hz, 1H), 5.73 (s, 1H), 5.44 (s, 1H), 4.78 (s, 1H), 2.28 (s, 6H) ppm; ^{13}C NMR (100 MHz, $CDCl_3$) δ 162.9 (dd, *J* = 247.5, 13.1 Hz), 147.6, 145.4 (t, *J* = 9.1 Hz), 139.6, 136.4, 130.5, 128.8, 126.7, 126.0, 119.0, 109.3 – 108.6 (m), 102.7(t, *J* = 25.5 Hz), 49.0, 19.4 ppm; ^{19}F NMR (376 MHz, $CDCl_3$) δ -110.0 (t, *J* = 8.3 Hz, 2F) ppm; IR(neat) ν 3075, 2920, 1619, 1587, 1482, 1459, 1418, 1326, 1117, 989, 915, 854, 835, 747, 729, 620, 537, 512, 446 cm^{-1}; HRMS-EI (m/z) M^+ calcd. for $C_{23}H_{20}F_2$ 334.1528; found 334.1527.

2,2'-(2-(3,5-dichlorophenyl)prop-2-ene-1,1-diyl)bis(methylbenzene) (3bac)

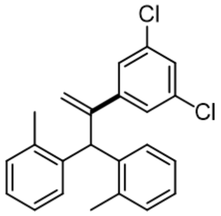

White solid, 61.7 mg, 84 % yield, m. p. 106.4-107.7 °C.
^{1}H NMR (400 MHz, $CDCl_3$) δ 7.29 (s, 2H), 7.22 – 7.11 (m, 7H), 7.00 (d, *J* = 7.5 Hz, 2H), 5.69 (s, 1H), 5.43 (s, 1H), 4.77 (s, 1H), 2.28 (s, 6H) ppm; ^{13}C NMR (100 MHz, $CDCl_3$) δ 147.4, 145.1, 139.5, 136.5, 134.9, 130.6, 128.9, 127.5, 126.8, 126.0, 124.7, 119.3, 48.9, 19.6 ppm; IR(neat) ν 3021, 2858, 1580, 1555, 1483, 1459, 1433, 1400, 1097, 927, 883, 846, 802, 758, 740, 727, 659, 611, 449 cm^{-1}; HRMS-EI (m/z) M^+ calcd. for $C_{23}H_{20}Cl_2$ 366.0937; found 366.0933.

5'-(3,3-di-*o*-tolylprop-1-en-2-yl)-1,1':3',1''-terphenyl (3bad)

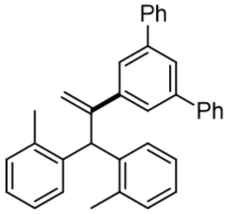

White solid, 74.3 mg, 83 % yield, m. p. 141.1-143.5 °C.
^{1}H NMR (400 MHz, $CDCl_3$) δ 7.70 (d, *J* = 12.3 Hz, 3H), 7.60 (d, *J* = 7.5 Hz, 4H), 7.49 (t, *J* = 7.6 Hz, 4H), 7.40 (t, *J* = 7.6 Hz, 2H), 7.27 – 7.16 (m, 8H), 5.87 (s, 1H), 5.70 (s, 1H), 4.82 (s, 1H), 2.36 (s, 6H) ppm;^{13}C NMR (100 MHz, $CDCl_3$) δ 149.2, 142.8, 141.7, 141.2, 140.3, 136.5, 130.4, 129.1, 128.8, 127.4, 127.2, 126.5, 125.9, 125.4, 124.1, 117.5, 49.3, 19.6 ppm; IR(neat) ν 3082, 1591, 1575, 1483, 1459, 910, 880, 755, 728, 695, 614, 481, 448 cm^{-1}; HRMS-DART (m/z) $[M+H]^+$ calcd. for $C_{35}H_{31}$ 451.2420; found 451.2421.

2,2'-(2-(3,4-difluorophenyl)prop-2-ene-1,1-diyl)bis(methylbenzene) (3bae)

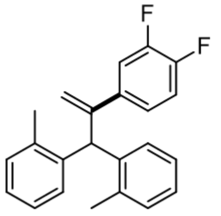

White solid, 55.5 mg, 83 % yield, m. p. 76.1-78.9 °C.

^{1}H NMR (400 MHz, $CDCl_3$) δ 7.25 – 7.11 (m, 8H), 7.07 – 7.01 (m, 3H), 5.66 (s, 1H), 5.44 (s, 1H), 4.73 (s, 1H), 2.27 (s, 6H) ppm; ^{13}C NMR (100 MHz, $CDCl_3$) δ 150.0 (dd, J = 248.3, 12.6 Hz), 149.7 (dd, J = 248.3, 12.3 Hz), 147.45, 139.80, 139.0 (dd, J = 5.2, 4.4 Hz), 136.4, 130.5, 128.8, 126.7, 125.9, 122.0 (dd, J = 6.0, 3.3 Hz), 117.0 (d, J = 17.1 Hz), 115.0 (d, J = 17.8 Hz), 49.1, 19.5 ppm; ^{19}F NMR (376 MHz, $CDCl_3$) δ -137.7 (ddd, J = 20.7, 11.9, 8.1 Hz, 1F), -139.4 (dq, J = 21.4, 6.6, 5.4 Hz, 1F) ppm; IR(neat) ν 3018, 2919, 1598, 1515, 1483, 1276, 1158, 873,814, 781, 764, 736, 611, 598, 445 cm^{-1}; HRMS-EI (m/z) M^{+} calcd. for $C_{23}H_{20}F_2$ 334.1528; found 334.1535.

2,2'-(2-(3,4-dichlorophenyl)prop-2-ene-1,1-diyl)bis(methylbenzene) (3baf)

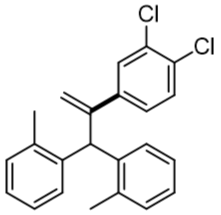

White solid, 61.9 mg, 84 % yield, m. p. 79.4-80.6 °C.

^{1}H NMR (400 MHz, $CDCl_3$) δ 7.54 (s, 1H), 7.32 (d, J = 8.4 Hz, 1H), 7.24 – 7.10 (m, 7H), 7.01 (d, J = 7.5 Hz, 2H), 5.70 (s, 1H), 5.44 (s, 1H), 4.76 (s, 1H), 2.28 (s, 6H) ppm; ^{13}C NMR (100 MHz, $CDCl_3$) δ 147.3, 141.9, 139.6, 136.4, 132.4, 131.3, 130.5, 130.3, 128.8, 128.0, 126.7, 125.9, 125.3, 118.4, 48.9, 19.5 ppm; IR(neat) ν 3018, 2951, 1466, 1375, 1131, 1102, 1052, 916, 894, 849, 754, 728, 437 cm^{-1}; HRMS-EI (m/z) M^{+} calcd. for $C_{23}H_{20}Cl_2$ 366.0937; found 366.0939.

2,2'-(2-(3,4,5-trifluorophenyl)prop-2-ene-1,1-diyl)bis(methylbenzene) (3bag)

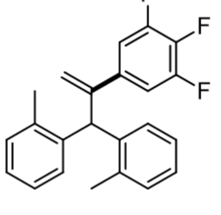

White solid, 57.7 mg, 82 % yield, m. p. 106.7-109.6 °C.

^{1}H NMR (400 MHz, $CDCl_3$) δ 7.24 – 7.11 (m, 6H), 7.06 – 6.97 (m, 4H), 5.67 (s, 1H), 5.38 (s, 1H), 4.76 (s, 1H), 2.27 (s, 6H) ppm; ^{13}C NMR (100 MHz, $CDCl_3$) δ 151.0 (ddd, J = 248.9, 10.0, 4.4 Hz), 146.8 (q, J = 2.0 Hz), 139.4, 138.0 (td, J = 7.3, 4.8 Hz), 139.0 (dt, J = 252.2 Hz, 15.4 Hz), 136.4, 130.6, 128.7, 126.8, 126.0, 118.8 (d, J = 1.6 Hz), 110.3 – 109.9 (m), 48.9, 19.4 ppm; ^{19}F NMR (376 MHz, $CDCl_3$) δ -134.4 (dd, J = 20.6, 8.9 Hz, 2F), -161.7 (tt, J = 20.6, 6.5 Hz, 1F) ppm; IR(neat) ν

3018, 2924, 1612, 1527, 1484, 1351, 1246, 1043, 785, 755, 728, 451 cm^{-1}; HRMS-EI (m/z) M^+ calcd. for $C_{23}H_{22}$ 298.1716; found 298.1714.

2,2'-(2-(3,4,5-trichlorophenyl)prop-2-ene-1,1-diyl)bis(methylbenzene) (3bah)

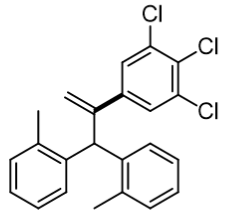

White solid, 58.9 mg, 73 % yield, m. p. 102.6-105.2 °C.

^{1}H NMR (400 MHz, $CDCl_3$) δ 7.41 (s, 2H), 7.23 – 7.11 (m, 6H), 6.98 (d, J = 7.6 Hz, 2H), 5.69 (s, 1H), 5.40 (s, 1H), 4.78 (s, 1H), 2.27 (s, 6H) ppm; ^{13}C NMR (100 MHz, $CDCl_3$) δ 146.6, 141.9, 139.2, 136.4, 134.0, 130.6, 130.1, 128.7, 126.8, 126.3, 126.0, 119.3, 48.7, 19.5 ppm; IR(neat) ν 2973, 2914, 1536, 1484, 1460, 1369, 920, 803, 760, 745, 729, 454 cm^{-1}; HRMS-DART (m/z) $[M+H]^+$ calcd. for $C_{23}H_{20}Cl_3$ 401.0625; found 401.0627.

2,2'-(2-(4-fluoro-3,5-dimethylphenyl)prop-2-ene-1,1-diyl)bis(methylbenzene) (3bai)

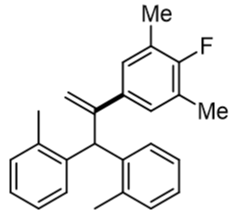

White solid, 40.3 mg, 58 % yield, m. p. 99.4-100.1 °C.

^{1}H NMR (400 MHz, $CDCl_3$) δ 7.22 – 7.09 (m, 6H), 7.07 – 7.02 (m, 4H), 5.59 (s, 1H), 5.48 (s, 1H), 4.62 (s, 1H), 2.28 (s, 6H), 2.21 (s, 6H) ppm; ^{13}C NMR (100 MHz, $CDCl_3$) δ 159.4 (d, J = 244.3 Hz), 148.6, 140.3, 137.2 (d, J = 4.2 Hz), 136.5, 130.3, 128.9, 126.5 (d, J = 4.8 Hz), 126.4, 125.8, 124.0 (d, J = 18.3 Hz), 116.6, 49.1, 19.5, 14.9 (d, J = 3.9 Hz) ppm; ^{19}F NMR (376 MHz, $CDCl_3$) δ -123.1 (s, 1F) ppm; IR(neat) ν 3017, 2923, 1484, 1459, 1180, 915, 886, 766, 743, 590, 428 cm^{-1}; HRMS-EI (m/z) M^+ calcd. for $C_{25}H_{25}F$ 344.1935; found 344.1939.

3-(3,3-di-*o*-tolylprop-1-en-2-yl)thiophene (3baj)

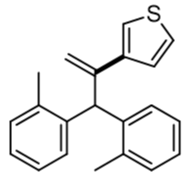

White solid, 23.1 mg, 38 % yield, m. p. 89.8-91.9 °C.

^{1}H NMR (400 MHz, $CDCl_3$) δ 7.25 – 7.09 (m, 8H), 7.04 – 6.97 (m, 3H), 5.73 (s, 1H), 5.44 (s, 1H), 4.62 (s, 1H), 2.25 (s, 6H) ppm; ^{13}C NMR (100 MHz, $CDCl_3$) δ 144.0, 143.0, 140.3, 136.5, 130.3, 128.8, 126.5, 126.0, 125.8, 125.1, 120.9, 115.4, 49.5, 19.4 ppm; IR(neat) ν 3014, 2920, 1618, 1600,

1481, 1459, 901, 879, 833, 758, 741, 727, 608, 588, 450 cm^{-1}; HRMS-EI (m/z) M$^+$ calcd. for $C_{21}H_{20}S$ 304.1280; found 304.1286.

2-(3,3-di-*o*-tolylprop-1-en-2-yl)furan (3bak)

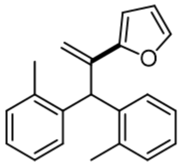

White solid, 31.9 mg, 55 % yield, m. p. 63.2-64.3 °C.

^{1}H NMR (400 MHz, $CDCl_3$) δ 7.30 (t, *J* = 1.7 Hz, 1H), 7.21 – 7.14 (m, 5H), 7.13 – 7.07 (m, 2H), 7.01 (d, *J* = 7.0 Hz, 2H), 6.53 (dd, *J* = 1.9, 0.9 Hz, 1H), 5.58 (s, 1H), 5.29 (s, 1H), 4.55 (s, 1H), 2.25 (s, 6H) ppm; ^{13}C NMR (100 MHz, $CDCl_3$) δ 142.8, 140.7, 140.0, 139.4, 136.5, 130.3, 128.7, 127.2, 126.5, 125.8, 114.3, 108.5, 49.1, 19.4 ppm; IR(neat) ν 3065, 2920, 1482, 1459, 1163, 1023, 968, 871, 832, 760, 730, 691, 560, 444 cm^{-1}; HRMS-EI (m/z) M$^+$ calcd. for $C_{21}H_{20}O$ 288.1509; found 288.1511.

5-(3,3-di-*o*-tolylprop-1-en-2-yl)benzofuran (3bal)

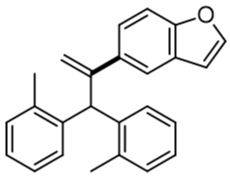

White solid, 26.3 mg, 39 % yield, m. p. 133.8-136.0 °C.

^{1}H NMR (400 MHz, $CDCl_3$) δ 7.64 (t, *J* = 1.3 Hz, 1H), 7.58 (d, *J* = 2.2 Hz, 1H), 7.41 – 7.40 (m, 2H), 7.21 – 7.18 (m, 2H), 7.17 – 7.11 (m, 4H), 7.09 (dd, *J* = 7.4, 1.9 Hz, 2H), 6.72 (d, *J* = 2.2 Hz, 1H), 5.69 (d, *J* = 1.2 Hz, 1H), 5.58 (s, 1H), 4.70 (s, 1H), 2.30 (s, 6H) ppm; ^{13}C NMR (100 MHz, $CDCl_3$) δ 154.4, 149.4, 145.3, 140.5, 137.3, 136.5, 130.3, 128.9, 127.4, 126.4, 125.8, 122.9, 118.5, 117.0, 111.1, 106.8, 49.8, 19.6 ppm; IR(neat) ν 3114, 2970, 1435, 1159, 1129, 1110, 990, 877, 787, 738, 600, 451 cm^{-1}; HRMS-EI (m/z) M$^+$ calcd. for $C_{25}H_{22}O$ 338.1665; found 338.1664.

2-(3,3-di-*o*-tolylprop-1-en-2-yl)-1-methyl-1*H*-indole (3bam)

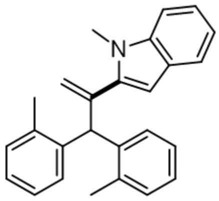

Yellow oil, 39.4 mg, 56 % yield.

^{1}H NMR (400 MHz, $CDCl_3$) δ 7.54 (d, *J* = 7.8 Hz, 1H), 7.32 (d, *J* = 8.2 Hz, 1H), 7.27 – 7.07 (m, 10H), 6.47 (s, 1H), 5.60 (s, 1H), 5.49 (s, 1H), 5.04 (s, 1H), 3.88 (s, 3H), 2.34 (s, 6H) ppm; ^{13}C NMR (100 MHz, $CDCl_3$) δ 141.9, 141.2, 139.9, 138.6, 136.6, 130.5, 128.8, 127.5, 126.6, 125.8, 121.9, 120.5, 119.8, 119.2, 109.6, 100.8, 50.9, 31.5, 19.5 ppm; IR(neat) ν 3015, 2917, 1602, 1460, 1380, 1270, 871, 761, 747, 731, 447, 436 cm^{-1}; HRMS-ESI (m/z) [M+H]$^+$ calcd. for $C_{26}H_{26}N$ 352.2060; found 352.2054.

2,2'-(2-phenylpropane-1,1-diyl)bis(methylbenzene) (TBE-M-H)

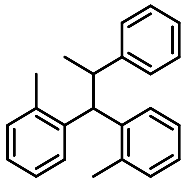

Prepared with following procedure:

3ba (59.6 mg, 0.2 mmol) was dissolved in methanol (5 ml) and hydrogenated with hydrogen over 10% Pd/C (6.0 mg) at room temperature, under 1 atm for 24 hours. At the end, the reaction mixture was filtered and methanol was removed to yield the **TBE-M-H** as a Colorless oil. Yield: 58.4 mg, 98%.

^{1}H NMR (400 MHz, $CDCl_3$) δ 7.32 (t, J = 7.6 Hz, 2H), 7.14 – 6.89 (m, 9H), 6.84 – 6.71 (m, 2H), 4.45 (d, J = 11.1 Hz, 1H), 3.47 (dq, J = 13.5, 6.9 Hz, 1H), 2.37 (s, 3H), 1.92 (s, 3H), 1.15 (d, J = 6.8 Hz, 3H) ppm; ^{13}C NMR (100 MHz, $CDCl_3$) δ 145.8, 141.7, 141.5, 136.6, 130.3, 130.0, 128.3, 128.1, 127.9, 127.7, 126.1, 125.8, 125.8, 125.5, 125.4, 48.1, 45.5, 21.1, 20.4, 19.9 ppm; HRMS-EI (m/z) M^+ calcd. for $C_{23}H_{24}$ 300.1873; found 300.1870.

8. Characterization of photophysical properties

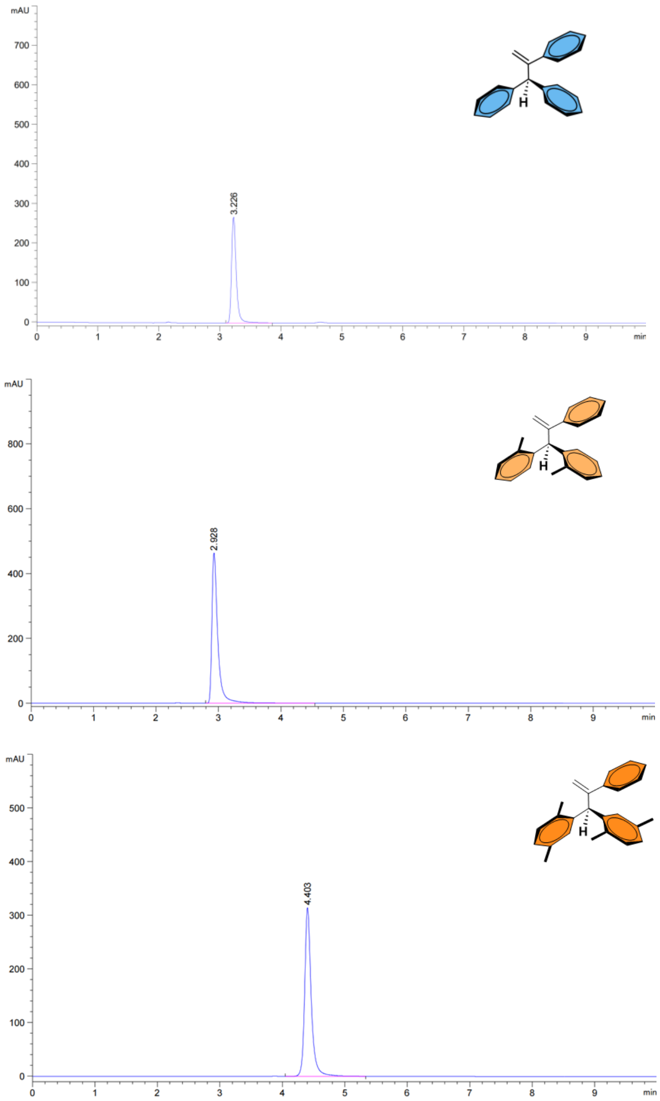

Figure S1. HPLC spectra of TBE, TBE-M, and TBE-2M.

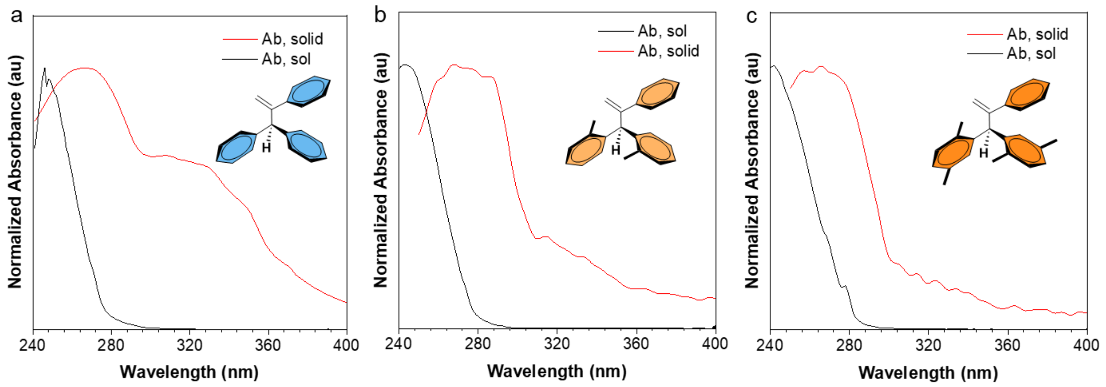

Figure S2. Normalized absorption spectra of (a) TBE (b) TBE-M and (c) TBE-2M at THF solution and solid state. Concentration=10^{-4} M.

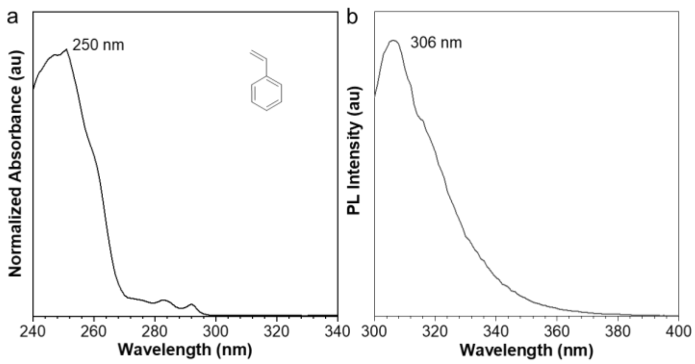

Figure S3. (a) Normalized absorption spectra of styrene in THF solution. (b) PL spectra of styrene in THF solution. λ_{ex} = 260 nm. Concentration (c) = 10^{-4} M.

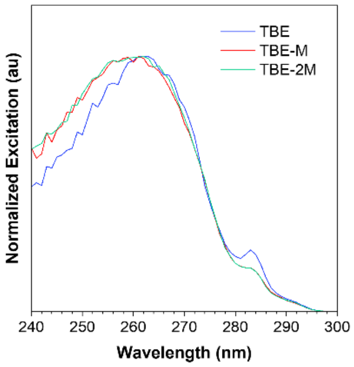

Figure S4. Normalized excitation spectra of TBE, TBE-M and TBE-2M in THF solution. Concentration (c) = 10^{-4} M.

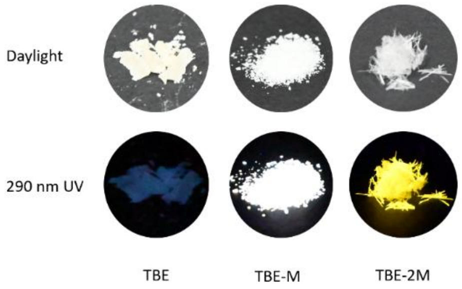

Figure S5. Photographs of TBE, TBE-M and TBE-2M under daylight and 290 nm UV.

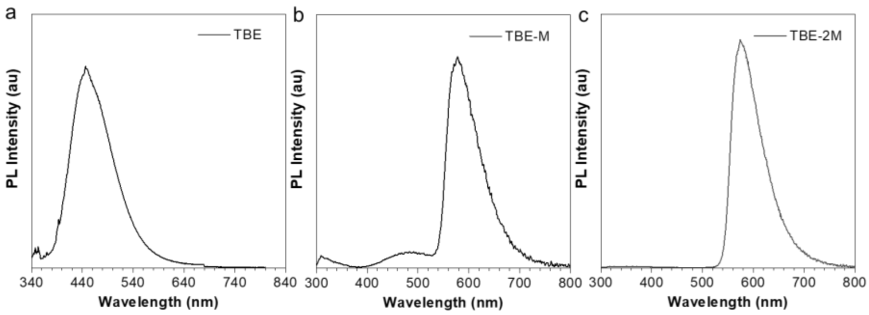

Figure S6. PL spectra of (a) TBE, (b)TBE-M and (c) TBE-2M in crystalline states. λ_{ex} = 290 nm.

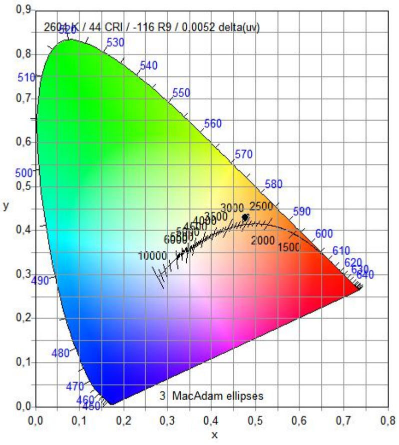

Figure S7. CIE spectrum of TBE-M under 290 nm excitation.

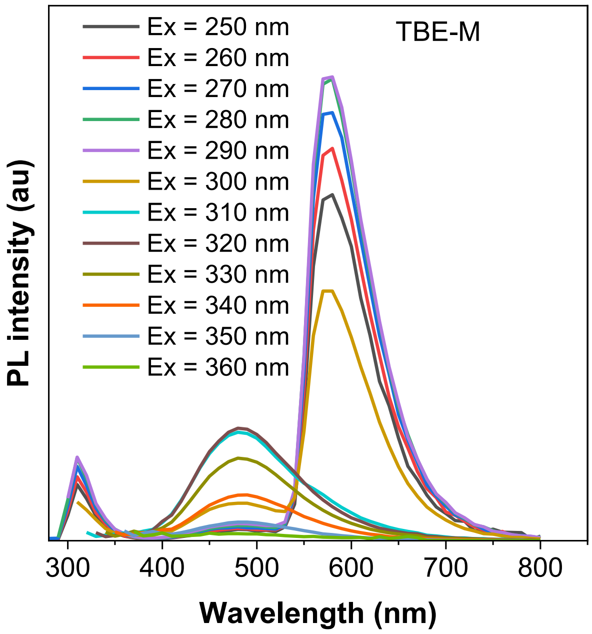

Figure S8. PL spectra of TBE-M under different excitation.

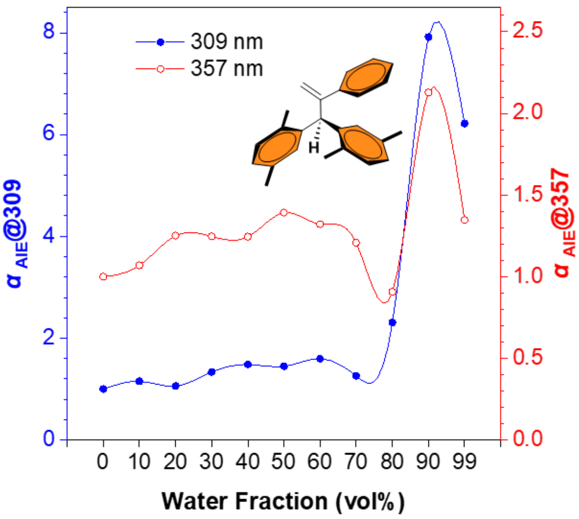

Figure S9. Plots of relative PL intensity (I/I_0) versus different water fractions (f_w). c = 10^{-4} M, λ_{ex} = 290 nm, I_0 = intensity at f_w = 0%. $\alpha_{AIE}= I/I_0$

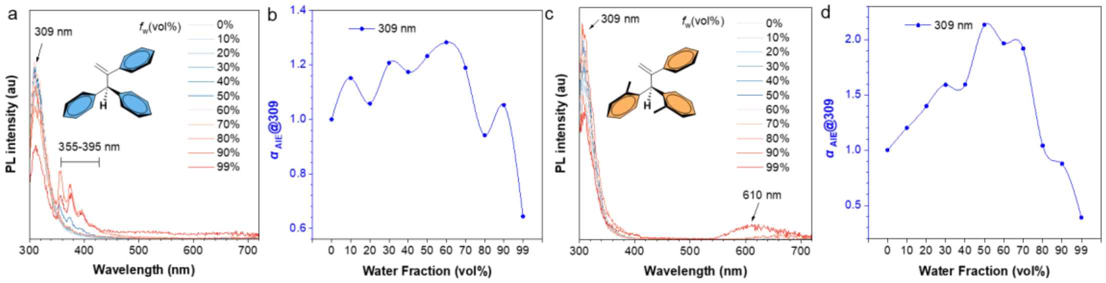

Figure S10. PL spectra of (a)TBE and (c)TBE-M in THF/water mixtures with different water fractions (f_w). Plots of relative PL intensity (I/I_0) of (b)TBE and (d)TBE-M versus different water fractions (f_w). c = 10^{-4} M, λ_{ex} = 290 nm, I_0 = intensity at f_w = 0%. $\alpha_{AIE}= I/I_0$

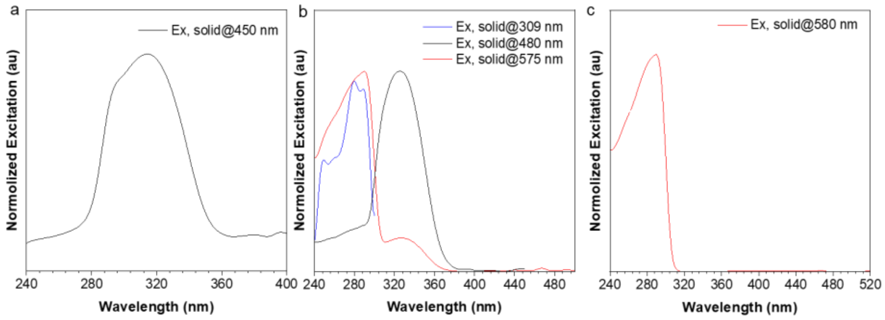

Figure S11. Normalized excitation spectra of (a) TBE measured at emission maximum of 450 nm, (b) TBE-M measured at emission maximum of 309, 480 and 575 nm, and (c) TBE-2M measured at emission maximum of 580 nm.

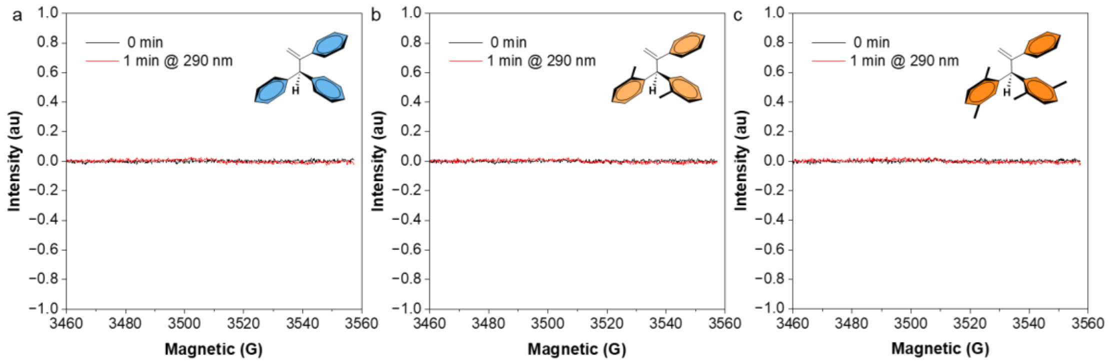

Figure S12. Solid-state ESR spectra of (a) TBE, (b) TBE-M, and (c) TBE-2M in solid state under dark or 290 nm UV light.

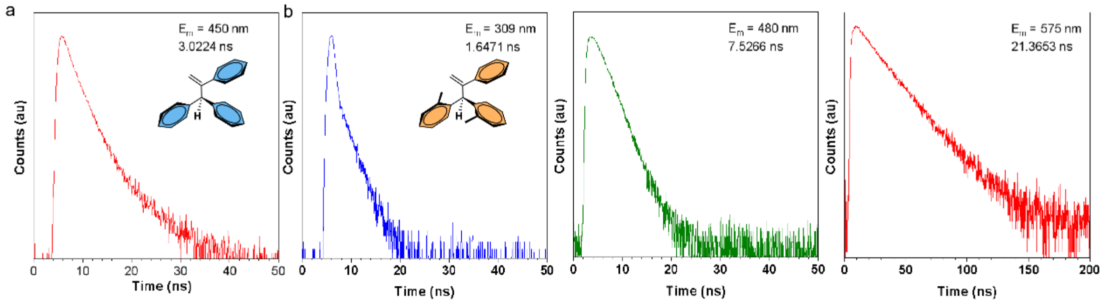

Figure S13. (a) Time-resolved PL decay curves of TBE measured at emission maximum of 450 nm. (b) Time-resolved PL decay curves of TBE-M measured at emission maximum of 309 nm, 480 nm and 575 nm, respectively.

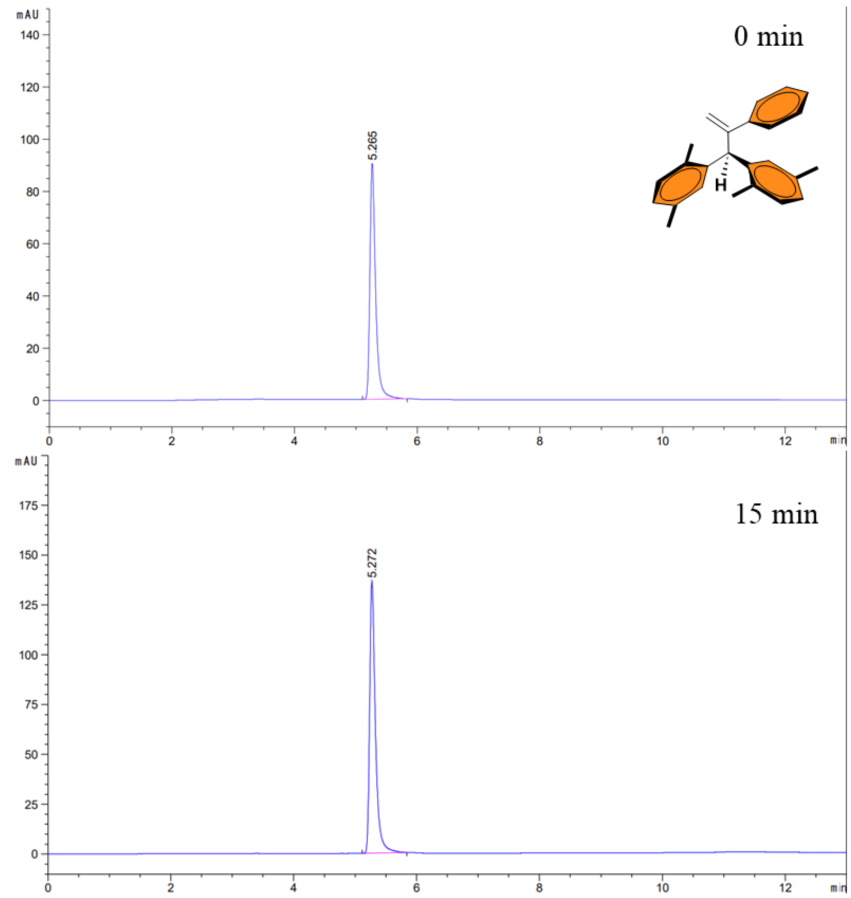

m/z	Theo. Mass	Delta (ppm)	RDB equiv.	Composition
326.2026	326.2026	-0.07	-7.0	$C_{12}H_{39}O_4Br$
	326.2029	-0.93	13.0	$C_{25}H_{26}$
	326.2020	1.78	4.0	$C_{16}H_{30}O_3N_2Si$

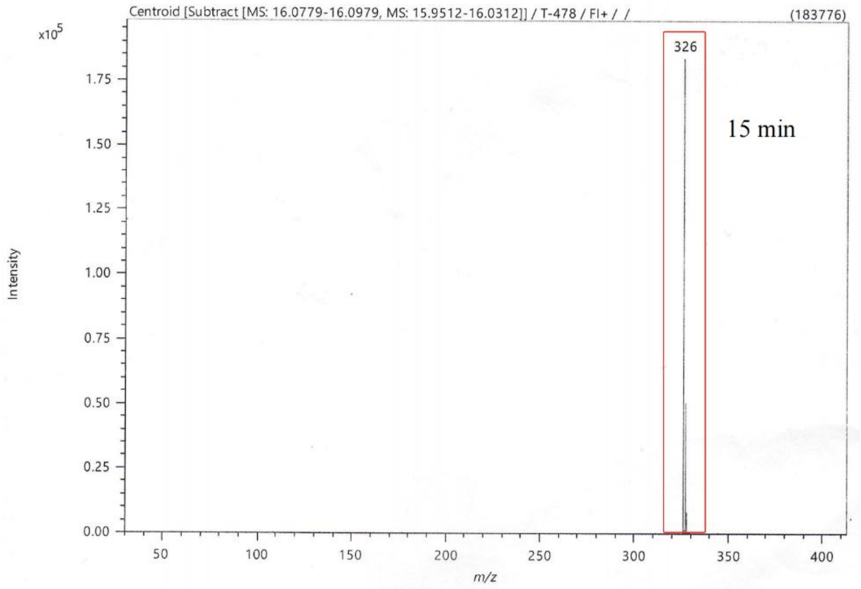

Figure S14. HPLC and FI-HRMS spectra of TBE-2M under the irradiation of 290 nm for 0 min

and 15 min.

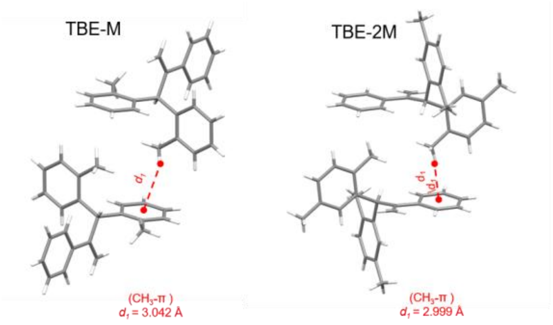

Figure S15. Single crystals of TBE-M and TBE-2M

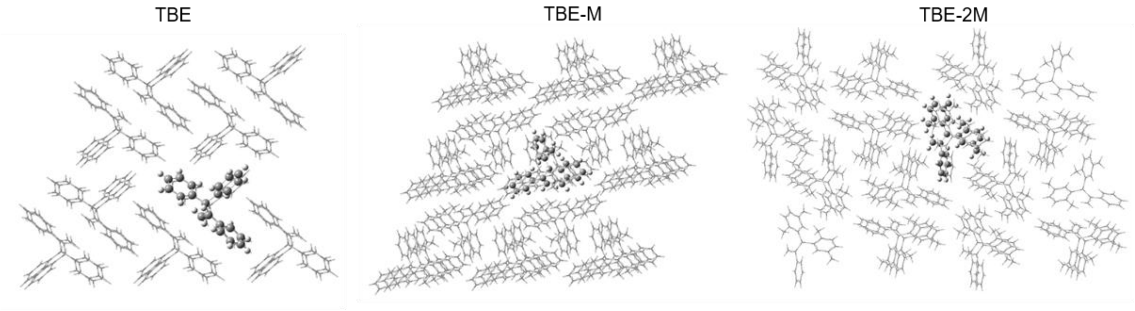

Figure S16. ONIOM calculation of TBE, TBE-M and TBE-2M monomer in crystal. The MM layer consisted of 17, 34, and 28 molecules for TBE, TBE-M, and TBE-2M, respectively.

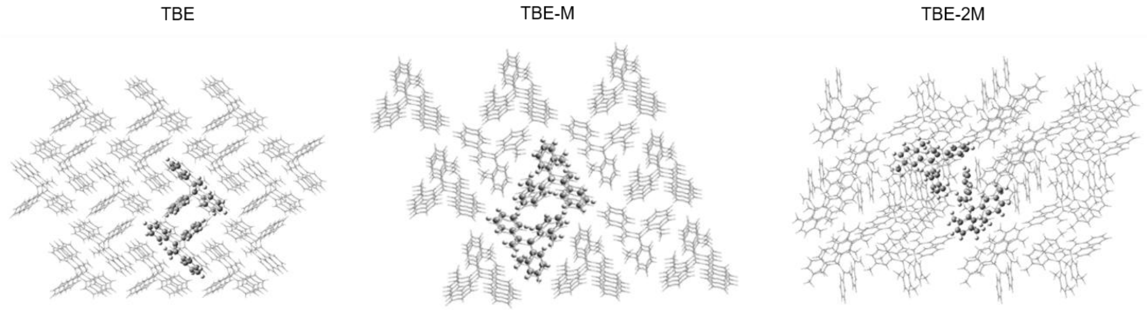

Figure S17. ONIOM calculation of TBE, TBE-M and TBE-2M dimer in crystal. The MM layer consisted of 49, 33, and 35 molecules for TBE, TBE-M, and TBE-2M, respectively.

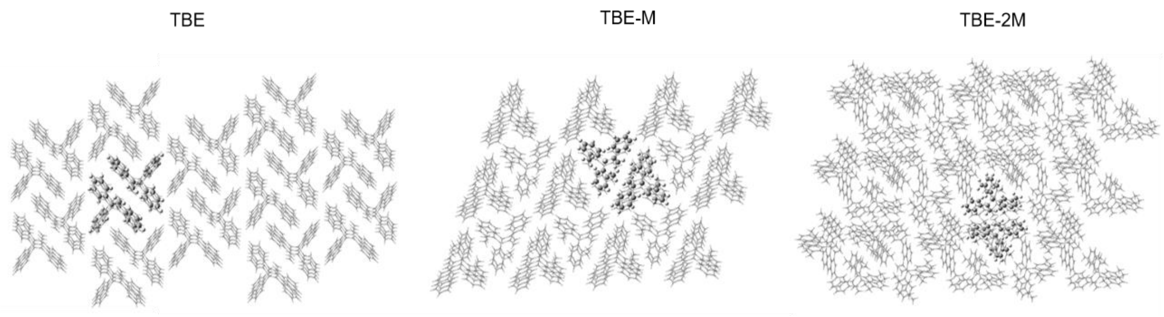

Figure S18. ONIOM calculation of TBE, TBE-M and TBE-2M dimer in crystal. The MM layer comprised 70, 46, and 73 molecules for TBE, TBE-M, and TBE-2M, respectively

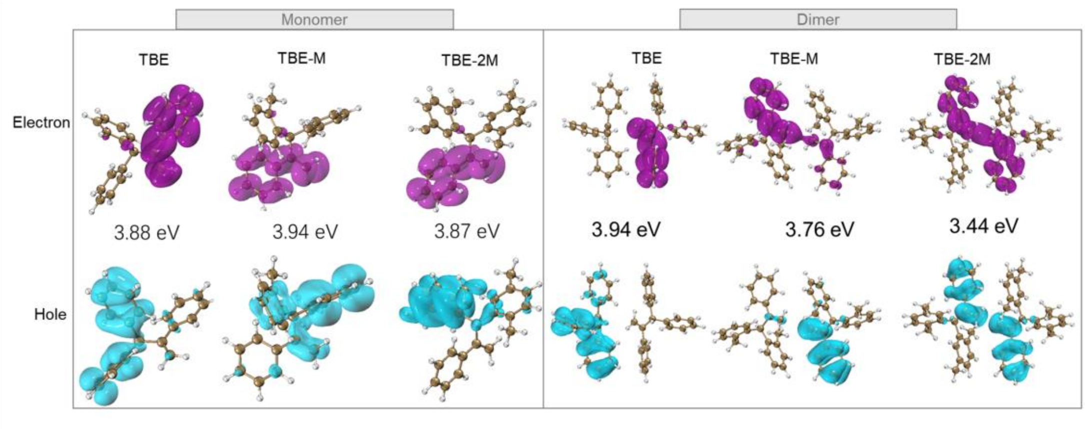

Figure S19. Electron and hole distribution of the different kind of TBE derivatives with optimized excited-state geometries.

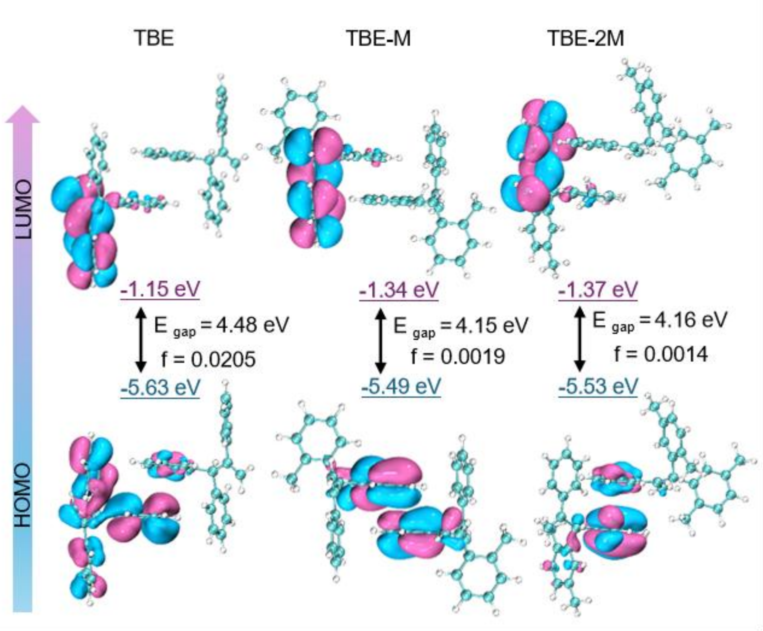

Figure S20 Frontier molecular orbitals of the dimers of different kind of TBE derivatives with optimized excited-state geometries calculated by the TD-DFT method at the B3LYP-D3/6-31G(d,p) level, Gaussian 16 program. [7]

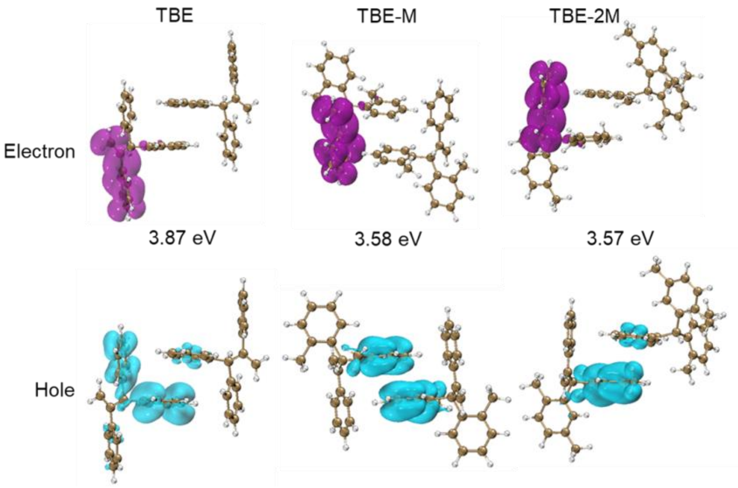

Figure S21. Electron and hole distribution of the dimers of different kind of TBE derivatives with optimized excited-state geometries.

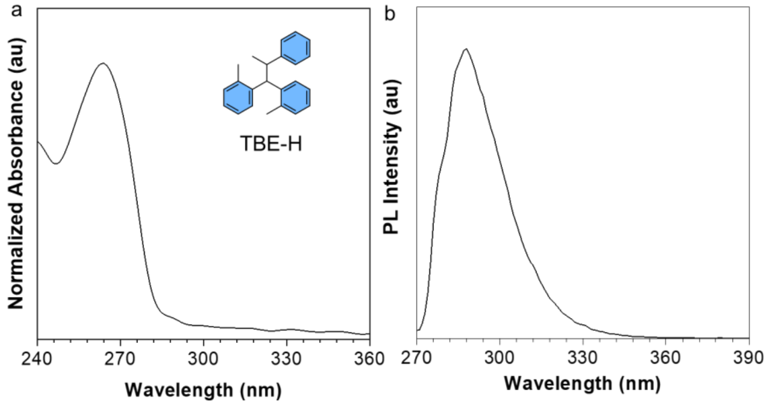

Figure S22. (a) Normalized absorption spectra of TBE-M-H. (b) PL spectra of TBE-M-H in THF solution. λ_{ex} = 260 nm. Concentration (c) = 10^{-4} M.

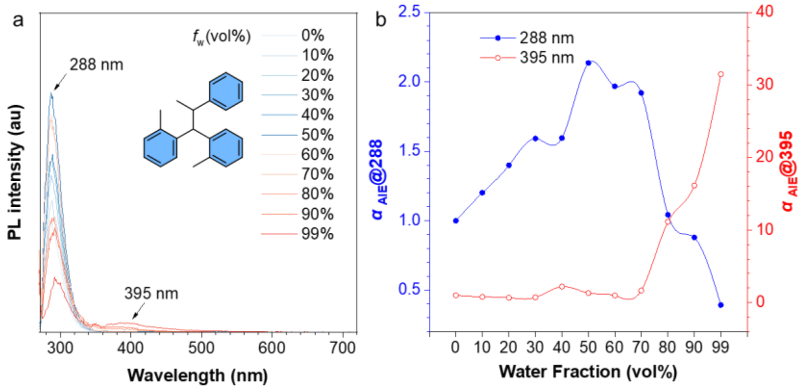

Figure S23. PL spectra of (a)TBE-M-H in THF/water mixtures with different water fractions (f_w). Plots of relative PL intensity (I/I_0) versus different water fractions (f_w). c = 10^{-4} M, λ_{ex} = 290 nm, I_0 = intensity at f_w = 0%.

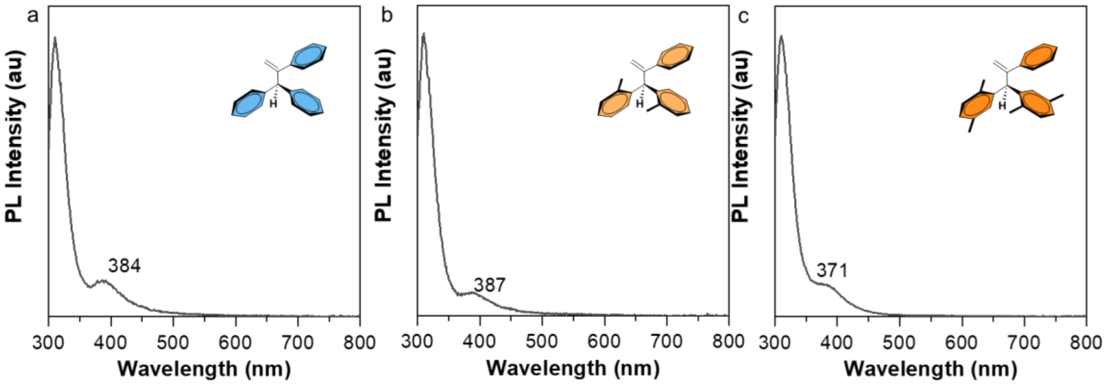

Figure S24. PL intensity spectra of (a) TBE, (b) TBE-M and (c) TBE-2M dopped in PMMA film (weight percentage = 1%). Ex = 290 nm

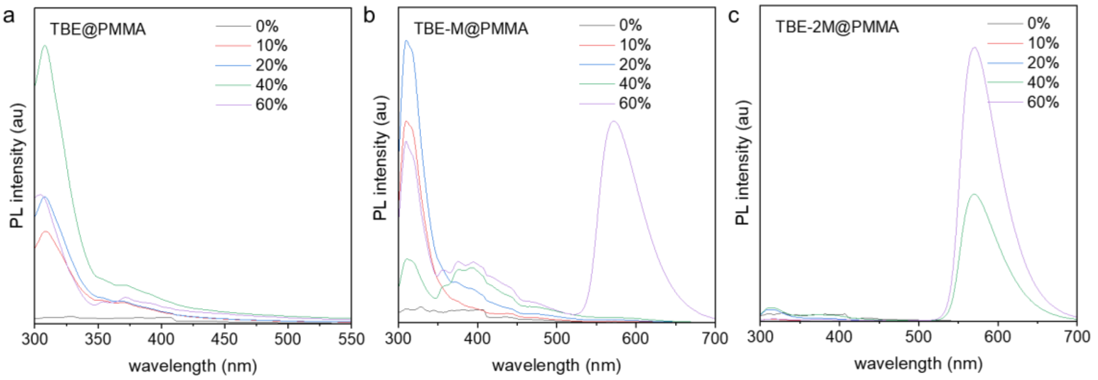

Figure S25. PL intensity spectra of (a) TBE, (b) TBE-M and (c) TBE-2M dopped in PMMA film (weight percentage = 0%, 10%, 20%, 40%, 60%). Ex = 290 nm

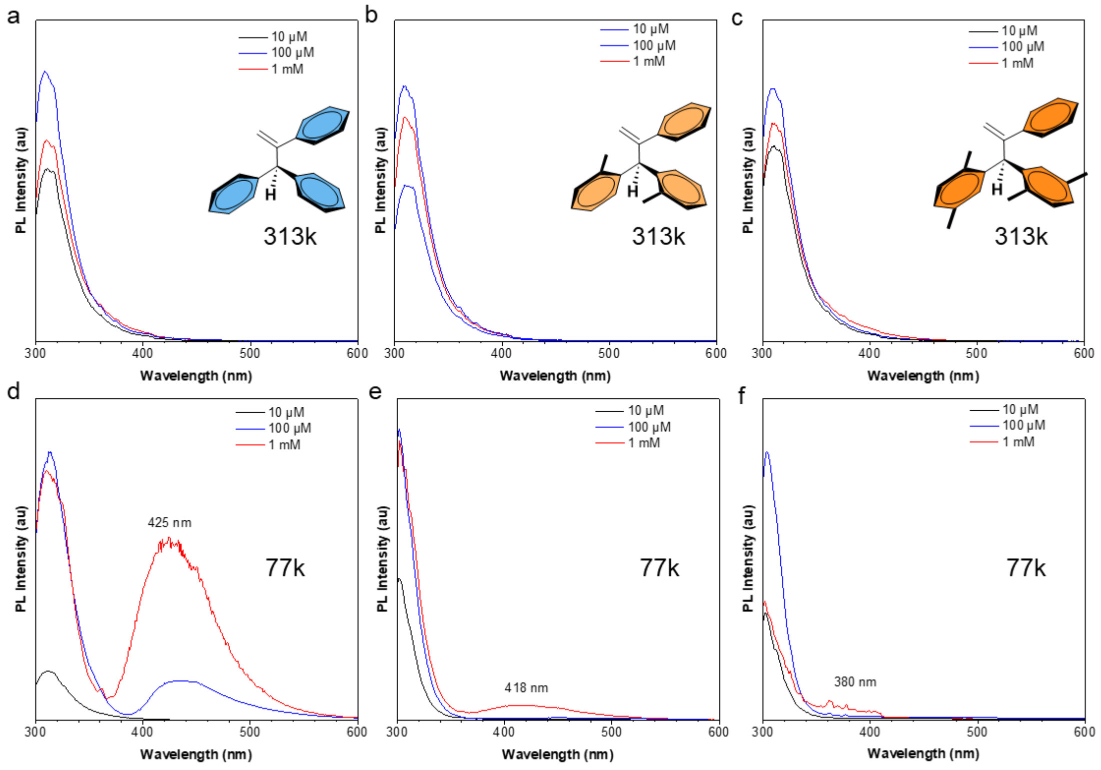

Figure S26. PL intensity spectra of (a) TBE, (b) TBE-M and (c) TBE-2M in 2-MeTHF solution measured at 313k temperature with different concentration changes. PL intensity spectra of (d) TBE, (e) TBE-M and (f) TBE-2M in 2-MeTHF solution measured at 77k temperature with different concentration changes. Ex = 290 nm

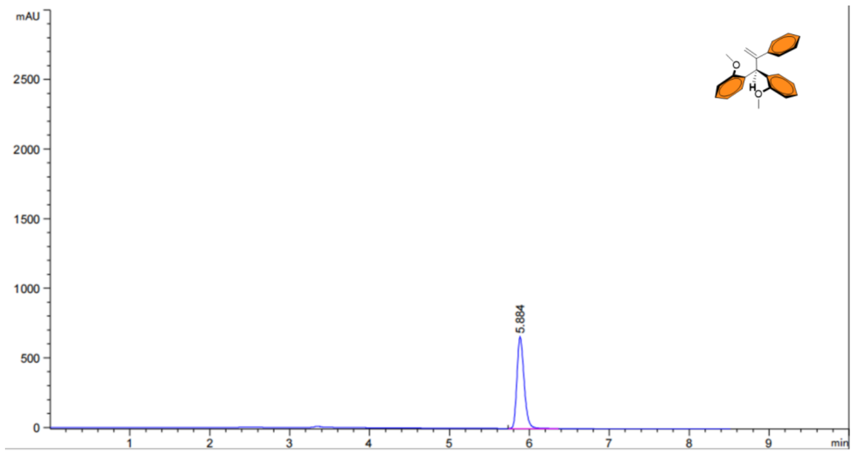

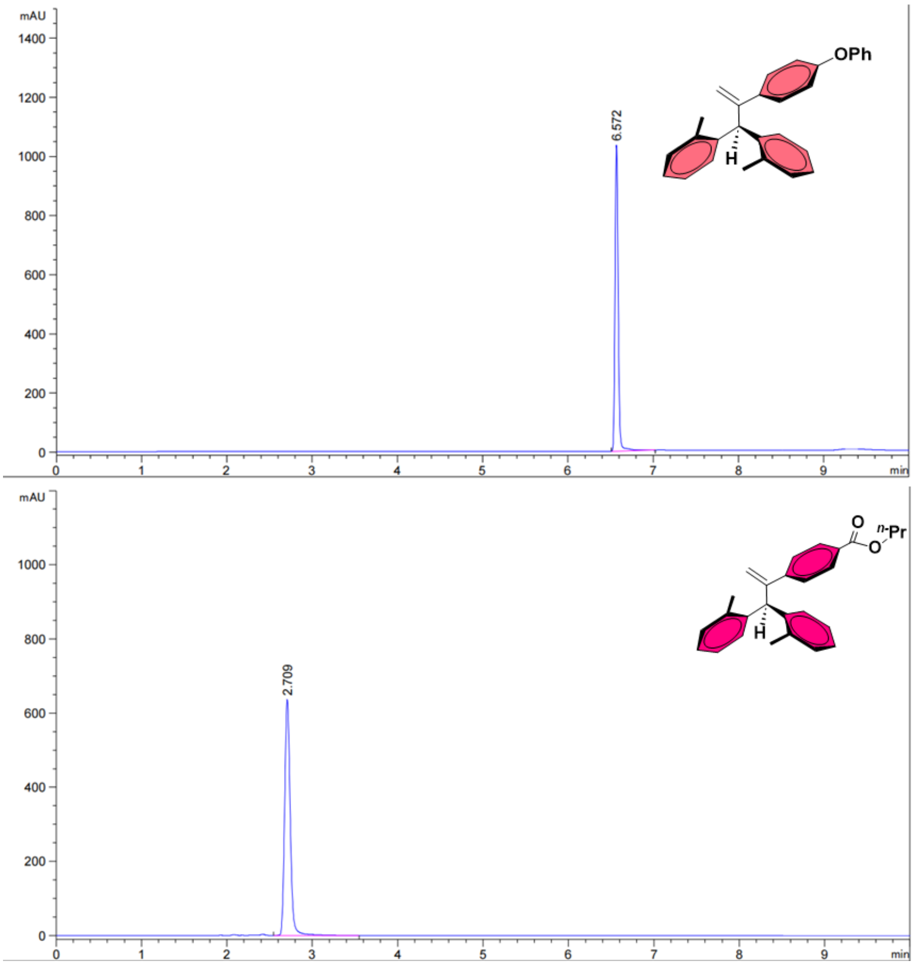

Figure S27. HPLC spectra of TBE-O, TBE-OPh, and TBE-E.

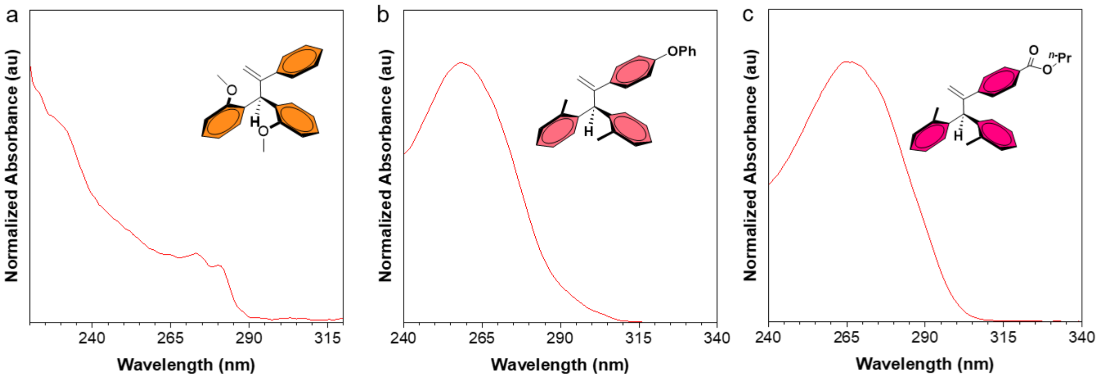

Figure S28. Absorption spectra of (a) TBE-O, (b) TBE-OPh, and (c) TBE-E in THF solution. Concentration (c) = 5 × 10 $^{-5}$ M.

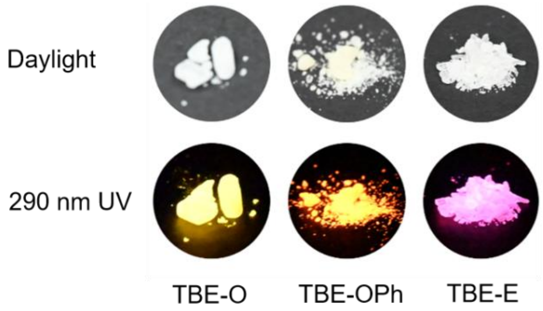

Figure S29. Photographs of TBE-O, TBE-OPh and TBE-E under daylight and 290 nm UV.

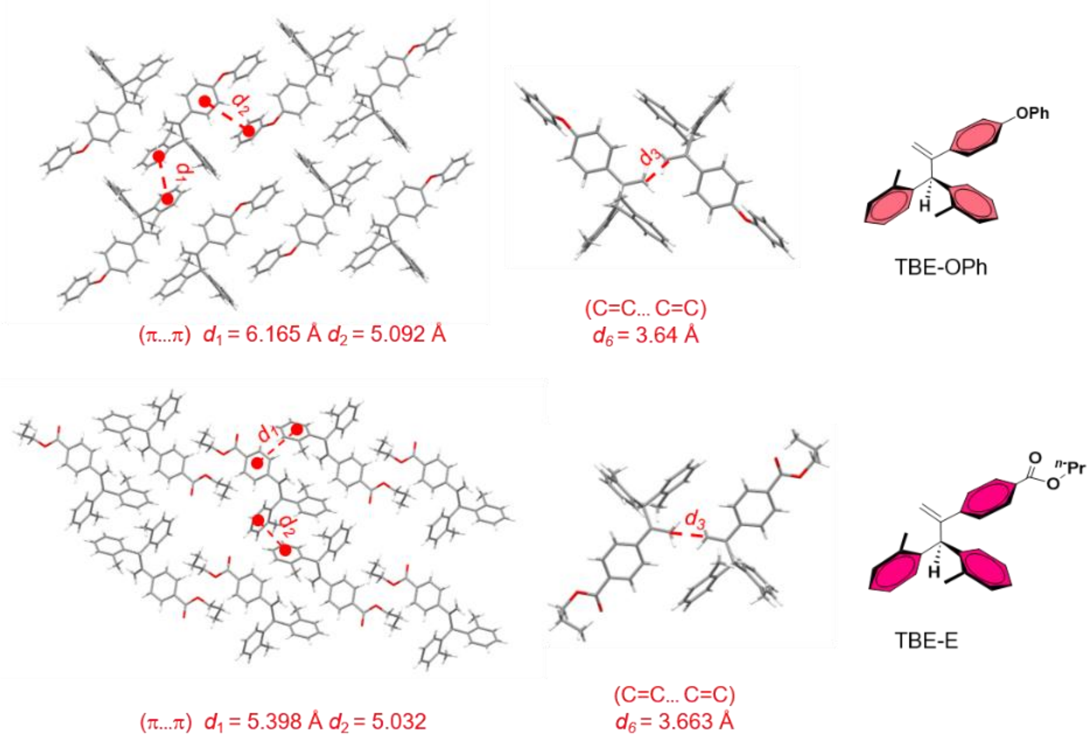

Figure S30. Crystal packing diagrams of TBE-OPh, and TBE-E

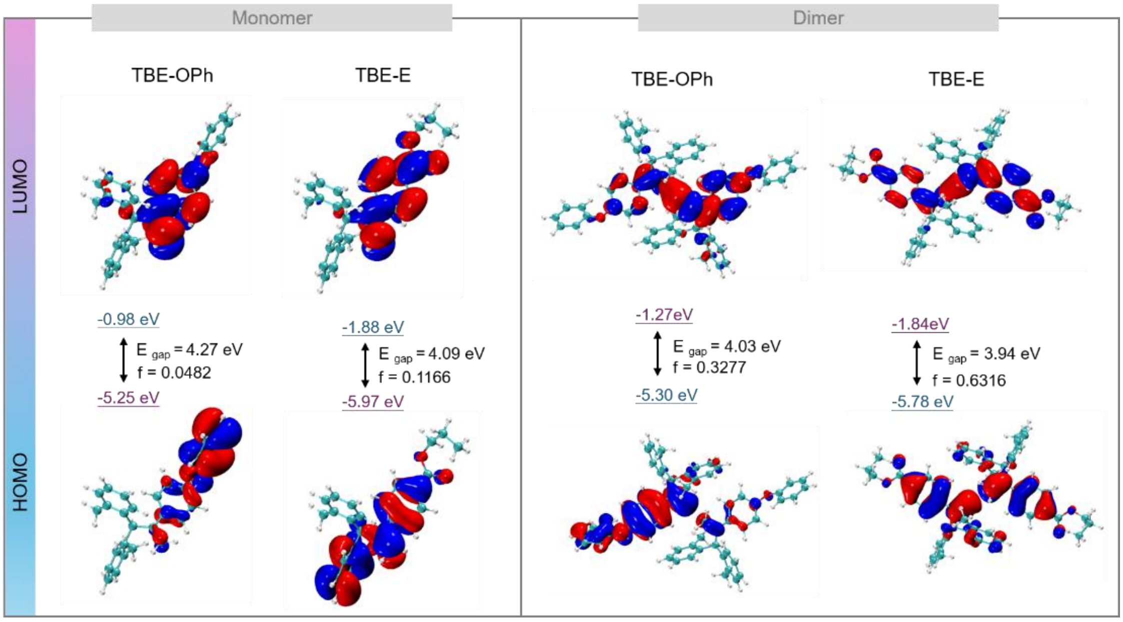

Figure S31. Theoretical calculation. (a) Frontier molecular orbitals of optimized excited-state geometries of monomer and dimer of TBE-OPh and TBE-E calculated by the ONIOM methods. The QM layer calculated by TD-DFT method at the B3LYP-D3/6-31G(d,p) level and the MM layer calculated by UFF method, Gaussian 16 program.

Table S8 Summary of the basic photophysical properties of all synthesized compounds

	Name	$\lambda_{ab, sol}$ (nm)	$\lambda_{em, sol}$ (nm)	$\lambda_{em., solid}$ (nm)
1	3aa	246	309	450
2	3ba	244	309	575
3	3ca	242	309	580
4	3da	234	308	466
5	3ea	244	308	488
6	3fa （liquid）	-	-	-
7	3ga （liquid）	-	-	-
8	3ha	235	361	453
9	3ia	243	307	565
10	3ja	236	282	580
11	3ka	246	307	492
12	3la	228	311	570
13	3ma	240	308	498
14	3na	245	327	431
15	3oa	244	337	447
16	3pa	222	326	358, 376
17	3qa	233	302	376
18	3bb	244	313	571
19	3bc	252	310	501
20	3bd	254	312	476
21	3be	245	397	483
22	3bf	245	312	474
23	3bg	251	314	494
24	3bh	254	344	495
25	3bi	278	330	455
26	3bj	263	325	437
27	3bk	267	322	640
28	3bl	277	336	500

**29**	**3bm**	**235**	**320**	**446**
**30**	**3bn**	**246**	**308**	**402**
**31**	**3bo**	**240**	**312**	**435**
**32**	**3bp**	**255**	**321**	**463**
**33**	**3bq**	**240**	**325**	**620**
**34**	**3br**	**248**	**313**	**471**
**35**	**3bs**	**242**	**333**	**450**
**36**	**3bt**	**249**	**314**	**484**
**37**	**3bu**	**248**	**311**	**586**
**38**	**3bv**	**242**	**316**	**501**
**39**	**3bw**	**245**	**330**	**444**
**40**	**3bx**	**243**	**326**	**444**
**41**	**3by**	**249**	**312**	**435**
**42**	**3bz**	**240**	**308**	**444**
**43**	**3baa**	**257**	**324**	**450**
**44**	**3bab**	**242**	**312**	**582**
**45**	**3bac**	**250**	**322**	**404**
**46**	**3bad**	**257**	**348**	**438**
**47**	**3bae**	**242**	**311**	**504**
**48**	**3baf**	**243**	**352**	**402**
**49**	**3bag**	**240**	**312**	**462**
**50**	**3bah**	**254**	**327**	**481**
**51**	**3bai**	**248**	**314**	**478**
**52**	**3baj**	**235**	**367**	**513**
**53**	**3bak**	**245**	**404**	**501**
**54**	**3bal**	**237**	**328**	**554**
**55**	**3bam**	**240**	**365**	**482**

Table S9 | Photophysical properties of TBE and its derivatives (TBE-R)[a]

TBE-R	$\lambda_{ab,sol}$ (nm)	λ_{sol} (nm)	λ_{solid} (nm)	$\lambda_{ex, solid}$ (nm)	τ_{solid} (ns)	Φ_{sol} (%)	Φ_{solid} (%)
TBE	246	309	450	315	3.0	2.5	1.5
TBE-M	244	309	309/480/575	279/329/326	1.6/7.5/21.4	4.5	19.8
TBE-2M	242	309	580	290	21.0	2.9	14.8

[a]Abbreviation: $\lambda_{ab, sol}$ = absorption maximum in THF solution, λ_{sol} = emission maximum in the THF solution, λ_{solid} = emission maximum in the solid state, $\lambda_{ex, solid}$ = excitation maximum in the solid state measured at emission maximum of 450 nm (TBE), 309 nm / 480 nm / 575 nm (TBE-M), and 580 nm (TBE-2M), τ_{solid} = emission lifetime in the solid state, Φ_{solid} = absolute luminescence quantum yield in solid state at an excitation wavelength of 290 nm. Φ_{sol} = absolute luminescence quantum yield in THF solution at an excitation wavelength of 260 nm.

Table S10. Cartesian coordinates of optimized TBE, TBE-M, and TBE-2M in the S_1 state calculated by the DFT, B3LYP-D3/6-31G(d,p), Gaussian 16 program.

TBE	X	Y	Z
C	-0.319664	0.963239	0.15526

C	0.660512	-1.336151	-0.262905
C	2.026398	-1.00745	0.017473
C	-0.356442	-0.514042	0.516738
H	-0.18662	-0.595459	1.594598
C	0.174032	1.373352	-1.115486
H	0.546002	0.605459	-1.781181
C	-1.837612	-0.758083	0.302989
C	0.24907	-2.165467	-1.305412
H	0.970601	-2.824928	-1.767765
H	-0.785612	-2.433141	-1.455671
C	2.489131	-0.50138	1.279376
H	1.788584	-0.346856	2.095715
C	-2.677058	-1.032996	1.404449
H	-2.235603	-1.116404	2.39123
C	4.3712	-0.904617	-0.739181
H	5.094427	-1.017286	-1.544367
C	-0.810693	1.949694	1.055564
H	-1.210497	1.632123	2.012711
C	-0.229279	3.679351	-0.518993
H	-0.184623	4.730842	-0.781166
C	4.803984	-0.47412	0.52736
H	5.850358	-0.27881	0.724043
C	0.226912	2.713055	-1.437852
H	0.635405	3.028832	-2.39123
C	3.040515	-1.176586	-0.988588
H	2.723751	-1.460556	-1.984858
C	3.831337	-0.275934	1.525419
H	4.137298	0.069368	2.510932
C	-2.429662	-0.628387	-0.983205
H	-1.793019	-0.416813	-1.831212
C	-0.742425	3.291979	0.731135
H	-1.084907	4.046096	1.431201
C	-4.597847	-1.137489	-0.05908
H	-5.663508	-1.28876	-0.186574
C	-3.79137	-0.830884	-1.157023
H	-4.224684	-0.750749	-2.146345
C	-4.03308	-1.242615	1.225092
H	-4.665106	-1.489775	2.071155

TBE-M	X	Y	Z
C	2.683244	-0.116684	-0.700074
H	2.506913	0.862356	-1.119931
C	3.887426	-0.758971	-0.877194
H	4.668695	-0.292159	-1.463948
C	4.106412	-2.047431	-0.300904
H	5.059786	-2.543585	-0.443667
C	3.107592	-2.658903	0.439536
H	3.280316	-3.631327	0.886427
C	1.863781	-2.043394	0.608049
C	1.630746	-0.73477	-0.000882
C	0.794157	-2.691063	1.415155
H	0.726664	-2.217614	2.403048
H	-0.173696	-2.558434	0.929459
H	1.002171	-3.750456	1.56297
C	0.282844	-0.092137	0.105757
H	-0.027711	-0.271807	1.142242
C	-0.742113	-0.828166	-0.769067
C	-0.279234	-1.401632	-1.959697
H	-0.961489	-1.837238	-2.67625
H	0.73949	-1.278125	-2.29539
C	-2.09166	-0.824616	-0.299619
C	-3.133528	-1.49373	-1.038054
H	-2.874405	-2.032356	-1.940302
C	-4.44984	-1.481263	-0.614857
H	-5.193407	-2.022751	-1.19563
C	-4.843411	-0.801727	0.549265
H	-5.877971	-0.781919	0.870376
C	-3.848947	-0.127673	1.282278
H	-4.124574	0.421126	2.17828
C	-2.526882	-0.135835	0.881299
H	-1.819368	0.44607	1.463144
C	0.327995	1.414968	-0.091502
C	-0.102972	1.960735	-1.30555
H	-0.449526	1.276888	-2.071012
C	-0.098313	3.334706	-1.513432

H	-0.44024	3.737347	-2.460962
C	0.325683	4.184409	-0.488568
H	0.322558	5.258872	-0.624638
C	0.765015	3.649533	0.71662
H	1.107917	4.31189	1.507258
C	0.786949	2.265709	0.933449
C	1.273777	1.758679	2.270474
H	1.599461	0.718072	2.252964
H	2.123858	2.354582	2.607628
H	0.492224	1.846122	3.033048

TBE-2M	X	Y	Z
C	2.157464	-1.99434	0.526684
C	3.518198	-2.241017	0.334268
H	3.920795	-3.194235	0.657233
C	4.357602	-1.31315	-0.277351
H	5.414461	-1.537176	-0.396286
C	3.850366	-0.098255	-0.741726
C	2.486843	0.152308	-0.557161
H	2.076576	1.08179	-0.937709
C	1.636828	-0.764593	0.0749
C	0.148202	-0.485625	0.268551
H	-0.072222	-0.856947	1.276336
C	-0.098448	1.003691	0.3335
C	0.311295	1.764978	1.509034
C	0.031858	3.14689	1.573838
H	0.321803	3.703118	2.458319
C	-0.577238	3.78172	0.520845
H	-0.782159	4.845532	0.562893
C	-0.958171	3.046504	-0.66074
C	-0.714831	1.671316	-0.713492
H	-1.021781	1.083706	-1.568394
C	-0.780977	-1.218122	-0.702723
C	-2.164346	-1.261996	-0.346937
C	-2.676642	-0.942406	0.958239
H	-1.997182	-0.671292	1.76152
C	-4.024802	-1.013493	1.260849

H	-4.348862	-0.779402	2.273045
C	-4.978414	-1.372369	0.290516
H	-6.035022	-1.419941	0.528063
C	-4.50601	-1.675907	-1.000972
H	-5.21525	-1.949701	-1.775415
C	-3.164705	-1.633762	-1.316815
H	-2.852366	-1.848532	-2.332214
C	-0.23625	-1.735775	-1.871779
H	-0.817152	-2.40687	-2.486746
H	0.818214	-1.666525	-2.087049
C	0.991295	1.097623	2.652397
H	0.31879	0.362885	3.110987
H	1.28417	1.819547	3.4117
H	1.86973	0.542842	2.312688
C	-1.593236	3.786449	-1.786677
H	-2.373602	4.459485	-1.417656
H	-2.017982	3.117057	-2.530199
H	-0.845884	4.423106	-2.279299
C	4.752901	0.895933	-1.427048
H	5.622299	1.132324	-0.805447
H	4.23607	1.83288	-1.640165
H	5.132169	0.502399	-2.376022
C	1.272829	-3.03875	1.154548
H	0.446826	-3.285351	0.481756
H	0.828977	-2.690642	2.095996
H	1.838914	-3.947181	1.373565

Table S11. Cartesian coordinates of optimized TBE, TBE-M and TBE-2M dimer in the S_1 state calculated by the DFT, B3LYP-D3/6-31G(d,p). in the S_1 state calculated by the DFT, B3LYP/6-31G(d,p), Gaussian 16 program. (crystalline state using fixed lower layers)

TBE dimer	X	Y	Z
C	2.823207	-0.02487	0.14026
C	5.115017	0.982546	-0.33606
C	5.927674	-0.22931	-0.02627
C	3.794712	1.123972	0.421974
H	4.041283	1.059702	1.488105
C	2.8532	-0.75692	-1.04885

H	3.62088	-0.54765	-1.78537
C	3.083069	2.456524	0.226675
C	5.549832	1.873639	-1.2347
H	6.50978	1.74393	-1.72291
H	4.982238	2.760041	-1.48901
C	6.212161	-0.60205	1.295347
H	5.842601	0.001146	2.116119
C	3.014333	3.381735	1.27249
H	3.503507	3.157014	2.214677
C	7.224663	-2.14218	-0.79138
H	7.607364	-2.74574	-1.60903
C	1.82313	-0.31432	1.078915
H	1.787458	0.254286	2.003637
C	0.920968	-2.04236	-0.35104
H	0.187468	-2.81862	-0.53807
C	7.504792	-2.49422	0.528668
H	8.106844	-3.36953	0.743152
C	1.912604	-1.76221	-1.28967
H	1.96328	-2.32964	-2.21353
C	6.447645	-1.0183	-1.06419
H	6.22392	-0.74965	-2.09179
C	6.999767	-1.71762	1.571273
H	7.221524	-1.97917	2.601211
C	2.448303	2.770119	-0.98413
H	2.481105	2.055988	-1.80119
C	0.878318	-1.3101	0.836733
H	0.102665	-1.51266	1.567368
C	1.707847	4.892945	-0.08819
H	1.175454	5.830317	-0.2078
C	1.776709	3.9814	-1.14442
H	1.299648	4.214041	-2.09011
C	2.324796	4.586588	1.123112
H	2.26518	5.283201	1.952383
C	-2.8935	-0.12787	-0.23574
C	-5.22283	-0.90598	0.403585
C	-5.9286	0.302025	0.092776
C	-3.98639	-1.15675	-0.45402
H	-4.24108	-1.13674	-1.51795

C	-2.82775	0.616477	0.98091
H	-3.61798	0.463141	1.703494
C	-3.19247	-2.44163	-0.24448
C	-5.48257	-1.69606	1.520615
H	-6.42328	-1.57508	2.040363
H	-4.9574	-2.61864	1.71359
C	-5.99414	0.877555	-1.22103
H	-5.50336	0.382103	-2.05305
C	-2.97631	-3.32509	-1.31761
H	-3.42321	-3.10437	-2.28033
C	-7.34976	2.173951	0.842281
H	-7.83751	2.702108	1.659217
C	-1.87018	0.060839	-1.21248
H	-1.91722	-0.52282	-2.12302
C	-0.80961	1.700726	0.199477
H	-0.00424	2.407534	0.363222
C	-7.44383	2.681088	-0.46641
H	-8.01547	3.574005	-0.68341
C	-1.81053	1.52121	1.183458
H	-1.77619	2.110108	2.092987
C	-6.63871	1.022477	1.116823
H	-6.54194	0.676025	2.139045
C	-6.75055	2.003681	-1.48722
H	-6.81147	2.37211	-2.50821
C	-2.59375	-2.73797	1.001457
H	-2.7597	-2.06897	1.836298
C	-0.85508	0.967471	-1.00503
H	-0.07527	1.109573	-1.74303
C	-1.67924	-4.78637	0.105566
H	-1.10382	-5.69627	0.235664
C	-1.85917	-3.90646	1.174458
H	-1.42891	-4.1322	2.14309
C	-2.24123	-4.4905	-1.14422
H	-2.10341	-5.17554	-1.97412
**TBE dimer**	**X**	**Y**	**Z**
C	-3.8332	-0.134425	0.682361
C	-2.590124	0.542986	-1.422722
C	-2.504934	1.998957	-1.143109

C	-2.59063	-0.328303	-0.175236
H	-1.708441	-0.05625	0.408224
C	-5.03606	0.384552	0.15429
H	-5.063645	0.702195	-0.879892
C	-2.549687	-1.824439	-0.354757
C	-2.685912	0.051942	-2.662027
H	-2.636727	0.708923	-3.520556
H	-2.74832	-1.011297	-2.859997
C	-1.517332	2.505481	-0.277846
H	-0.757133	1.842182	0.123061
C	-1.436073	-2.555206	0.10951
H	-0.573606	-2.035677	0.508283
C	-3.343239	4.256814	-1.469497
H	-4.050984	4.936928	-1.933585
C	-3.80983	-0.569497	2.031645
H	-2.893847	-0.991333	2.426498
C	-6.110503	0.094265	2.301638
H	-6.994727	0.189166	2.921949
C	-2.364962	4.745654	-0.602131
H	-2.313602	5.807027	-0.389037
C	-6.154575	0.512819	0.960557
H	-7.067632	0.938663	0.558676
C	-3.411121	2.892109	-1.739596
H	-4.171652	2.505637	-2.411925
C	-1.445365	3.870659	-0.017322
H	-0.655103	4.247588	0.623823
C	-3.656276	-2.518741	-0.93809
H	-4.517471	-1.95502	-1.276388
C	-4.934784	-0.448987	2.832961
H	-4.908303	-0.782502	3.864017
C	-2.498739	-4.60286	-0.624262
H	-2.4633	-5.68155	-0.72895
C	-3.617591	-3.894939	-1.081246
H	-4.448007	-4.421574	-1.536065
C	-1.402214	-3.92906	-0.035675
H	-0.515786	-4.474797	0.266402
C	4.334354	0.100808	0.226804
C	1.89298	-0.497408	-0.088642

C	2.01004	-1.919395	0.106405
C	2.893458	0.371815	0.683265
H	2.850691	0.091041	1.741598
C	4.619887	-0.391708	-1.050907
H	3.797436	-0.643408	-1.710371
C	2.656428	1.877945	0.642901
C	1.067563	0.061583	-1.052144
H	0.395105	-0.565543	-1.620369
H	1.115217	1.101135	-1.335821
C	2.355121	-2.533406	1.35944
H	2.552022	-1.904883	2.2218
C	2.30244	2.574721	1.802366
H	2.168281	2.021954	2.727296
C	1.897085	-4.204979	-0.827888
H	1.790447	-4.847097	-1.701218
C	5.401558	0.398573	1.081644
H	5.190227	0.786869	2.074393
C	6.997186	-0.268535	-0.605932
H	8.022635	-0.413216	-0.931661
C	2.156688	-4.782357	0.433224
H	2.259627	-5.852942	0.553607
C	5.939657	-0.57428	-1.463559
H	6.142406	-0.965196	-2.456385
C	1.794461	-2.836883	-0.986961
H	1.641824	-2.418705	-1.976822
C	2.398699	-3.903707	1.517146
H	2.636351	-4.31645	2.495039
C	2.869577	2.620915	-0.528852
H	3.188321	2.104578	-1.427594
C	6.722748	0.218698	0.672351
H	7.535796	0.461435	1.350383
C	2.348676	4.684519	0.621737
H	2.251952	5.765485	0.621562
C	2.701234	4.002507	-0.545371
H	2.871292	4.552449	-1.464996
C	2.158545	3.964829	1.799321
H	1.927233	4.489975	2.720252

TBE-M dimer	X	Y	Z
C	-5.11168	1.963296	0.709983
H	-4.10846	2.319171	0.90218
C	-6.20148	2.741942	1.099369
H	-6.03011	3.687502	1.60268
C	-7.4929	2.286977	0.849031
H	-8.35697	2.867177	1.155934
C	-7.6745	1.066419	0.197682
H	-8.68106	0.707368	-0.00068
C	-6.59038	0.272321	-0.1849
C	-5.28184	0.725259	0.087391
C	-6.81354	-1.06362	-0.83899
H	-6.53503	-1.05483	-1.90028
H	-6.19871	-1.82026	-0.34817
H	-7.86116	-1.35972	-0.77127
C	-4.09742	-0.17132	-0.26453
H	-4.31884	-0.57184	-1.25952
C	-3.92087	-1.37084	0.680538
C	-4.33632	-1.22625	1.998681
H	-4.19964	-2.01481	2.725358
H	-4.88543	-0.35646	2.327349
C	-3.23818	-2.50839	0.143913
C	-2.98259	-3.6886	0.940893
H	-3.34329	-3.71841	1.961652
C	-2.34429	-4.80041	0.424562
H	-2.22135	-5.67639	1.058051
C	-1.86911	-4.83894	-0.90079
H	-1.36266	-5.71041	-1.29711
C	-2.08462	-3.69671	-1.69569
H	-1.74167	-3.69139	-2.72645
C	-2.73407	-2.5798	-1.203
H	-2.86903	-1.73839	-1.87357
C	-2.80515	0.621779	-0.41527
C	-1.81533	0.528567	0.546841
H	-2.01229	-0.09415	1.408135
C	-0.58832	1.209899	0.411231
H	0.155344	1.146281	1.195417
C	-0.35185	2.041245	-0.71194

H	0.582437	2.580919	-0.80121
C	-1.3363	2.154545	-1.66704
H	-1.18736	2.792922	-2.53282
C	-2.56993	1.459209	-1.54381
C	-3.58321	1.605754	-2.63475
H	-4.60228	1.469873	-2.27601
H	-3.50021	2.586015	-3.10696
H	-3.3941	0.853018	-3.41154
C	5.108357	-1.98509	-0.65401
H	4.105229	-2.3711	-0.75951
C	6.196535	-2.75499	-1.06077
H	6.022419	-3.72013	-1.52136
C	7.486521	-2.26853	-0.87296
H	8.349595	-2.84082	-1.19366
C	7.671586	-1.03133	-0.25636
H	8.678807	-0.66048	-0.08655
C	6.589113	-0.24124	0.137324
C	5.281805	-0.72328	-0.08702
C	6.828827	1.105249	0.768135
H	6.456312	1.149438	1.798799
H	6.316485	1.890265	0.206391
H	7.894092	1.341821	0.790163
C	4.08443	0.1522	0.271118
H	4.310468	0.581841	1.252342
C	3.908906	1.33459	-0.68903
C	4.330195	1.243625	-1.95874
H	4.177647	2.034781	-2.68209
H	4.84185	0.357899	-2.31281
C	3.214585	2.535418	-0.14995
C	3.112142	3.728033	-0.89182
H	3.556053	3.786109	-1.87682
C	2.473811	4.852009	-0.37746
H	2.432632	5.761652	-0.96784
C	1.906543	4.82138	0.898567
H	1.409175	5.696517	1.302361
C	1.997276	3.652291	1.648889
H	1.573054	3.602846	2.64485
C	2.641721	2.529481	1.133758

H	2.677209	1.636014	1.743513
C	2.777371	-0.6296	0.424749
C	1.793637	-0.59096	-0.55545
H	1.974313	-0.01053	-1.44755
C	0.577568	-1.28996	-0.41401
H	-0.1632	-1.28229	-1.20365
C	0.349506	-2.07765	0.729873
H	-0.57612	-2.63378	0.826806
C	1.336698	-2.13699	1.699144
H	1.190229	-2.75881	2.577076
C	2.55201	-1.42776	1.574461
C	3.586086	-1.54006	2.6581
H	4.599064	-1.56014	2.252218
H	3.432817	-2.44899	3.238924
H	3.525702	-0.69039	3.351601
**TBE-M dimer**	**X**	**Y**	**Z**
C	2.942339	2.686511	-0.412891
H	3.57912	2.469168	-1.259883
C	2.238911	3.884772	-0.375284
H	2.315608	4.572421	-1.209192
C	1.432032	4.170568	0.726404
H	0.851451	5.084153	0.76768
C	1.361152	3.26324	1.776648
H	0.73583	3.486	2.635399
C	2.048072	2.039964	1.749534
C	2.847091	1.747162	0.622383
C	1.849363	1.104517	2.921815
H	1.943422	1.654528	3.862502
H	2.560815	0.277287	2.95896
H	0.843615	0.673049	2.893886
C	3.57003	0.400408	0.491236
H	3.902197	0.134115	1.498574
C	2.622562	-0.732392	0.067799
C	1.405393	-0.431334	-0.525892
H	0.682707	-1.179882	-0.819169
H	1.121715	0.594668	-0.707485
C	2.991092	-2.107961	0.330819
C	2.120065	-3.201062	0.002898

H	1.158883	-3.023664	-0.460592
C	2.46273	-4.502672	0.317795
H	1.770571	-5.305734	0.094009
C	3.683024	-4.779576	0.947464
H	3.950527	-5.801281	1.194798
C	4.567561	-3.729787	1.262258
H	5.514686	-3.943747	1.742562
C	4.234771	-2.427098	0.959654
H	4.937523	-1.639036	1.19043
C	4.839021	0.462212	-0.371629
C	4.840186	0.014484	-1.697728
H	3.924206	-0.379183	-2.122153
C	5.99025	0.068226	-2.485265
H	5.961204	-0.283216	-3.512255
C	7.167439	0.57081	-1.937249
H	8.076962	0.616146	-2.524521
C	7.173531	1.03559	-0.623282
H	8.087272	1.45192	-0.206846
C	6.024544	1.000684	0.175186
C	6.08258	1.544839	1.583546
H	5.183629	2.107818	1.843665
H	6.932525	2.219002	1.687933
H	6.202411	0.749969	2.332007
C	-2.876473	-2.717637	0.409107
H	-3.545055	-2.570232	1.247063
C	-2.160792	-3.910396	0.298776
H	-2.272094	-4.668016	1.0672
C	-1.334082	-4.119433	-0.803191
H	-0.802296	-5.058908	-0.929308
C	-1.232455	-3.121125	-1.778381
H	-0.619209	-3.290489	-2.661612
C	-1.931162	-1.911342	-1.662552
C	-2.770975	-1.699905	-0.543421
C	-1.799312	-0.849702	-2.724551
H	-2.700254	-0.783135	-3.343461
H	-1.667297	0.131905	-2.264642
H	-0.959243	-1.055395	-3.390341
C	-3.542671	-0.382499	-0.401967

H	-3.860883	-0.129623	-1.417824
C	-2.664573	0.785612	0.082803
C	-1.603634	0.502446	0.956534
H	-1.111503	1.2998	1.501346
H	-1.478141	-0.495858	1.356471
C	-3.025964	2.106598	-0.356412
C	-2.247281	3.264743	0.003131
H	-1.356744	3.131556	0.605053
C	-2.577719	4.538447	-0.426467
H	-1.941695	5.375307	-0.140629
C	-3.70701	4.777274	-1.226637
H	-3.971689	5.778015	-1.549513
C	-4.500614	3.670302	-1.574278
H	-5.39494	3.818566	-2.172504
C	-4.182338	2.389752	-1.158359
H	-4.866024	1.589528	-1.419366
C	-4.838982	-0.54218	0.406756
C	-4.908209	-0.089717	1.729986
H	-4.017945	0.353151	2.16048
C	-6.085398	-0.177818	2.469923
H	-6.108275	0.190657	3.491503
C	-7.229353	-0.713794	1.880532
H	-8.162208	-0.775598	2.428988
C	-7.170126	-1.18241	0.57124
H	-8.057154	-1.617508	0.11539
C	-5.98789	-1.116943	-0.17715
C	-5.999592	-1.646976	-1.593017
H	-5.027626	-2.026388	-1.913188
H	-6.710891	-2.470534	-1.676705
H	-6.308507	-0.873721	-2.30647

**TBE-2M dimer**	**X**	**Y**	**Z**
C	-6.9435	-0.44244	-1.06928
C	-8.07885	-1.24528	-0.93666
H	-8.9438	-1.03355	-1.5594
C	-8.12522	-2.30451	-0.0307
H	-9.01887	-2.91817	0.04255

C	-7.02987	-2.58156	0.787632
C	-5.89301	-1.77282	0.659114
H	-5.03521	-1.98071	1.289722
C	-5.82523	-0.71826	-0.25529
C	-4.56764	0.13339	-0.42412
H	-4.50088	0.309719	-1.50012
C	-3.31856	-0.65372	-0.072
C	-2.82359	-1.67748	-0.9592
C	-1.62343	-2.36459	-0.63517
H	-1.27052	-3.11822	-1.32048
C	-0.92633	-2.0827	0.508187
H	-0.00273	-2.59975	0.739443
C	-1.43098	-1.09915	1.418918
C	-2.615	-0.39582	1.081629
H	-2.99969	0.372941	1.739943
C	-4.58743	1.499546	0.267954
C	-3.76801	2.519839	-0.31146
C	-3.11538	2.408088	-1.58883
H	-3.24761	1.516533	-2.19286
C	-2.33673	3.420665	-2.11699
H	-1.89898	3.286198	-3.10245
C	-2.11043	4.620762	-1.41479
H	-1.4699	5.393269	-1.82078
C	-2.7312	4.761823	-0.15837
H	-2.59012	5.681524	0.404969
C	-3.52043	3.764773	0.380854
H	-3.96073	3.916076	1.358768
C	-6.91993	0.70405	-2.04498
H	-6.2394	0.514943	-2.8844
H	-6.56934	1.612724	-1.5508
H	-7.91293	0.883943	-2.46007
C	-7.0647	-3.7221	1.774916
H	-7.50181	-3.41713	2.731139
H	-6.06055	-4.10233	1.981702
H	-7.66513	-4.55496	1.399416
C	-5.26055	1.591124	1.47911
H	-5.30291	2.520424	2.02648
H	-5.89119	0.789483	1.831593

C	-3.51421	-1.99042	-2.24093
H	-4.59378	-2.0716	-2.0956
H	-3.12778	-2.90634	-2.68998
H	-3.35761	-1.16828	-2.95141
C	-0.7459	-0.83698	2.706718
H	-1.45001	-0.93865	3.540356
H	-0.39703	0.20532	2.729649
H	0.108346	-1.49493	2.86795
C	4.353898	-3.02901	0.334137
C	4.935357	-4.15898	-0.24683
H	4.647206	-5.13732	0.121311
C	5.860964	-4.0569	-1.27937
H	6.305165	-4.95644	-1.69753
C	6.234372	-2.80387	-1.77279
C	5.651173	-1.67051	-1.19556
H	5.934117	-0.68737	-1.55586
C	4.722215	-1.75901	-0.15333
C	4.089223	-0.50252	0.44467
H	3.979698	-0.7125	1.515183
C	4.998834	0.719658	0.345213
C	6.13609	0.802222	1.176254
C	6.964095	1.922075	1.06138
H	7.839583	1.997667	1.701304
C	6.693648	2.936531	0.147114
H	7.352867	3.797558	0.088659
C	5.576734	2.861216	-0.68789
C	4.742189	1.743328	-0.56995
H	3.860837	1.673471	-1.19913
C	2.664179	-0.26137	-0.06406
C	1.829046	0.748357	0.646801
C	2.108983	1.15291	1.961218
H	2.96435	0.746866	2.488553
C	1.305777	2.095957	2.609312
H	1.553171	2.398791	3.62244
C	0.211233	2.668155	1.955243
H	-0.39455	3.423448	2.444375
C	-0.08727	2.282109	0.647553
H	-0.92226	2.726907	0.119699

C	0.718804	1.341087	0.009723
H	0.505391	1.098705	-1.0224
C	2.170436	-0.97232	-1.08852
H	1.140189	-0.86968	-1.4015
H	2.779328	-1.69289	-1.61835
C	6.48245	-0.28543	2.162112
H	5.669616	-0.4722	2.874923
H	7.366219	-0.00981	2.739894
H	6.687308	-1.23558	1.657536
C	5.287805	3.95461	-1.68459
H	5.60943	4.925307	-1.30011
H	4.222466	4.023794	-1.90827
H	5.813129	3.788102	-2.63201
C	7.237916	-2.69481	-2.89374
H	8.154246	-3.24526	-2.65803
H	7.516317	-1.65813	-3.08534
H	6.843455	-3.11425	-3.82569
C	3.354321	-3.19924	1.451845
H	2.405177	-2.7069	1.214491
H	3.71374	-2.76519	2.392932
H	3.15585	-4.25848	1.634521
**TBE-2M dimer**	**X**	**Y**	**Z**
C	2.029129	-2.050396	-1.916058
C	1.686798	-3.40655	-1.974134
H	1.149424	-3.768587	-2.846879
C	2.039475	-4.296593	-0.959497
H	1.780139	-5.348553	-1.043811
C	2.733852	-3.847275	0.166265
C	3.081129	-2.491857	0.222385
H	3.656958	-2.13317	1.07011
C	2.75528	-1.588439	-0.79465
C	3.222681	-0.130468	-0.739653
H	3.412064	0.136213	-1.783706
C	4.583607	0.022995	-0.031724
C	5.763012	-0.369632	-0.704926
C	6.984294	-0.225495	-0.043626
H	7.889498	-0.525158	-0.558819
C	7.069469	0.291631	1.245945

H	8.041877	0.395027	1.722763
C	5.914071	0.681849	1.92512
C	4.68442	0.540953	1.263966
H	3.773985	0.855501	1.765359
C	2.158748	0.857064	-0.242588
C	2.293182	2.243352	-0.635968
C	3.294828	2.684782	-1.557044
H	3.993306	1.975459	-1.984336
C	3.415659	4.012545	-1.924744
H	4.18157	4.295408	-2.64037
C	2.567827	4.991101	-1.38086
H	2.697507	6.035391	-1.646274
C	1.574516	4.593969	-0.476677
H	0.89908	5.333059	-0.052939
C	1.430505	3.263224	-0.113722
H	0.641433	3.002696	0.581206
C	1.647142	-1.131342	-3.054298
H	2.525758	-0.682907	-3.533622
H	1.020647	-0.30006	-2.716318
H	1.09942	-1.679067	-3.824636
C	3.117445	-4.793111	1.279732
H	2.323869	-4.871385	2.032
H	4.024339	-4.459365	1.793109
H	3.302014	-5.802835	0.90061
C	1.135867	0.403327	0.607146
H	0.500631	1.091458	1.147017
H	1.16118	-0.612701	0.971152
C	5.751649	-0.920269	-2.111897
H	5.045095	-1.74871	-2.222303
H	6.745668	-1.287325	-2.382381
H	5.472391	-0.156794	-2.851086
C	5.998043	1.223504	3.332816
H	5.743445	0.462546	4.079912
H	5.311368	2.059326	3.487695
H	7.010095	1.574467	3.560098
C	-2.0892	2.064505	1.972022
C	-1.737193	3.418187	2.02165
H	-1.214068	3.784694	2.900807

C	-2.057636	4.299486	0.98899
H	-1.78565	5.348734	1.065537
C	-2.737665	3.844263	-0.142745
C	-3.084932	2.489061	-0.195822
H	-3.643272	2.125273	-1.052733
C	-2.784165	1.593223	0.834838
C	-3.237397	0.13048	0.761937
H	-3.431284	-0.157402	1.800534
C	-4.591732	-0.027172	0.037995
C	-5.779513	0.362892	0.698723
C	-6.993577	0.207598	0.026195
H	-7.904947	0.50499	0.530744
C	-7.065864	-0.320169	-1.259245
H	-8.034396	-0.438261	-1.739947
C	-5.903083	-0.707448	-1.926856
C	-4.679588	-0.552634	-1.256681
H	-3.763647	-0.865827	-1.74838
C	-2.151192	-0.831338	0.258316
C	-2.266247	-2.230693	0.605427
C	-3.319878	-2.727193	1.4343
H	-4.08118	-2.052375	1.806268
C	-3.415802	-4.065984	1.768222
H	-4.234425	-4.393702	2.401757
C	-2.48681	-4.999441	1.277684
H	-2.590331	-6.053216	1.517287
C	-1.445769	-4.547722	0.45721
H	-0.710855	-5.250384	0.072977
C	-1.32861	-3.20559	0.129151
H	-0.496052	-2.900533	-0.49168
C	-1.748885	1.163325	3.137966
H	-2.647175	0.836085	3.678953
H	-1.220245	0.258574	2.819675
H	-1.113238	1.68905	3.854954
C	-3.120947	4.781259	-1.262266
H	-2.345273	4.832206	-2.034839
H	-4.046584	4.455748	-1.746802
H	-3.278131	5.799553	-0.894691
C	-1.110714	-0.343599	-0.548893

H	-0.464041	-1.01017	-1.099614
H	-1.124418	0.686898	-0.865795
C	-5.791289	0.913169	2.107136
H	-5.066907	1.722246	2.244692
H	-6.783007	1.306928	2.3493
H	-5.556528	0.140004	2.852708
C	-5.980465	-1.263894	-3.328949
H	-5.763788	-0.500749	-4.085573
H	-5.26763	-2.077064	-3.48603
H	-6.982529	-1.651861	-3.539336

Table S12 Experimental and theoretical values of emission maxima for all compounds

Compound	Wavelength of Emission (nm)	Wavelength of Emission (nm)	Wavelength of Emission (nm)
	Exp.	Calc. (Monomer)	Calc. (Dimer)
TBE	450	319.17	314.48
TBE-M	309/480 /575	314.52	330.06
TBE-2M	580	343.65	360.75

9. Crystal data and structure refinement for 3aa

Single crystals suitable for X-ray diffraction were obtained by slow evaporation of the solvent from EtOH:MeOH = 10:1 at rt. X-ray structure of product **3aa** with 50% ellipsoid probability. Crystal data have been deposited to CCDC, number 2405806.

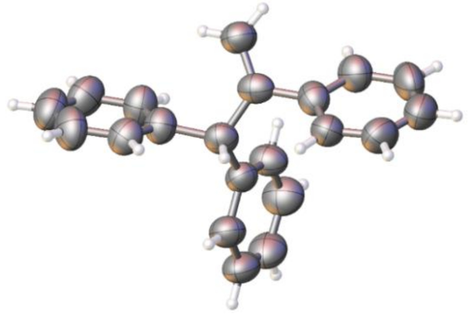

Identification code	exp_4135_auto
Empirical formula	$C_{21}H_{18}$
Formula weight	270.35
Temperature/K	292(6)
Crystal system	monoclinic
Space group	$P2_1/n$
a/Å	10.9069(8)
b/Å	6.1227(5)
c/Å	23.238(2)
α/°	90
β/°	90.408(7)
γ/°	90
Volume/$Å^3$	1551.8(2)
Z	4
$\rho_{calc}g/cm^3$	1.157
μ/mm^{-1}	0.490
F(000)	576.0
Crystal size/mm^3	0.28 × 0.22 × 0.16
Radiation	Cu Kα (λ = 1.54184)
2Θ range for data collection/°	7.608 to 134.154
Index ranges	$-13 \le h \le 13$, $-7 \le k \le 7$, $-27 \le l \le 27$
Reflections collected	37607
Independent reflections	2760 [R_{int} = 0.1266, R_{sigma} = 0.0666]
Data/restraints/parameters	2760/0/191
Goodness-of-fit on F^2	1.110
Final R indexes [I>=2σ (I)]	R_1 = 0.1047, wR_2 = 0.3266
Final R indexes [all data]	R_1 = 0.1278, wR_2 = 0.3468
Largest diff. peak/hole / e $Å^{-3}$	0.45/-0.20

Table S13. Structure of compound **3aa** determined by X-ray diffraction analysis. ORTEP diagram (thermal ellipsoids are set at a 50% probability level)

10. Crystal data and structure refinement for 3ba

Single crystals suitable for X-ray diffraction were obtained by slow evaporation of the solvent from ^{i}-BuOH:EtOH = 5:1 at rt. X-ray structure of product **3ba** with 50% ellipsoid probability. Crystal data have been deposited to CCDC, number 2405805.

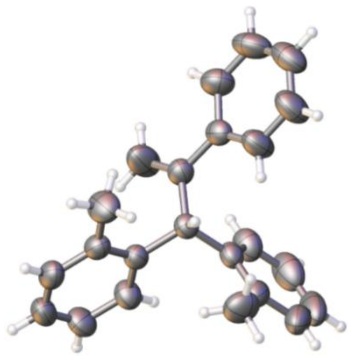

Identification code	mj24247_0m
Empirical formula	$C_{23}H_{22}$
Formula weight	298.40
Temperature/K	296
Crystal system	triclinic
Space group	P-1
a/Å	6.36940(10)
b/Å	12.2446(2)
c/Å	12.3548(2)
α/°	68.6000(10)
β/°	79.3540(10)
γ/°	84.4600(10)
Volume/Å^3	881.24(3)
Z	2
ρ_{calc}g/cm^3	1.125
μ/mm^{-1}	0.301
F(000)	320.0
Crystal size/mm^3	0.17 × 0.17 × 0.05
Radiation	GaKα (λ = 1.34139)
2Θ range for data collection/°	6.748 to 109.852
Index ranges	$-7 \leq h \leq 7$, $-14 \leq k \leq 14$, $-14 \leq l \leq 15$
Reflections collected	13896
Independent reflections	3331 [R_{int} = 0.0546, R_{sigma} = 0.0487]
Data/restraints/parameters	3331/0/210
Goodness-of-fit on F^2	1.084
Final R indexes [I>=2σ (I)]	R_1 = 0.0476, wR_2 = 0.1298
Final R indexes [all data]	R_1 = 0.0740, wR_2 = 0.1398
Largest diff. peak/hole / e Å^{-3}	0.16/-0.15

Table S14. Structure of compound **3ba** determined by X-ray diffraction analysis. ORTEP diagram (thermal ellipsoids are set at a 50% probability level)

11. Crystal data and structure refinement for 3ca

Single crystals suitable for X-ray diffraction were obtained by slow evaporation of the solvent from EtOH at rt. X-ray structure of product **3ca** with 50% ellipsoid probability. Crystal data have been deposited to CCDC, number 2405804.

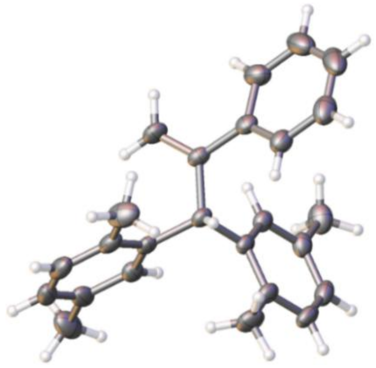

Identification code	exp_4052_auto
Empirical formula	$C_{25}H_{26}$
Formula weight	326.46
Temperature/K	173.00(10)
Crystal system	triclinic
Space group	P-1
a/Å	6.4054(2)
b/Å	14.0454(4)
c/Å	22.1215(5)
α/°	103.126(2)
β/°	96.307(2)
γ/°	97.867(2)
Volume/Å^3	1899.35(9)
Z	4
ρ_{calc}g/cm^3	1.142
μ/mm^{-1}	0.477
F(000)	704.0
Crystal size/mm^3	0.32 × 0.13 × 0.11
Radiation	Cu Kα (λ = 1.54184)
2Θ range for data collection/°	6.554 to 134.15
Index ranges	$-7 \le h \le 7$, $-16 \le k \le 16$, $-26 \le l \le 26$
Reflections collected	45202
Independent reflections	6769 [R_{int} = 0.1210, R_{sigma} = 0.0726]
Data/restraints/parameters	6769/0/460
Goodness-of-fit on F^2	1.069
Final R indexes [I>=2σ (I)]	R_1 = 0.0533, wR_2 = 0.1448
Final R indexes [all data]	R_1 = 0.0634, wR_2 = 0.1542
Largest diff. peak/hole / e Å^{-3}	0.22/-0.20

Table S15. Structure of compound **3ca** determined by X-ray diffraction analysis. ORTEP diagram (thermal ellipsoids are set at a 50% probability level)

12. Crystal data and structure refinement for 3ja

Single crystals suitable for X-ray diffraction were obtained by slow evaporation of the solvent from Hexane at rt. X-ray structure of product **3ja** with 50% ellipsoid probability. Crystal data have been deposited to CCDC, number 2473694.

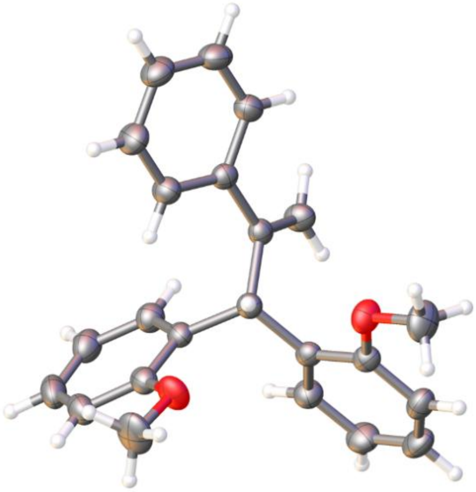

Identification code	exp_4236_auto
Empirical formula	$C_{23}H_{22}O_2$
Formula weight	330.40
Temperature/K	172(1)
Crystal system	orthorhombic
Space group	Pbca
a/Å	15.3747(2)
b/Å	12.6507(2)
c/Å	18.3582(3)
α/°	90
β/°	90
γ/°	90
Volume/Å^3	3570.68(9)
Z	8
ρ_{calc}g/cm^3	1.229
μ/mm^{-1}	0.603
F(000)	1408.0
Crystal size/mm^3	0.22 × 0.16 × 0.12
Radiation	Cu Kα (λ = 1.54184)
2Θ range for data collection/°	9.636 to 134.112
Index ranges	$-18 \le h \le 18$, $-15 \le k \le 15$, $-21 \le l \le 21$
Reflections collected	97718
Independent reflections	3191 [R_{int} = 0.0982, R_{sigma} = 0.0216]
Data/restraints/parameters	3191/0/228
Goodness-of-fit on F^2	1.046
Final R indexes [I>=2σ (I)]	R_1 = 0.0555, wR_2 = 0.1003
Final R indexes [all data]	R_1 = 0.0618, wR_2 = 0.1037
Largest diff. peak/hole / e Å^{-3}	0.12/-0.22

Table S16. Structure of compound **3ja** determined by X-ray diffraction analysis. ORTEP diagram (thermal ellipsoids are set at a 50% probability level)

13. Crystal data and structure refinement for 3bq

Single crystals suitable for X-ray diffraction were obtained by slow evaporation of the solvent from Hexane at rt. X-ray structure of product **3bq** with 50% ellipsoid probability. Crystal data have been deposited to CCDC, number 2473695.

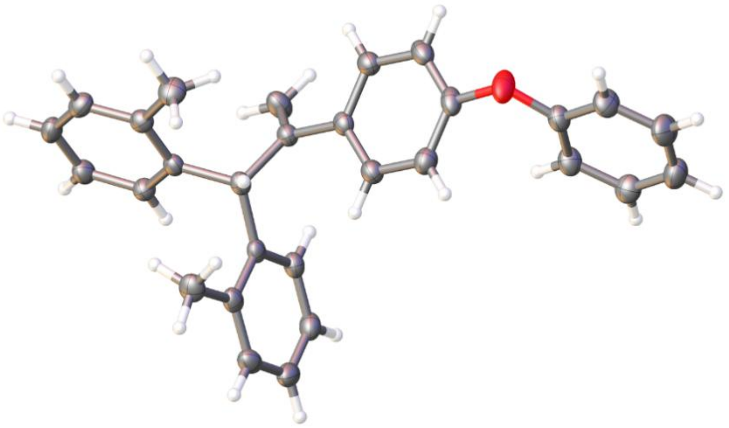

Identification code	exp_4144_auto
Empirical formula	$C_{29}H_{26}O$
Formula weight	390.50
Temperature/K	169.99(10)
Crystal system	monoclinic
Space group	$P2_1/n$
a/Å	11.43510(10)
b/Å	11.47540(10)
c/Å	16.43970(10)
α/°	90
β/°	95.9830(10)
γ/°	90
Volume/Å^3	2145.51(3)
Z	4
ρ_{calc}g/cm^3	1.209
μ/mm^{-1}	0.546
F(000)	832.0
Crystal size/mm^3	0.32 × 0.26 × 0.12
Radiation	Cu Kα (λ = 1.54184)
2Θ range for data collection/°	9.416 to 134.148
Index ranges	$-13 \le h \le 13$, $-13 \le k \le 13$, $-19 \le l \le 19$
Reflections collected	53829
Independent reflections	3832 [R_{int} = 0.0404, R_{sigma} = 0.0151]
Data/restraints/parameters	3832/0/274
Goodness-of-fit on F^2	1.030
Final R indexes [I>=2σ (I)]	R_1 = 0.0318, wR_2 = 0.0762
Final R indexes [all data]	R_1 = 0.0336, wR_2 = 0.0775
Largest diff. peak/hole / e Å^{-3}	0.21/-0.15

Table S17. Structure of compound **3bq** determined by X-ray diffraction analysis. ORTEP diagram (thermal ellipsoids are set at a 50% probability level)

14. Crystal data and structure refinement for 3bk

Single crystals suitable for X-ray diffraction were obtained by slow evaporation of the solvent from EtOH at rt. X-ray structure of product **3bk** with 50% ellipsoid probability. Crystal data have been deposited to CCDC, number 2473696.

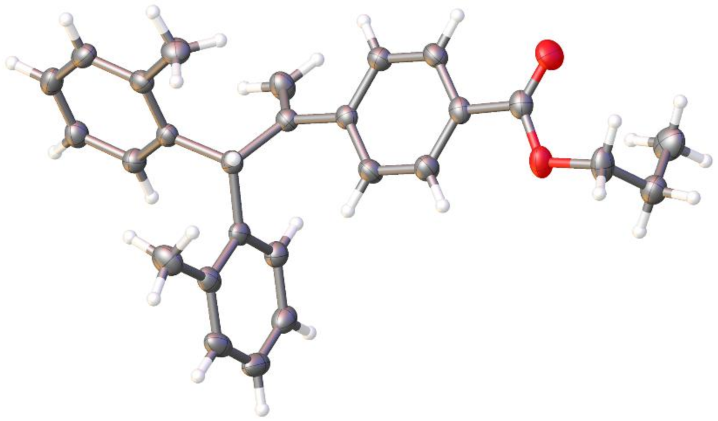

Identification code	exp_4156_auto
Empirical formula	$C_{27}H_{28}O_2$
Formula weight	384.49
Temperature/K	169.99(10)
Crystal system	monoclinic
Space group	$P2_1/n$
a/Å	11.1933(2)
b/Å	16.6053(2)
c/Å	11.5936(2)
α/°	90
β/°	95.3090(10)
γ/°	90
Volume/Å^3	2145.64(6)
Z	4
ρ_{calc}g/cm^3	1.190
μ/mm^{-1}	0.569
F(000)	824.0
Crystal size/mm^3	0.26 × 0.22 × 0.16
Radiation	Cu Kα (λ = 1.54184)
2Θ range for data collection/°	9.558 to 134.144
Index ranges	$-13 \leq h \leq 13$, $-19 \leq k \leq 19$, $-13 \leq l \leq 13$
Reflections collected	57036
Independent reflections	3813 [R_{int} = 0.0672, R_{sigma} = 0.0256]
Data/restraints/parameters	3813/0/265
Goodness-of-fit on F^2	1.058
Final R indexes [I>=2σ (I)]	R_1 = 0.0354, wR_2 = 0.0912
Final R indexes [all data]	R_1 = 0.0402, wR_2 = 0.0947
Largest diff. peak/hole / e Å^{-3}	0.16/-0.17

Table S18. Structure of compound **3bk** determined by X-ray diffraction analysis. ORTEP diagram (thermal ellipsoids are set at a 50% probability level)

15.References

1. J. Liu, Z. Han, X. Wang, Z. Wang, K. Ding, *J. Am. Chem. Soc.* **2015** *137*, 15346-15349.

2. Cui, P., Wang, Q., McCollom, S. P., Manor, B. C., Carroll, P. J., Tomson, N. C. *Angew. Chem., Int. Ed.* **2017** *56*, 15979-15983.

3. Chen, K., Zhu, H., Li, S., Bai, J., Guo, Y., Ding, K., Peng, Q., Wang, X., *J. Am. Chem. Soc.* **2023**, *145*, 24877-24888.

4. Thierer, L. M., Brooks, S. H., Weberg, A. B., Cui, P., Zhang, S.; Gau, M. R.; Manor, B. C., Carroll, P. J., Tomson, N. C. *Inorg. Chem.* **2022** *61*, 6263-6280.

5. Spackman, M. A.; Jayatilaka, D. *Hirshfeld Surface Analysis. CrystEngComm*, **2009**, *11*, 19-32

6. Lu T.; Chen F. Multiwfn: A Multifunctional Wavefunction Analyzer. *J. Comput. Chem.* **2012**, *33*, 580–592.

7. M. J. Frisch, G. W. Trucks, H. B. Schlegel, G. E. Scuseria, M. A. Robb, J. R. Cheeseman, G. Scalmani, V. Barone, G. A. Petersson, H. Nakatsuji, X. Li, M. Caricato, A. V. Marenich, J. Bloino, B. G. Janesko, R. Gomperts, B. Mennucci, H. P. Hratchian, J. V. Ortiz, A. F. Izmaylov, J. L. Sonnenberg, D. Williams-Young, F. Ding, F. Lipparini, F. Egidi, J. Goings, B. Peng, A. Petrone, T. Henderson, D. Ranasinghe, V. G. Zakrzewski, J. Gao, N. Rega, G. Zheng, W. Liang, M. Hada, M. Ehara, K. Toyota, R. Fukuda,J. Hasegawa, M. Ishida, T. Nakajima, Y. Honda, O. Kitao, H. Nakai, T. Vreven, K. Throssell, J. A. Montgomery, Jr., J. E. Peralta, F. Ogliaro, M. J. Bearpark, J. J. Heyd, E. N. Brothers, K. N. Kudin, V. N. Staroverov, T. A. Keith, R. Kobayashi, J. Normand, K. Raghavachari, A. P. Rendell, J. C. Burant, S. S. Iyengar, J. Tomasi, M. Cossi, J. M. Millam, M. Klene, C. Adamo, R. Cammi, J. W. Ochterski, R. L. Martin, K. Morokuma, O. Farkas, J. B. Foresman, and D. J. Fox, Gaussian 16, Gaussian, Inc., Wallingford CT, 2016.

16. NMR spectra of the allenes

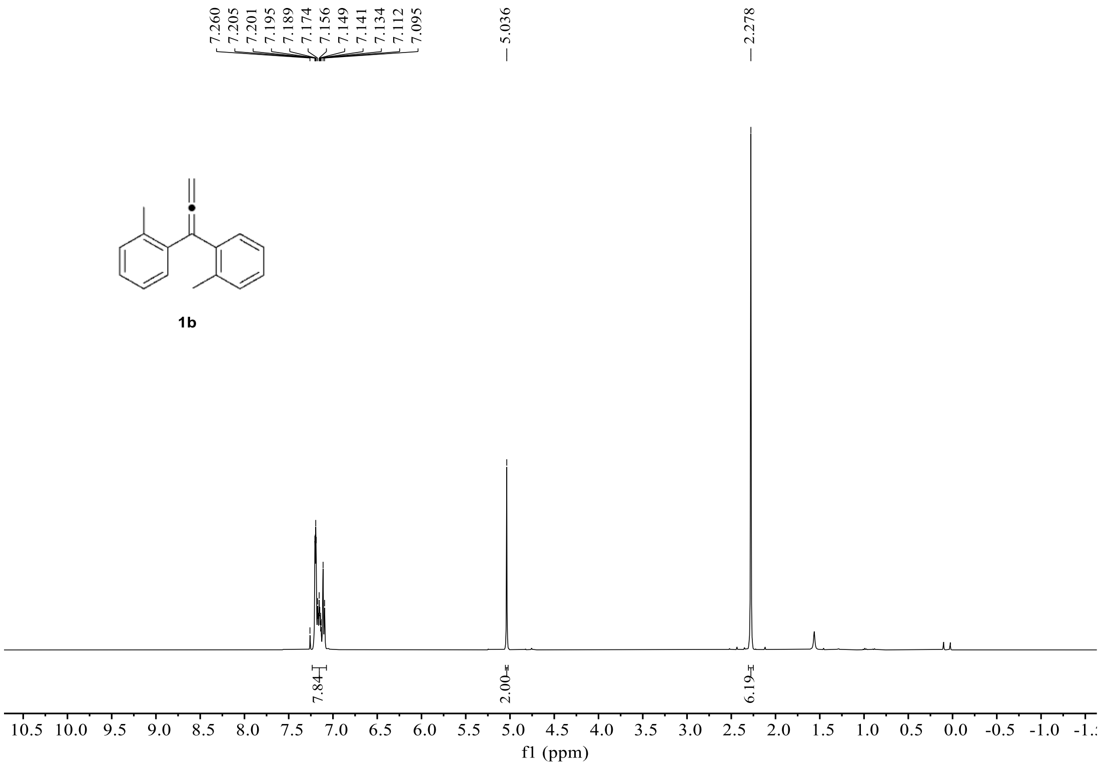

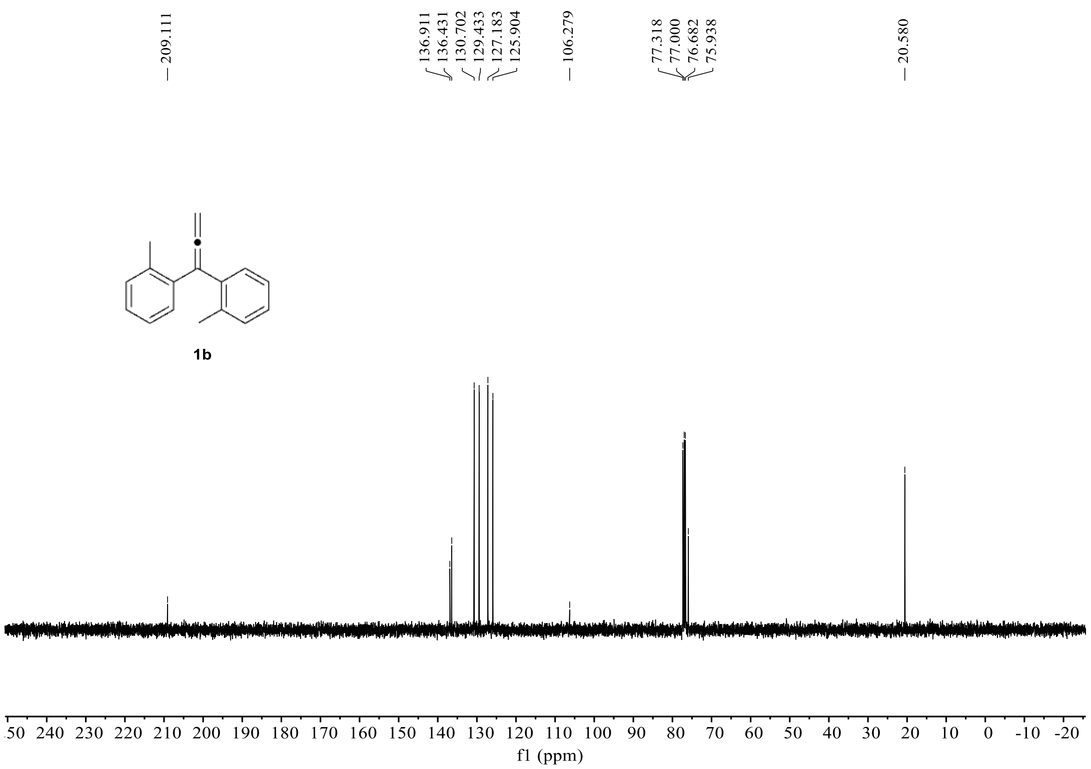

Figure S32. ^{1}H NMR ($CDCl_3$, 400 MHz) and ^{13}C NMR ($CDCl_3$, 100 MHz) spectra of compound **1b**

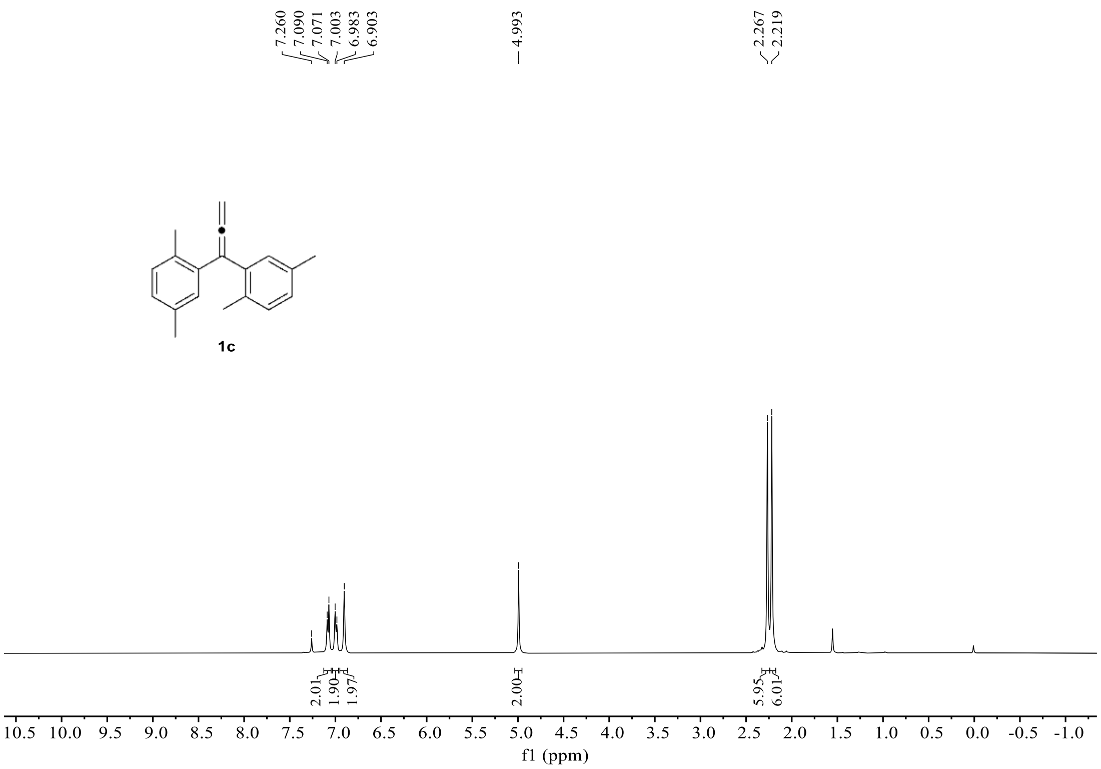

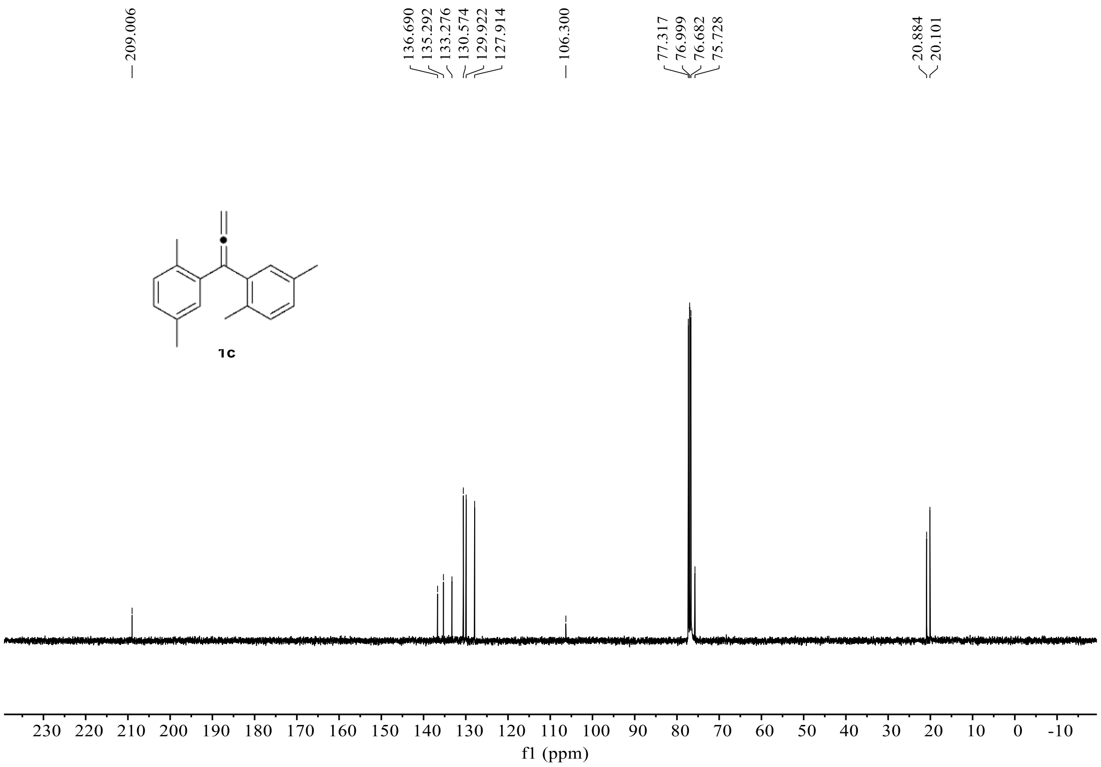

Figure S33. ^{1}H NMR ($CDCl_3$, 400 MHz) and ^{13}C NMR ($CDCl_3$, 100 MHz) spectra of compound **1c**

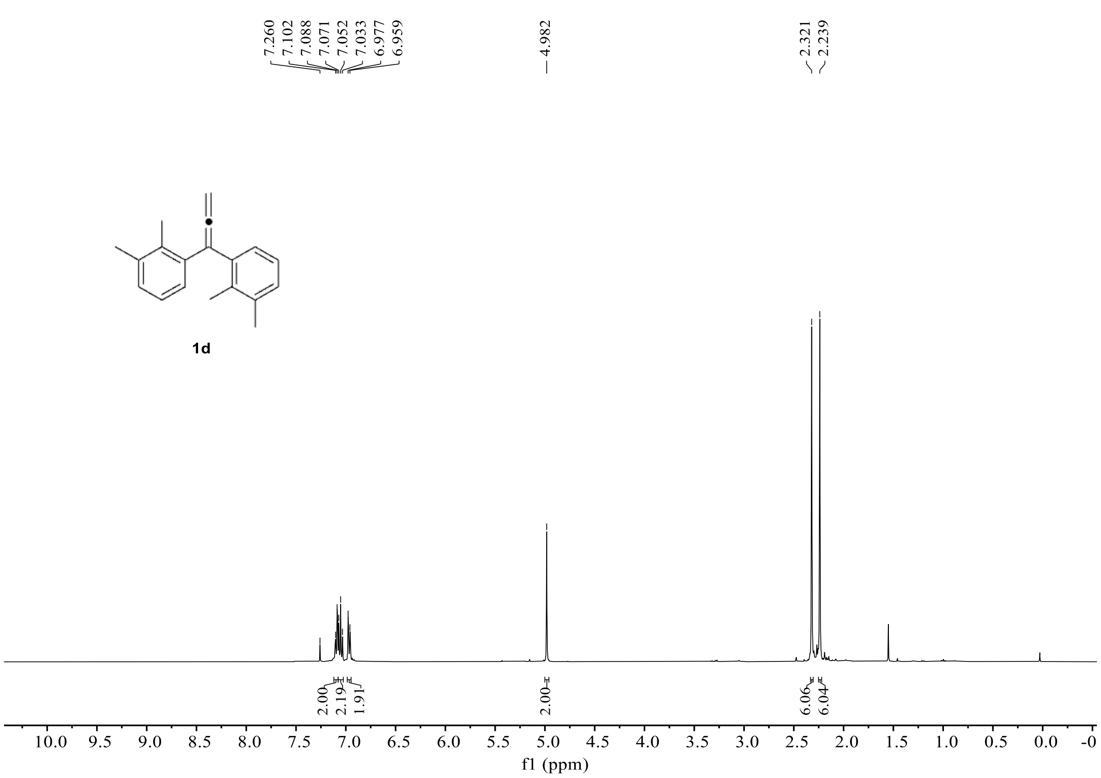

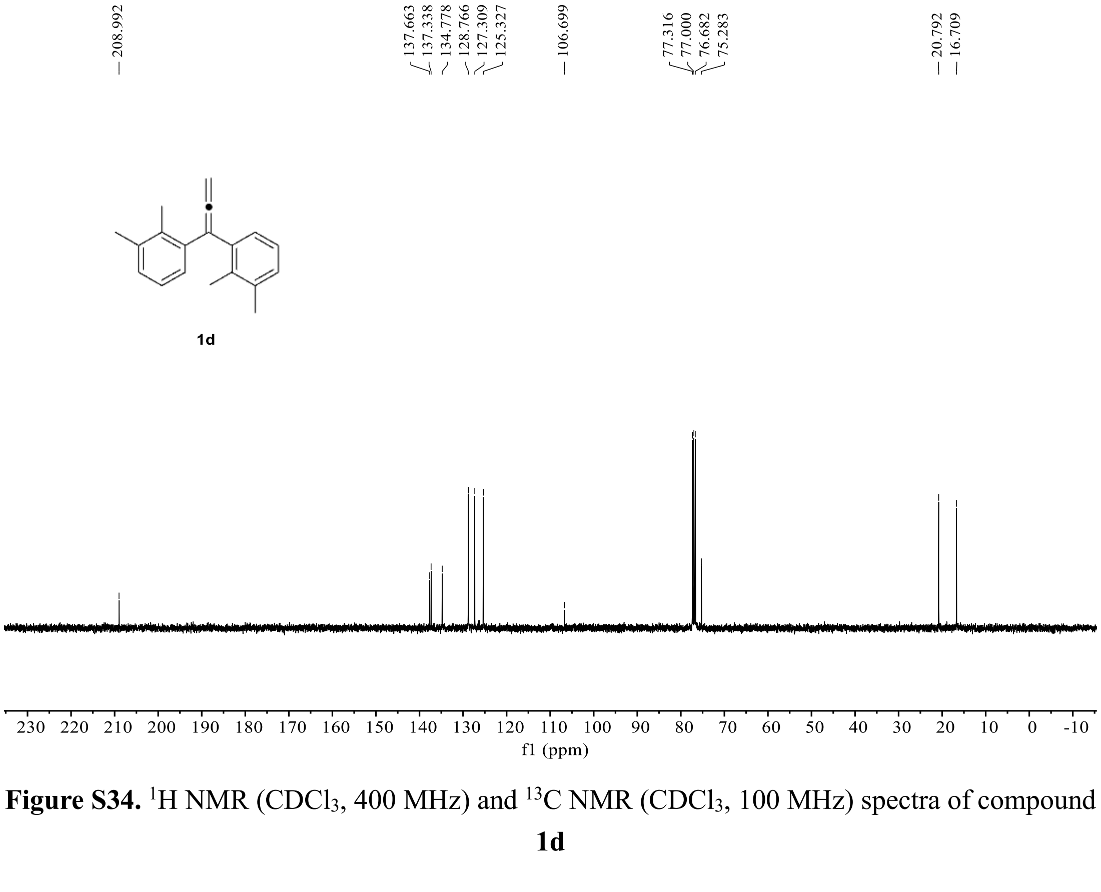

Figure S34. ^{1}H NMR ($CDCl_3$, 400 MHz) and ^{13}C NMR ($CDCl_3$, 100 MHz) spectra of compound **1d**

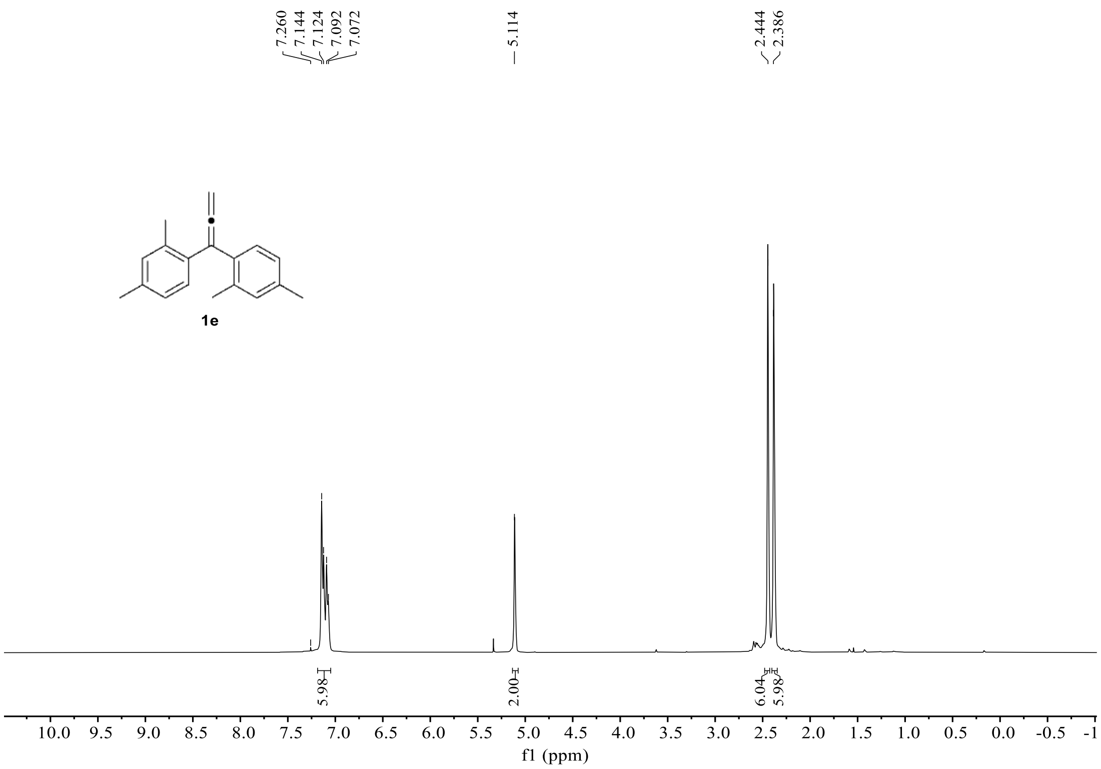

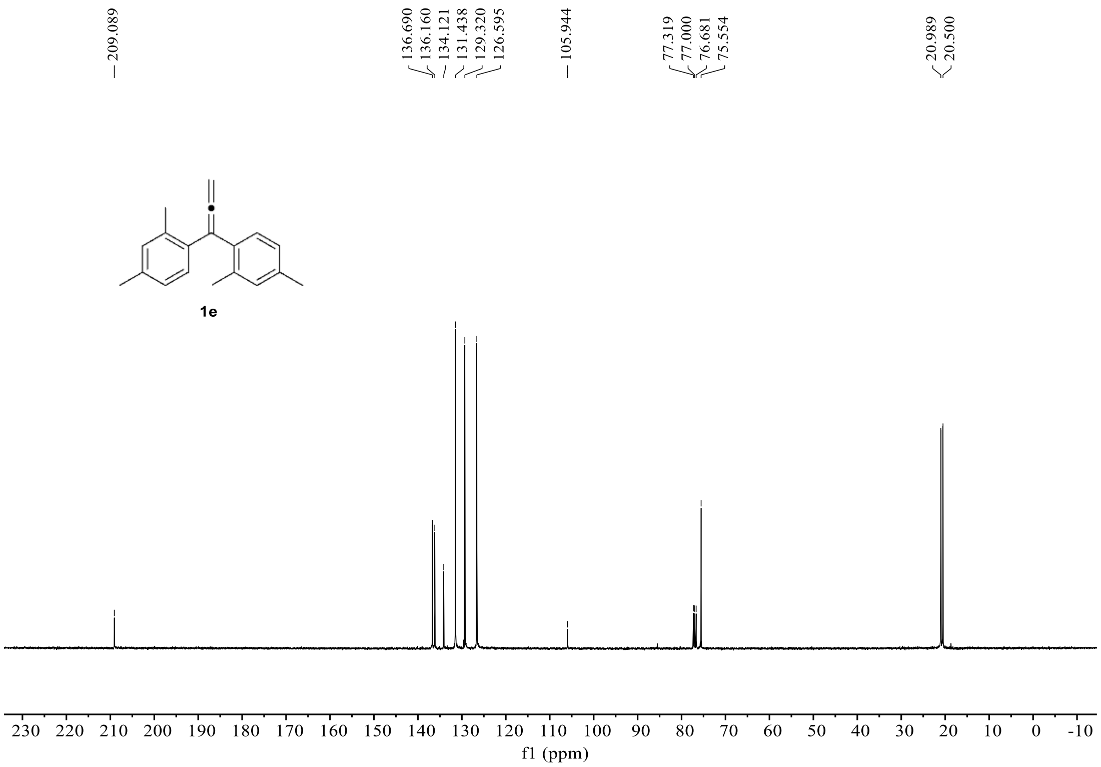

Figure S35. ^{1}H NMR ($CDCl_3$, 400 MHz) and ^{13}C NMR ($CDCl_3$, 100 MHz) spectra of compound **1e**

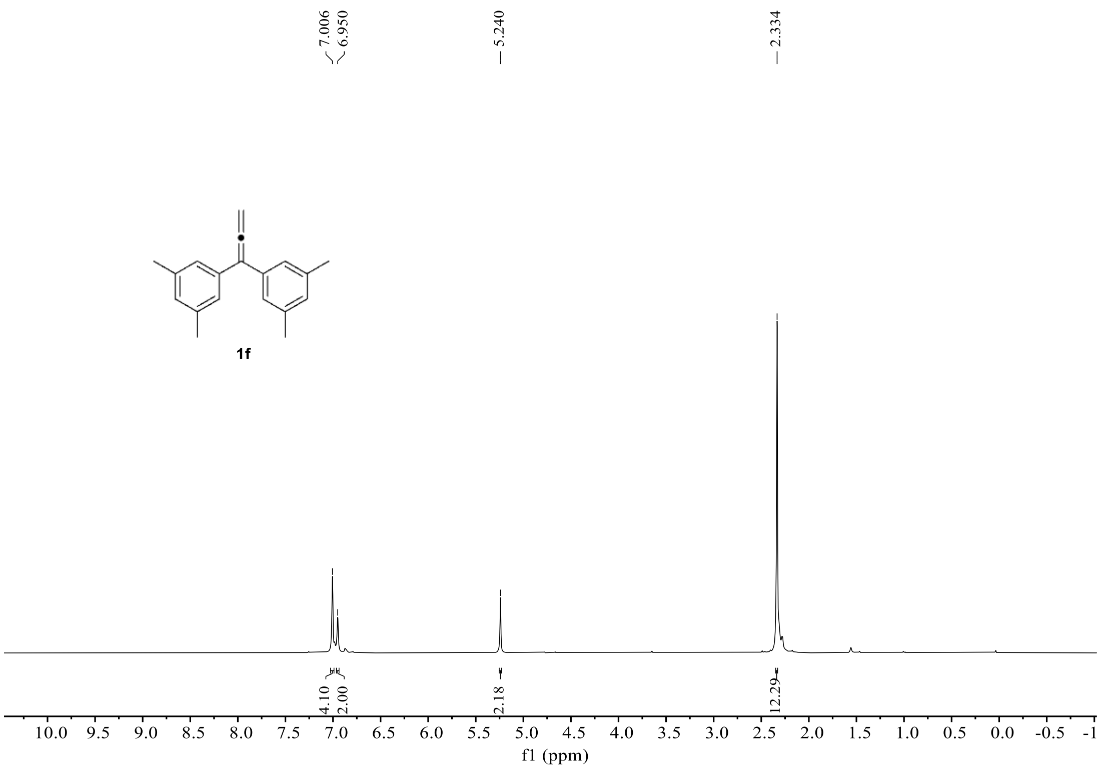

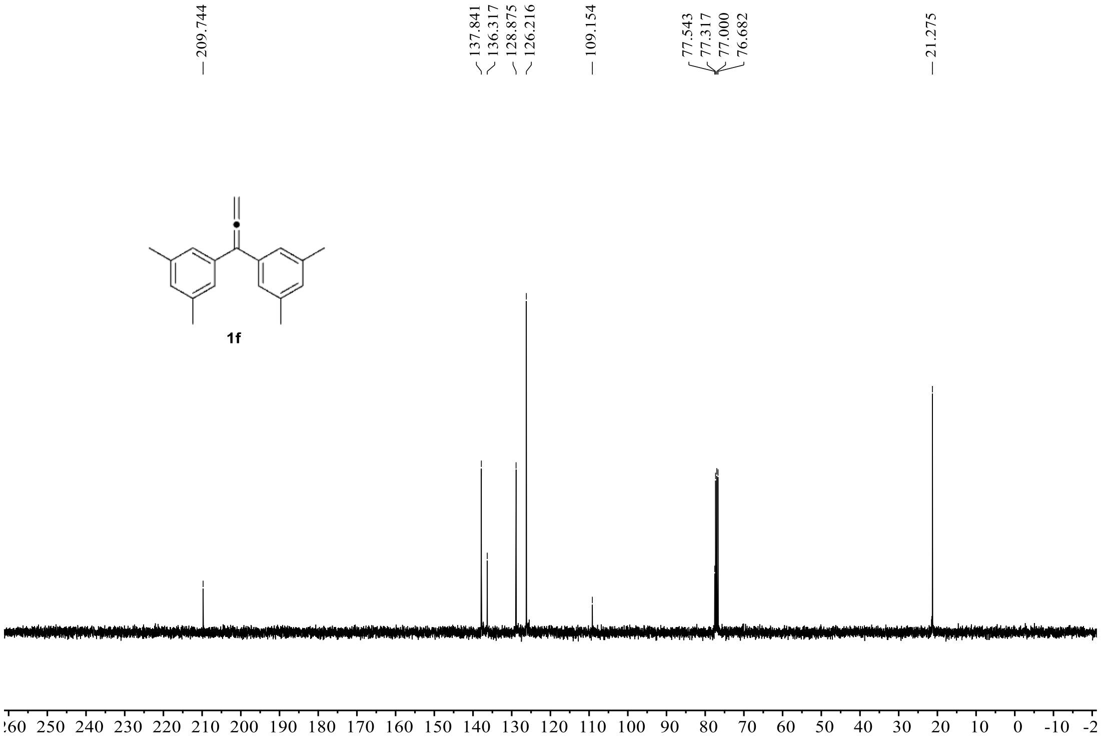

Figure S36. ^{1}H NMR ($CDCl_3$, 400 MHz) and ^{13}C NMR ($CDCl_3$, 100 MHz) spectra of compound **1f**

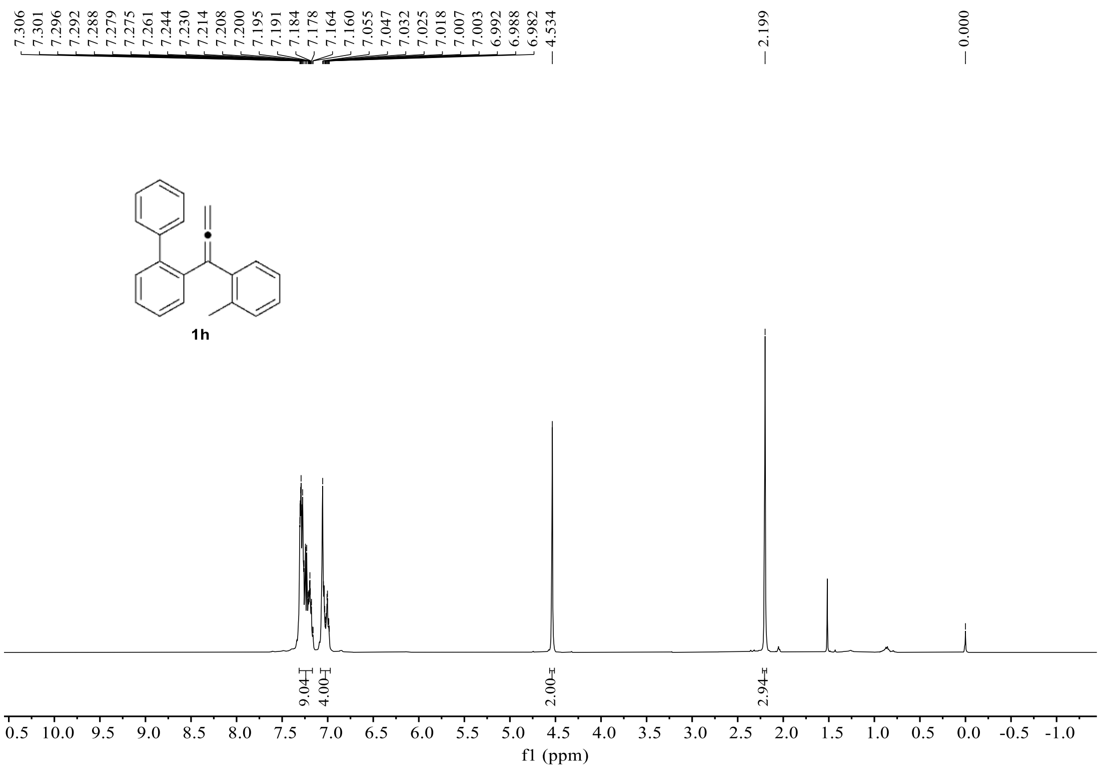

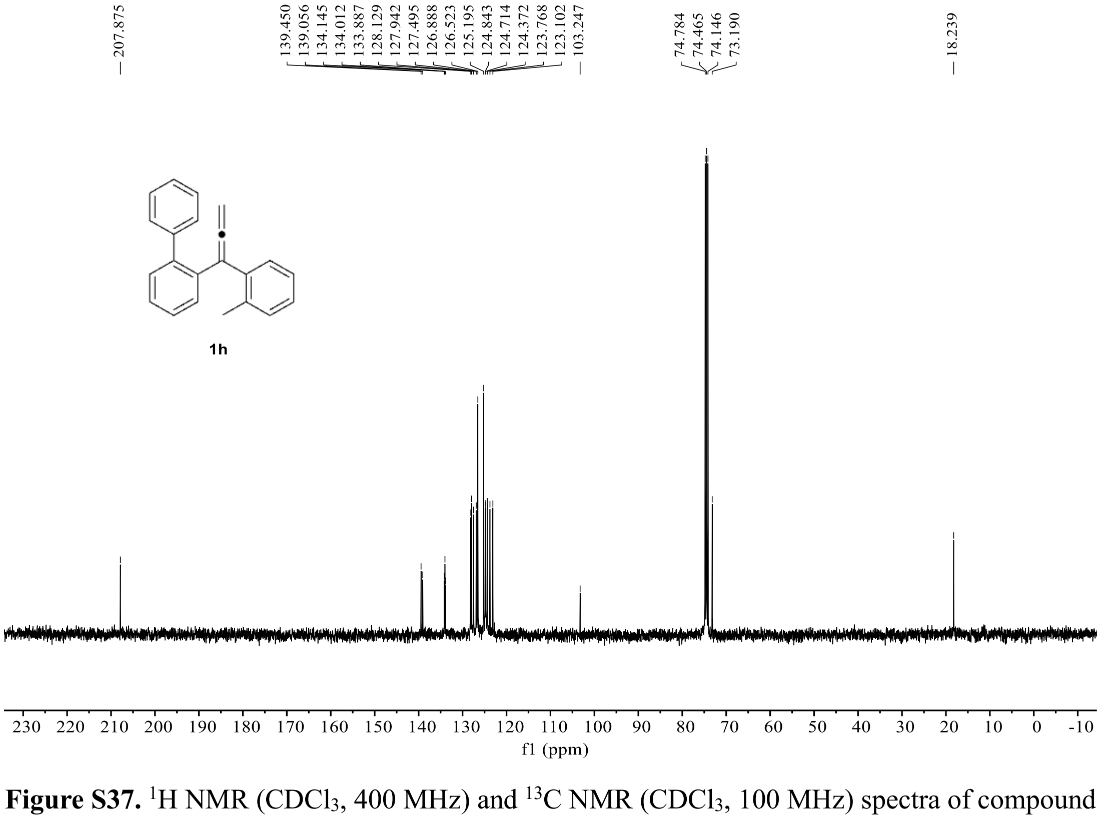

Figure S37. ^{1}H NMR ($CDCl_3$, 400 MHz) and ^{13}C NMR ($CDCl_3$, 100 MHz) spectra of compound **1h**

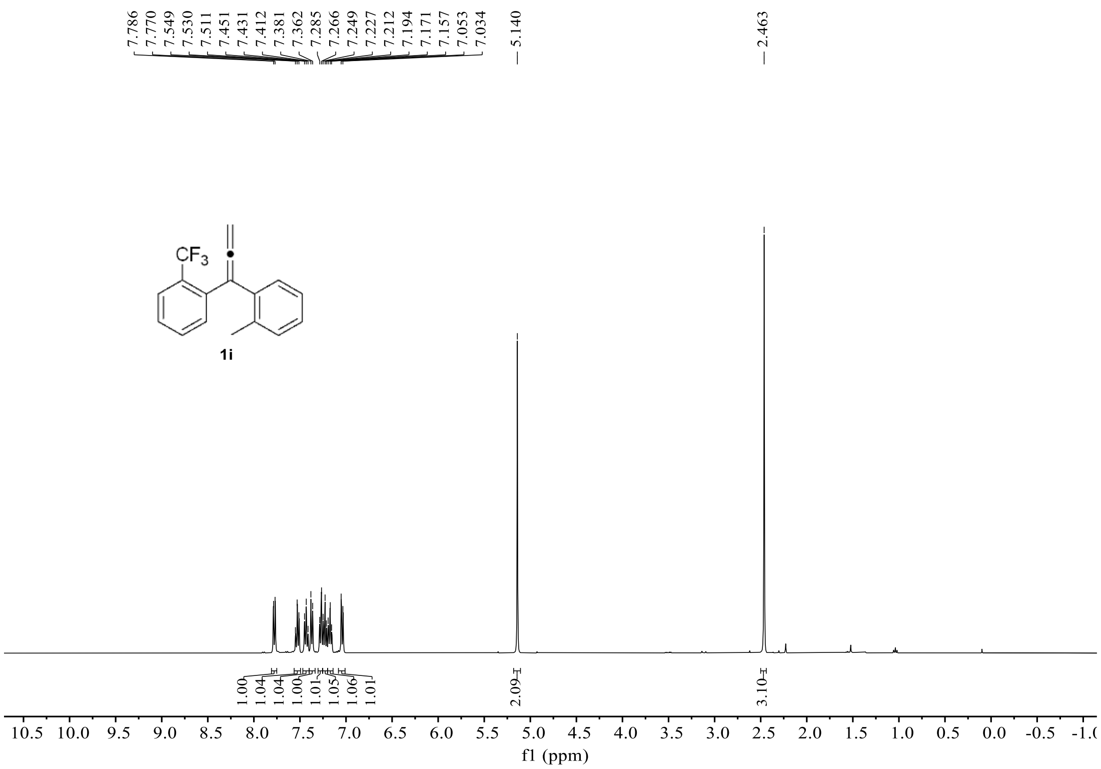
7.786
7.770
7.549
7.530
7.511
7.451
7.431
7.412
7.381
7.362
7.285
7.266
7.249
7.227
7.212
7.194
7.171
7.157
7.053
7.034
5.140
2.463
CF3
1i
1.00
1.04
1.04
1.00
1.01
1.05
1.06
1.01
2.09
3.10
10.5 10.0 9.5 9.0 8.5 8.0 7.5 7.0 6.5 6.0 5.5 5.0 4.5 4.0 3.5 3.0 2.5 2.0 1.5 1.0 0.5 0.0 -0.5
f1 (ppm)

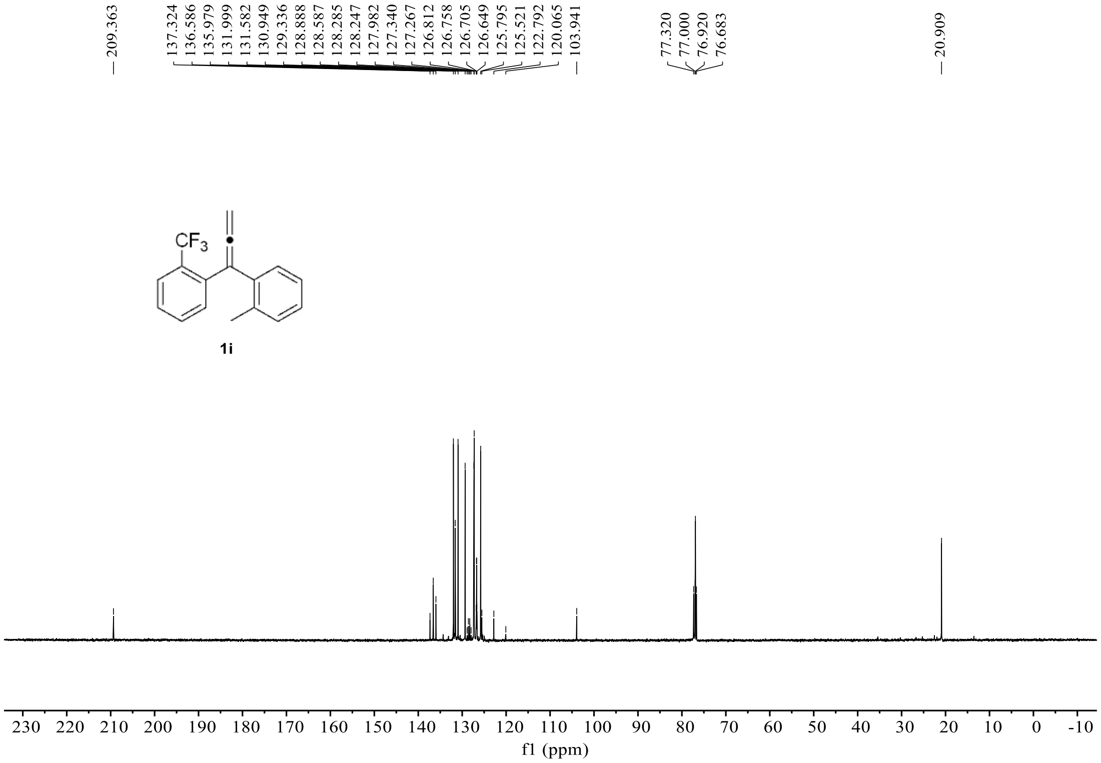
209.363
137.324
136.586
135.979
131.999
131.582
130.949
129.336
128.888
128.587
128.285
128.247
127.982
127.340
127.267
126.812
126.758
126.705
126.649
125.795
125.521
122.792
120.065
103.941
77.320
77.000
76.920
76.683
20.909
CF3
1i
230 220 210 200 190 180 170 160 150 140 130 120 110 100 90 80 70 60 50 40 30 20 10 0 -10
f1 (ppm)

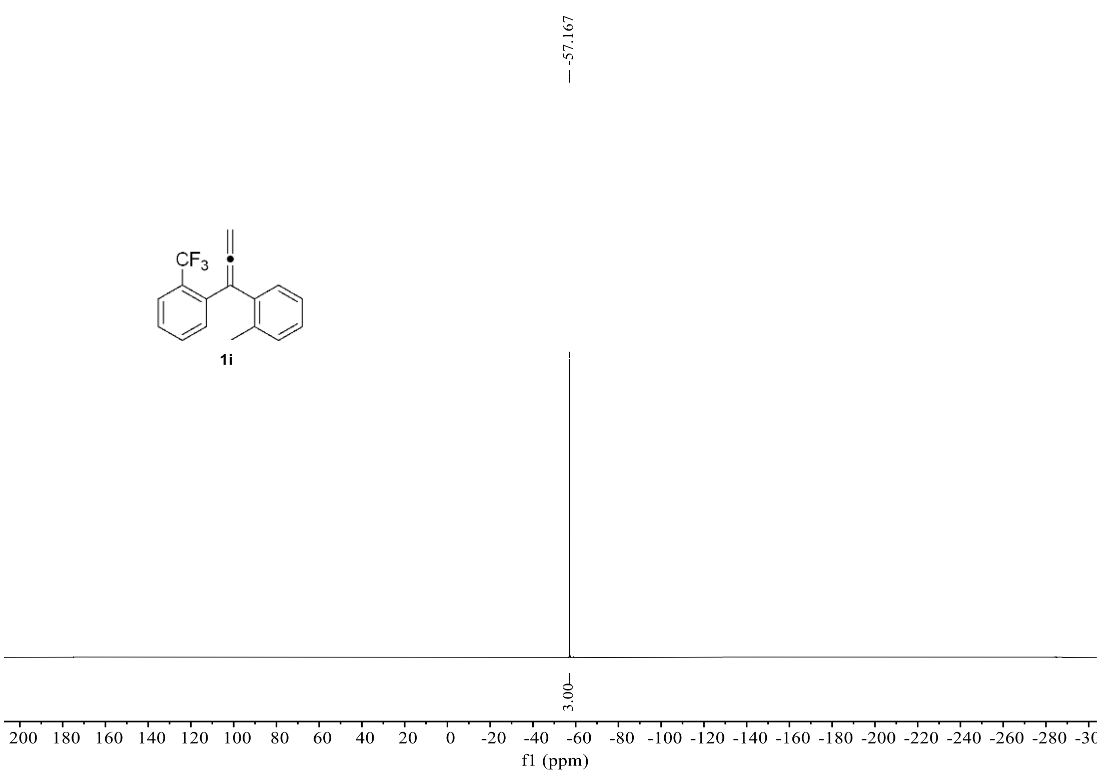

Figure S38. ^{1}H NMR ($CDCl_3$, 400 MHz), ^{13}C NMR ($CDCl_3$, 100 MHz), ^{19}F NMR ($CDCl_3$, 376 MHz) spectra of compound **1i**

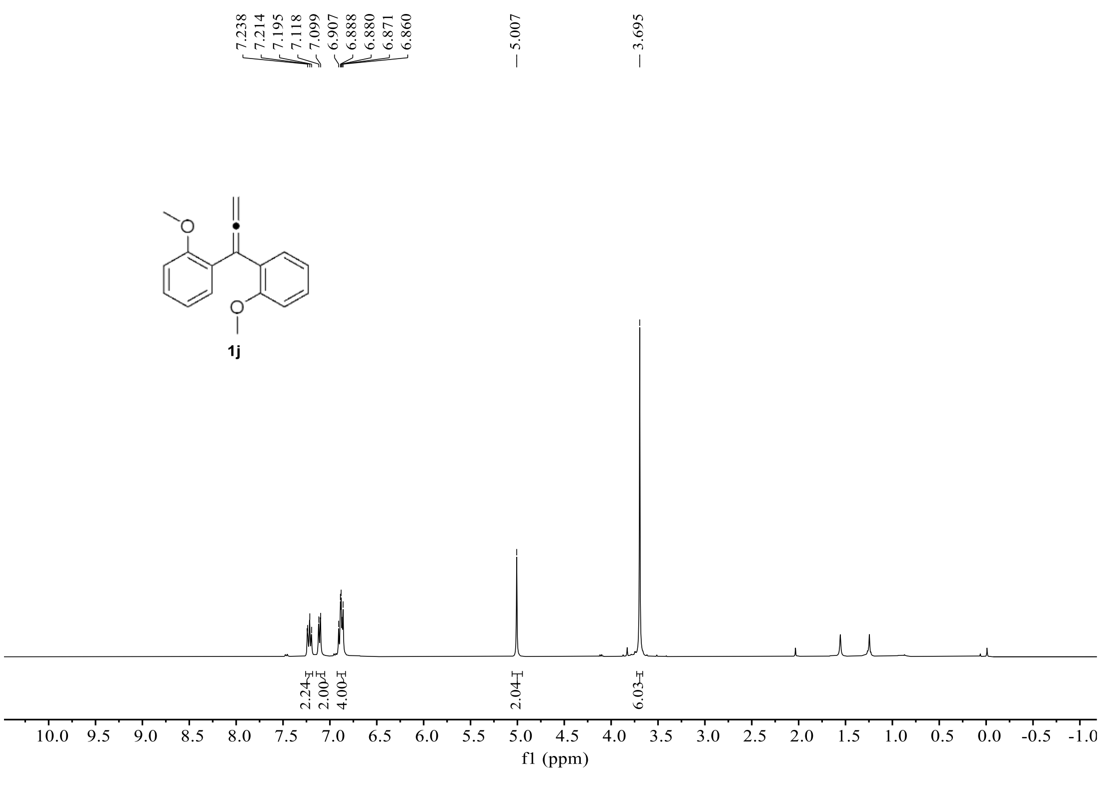

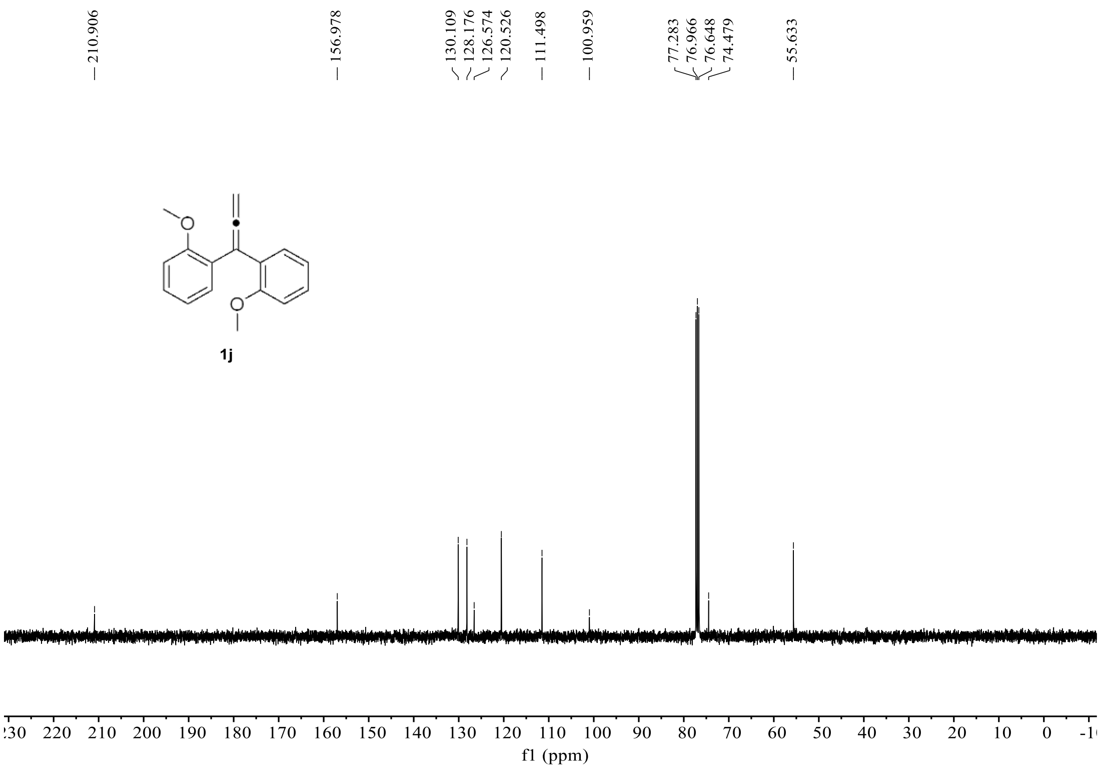

Figure S39. ^{1}H NMR ($CDCl_3$, 400 MHz) and ^{13}C NMR ($CDCl_3$, 100 MHz) spectra of compound **1j**

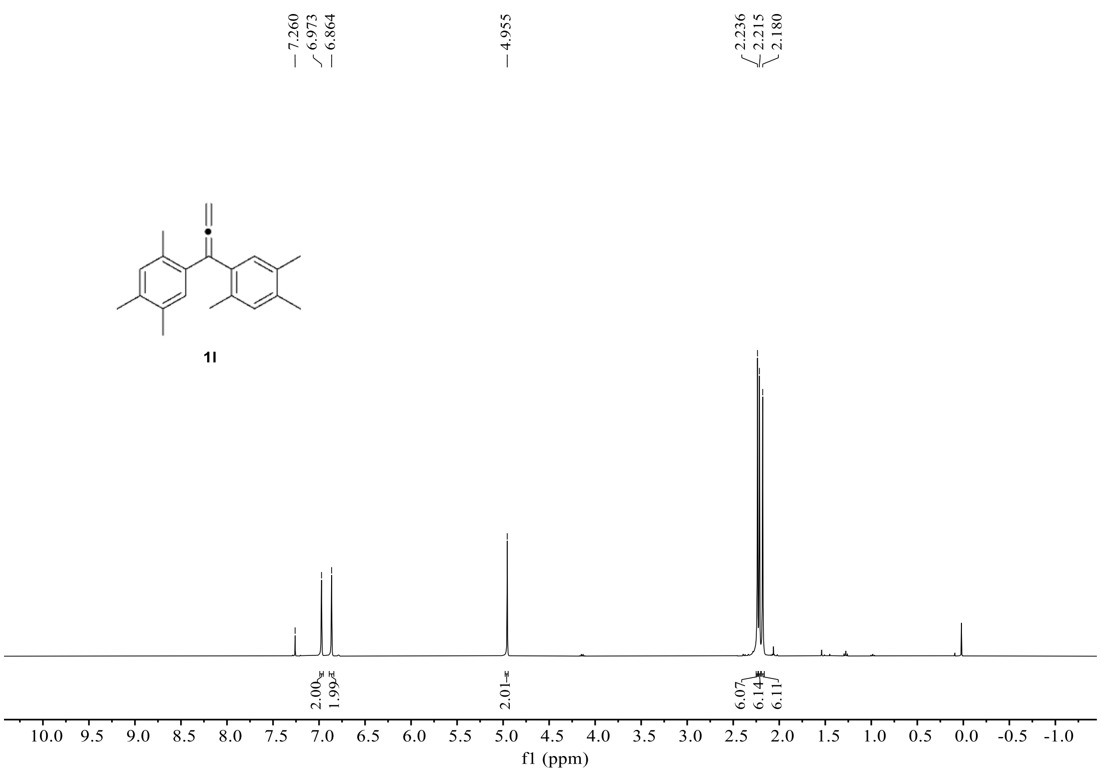

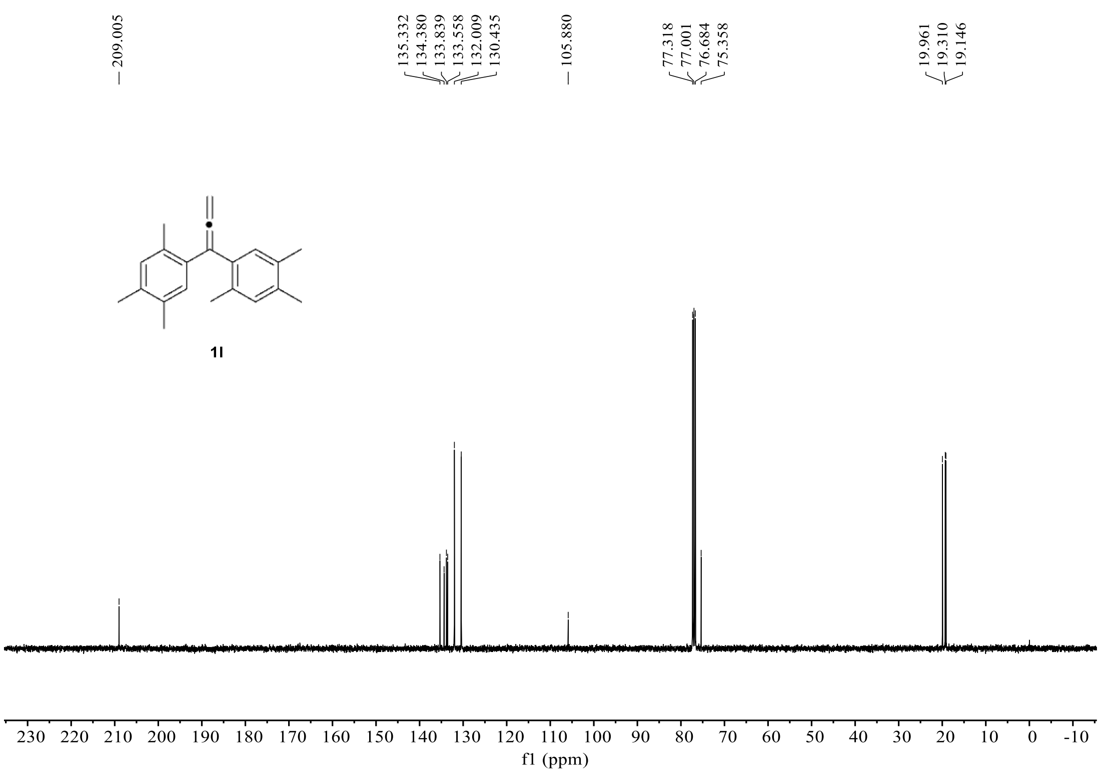

Figure S40. ^{1}H NMR ($CDCl_3$, 400 MHz) and ^{13}C NMR ($CDCl_3$, 100 MHz) spectra of compound **1l**

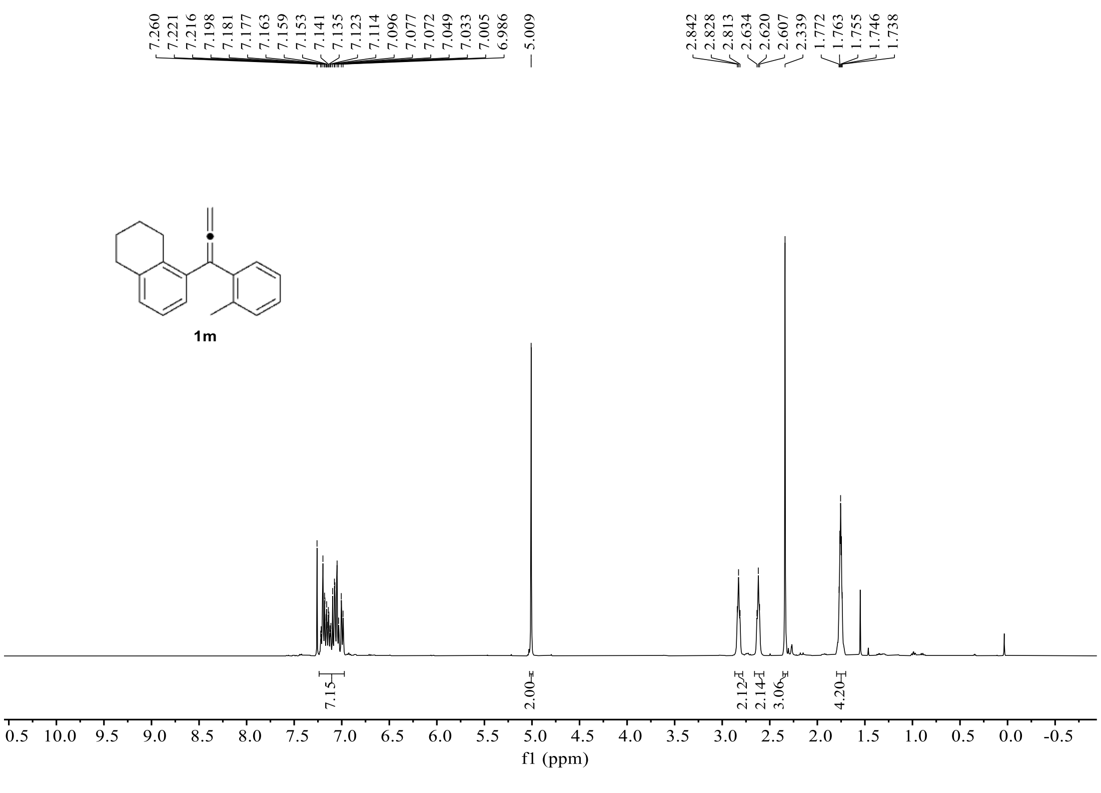

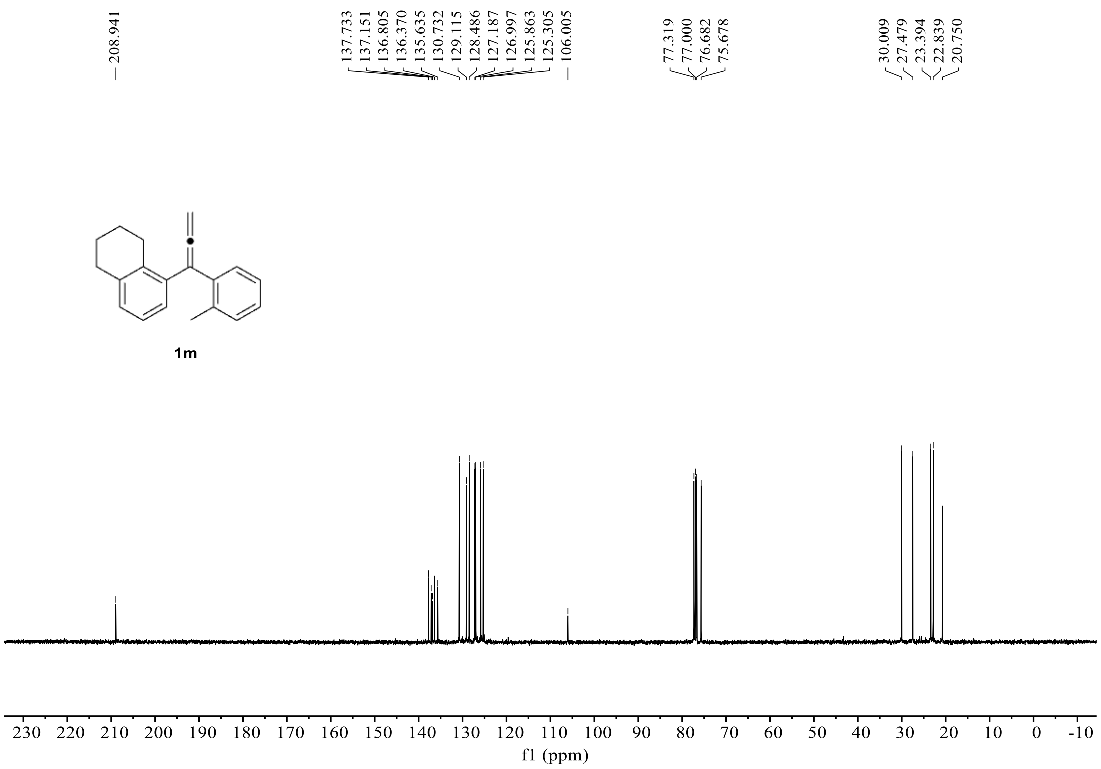

Figure S41. ^{1}H NMR ($CDCl_3$, 400 MHz) and ^{13}C NMR ($CDCl_3$, 100 MHz) spectra of compound **1m**

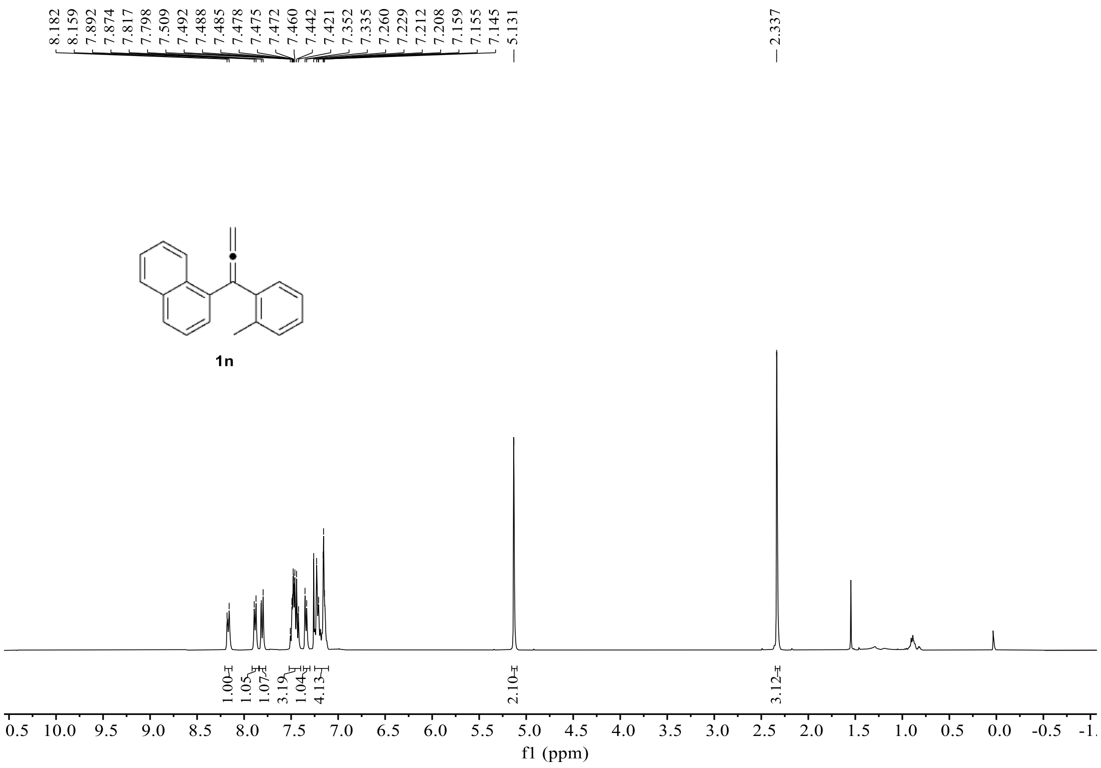

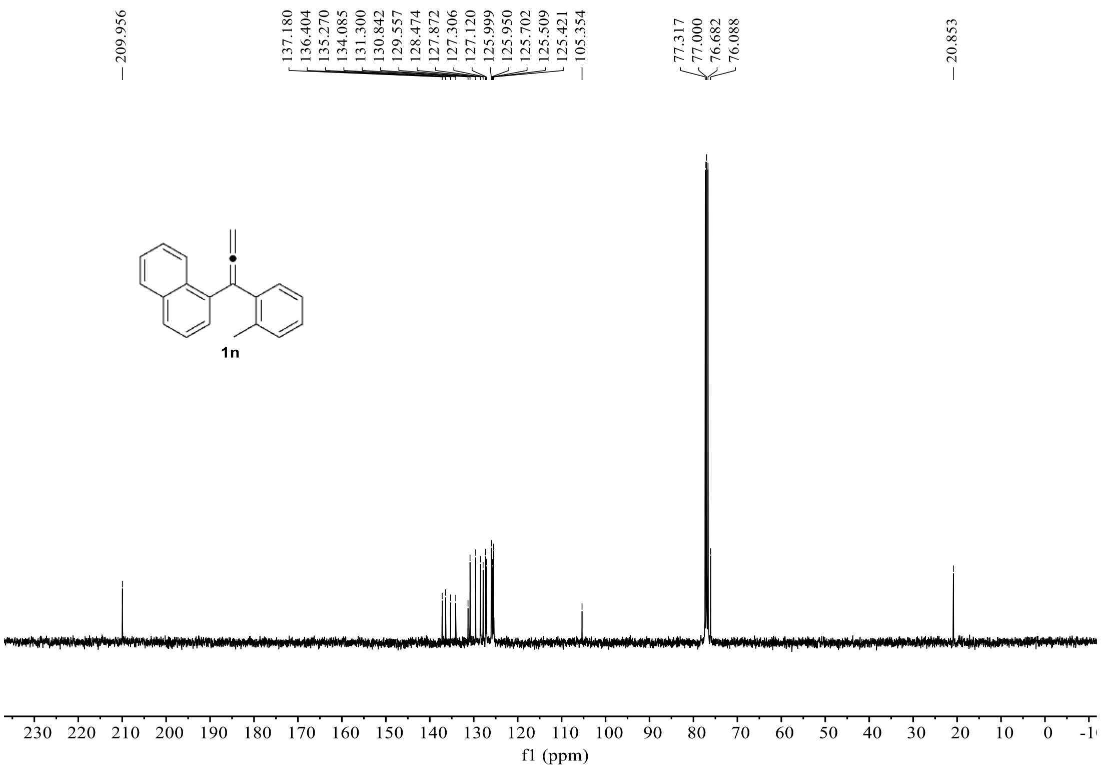

Figure S42. ^{1}H NMR ($CDCl_3$, 400 MHz) and ^{13}C NMR ($CDCl_3$, 100 MHz) spectra of compound **1n**

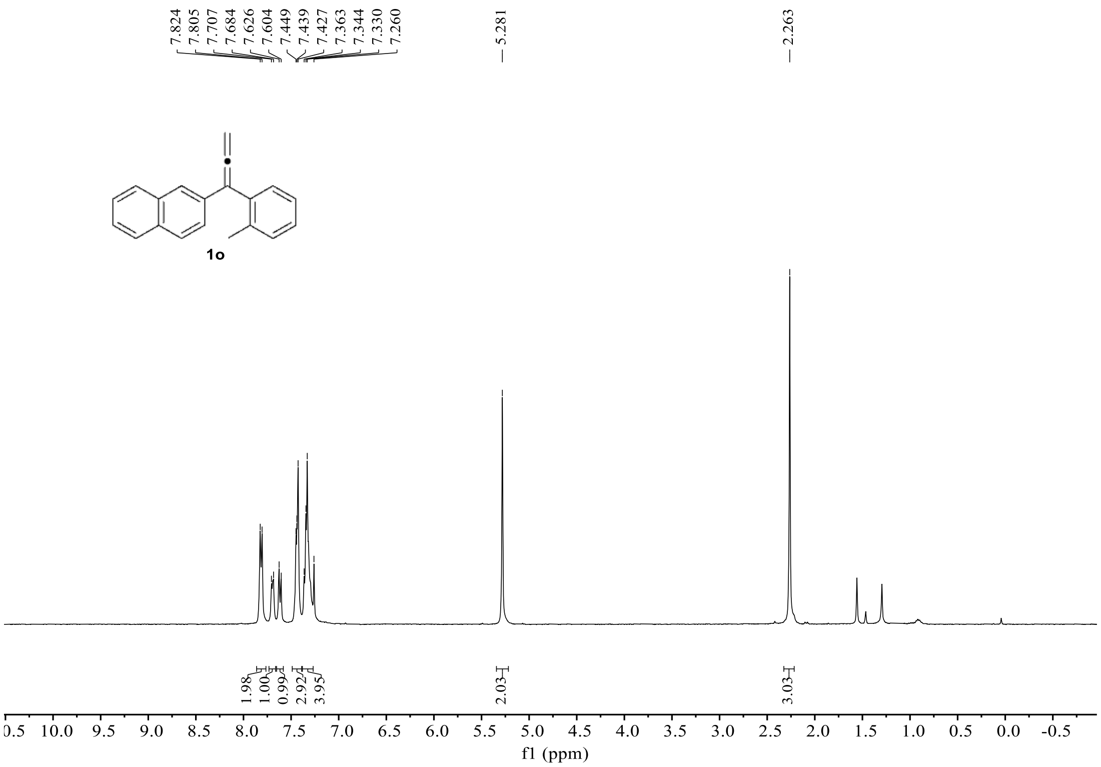

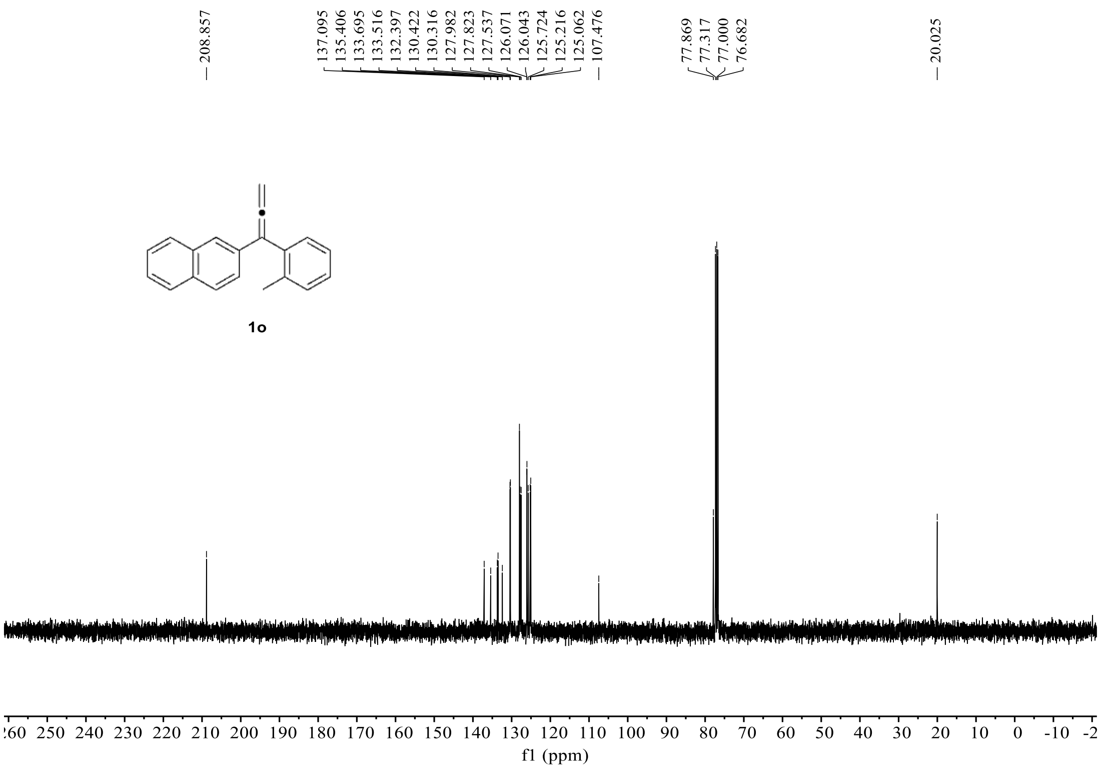

Figure S43. ^{1}H NMR ($CDCl_3$, 400 MHz) and ^{13}C NMR ($CDCl_3$, 100 MHz) spectra of compound **1o**

17. NMR spectra of the 1,2-hydroarylation products

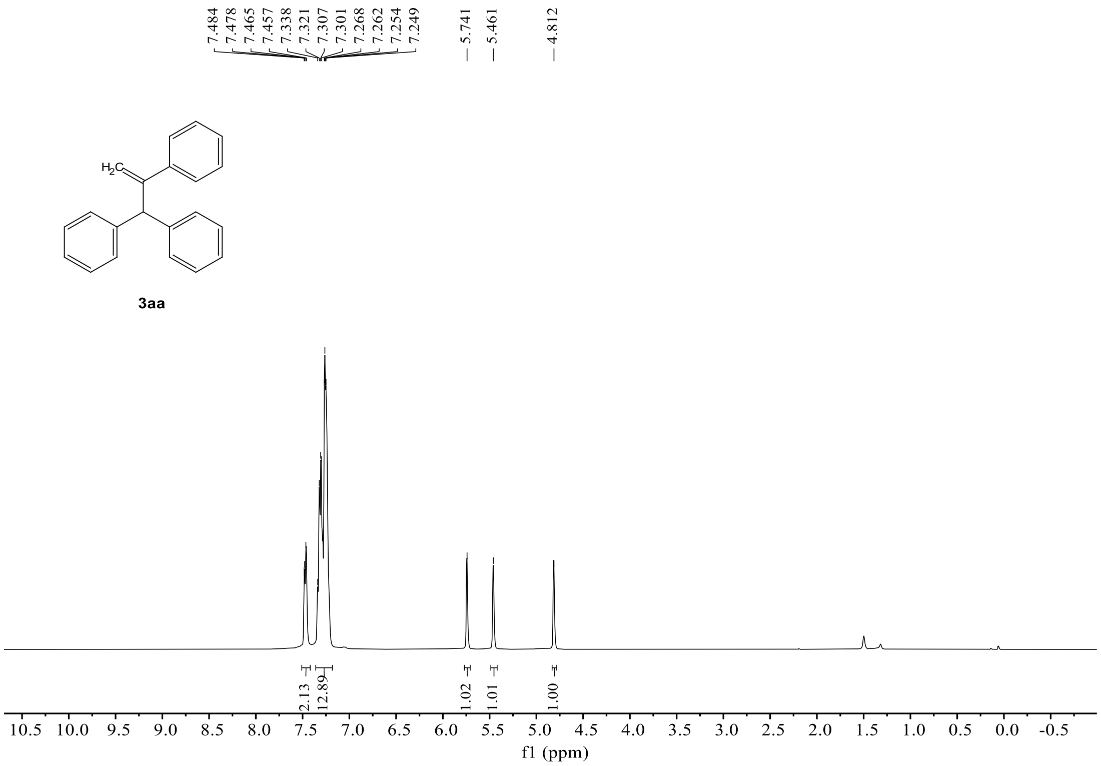

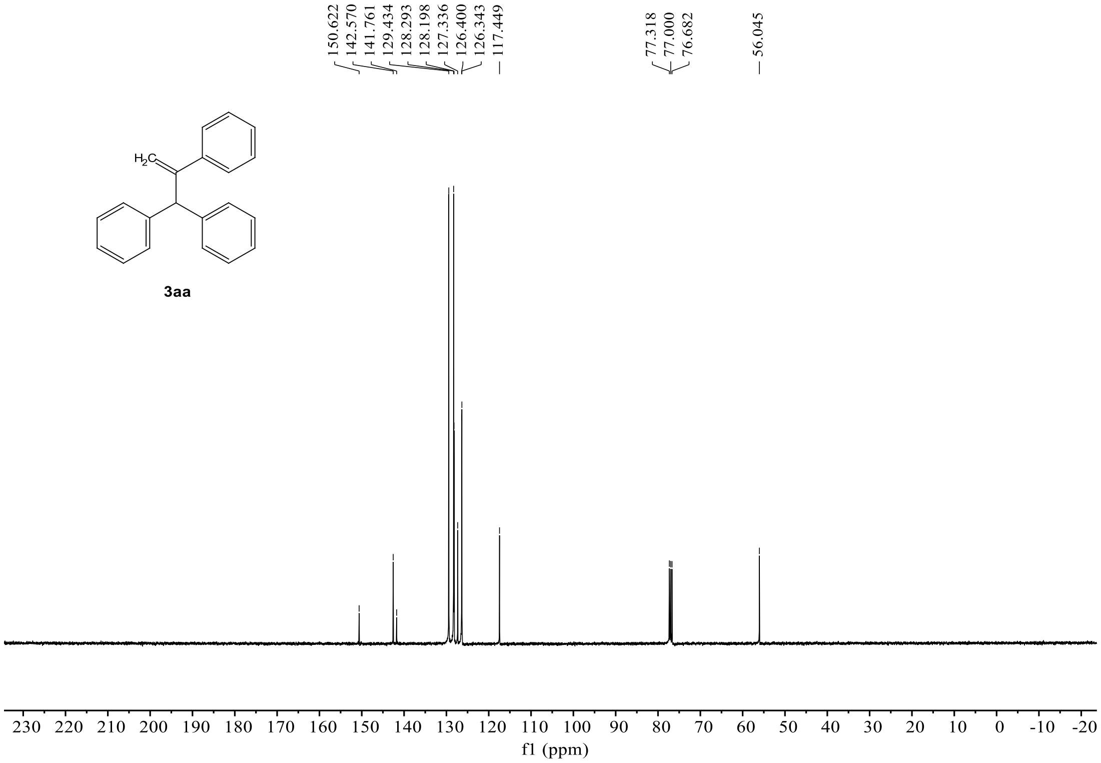

Figure S44. ^{1}H NMR ($CDCl_3$, 400 MHz) and ^{13}C NMR ($CDCl_3$, 100 MHz) spectra of compound **3aa**

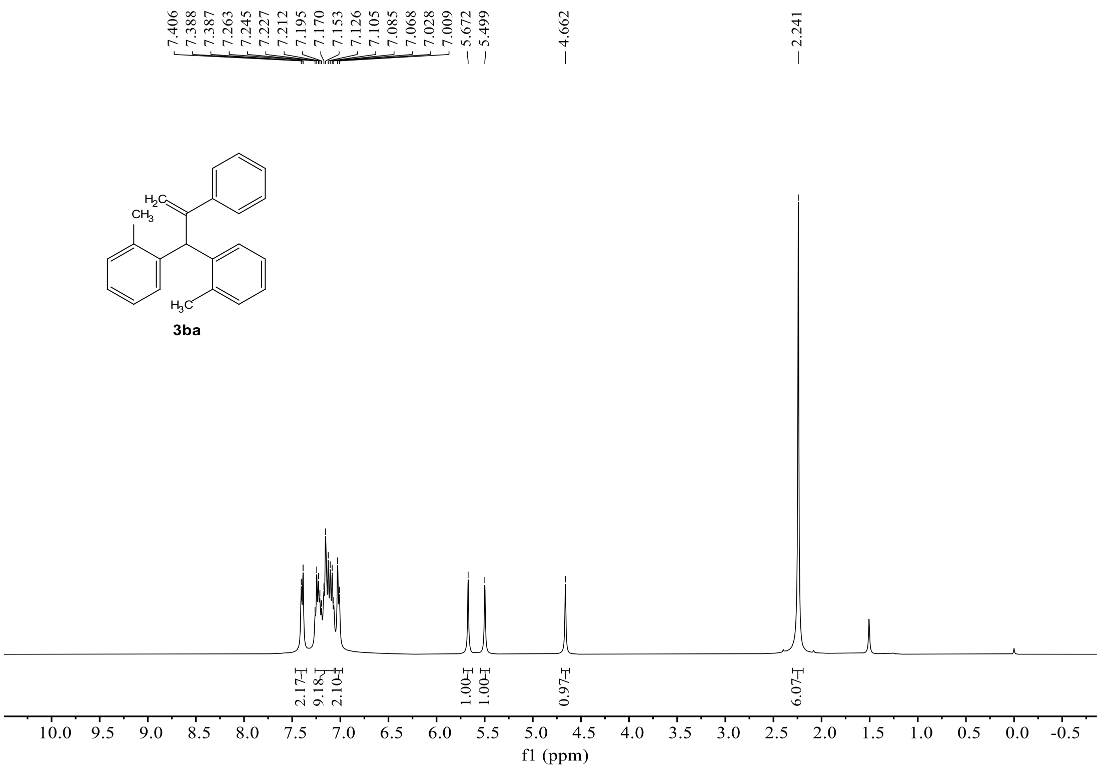

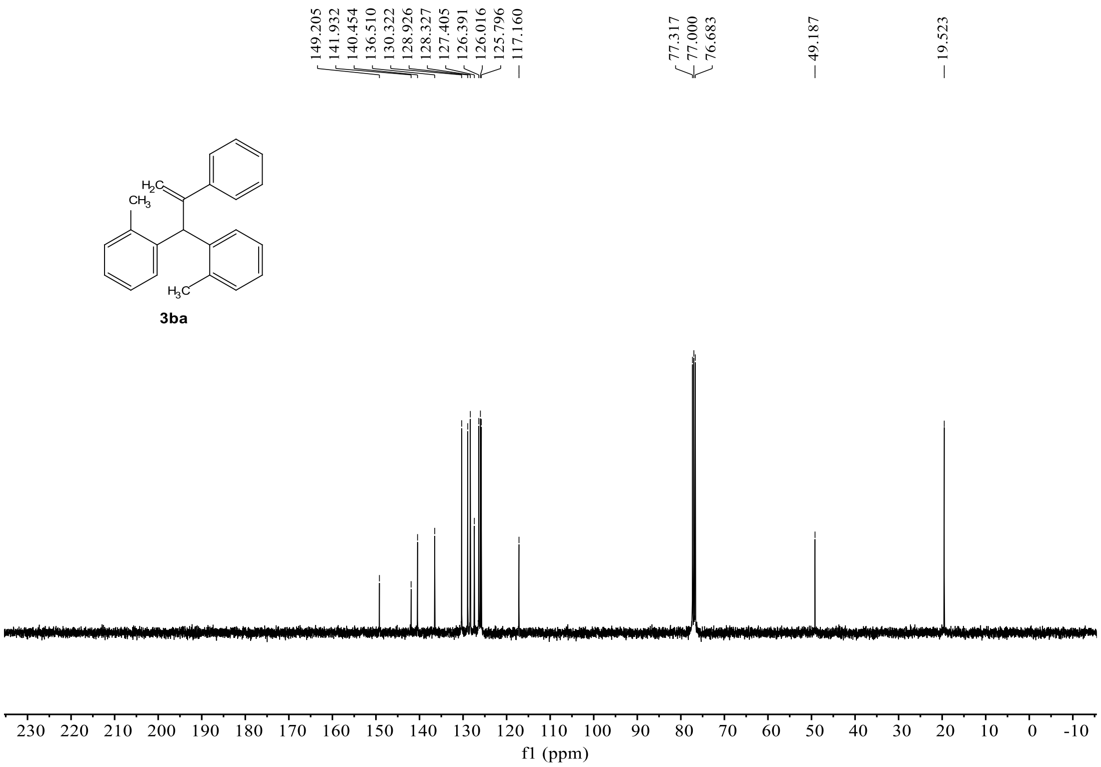

Figure S45. ^{1}H NMR ($CDCl_3$, 400 MHz) and ^{13}C NMR ($CDCl_3$, 100 MHz) spectra of compound **3ba**

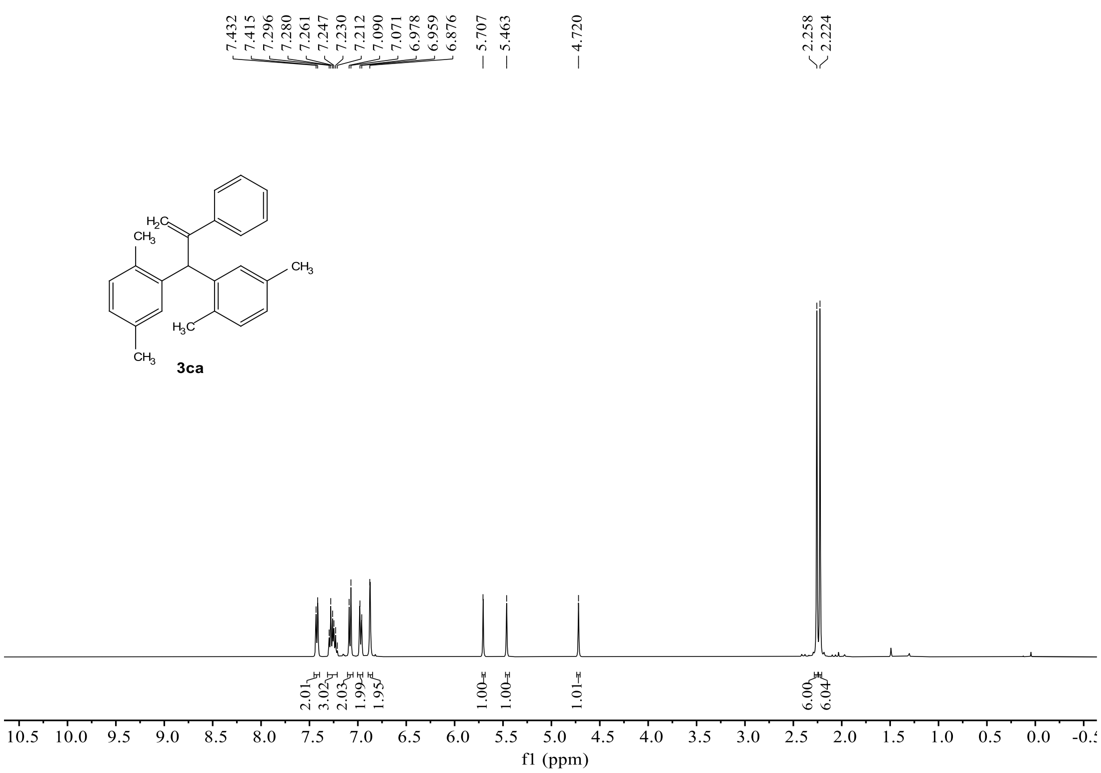

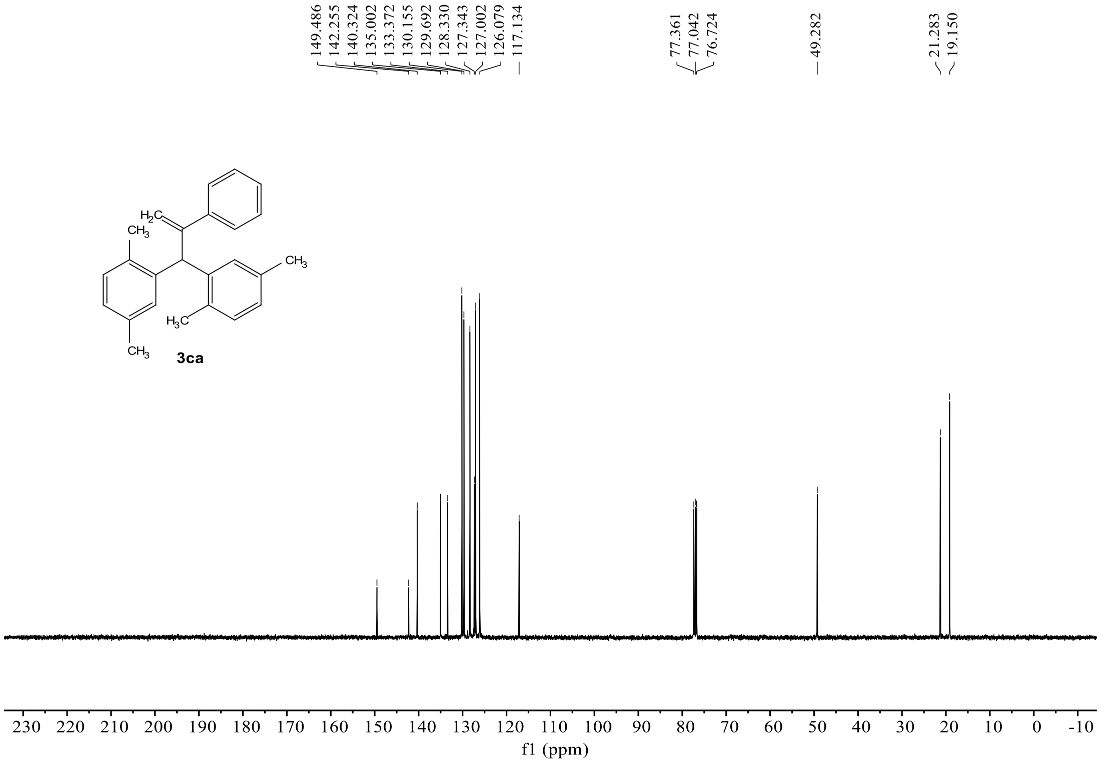

Figure S46. ^{1}H NMR ($CDCl_3$, 400 MHz) and ^{13}C NMR ($CDCl_3$, 100 MHz) spectra of compound **3ca**

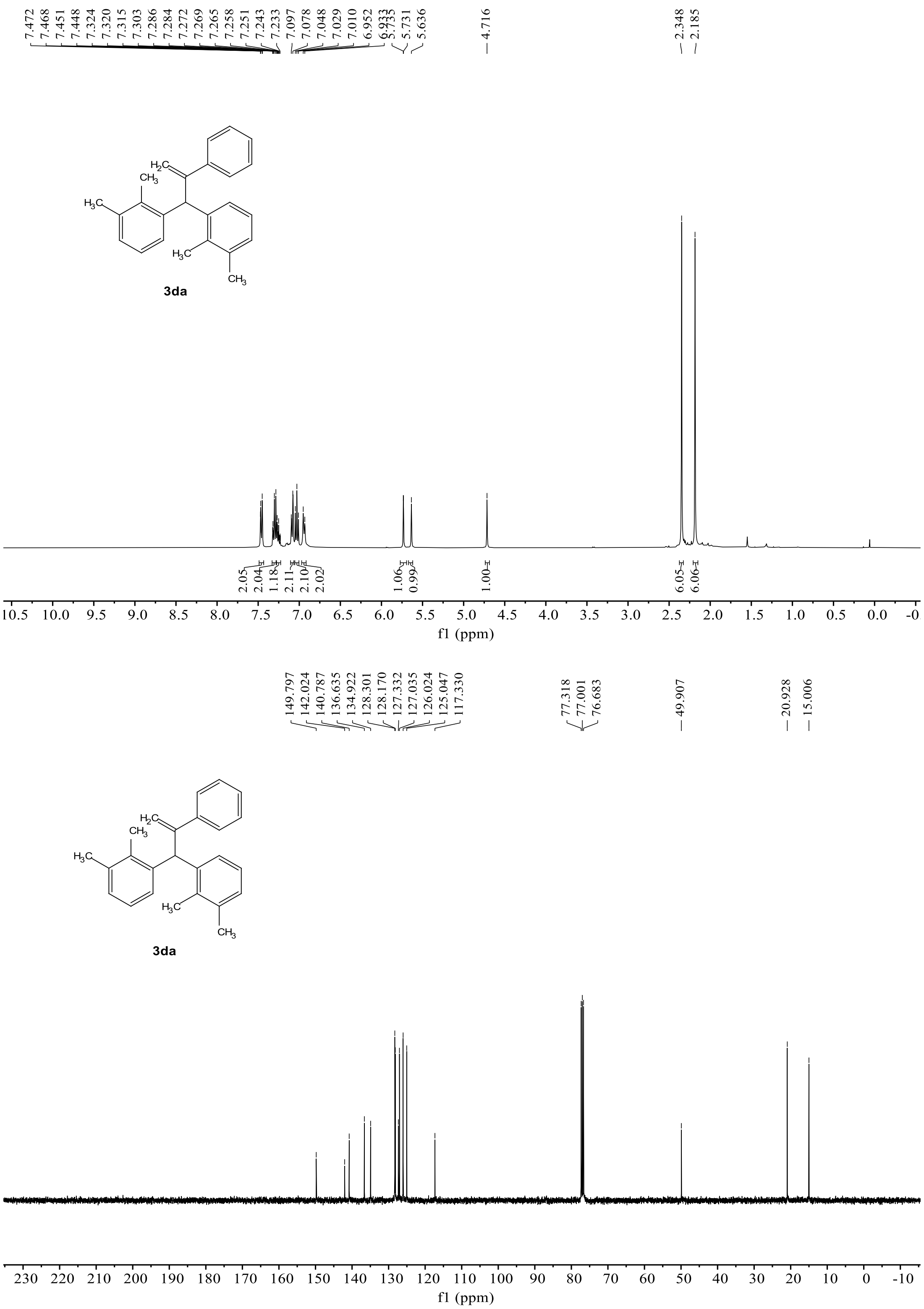

Figure S47. ^{1}H NMR ($CDCl_3$, 400 MHz) and ^{13}C NMR ($CDCl_3$, 100 MHz) spectra of compound **3da**

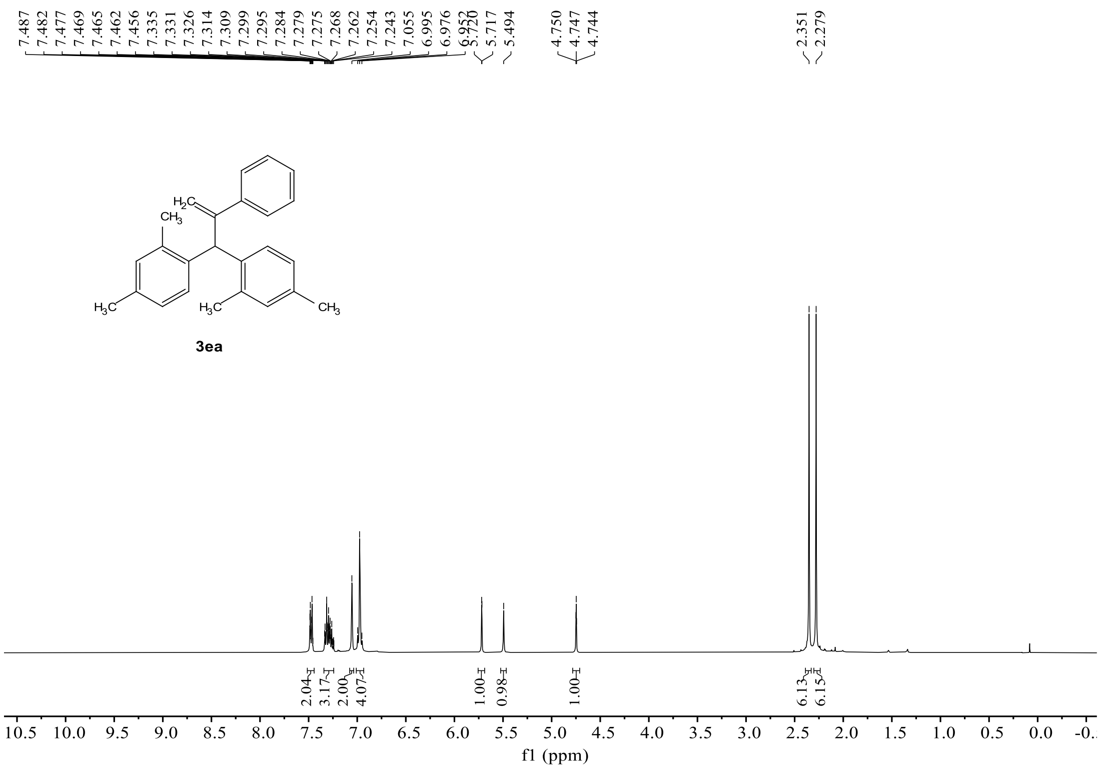

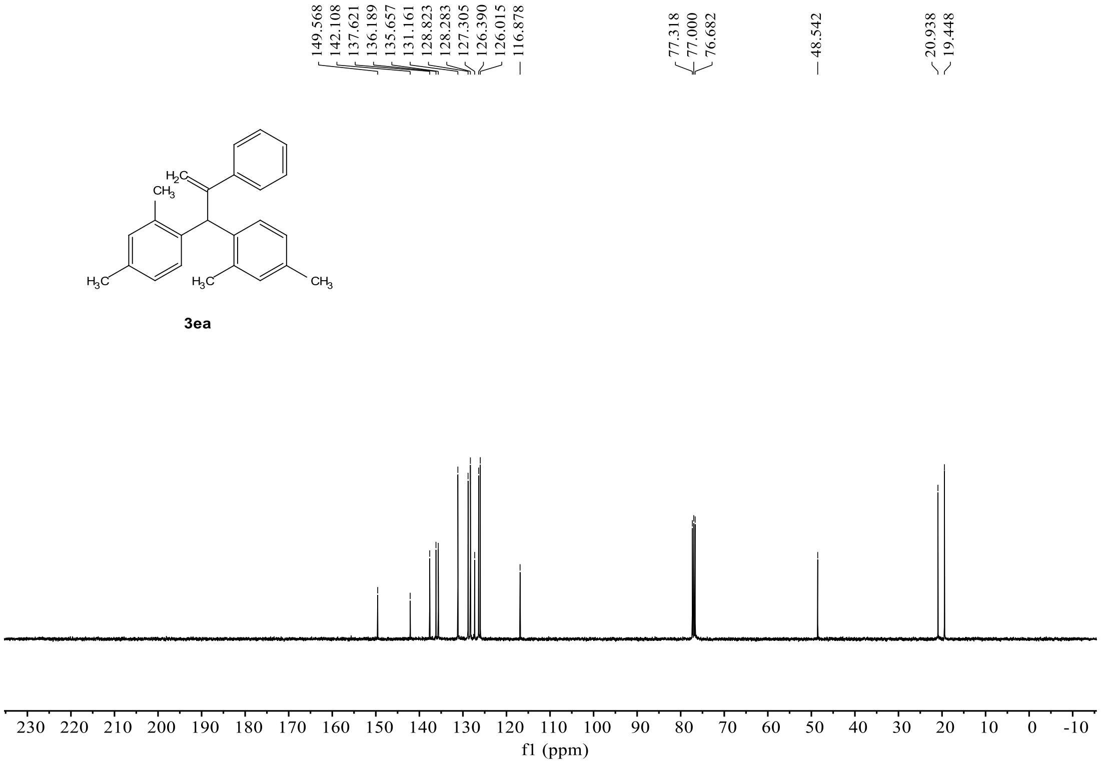

Figure S48. ^{1}H NMR ($CDCl_3$, 400 MHz) and ^{13}C NMR ($CDCl_3$, 100 MHz) spectra of compound **3ea**

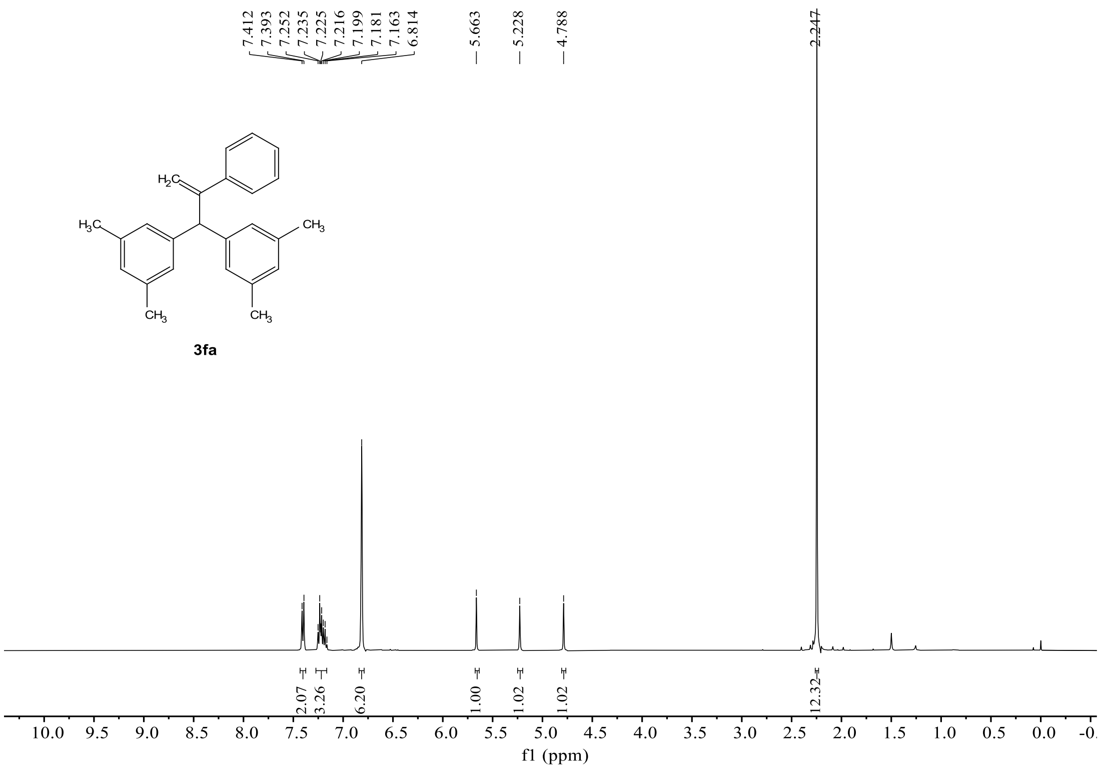

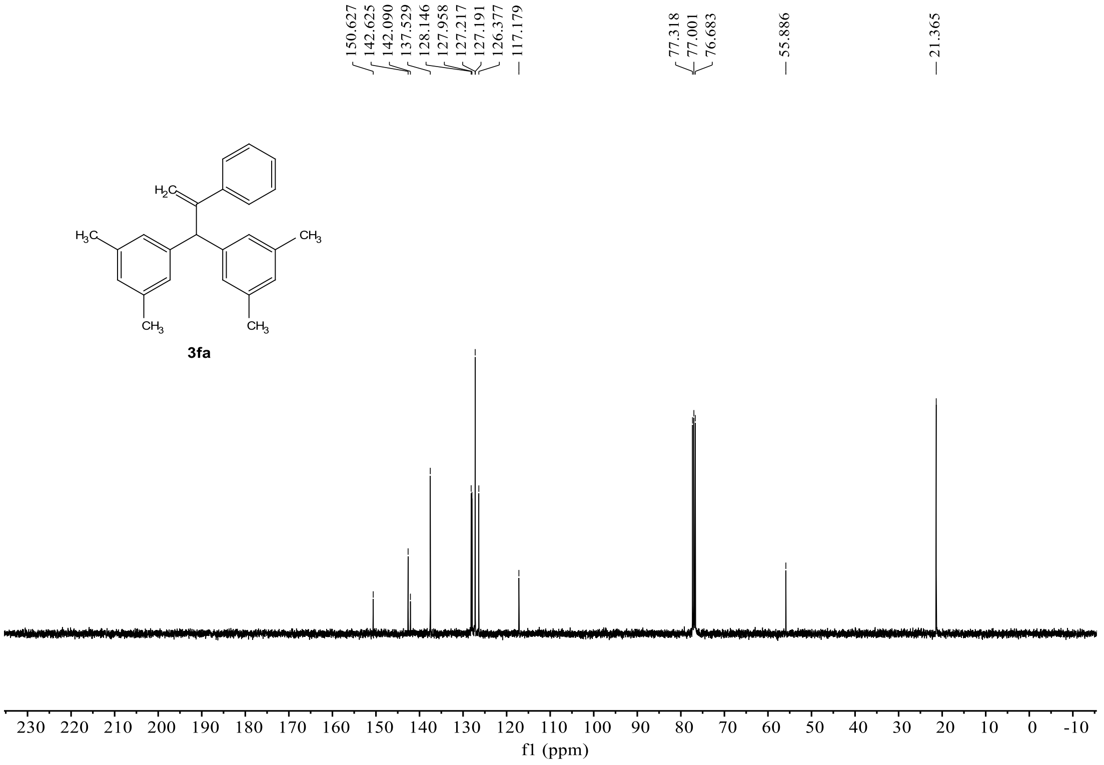

Figure S49. ^{1}H NMR ($CDCl_3$, 400 MHz) and ^{13}C NMR ($CDCl_3$, 100 MHz) spectra of compound **3fa**

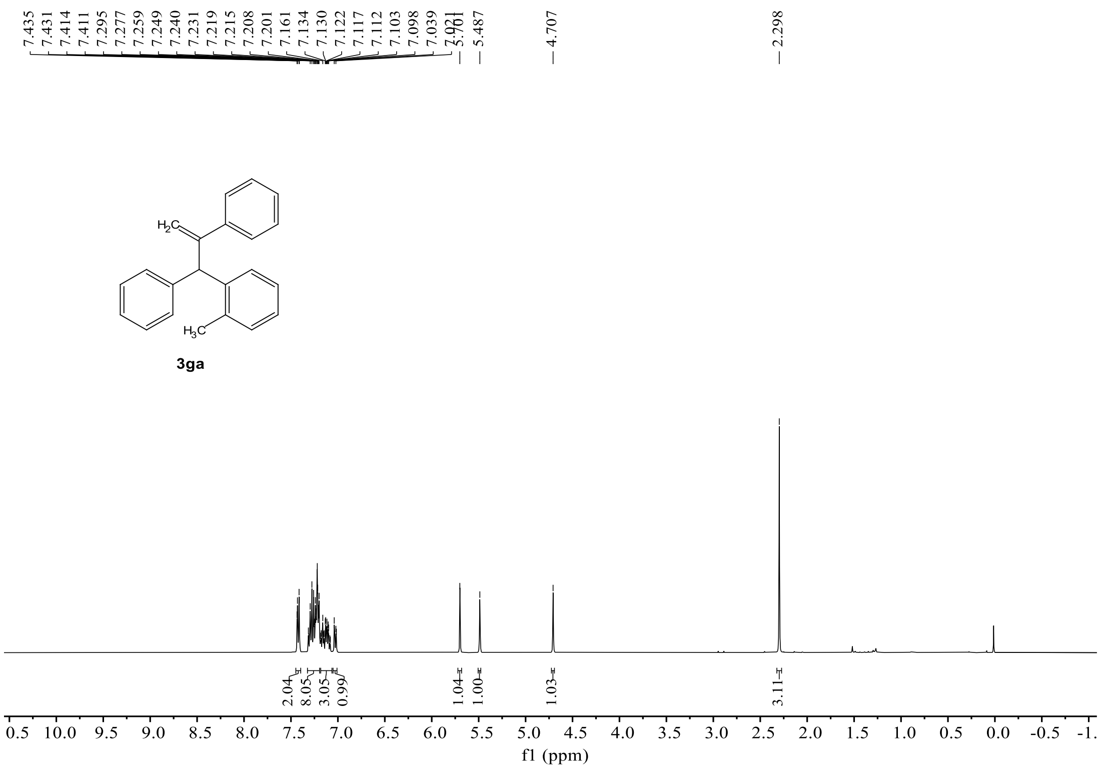

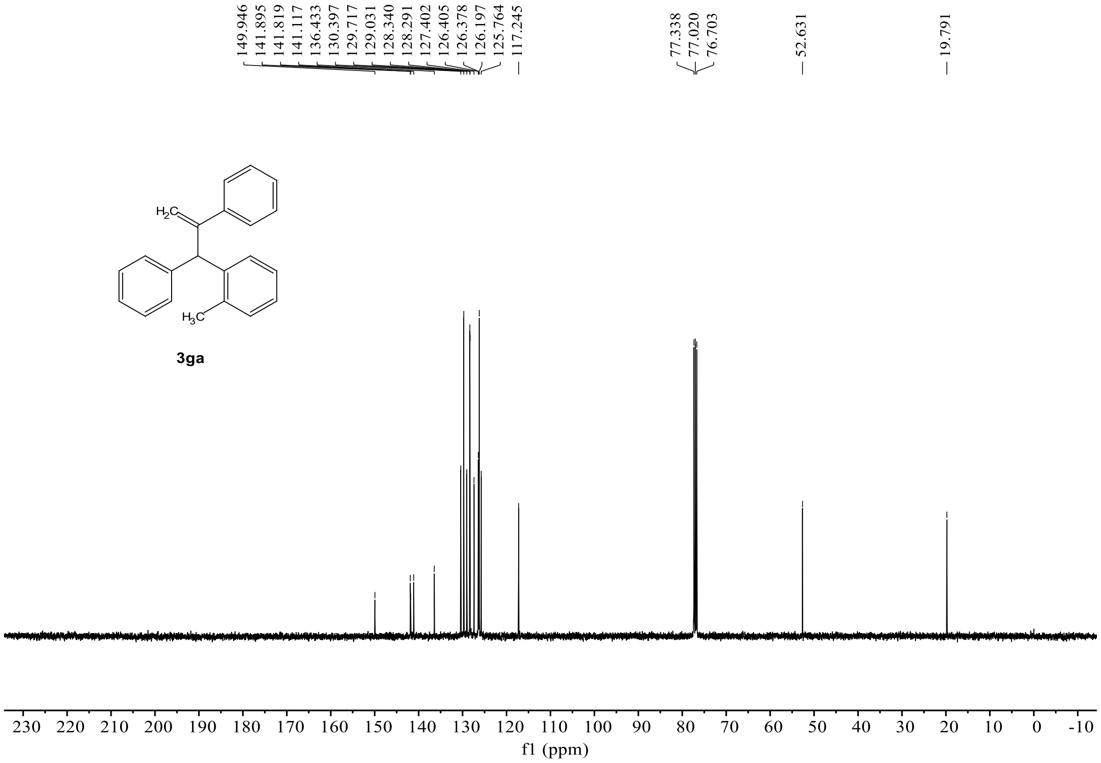

Figure S50. ^{1}H NMR ($CDCl_3$, 400 MHz) and ^{13}C NMR ($CDCl_3$, 100 MHz) spectra of compound **3ga**

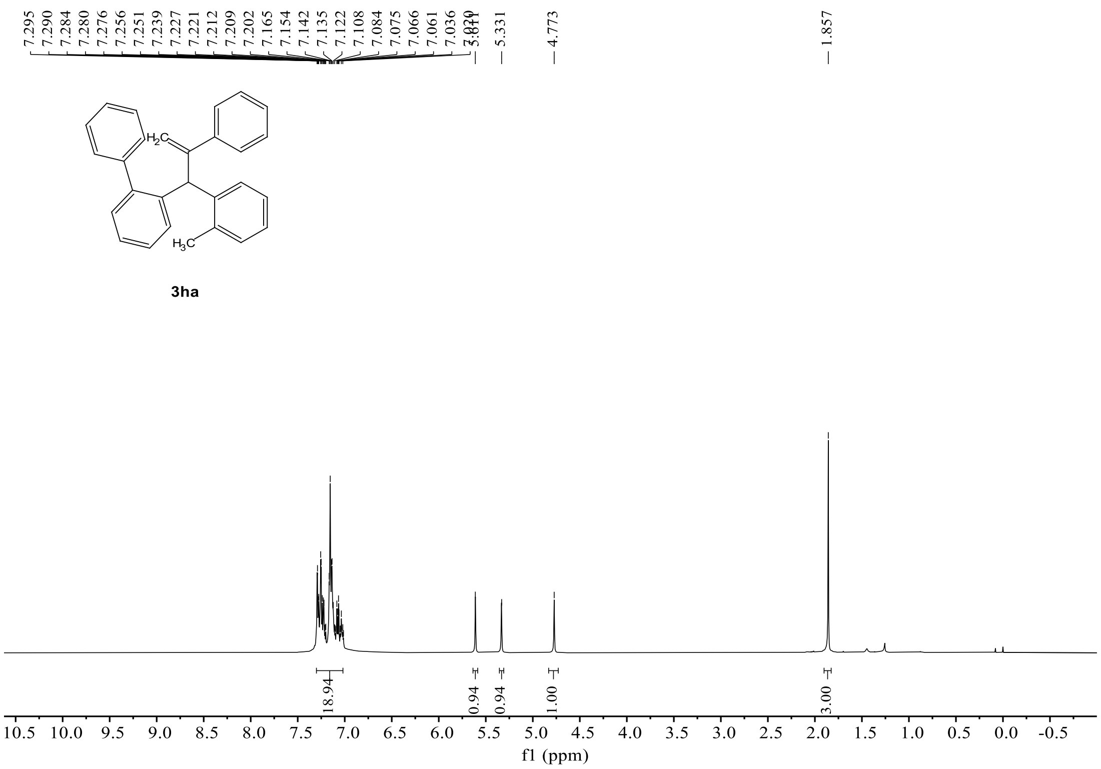

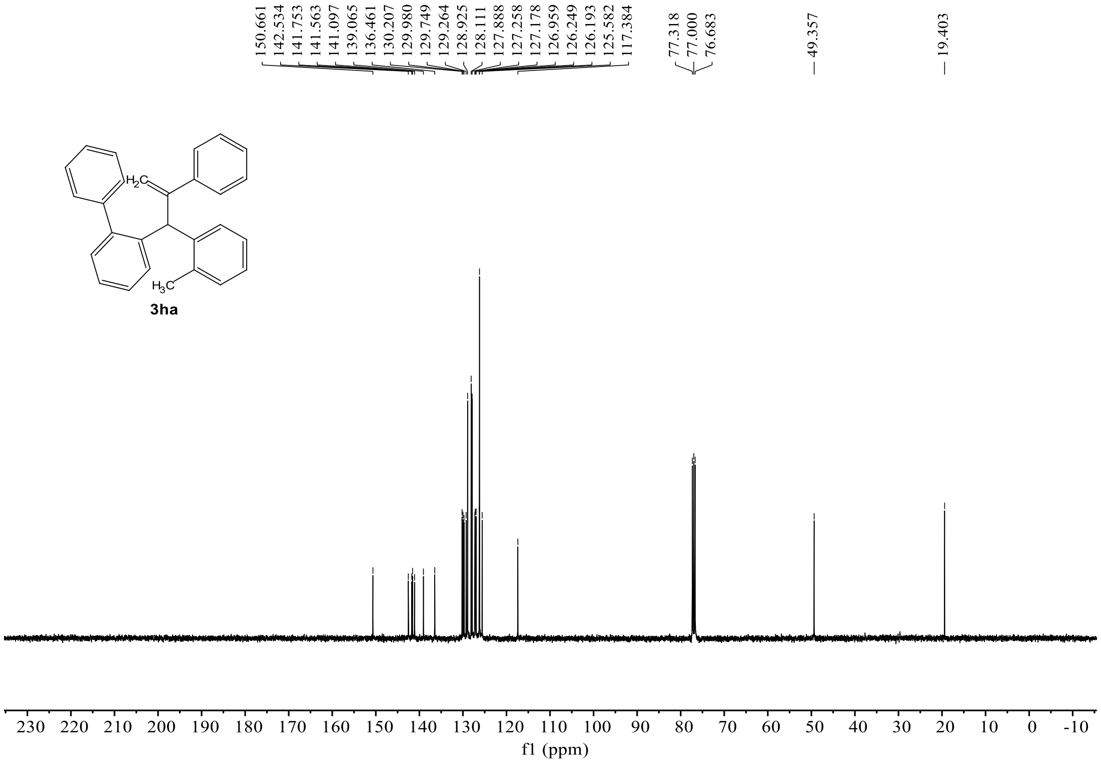

Figure S51. ^{1}H NMR ($CDCl_3$, 400 MHz) and ^{13}C NMR ($CDCl_3$, 100 MHz) spectra of compound **3ha**

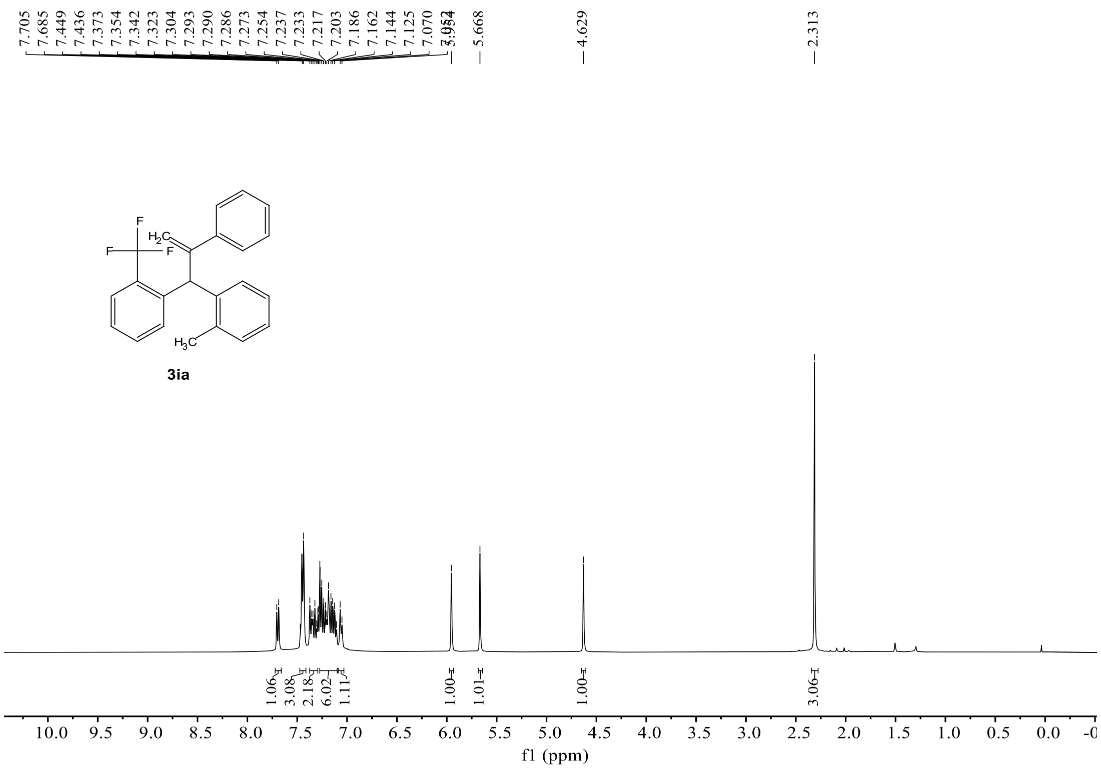
7.705
7.685
7.449
7.436
7.373
7.354
7.342
7.323
7.304
7.293
7.290
7.286
7.273
7.254
7.237
7.233
7.217
7.203
7.186
7.162
7.144
7.125
7.070
5.668
4.629
2.313
F
F
F
H_2C
H_3C
3ia
1.06
3.08
2.18
6.02
1.11
1.00
1.01
1.00
3.06
10.0 9.5 9.0 8.5 8.0 7.5 7.0 6.5 6.0 5.5 5.0 4.5 4.0 3.5 3.0 2.5 2.0 1.5 1.0 0.5 0.0
f1 (ppm)

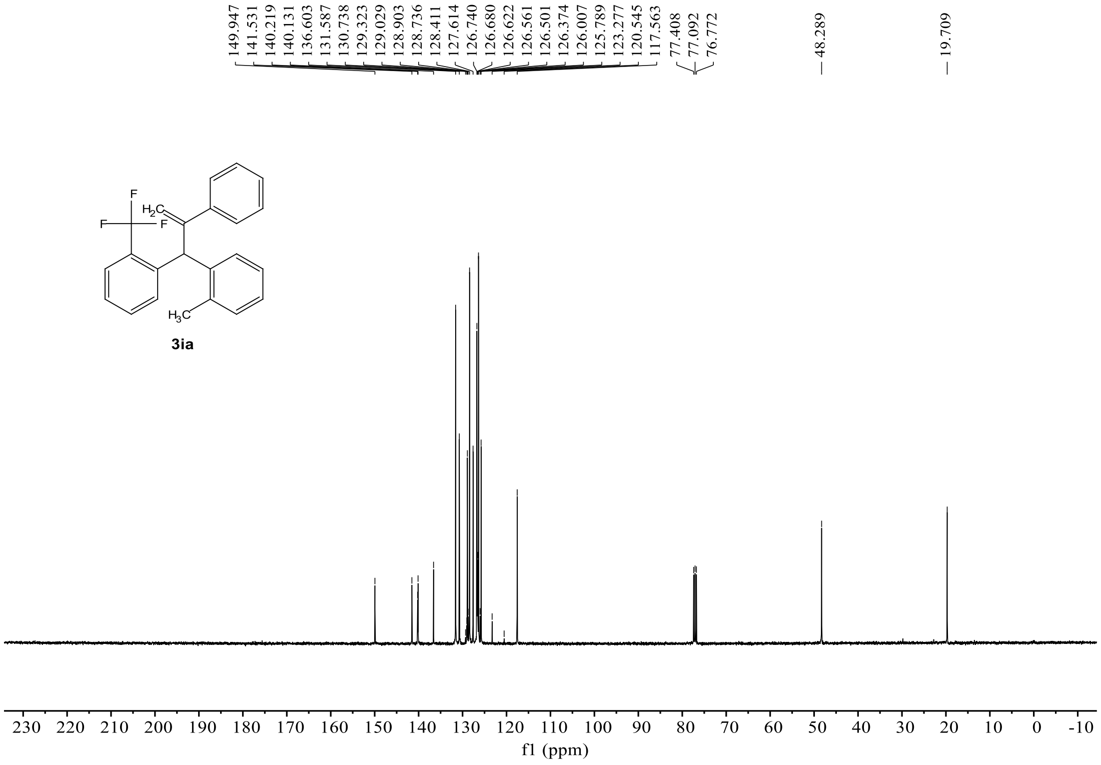
149.947
141.531
140.219
140.131
136.603
131.587
130.738
129.323
129.029
128.903
128.736
128.411
127.614
126.740
126.680
126.622
126.561
126.501
126.374
126.007
125.789
123.277
120.545
117.563
77.408
77.092
76.772
48.289
19.709
F
F
F
H_2C
H_3C
3ia
230 220 210 200 190 180 170 160 150 140 130 120 110 100 90 80 70 60 50 40 30 20 10 0 -10
f1 (ppm)

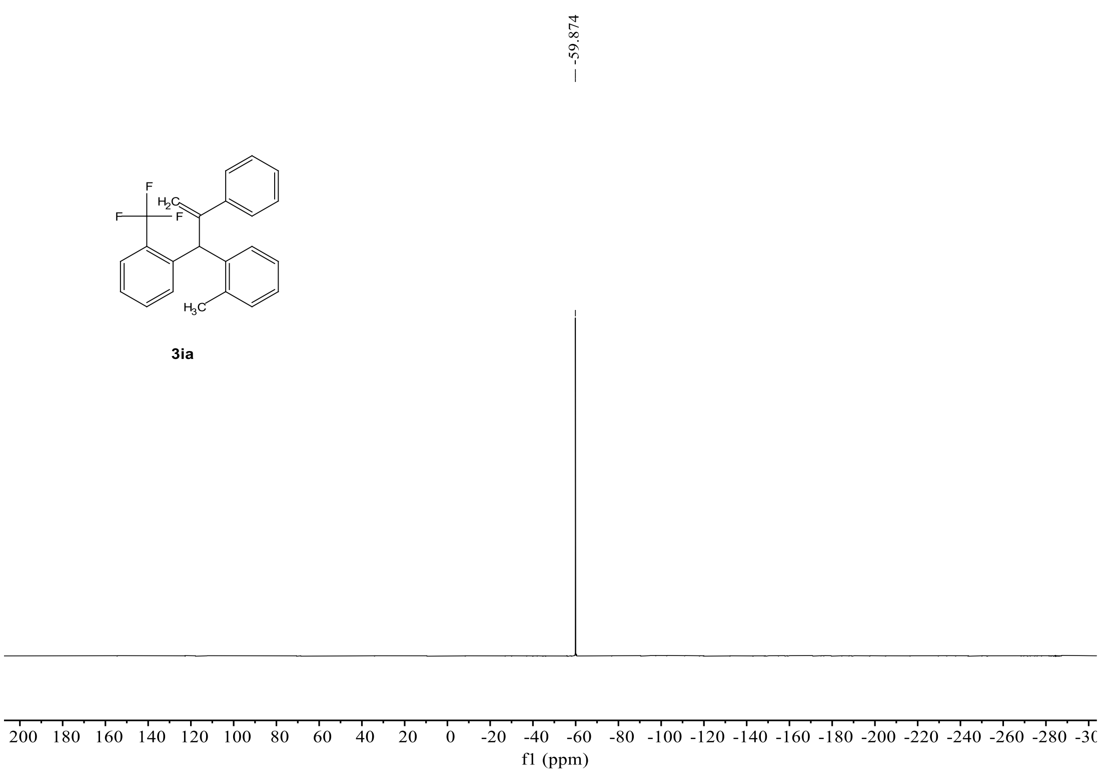

Figure S52. ^{1}H NMR ($CDCl_3$, 400 MHz), ^{13}C NMR ($CDCl_3$, 100 MHz), ^{19}F NMR ($CDCl_3$, 376 MHz) spectra of compound **3ia**

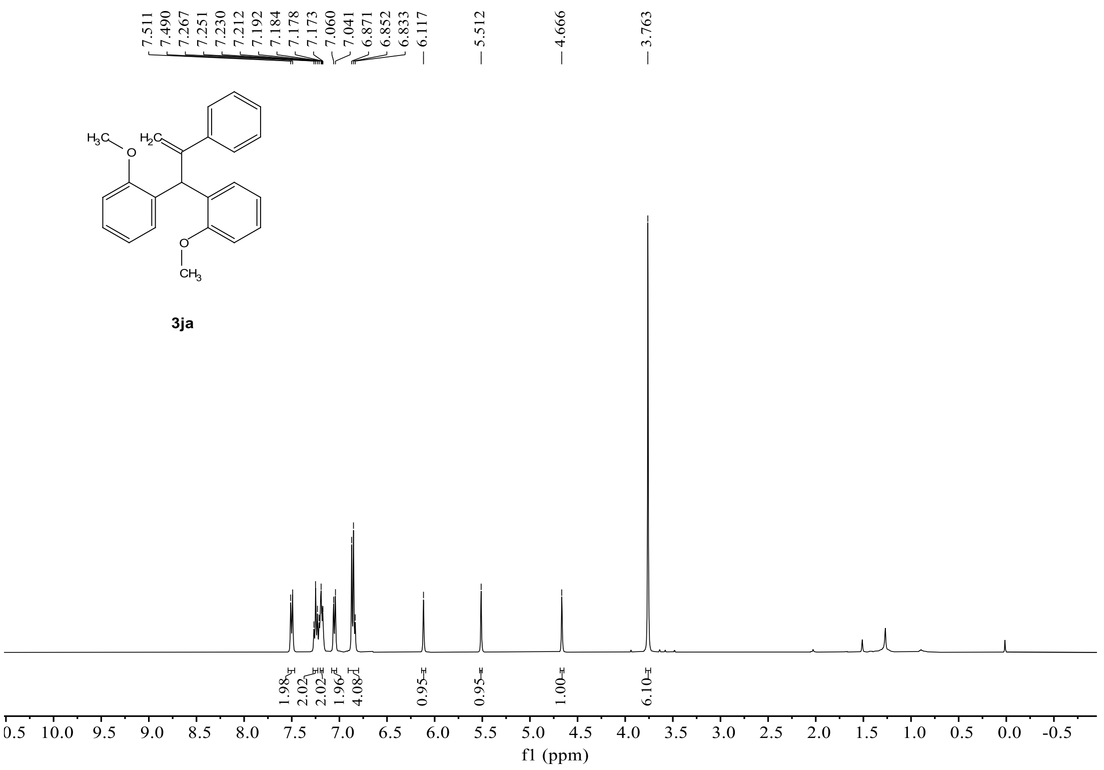

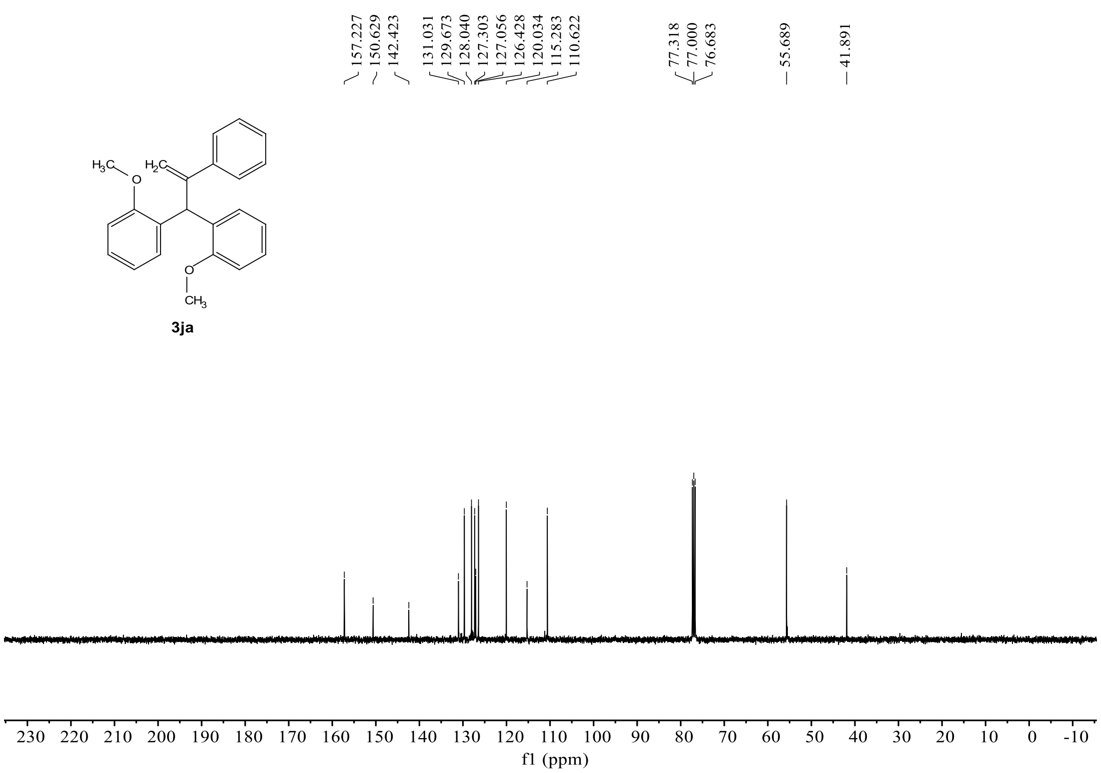

Figure S53. ^{1}H NMR ($CDCl_3$, 400 MHz) and ^{13}C NMR ($CDCl_3$, 100 MHz) spectra of compound **3ja**

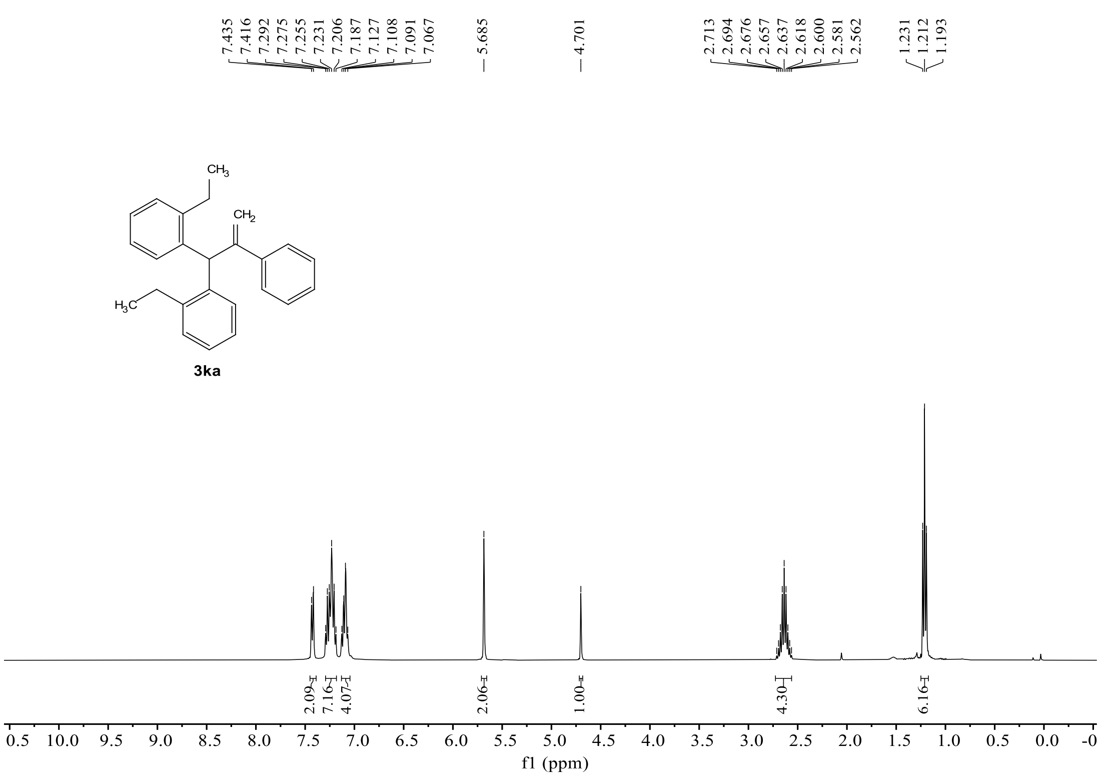

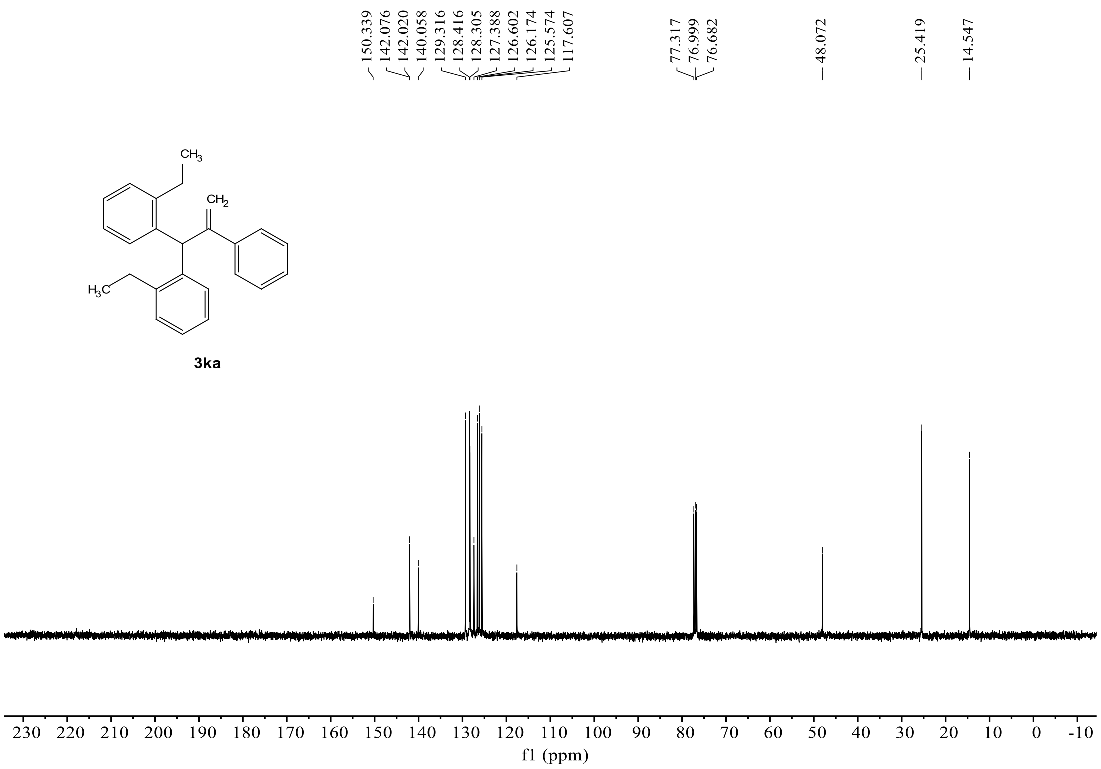

Figure S54. ^{1}H NMR ($CDCl_3$, 400 MHz) and ^{13}C NMR ($CDCl_3$, 100 MHz) spectra of compound **3ka**

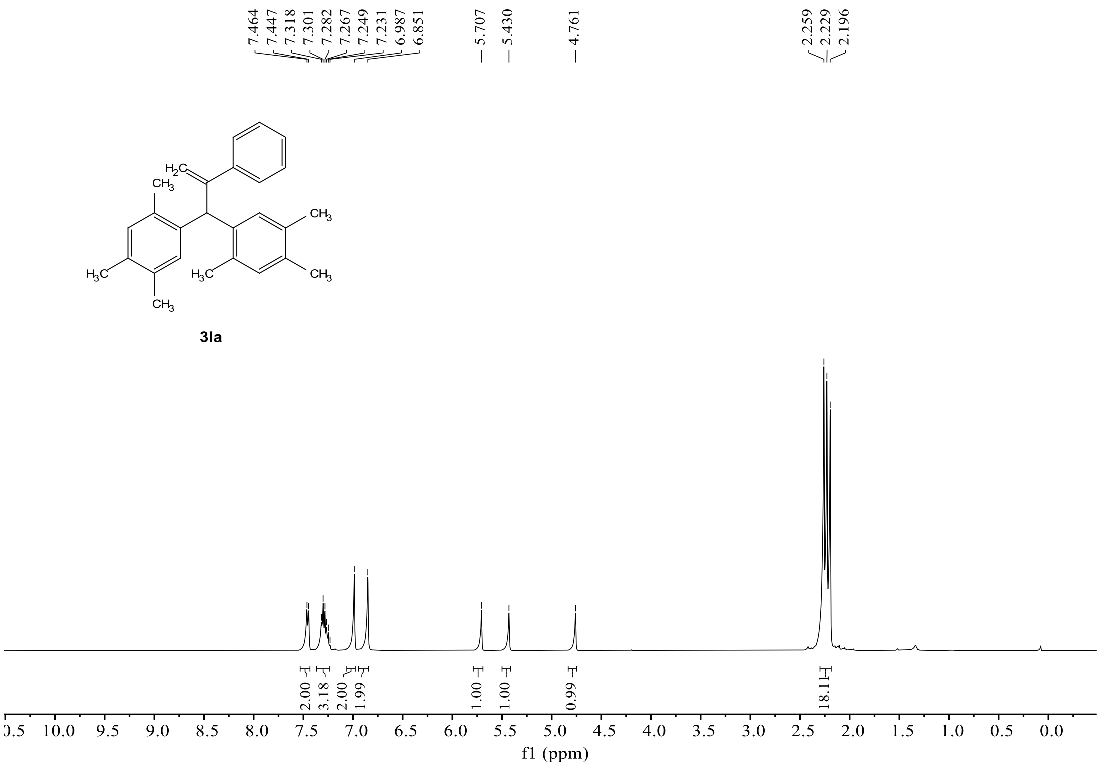

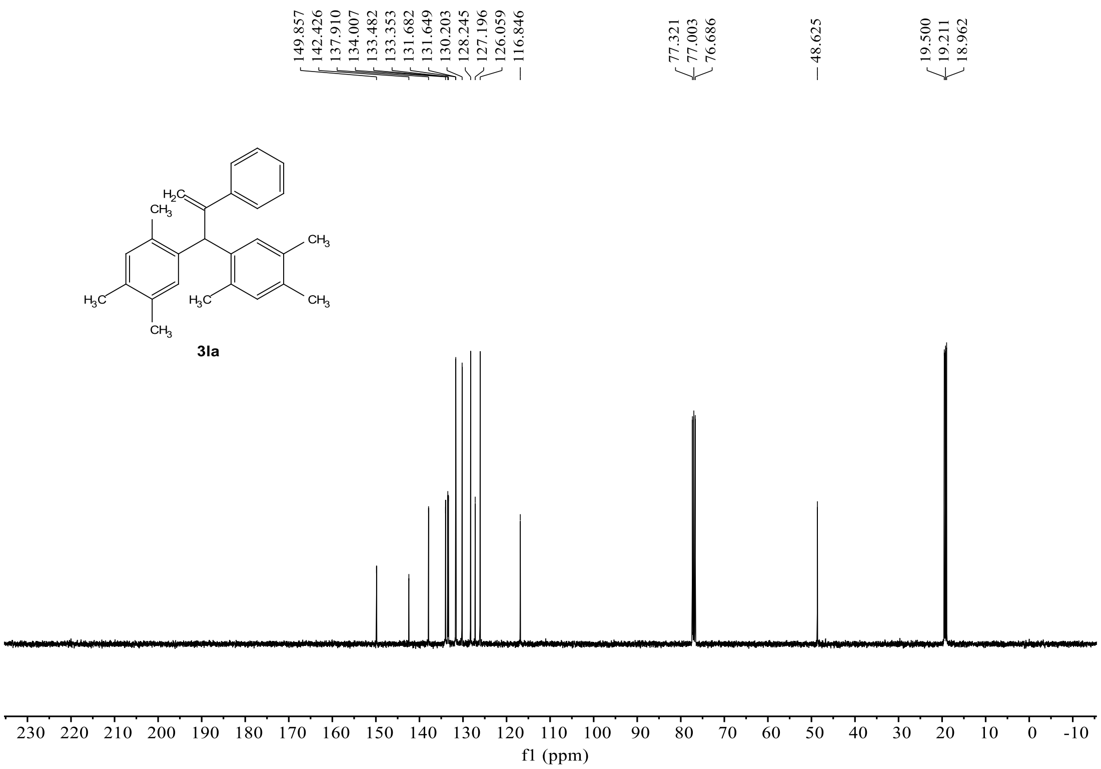

Figure S55. ^{1}H NMR ($CDCl_3$, 400 MHz) and ^{13}C NMR ($CDCl_3$, 100 MHz) spectra of compound **3la**

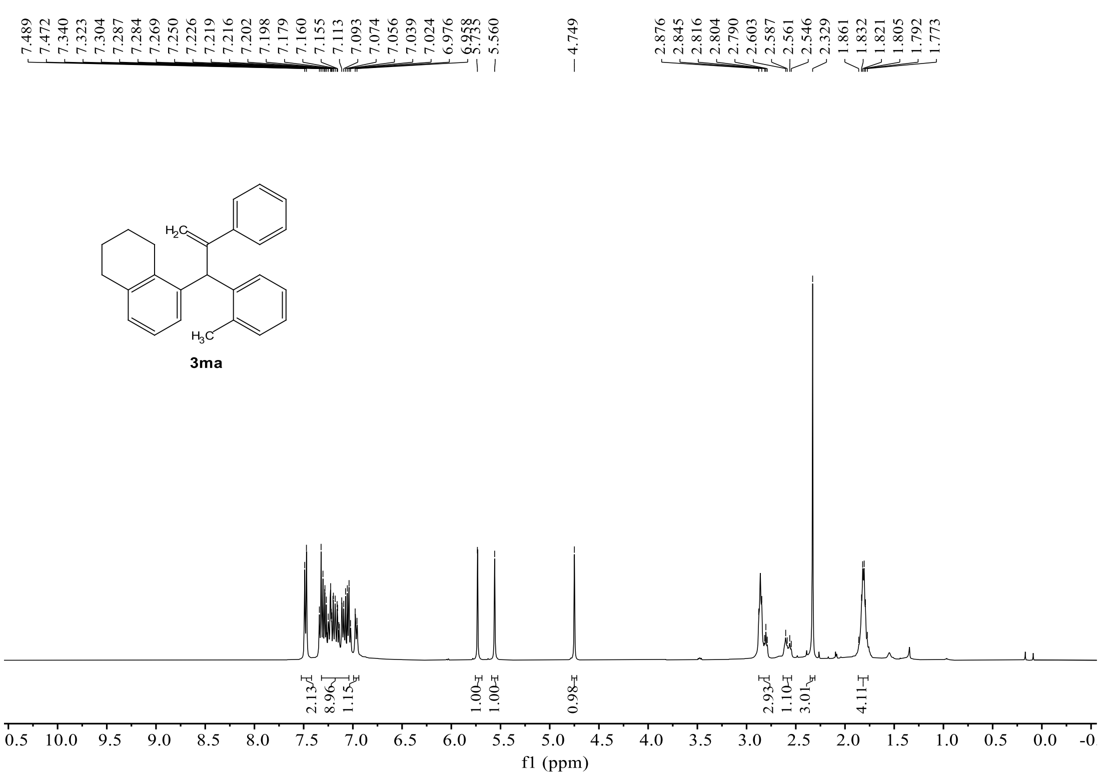

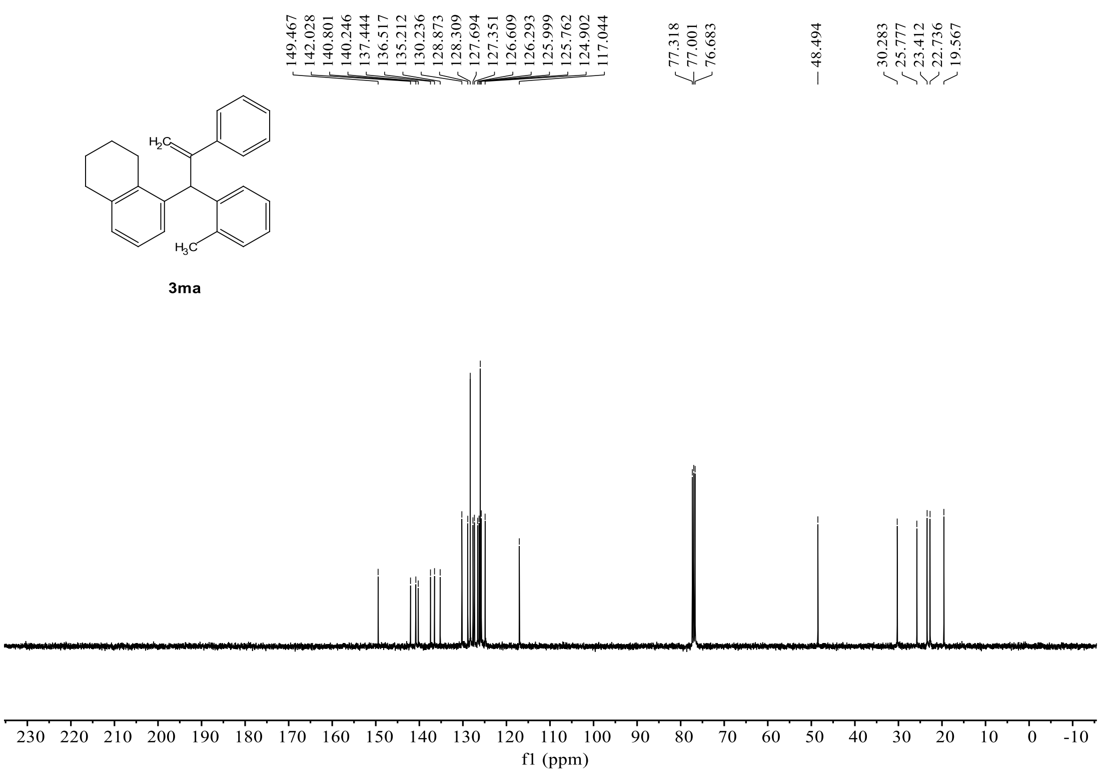

Figure S56. ^{1}H NMR ($CDCl_3$, 400 MHz) and ^{13}C NMR ($CDCl_3$, 100 MHz) spectra of compound **3ma**

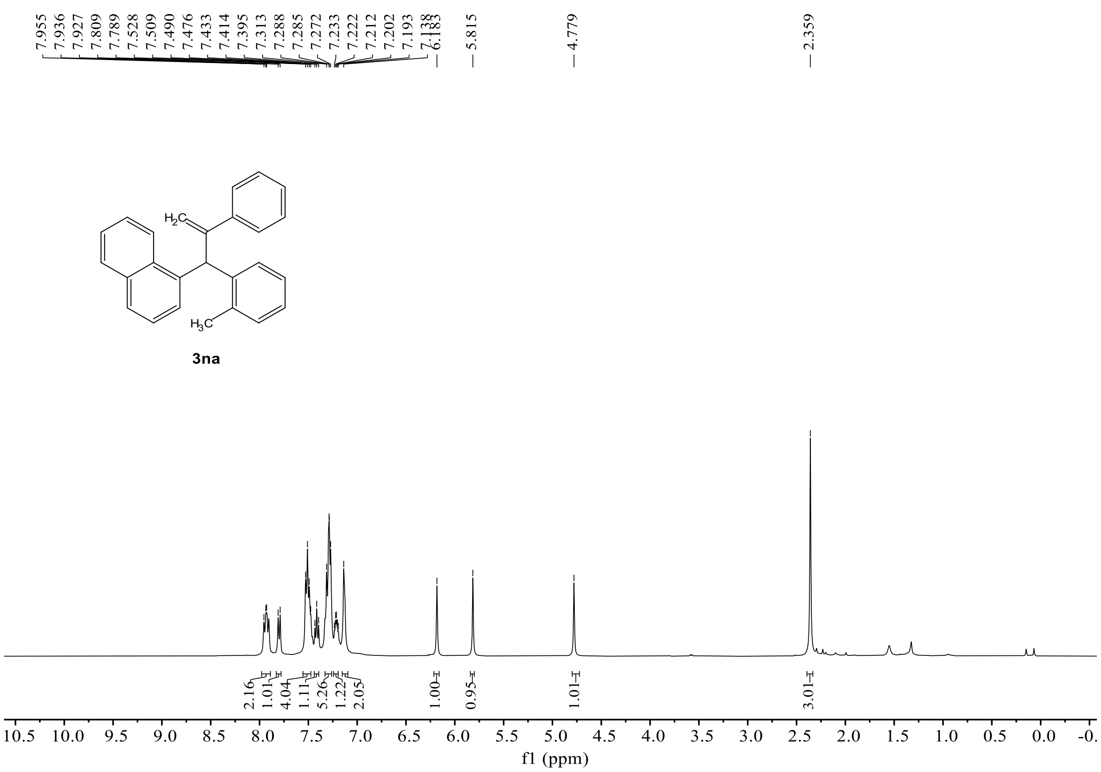

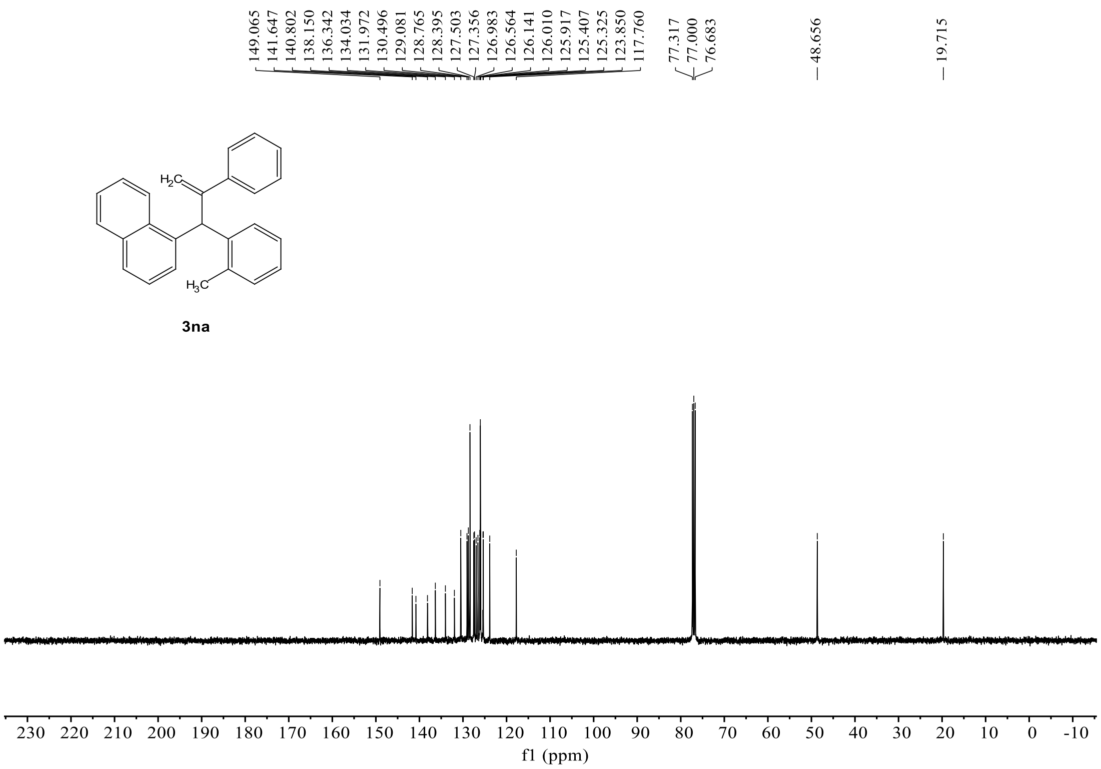

Figure S57. ^{1}H NMR ($CDCl_3$, 400 MHz) and ^{13}C NMR ($CDCl_3$, 100 MHz) spectra of compound **3na**

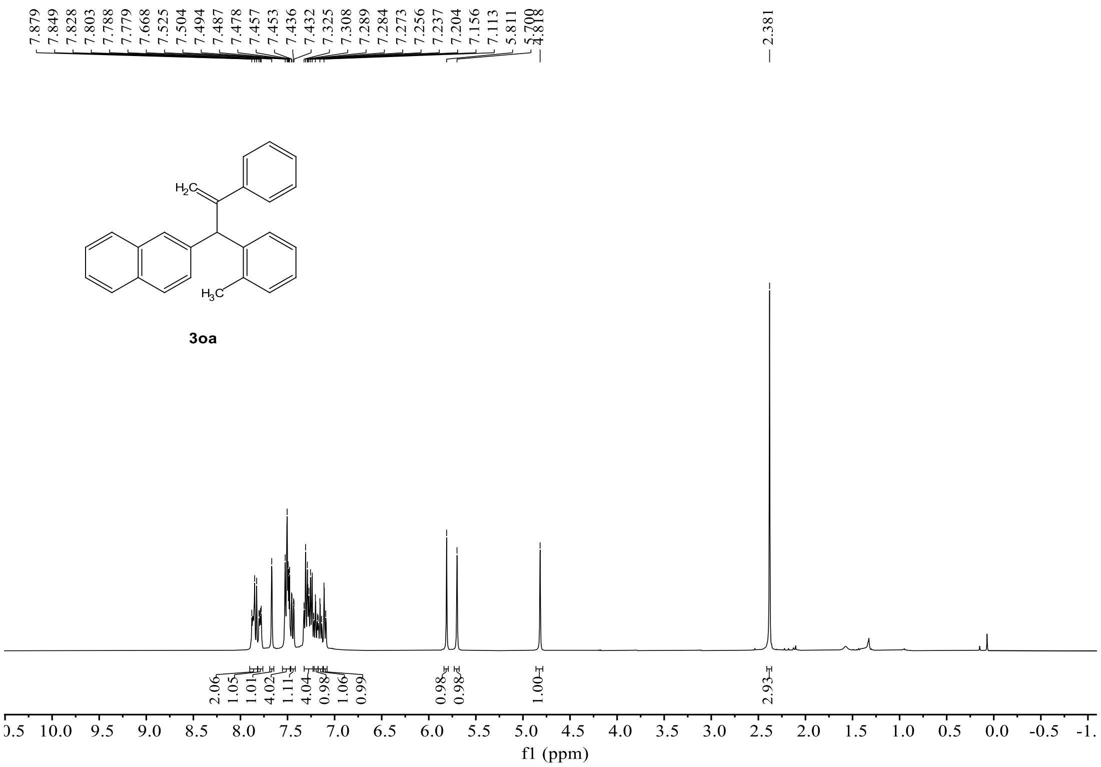

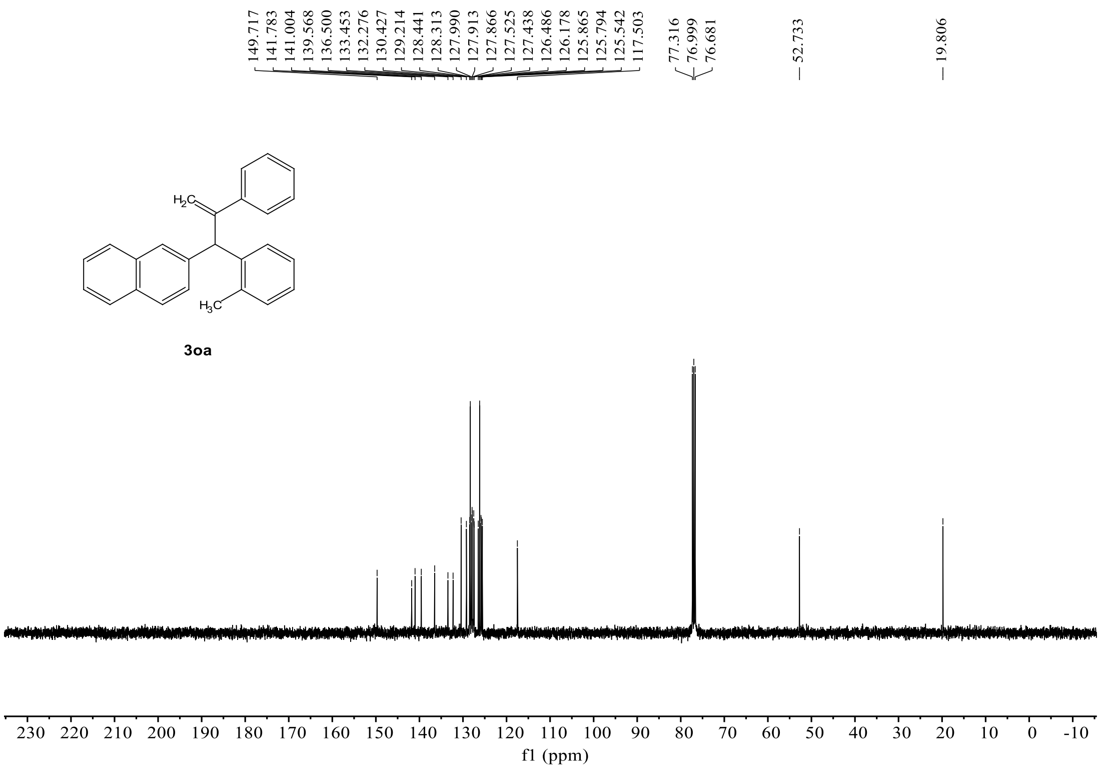

Figure S58. ^{1}H NMR ($CDCl_3$, 400 MHz) and ^{13}C NMR ($CDCl_3$, 100 MHz) spectra of compound **3oa**

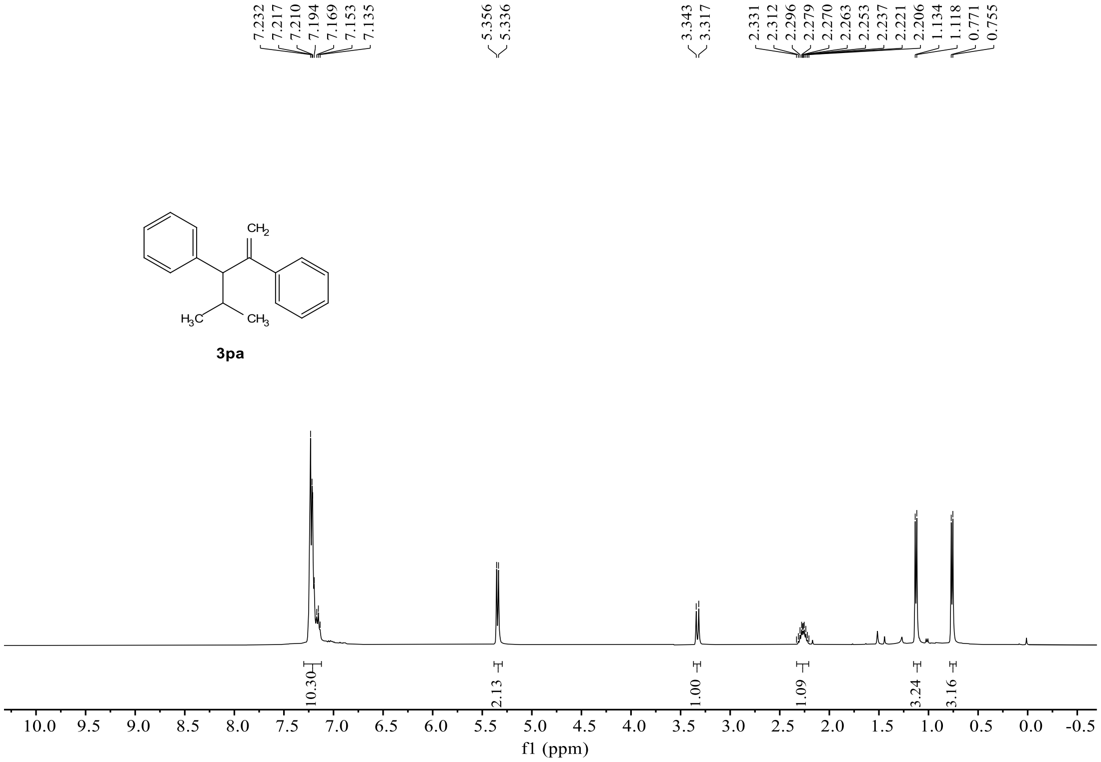

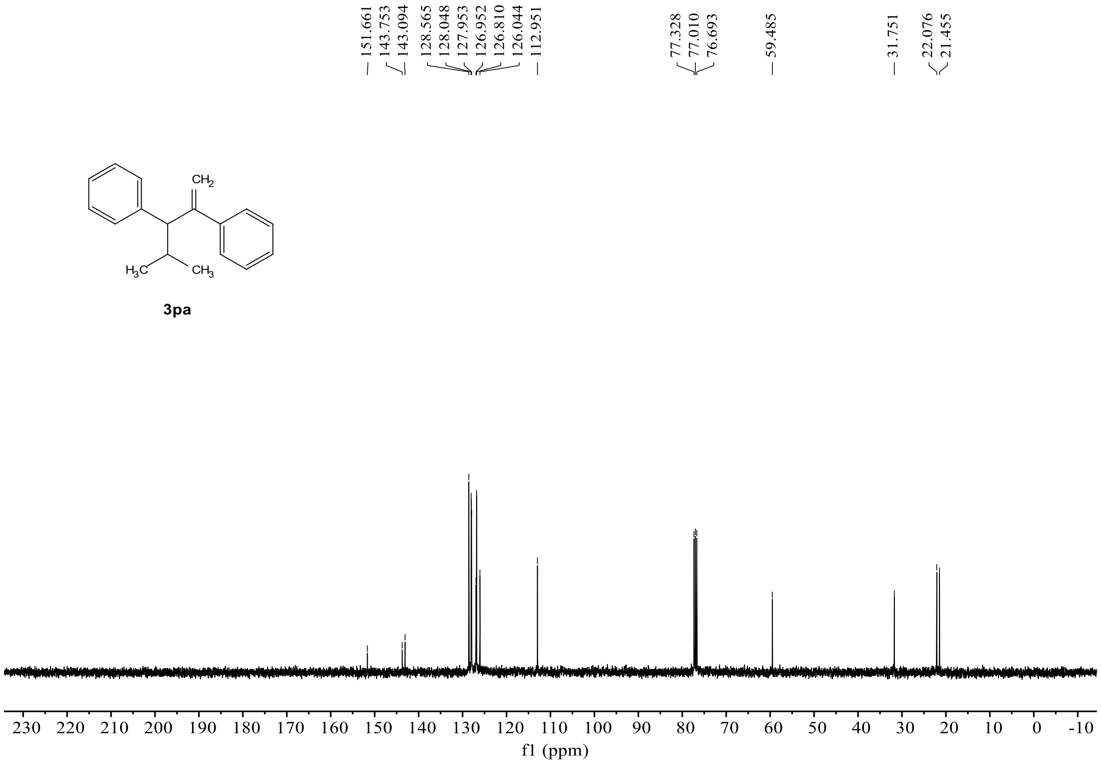

Figure S59. ^{1}H NMR ($CDCl_3$, 400 MHz) and ^{13}C NMR ($CDCl_3$, 100 MHz) spectra of compound **3pa**

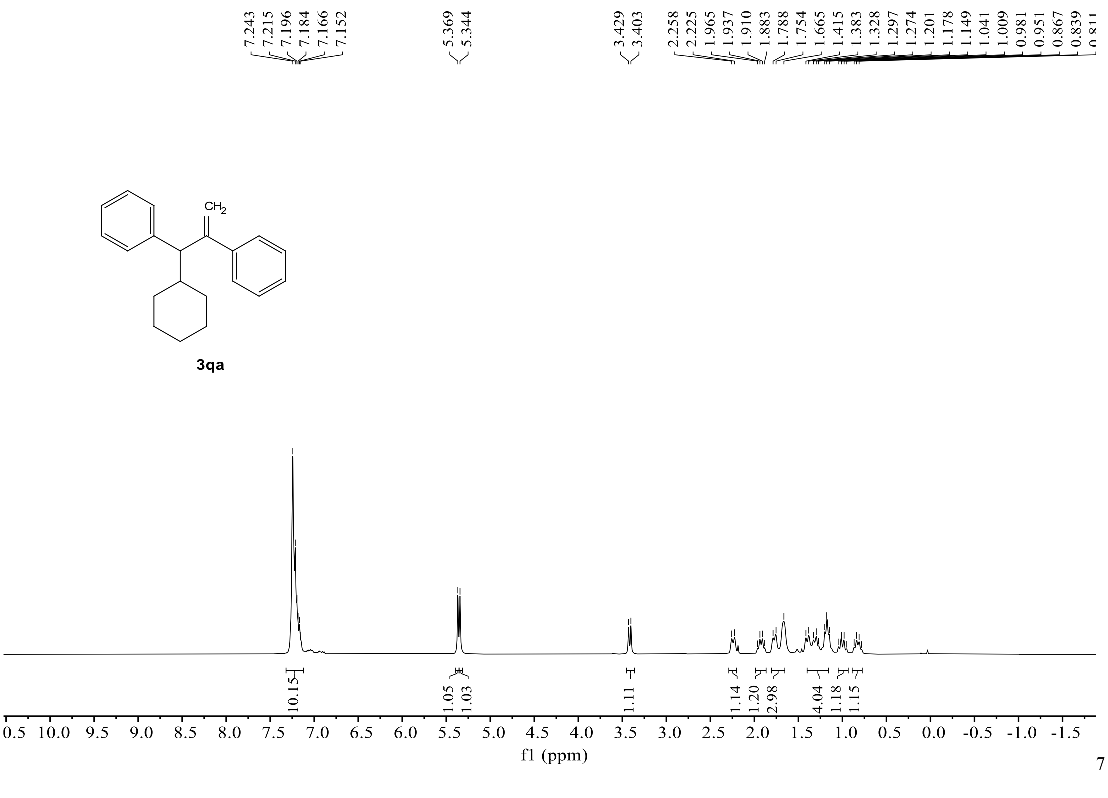

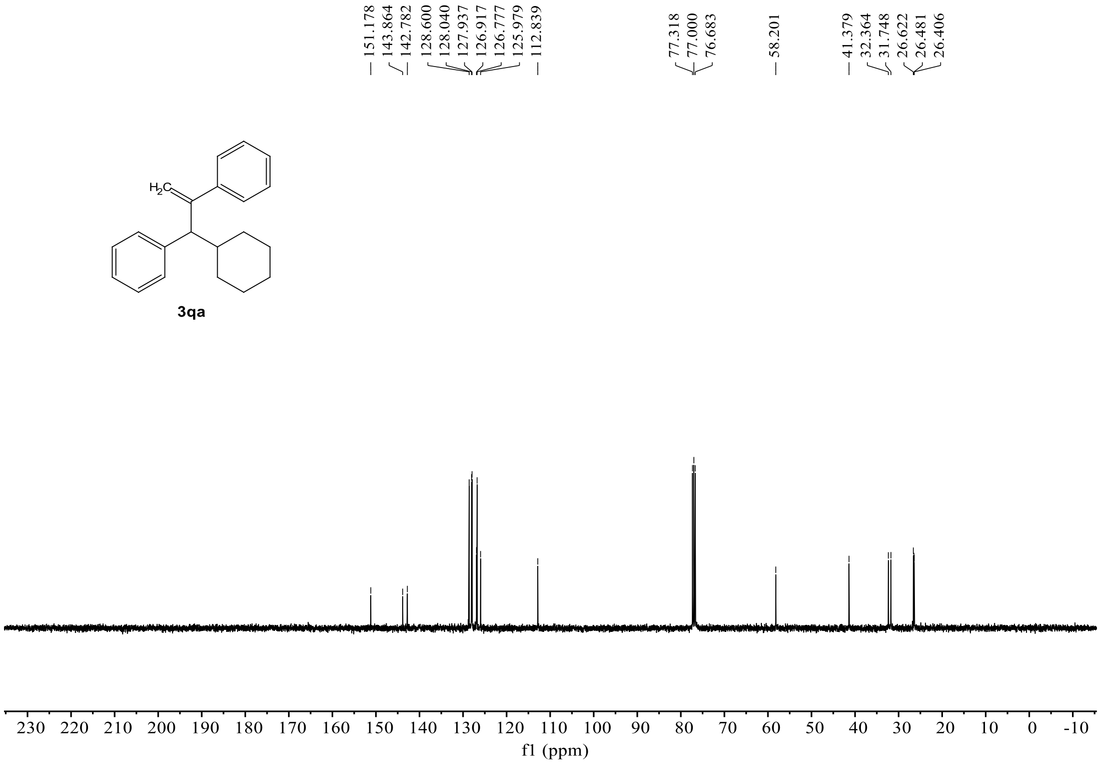

Figure S60. ^{1}H NMR ($CDCl_3$, 400 MHz) and ^{13}C NMR ($CDCl_3$, 100 MHz) spectra of compound **3qa**

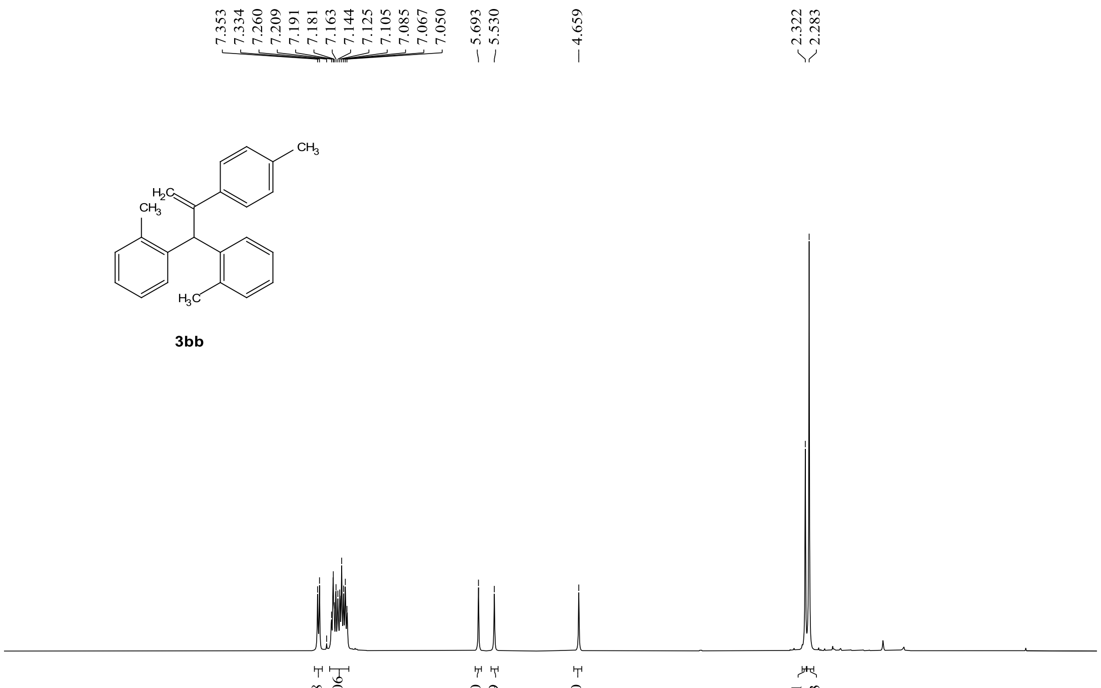

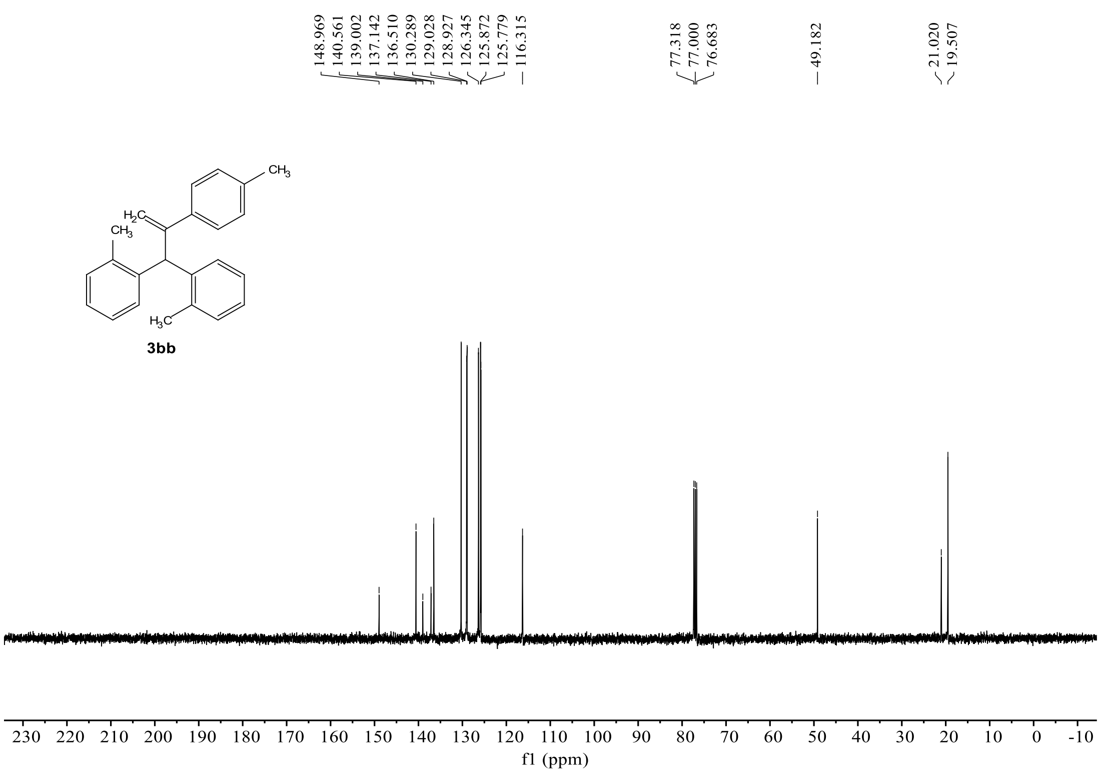

Figure S61. ^{1}H NMR ($CDCl_3$, 400 MHz) and ^{13}C NMR ($CDCl_3$, 100 MHz) spectra of compound **3bb**

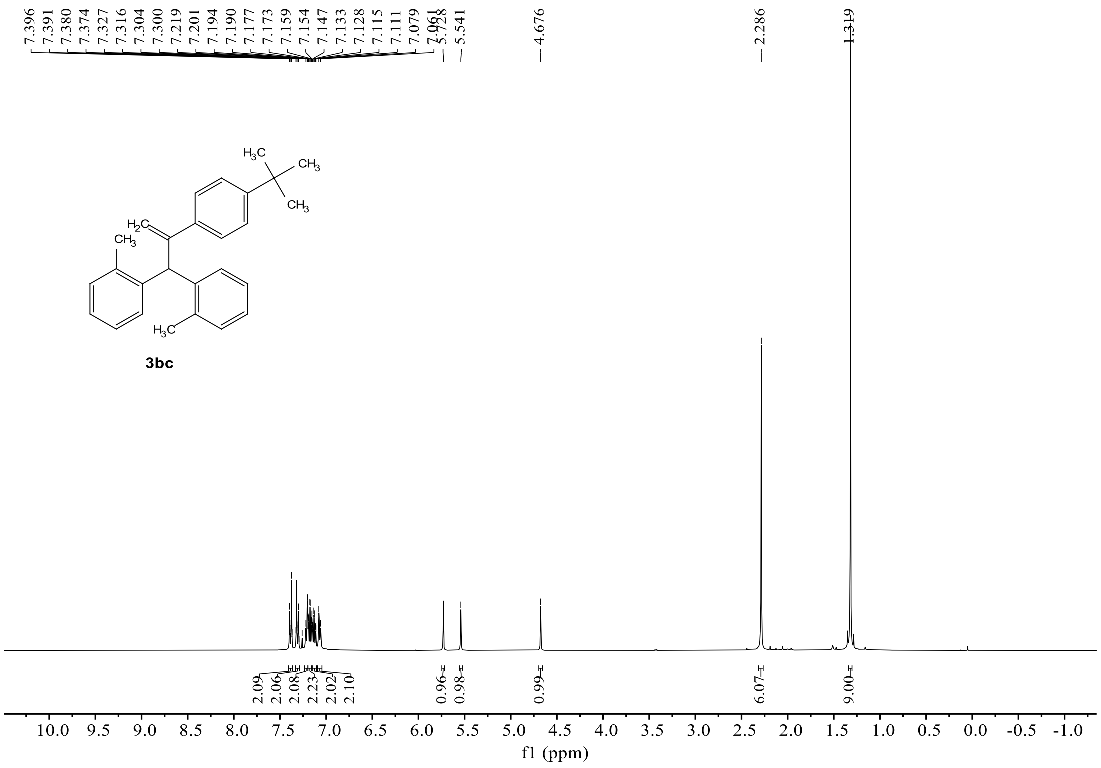

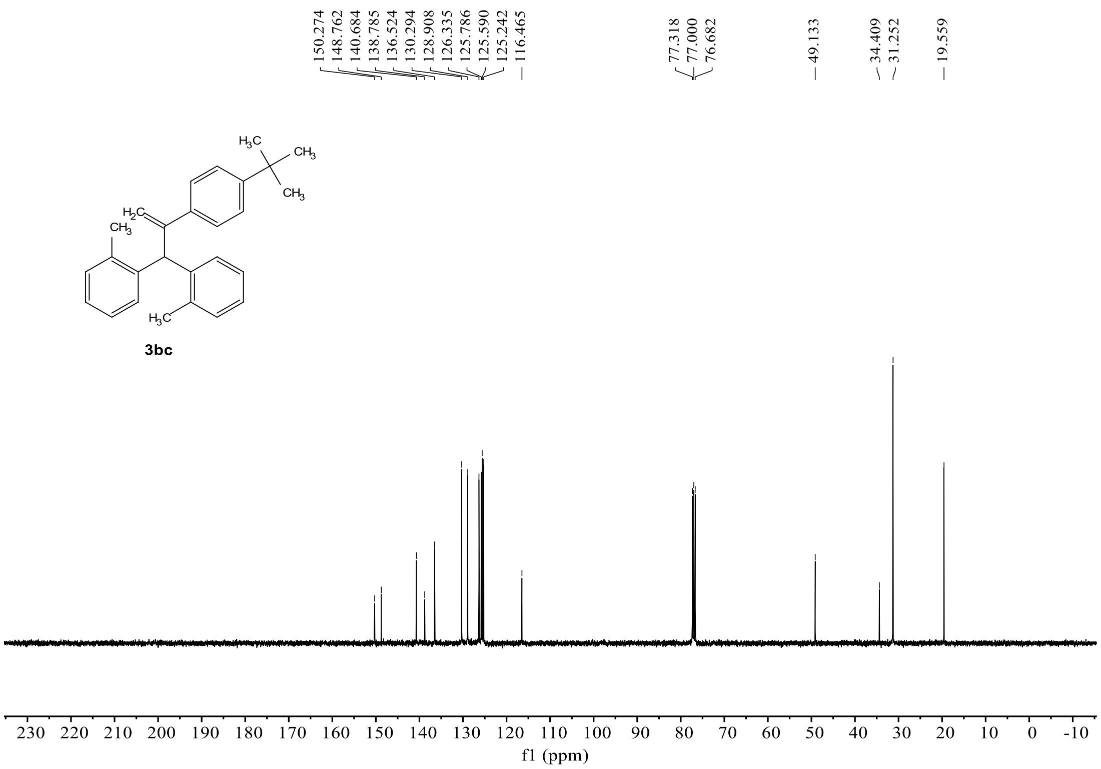

Figure S62. ^{1}H NMR ($CDCl_3$, 400 MHz) and ^{13}C NMR ($CDCl_3$, 100 MHz) spectra of compound **3bc**

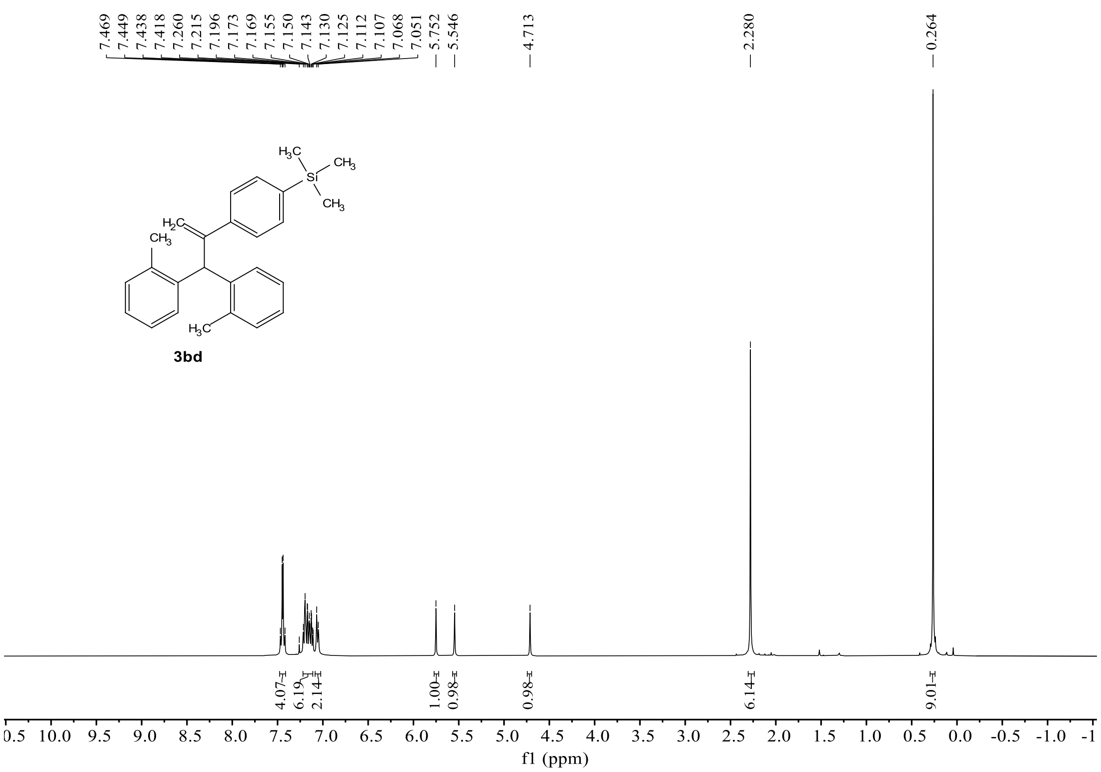

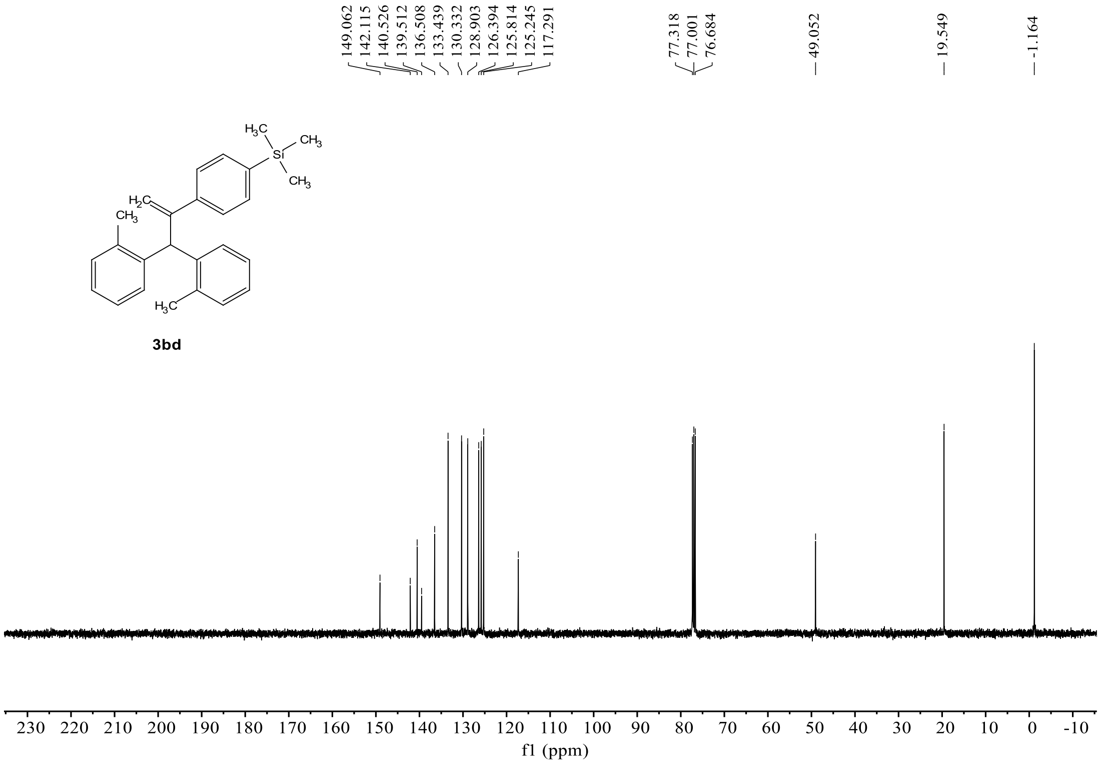

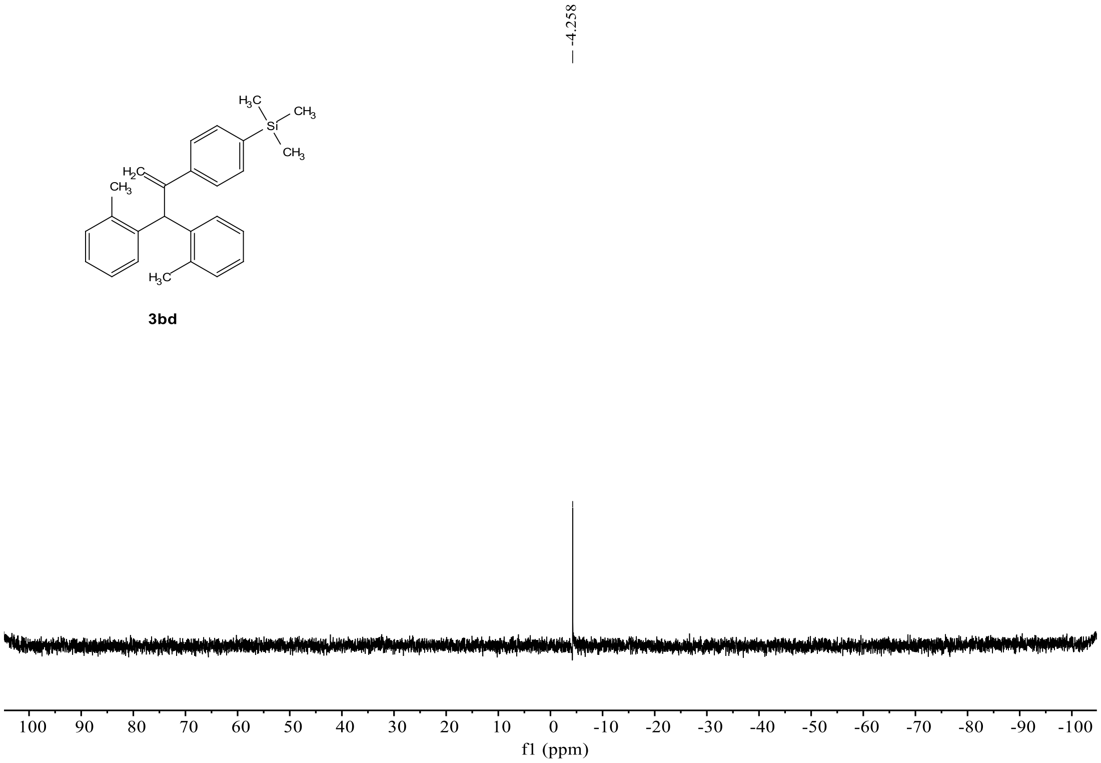

Figure S63. ^{1}H NMR ($CDCl_3$, 400 MHz), ^{13}C NMR ($CDCl_3$, 100 MHz), ^{29}Si NMR (80 MHz, $CDCl_3$) spectra of compound **3bd**

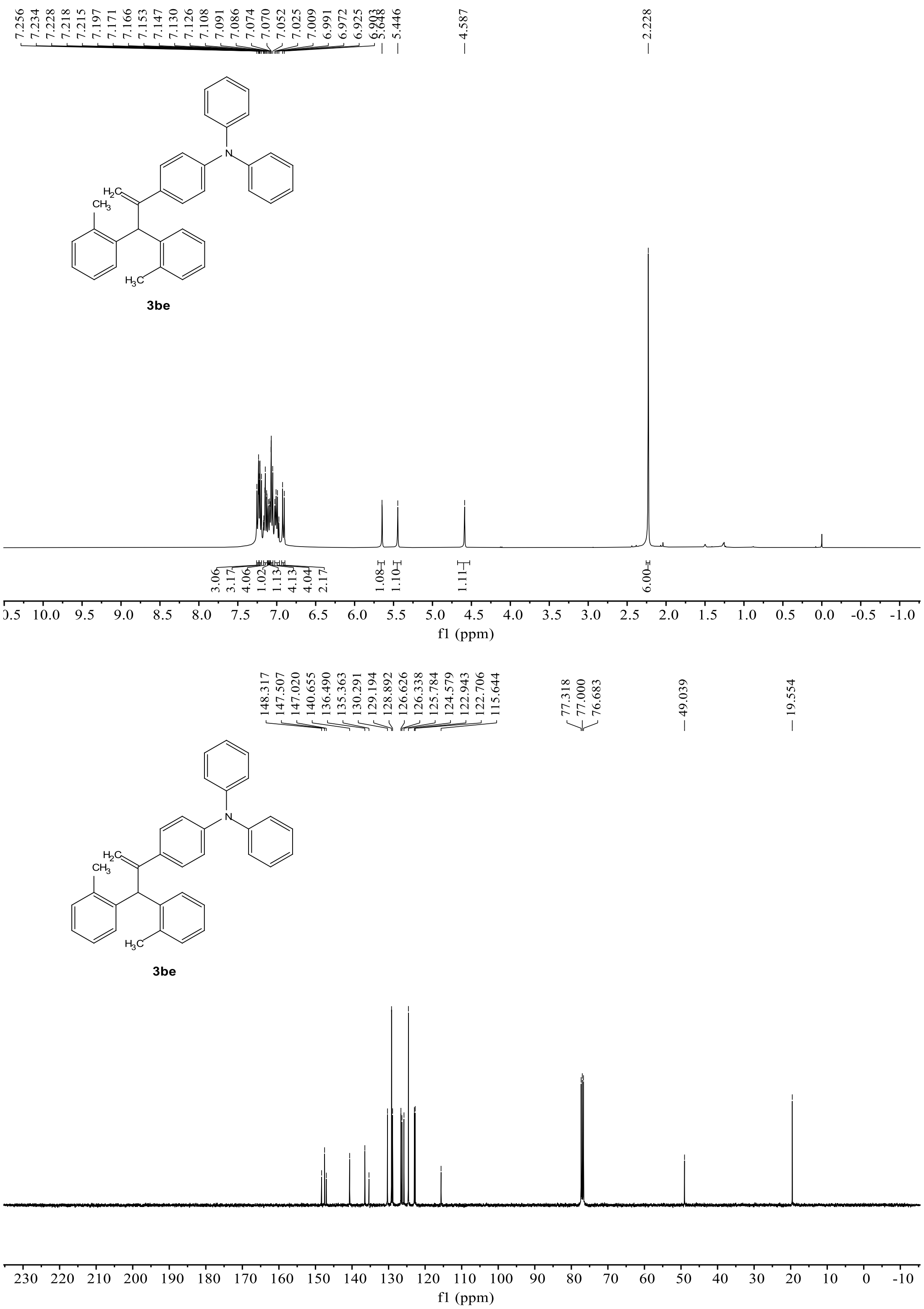

Figure S64. ^{1}H NMR ($CDCl_3$, 400 MHz) and ^{13}C NMR ($CDCl_3$, 100 MHz) spectra of compound **3be**

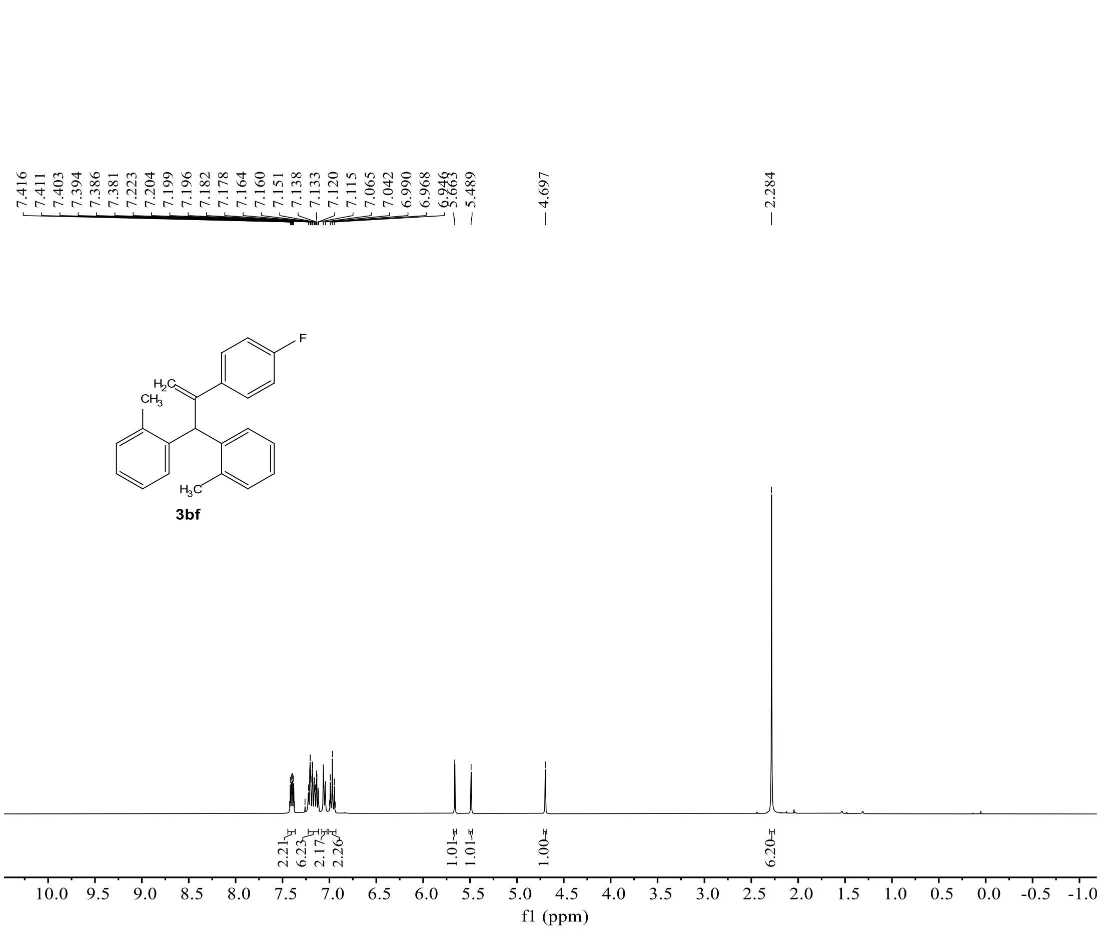
7.416
7.411
7.403
7.394
7.386
7.381
7.223
7.204
7.199
7.196
7.182
7.178
7.164
7.160
7.151
7.138
7.133
7.120
7.115
7.065
7.042
6.990
6.968
6.946
5.663
5.489
4.697
2.284
F
H2C
CH3
H3C
3bf
2.21
6.23
2.17
2.26
1.01
1.01
1.00
6.20
10.0 9.5 9.0 8.5 8.0 7.5 7.0 6.5 6.0 5.5 5.0 4.5 4.0 3.5 3.0 2.5 2.0 1.5 1.0 0.5 0.0 -0.5 -1.0
f1 (ppm)

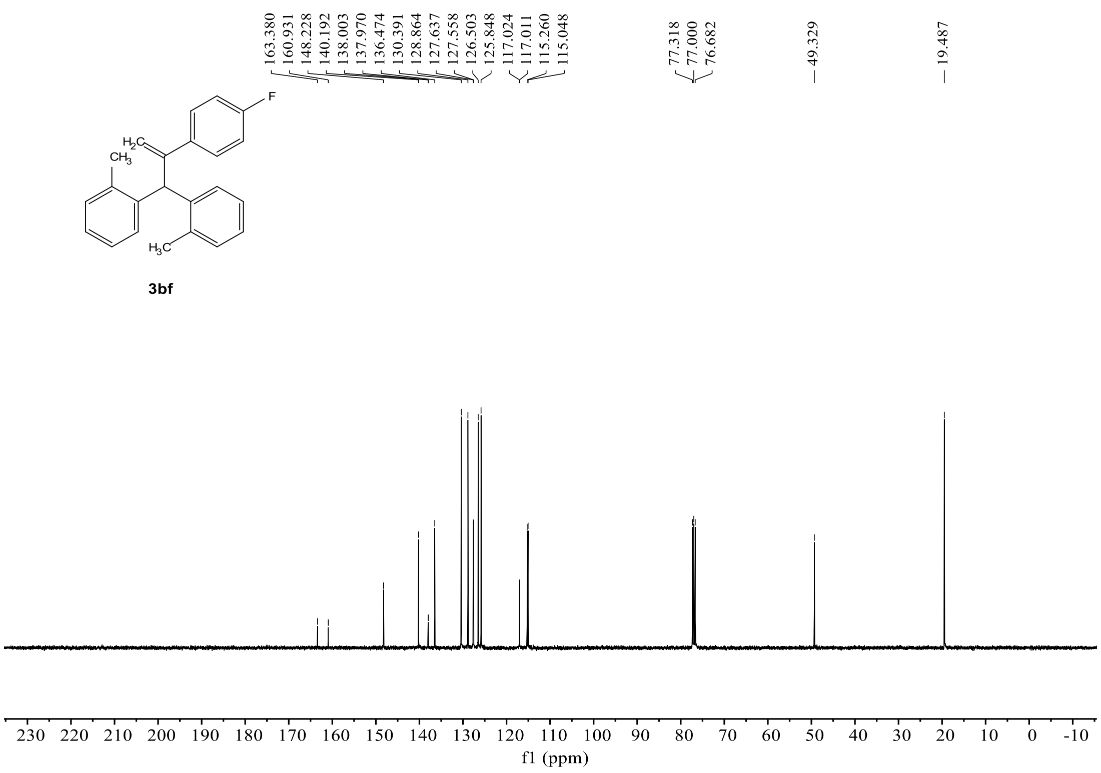
163.380
160.931
148.228
140.192
138.003
137.970
136.474
130.391
128.864
127.637
127.558
126.503
125.848
117.024
117.011
115.260
115.048
77.318
77.000
76.682
49.329
19.487
F
H2C
CH3
H3C
3bf
230 220 210 200 190 180 170 160 150 140 130 120 110 100 90 80 70 60 50 40 30 20 10 0 -10
f1 (ppm)

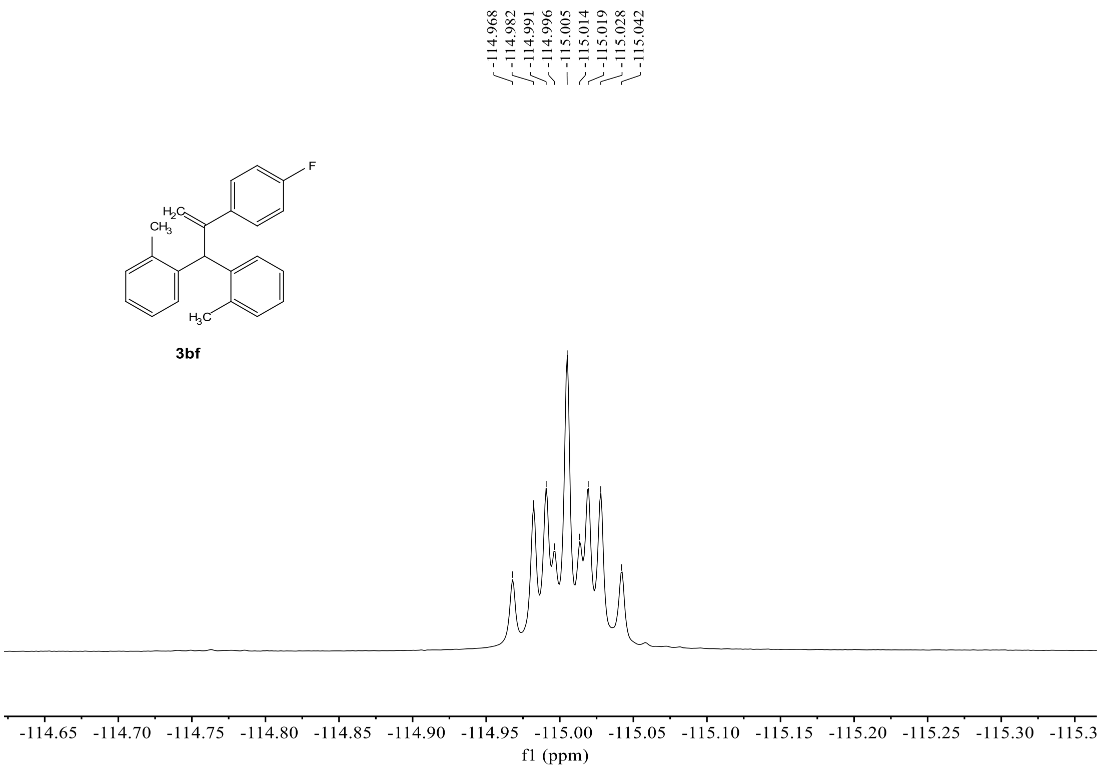

Figure S65. ^{1}H NMR ($CDCl_3$, 400 MHz), ^{13}C NMR ($CDCl_3$, 100 MHz), ^{19}F NMR ($CDCl_3$, 376 MHz) spectra of compound **3bf**

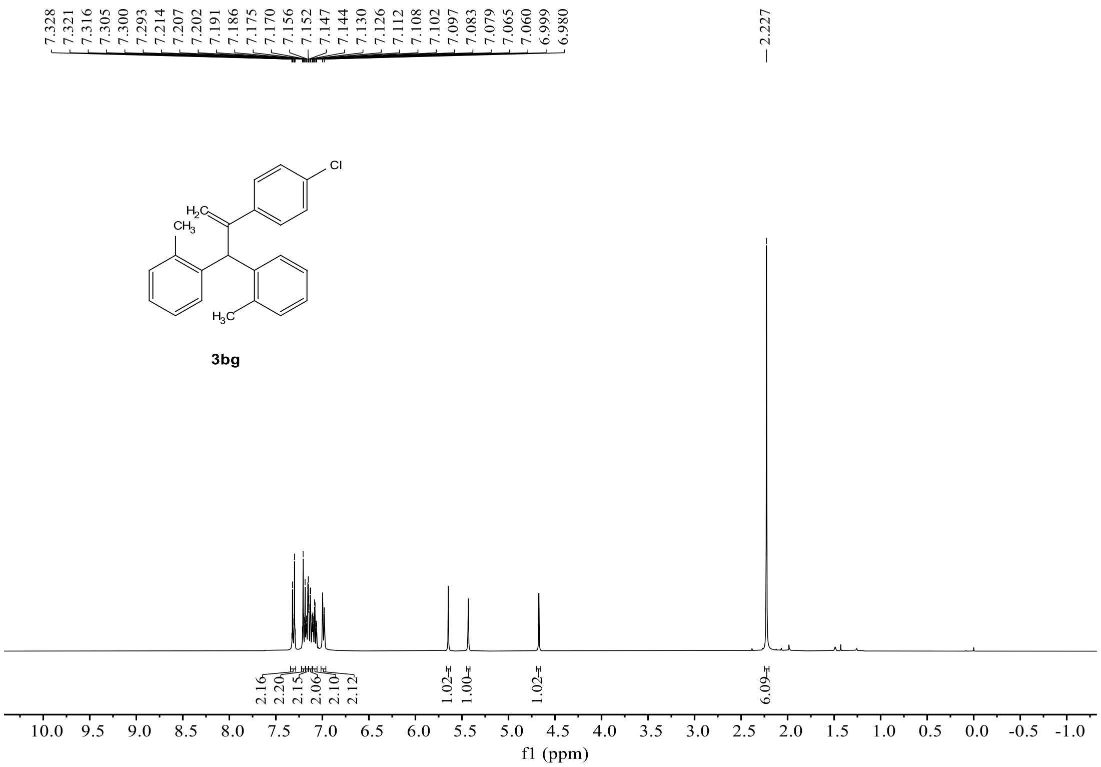

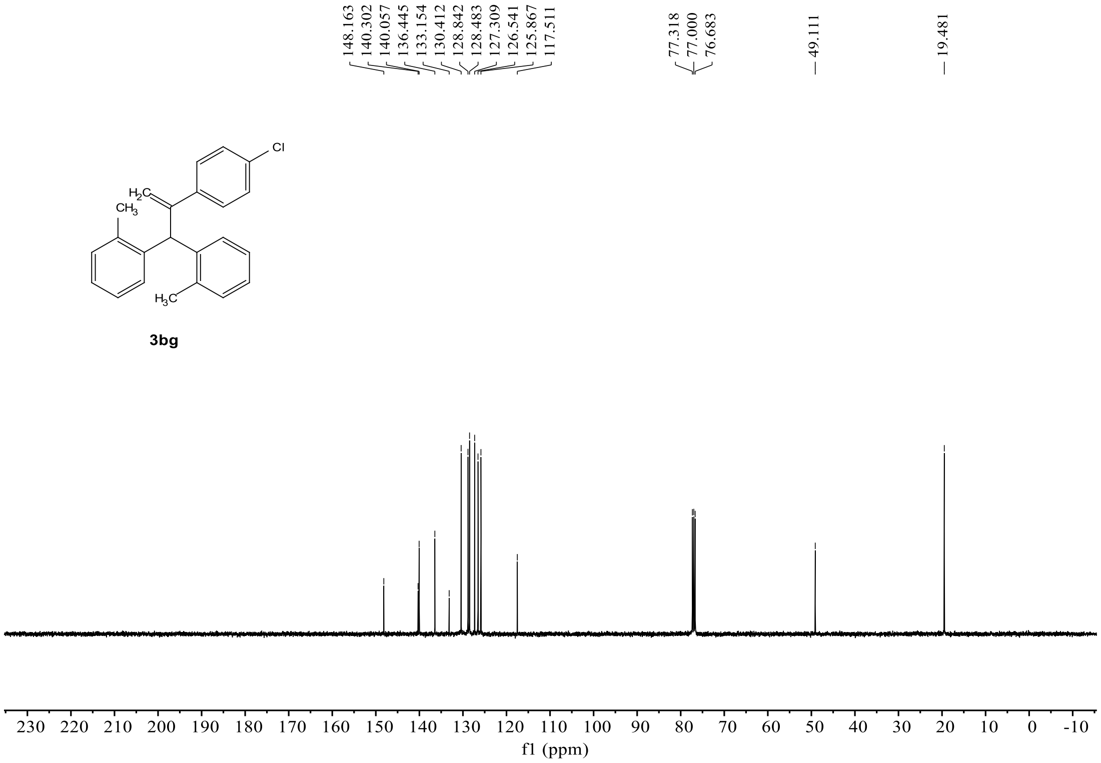

Figure S66. ^{1}H NMR ($CDCl_3$, 400 MHz) and ^{13}C NMR ($CDCl_3$, 100 MHz) spectra of compound **3bg**

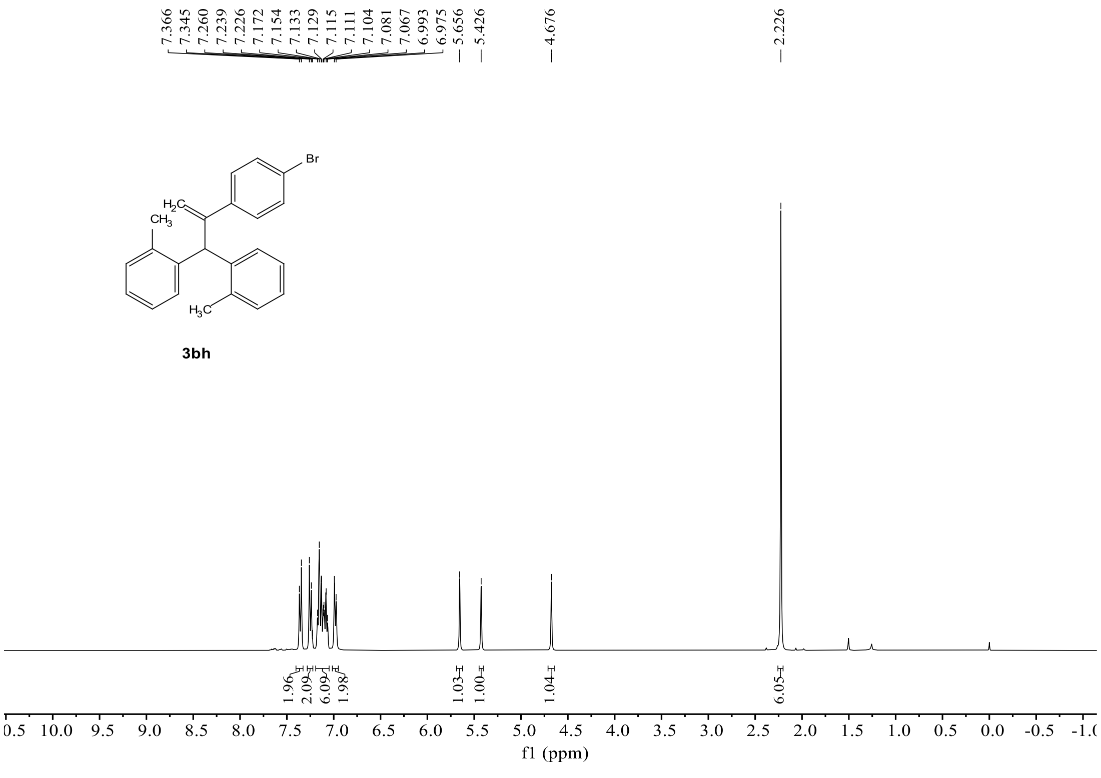

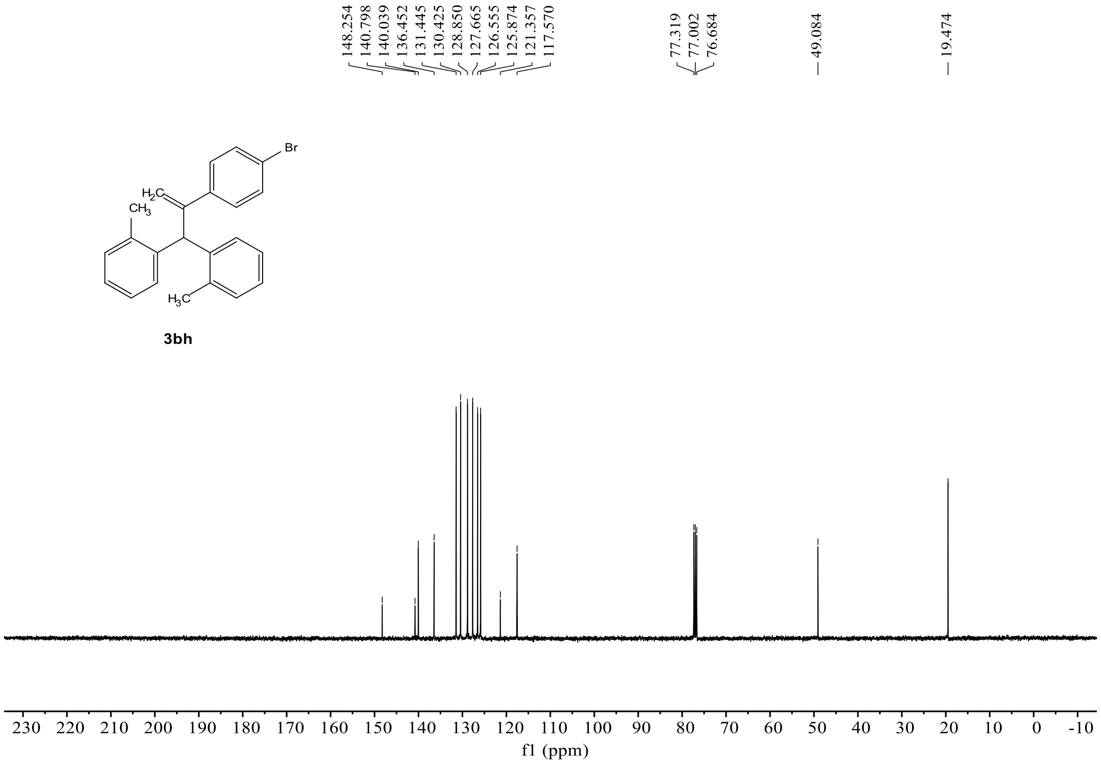

Figure S67. ^{1}H NMR ($CDCl_3$, 400 MHz) and ^{13}C NMR ($CDCl_3$, 100 MHz) spectra of compound **3bh**

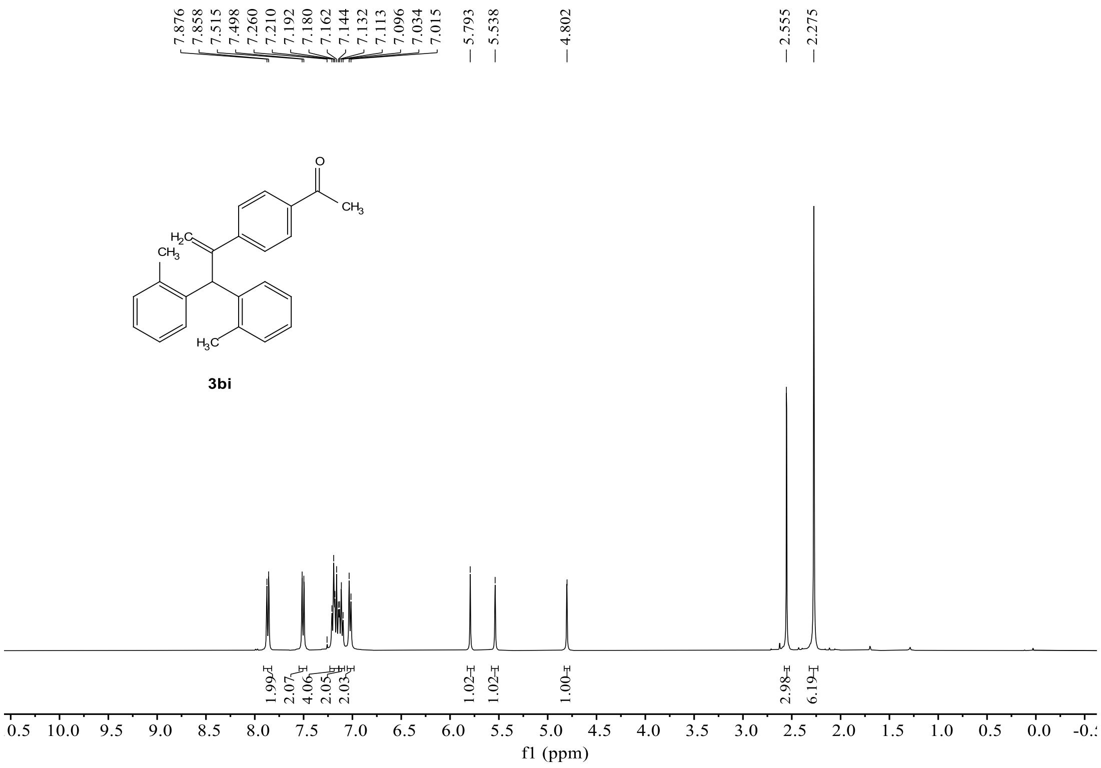

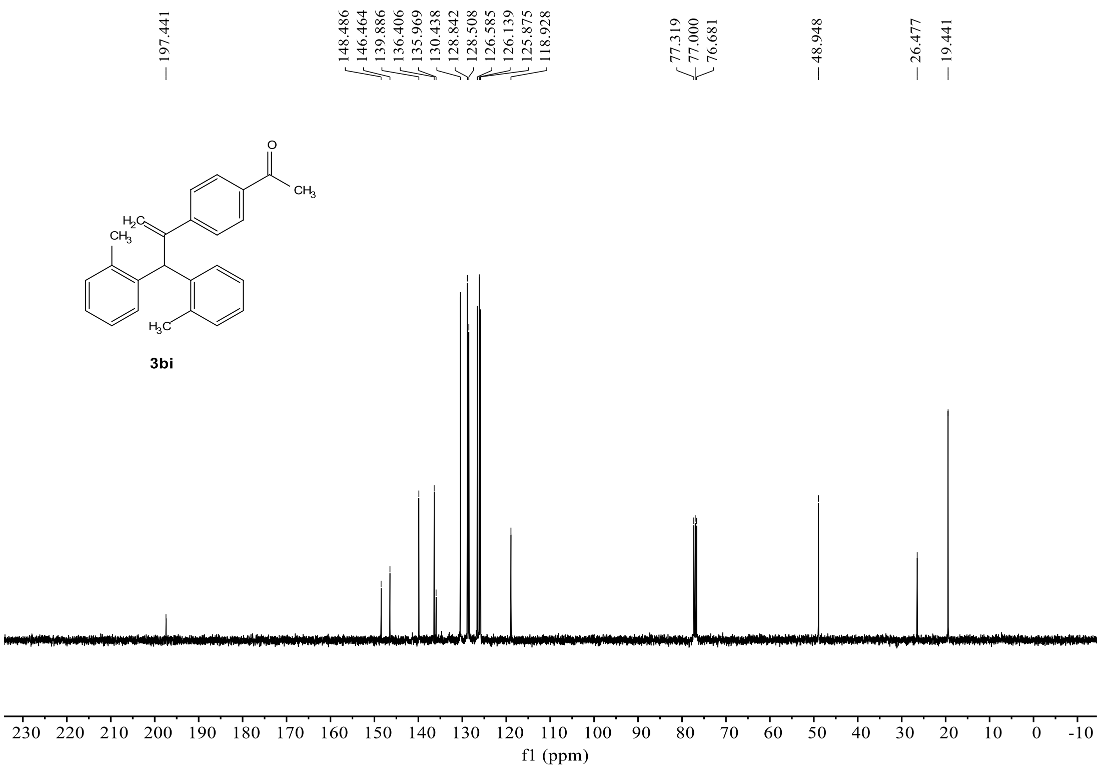

Figure S68. ^{1}H NMR ($CDCl_3$, 400 MHz) and ^{13}C NMR ($CDCl_3$, 100 MHz) spectra of compound **3bi**

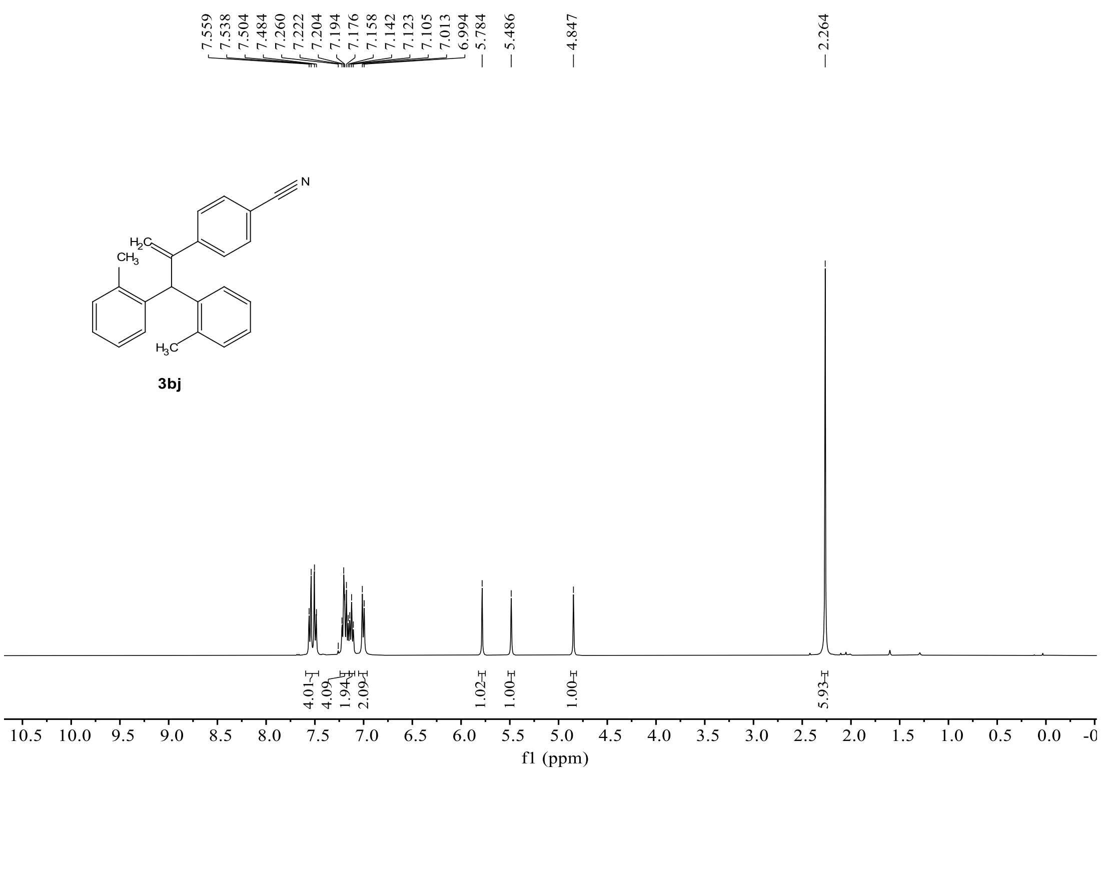

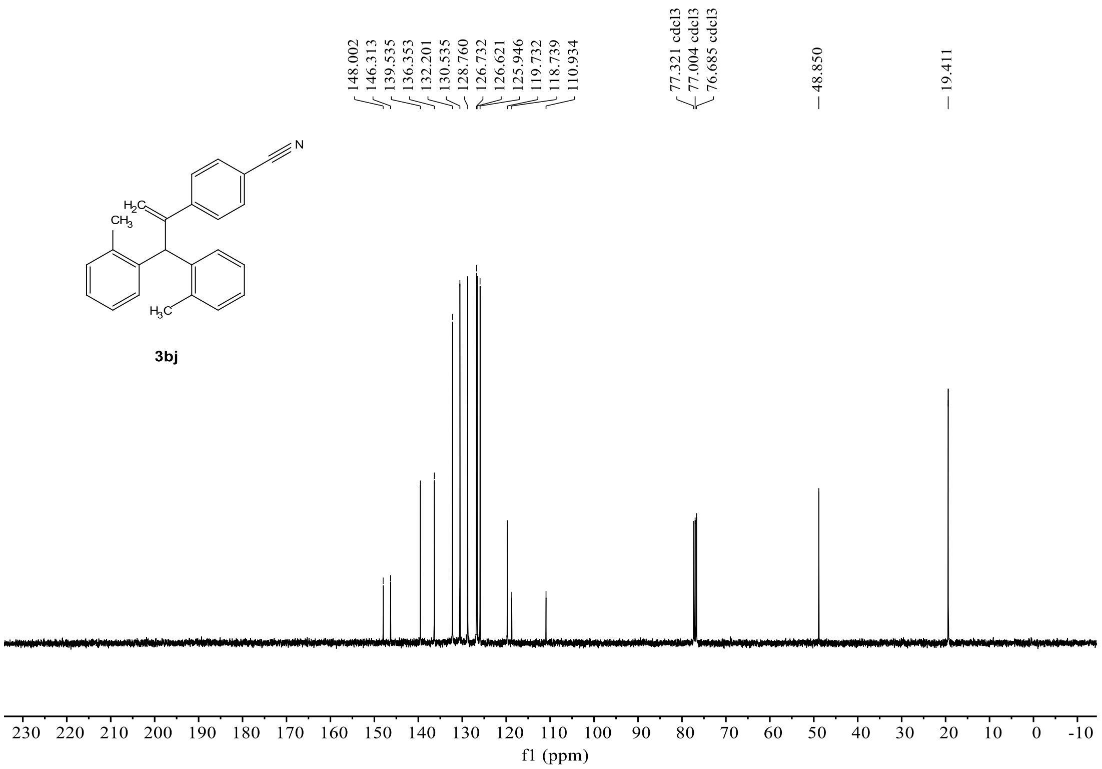

Figure S69. ^{1}H NMR ($CDCl_3$, 400 MHz) and ^{13}C NMR ($CDCl_3$, 100 MHz) spectra of compound **3bj**

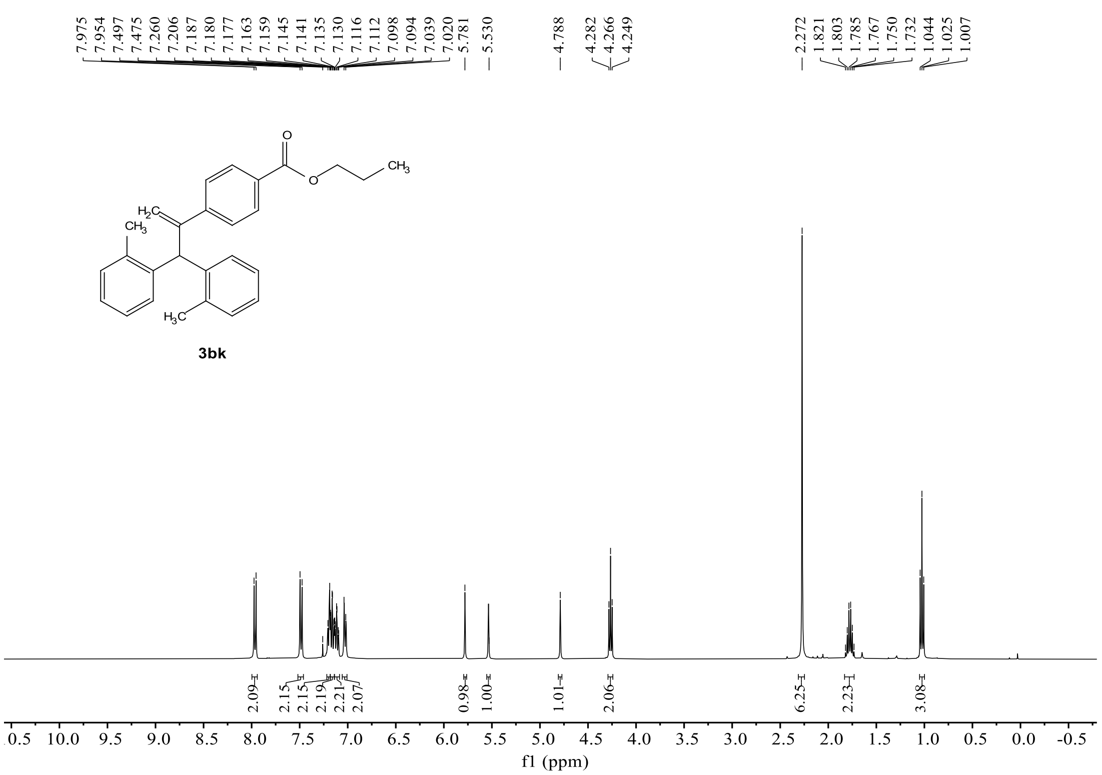

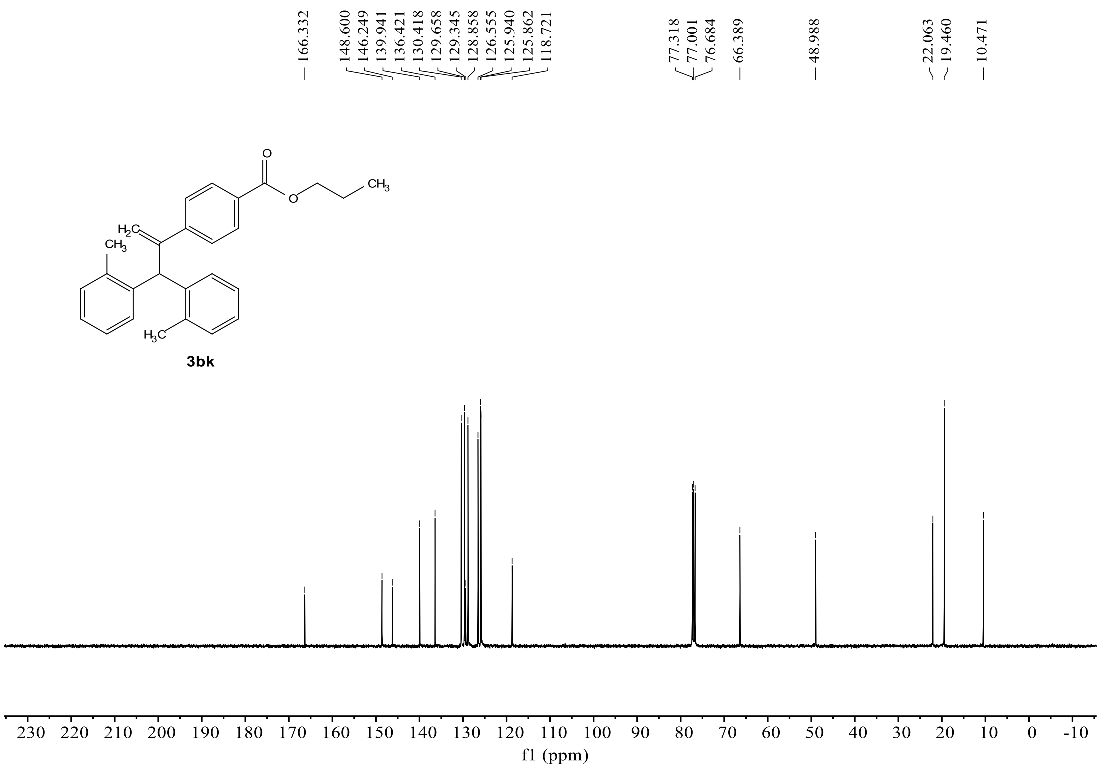

Figure S70. ^{1}H NMR ($CDCl_3$, 400 MHz) and ^{13}C NMR ($CDCl_3$, 100 MHz) spectra of compound **3bk**

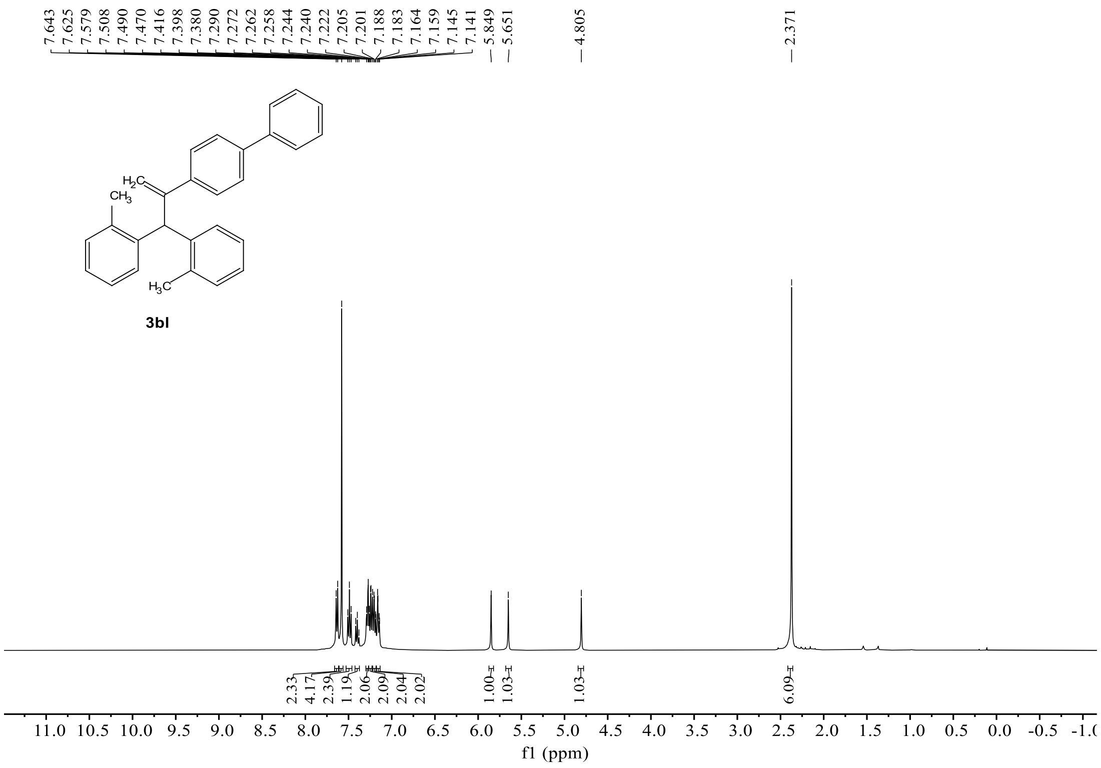

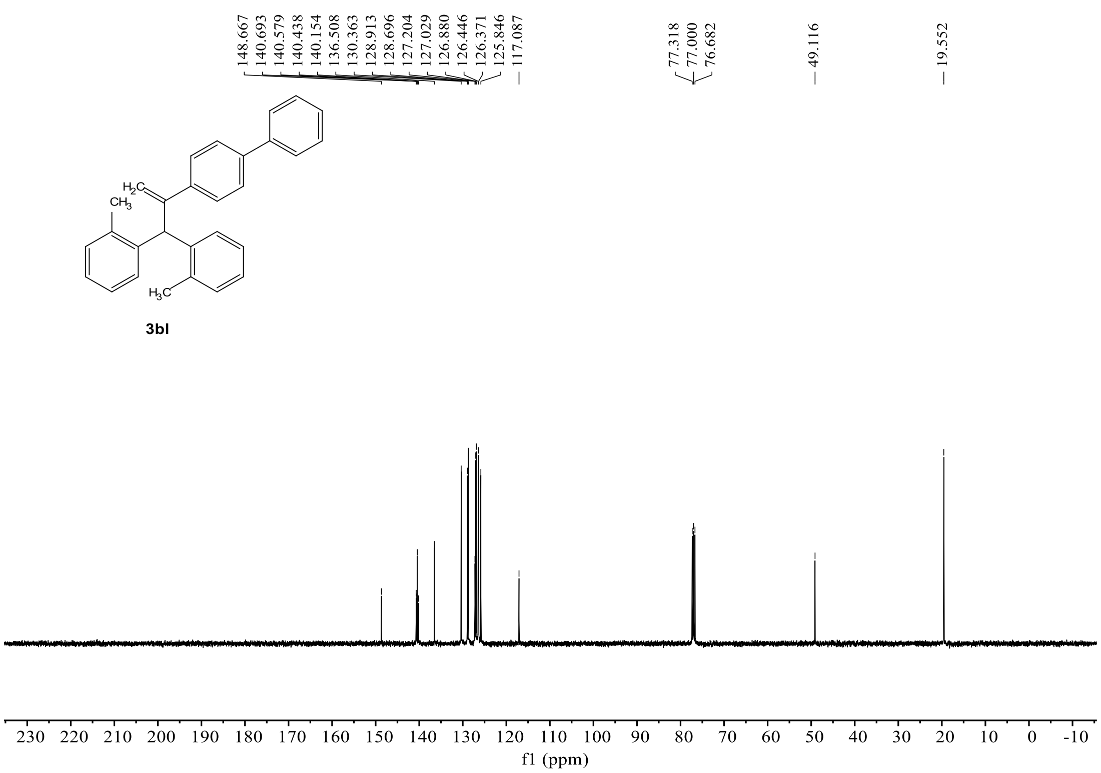

Figure S71. ^{1}H NMR ($CDCl_3$, 400 MHz) and ^{13}C NMR ($CDCl_3$, 100 MHz) spectra of compound **3bl**

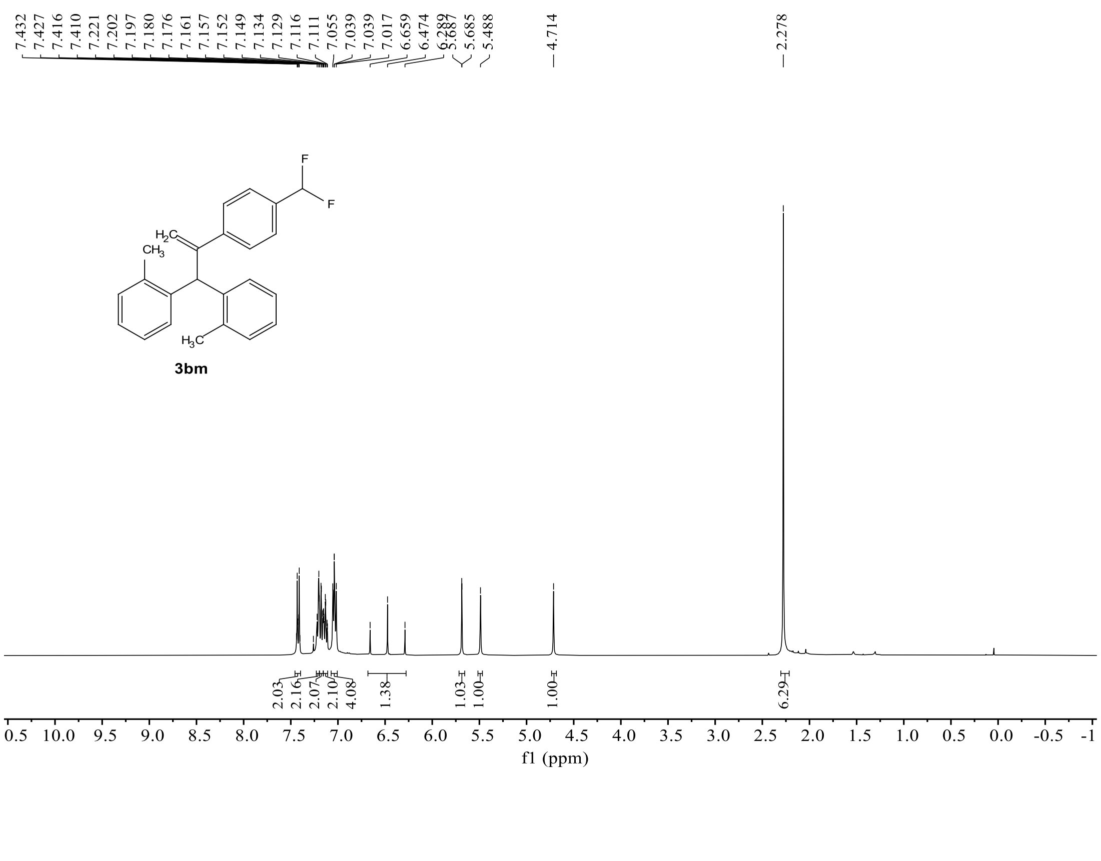

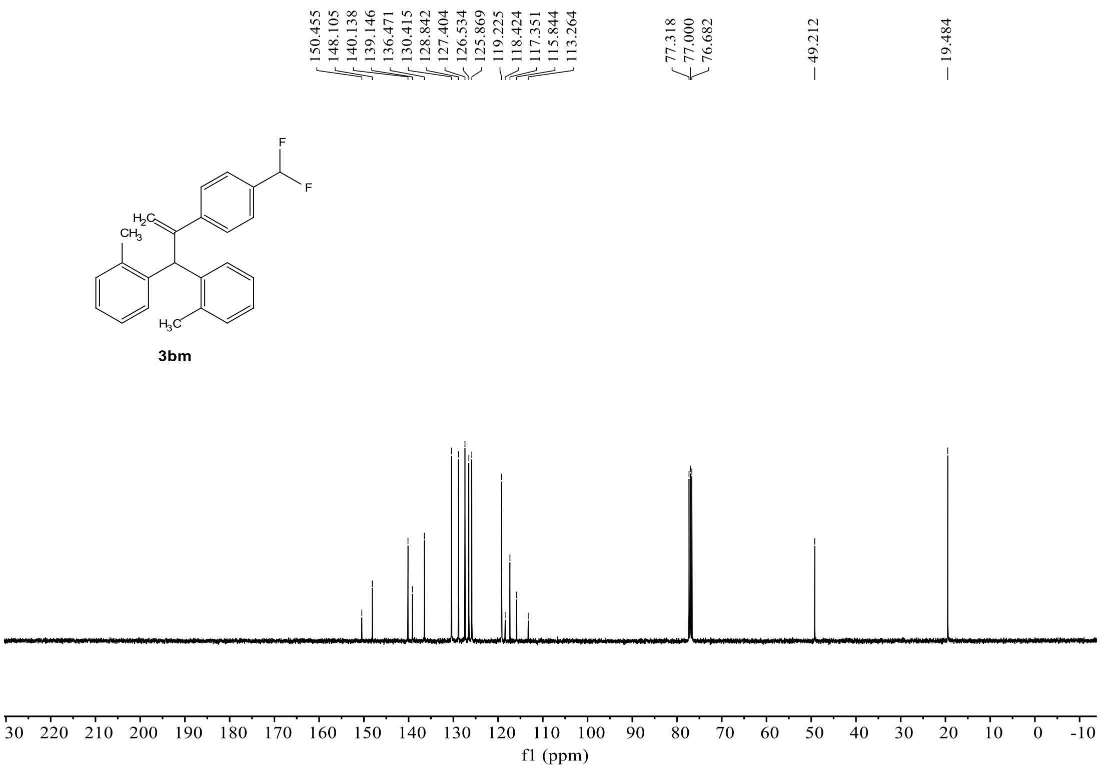

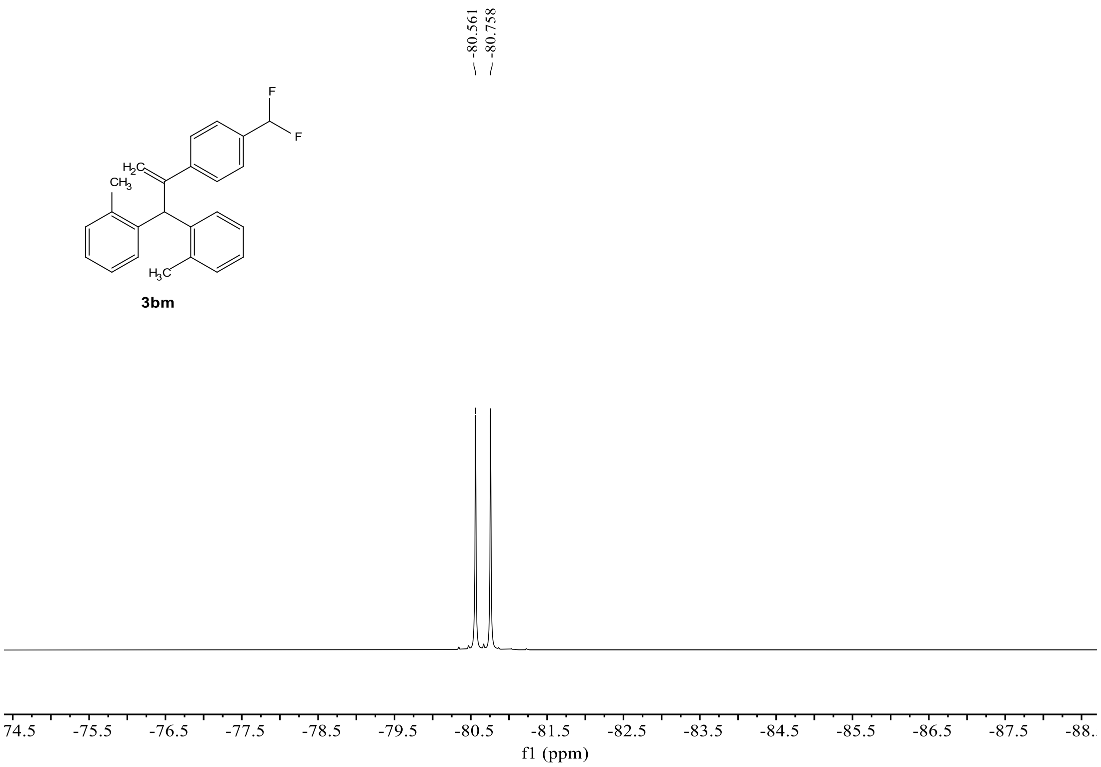

Figure S72. ^{1}H NMR ($CDCl_3$, 400 MHz), ^{13}C NMR ($CDCl_3$, 100 MHz), ^{19}F NMR ($CDCl_3$, 376 MHz) spectra of compound **3bm**

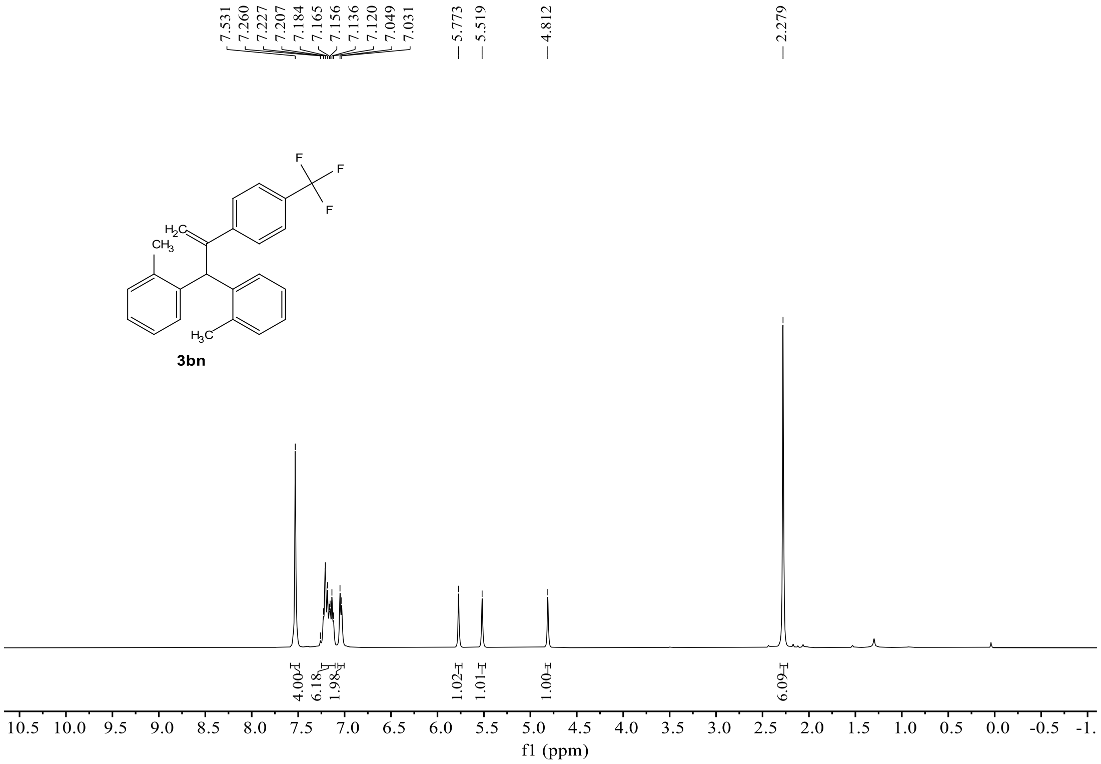
7.531
7.260
7.227
7.207
7.184
7.165
7.156
7.136
7.120
7.049
7.031
5.773
5.519
4.812
2.279
F
F
F
H_2C
CH_3
H_3C
3bn
4.00
6.18
1.98
1.02
1.01
1.00
6.09
10.5 10.0 9.5 9.0 8.5 8.0 7.5 7.0 6.5 6.0 5.5 5.0 4.5 4.0 3.5 3.0 2.5 2.0 1.5 1.0 0.5 0.0 -0.5 -1.
f1 (ppm)

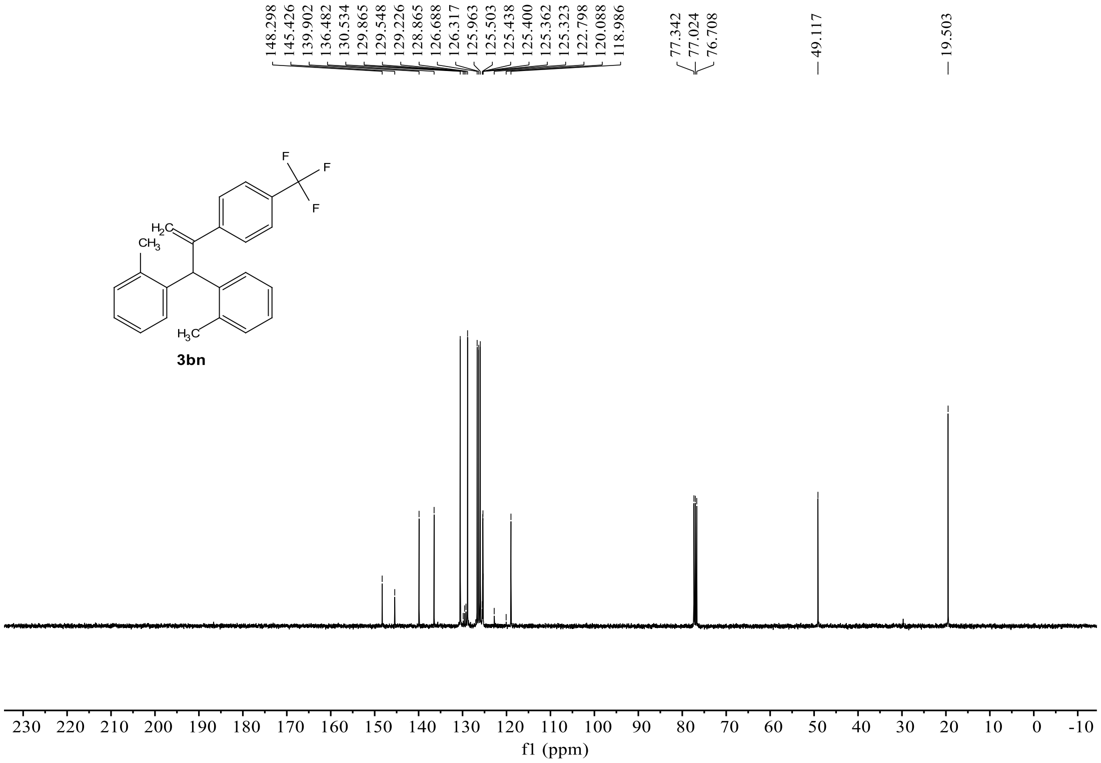
148.298
145.426
139.902
136.482
130.534
129.865
129.548
129.226
128.865
126.688
126.317
125.963
125.503
125.438
125.400
125.362
125.323
122.798
120.088
118.986
77.342
77.024
76.708
49.117
19.503
F
F
F
H_2C
CH_3
H_3C
3bn
230 220 210 200 190 180 170 160 150 140 130 120 110 100 90 80 70 60 50 40 30 20 10 0 -10
f1 (ppm)

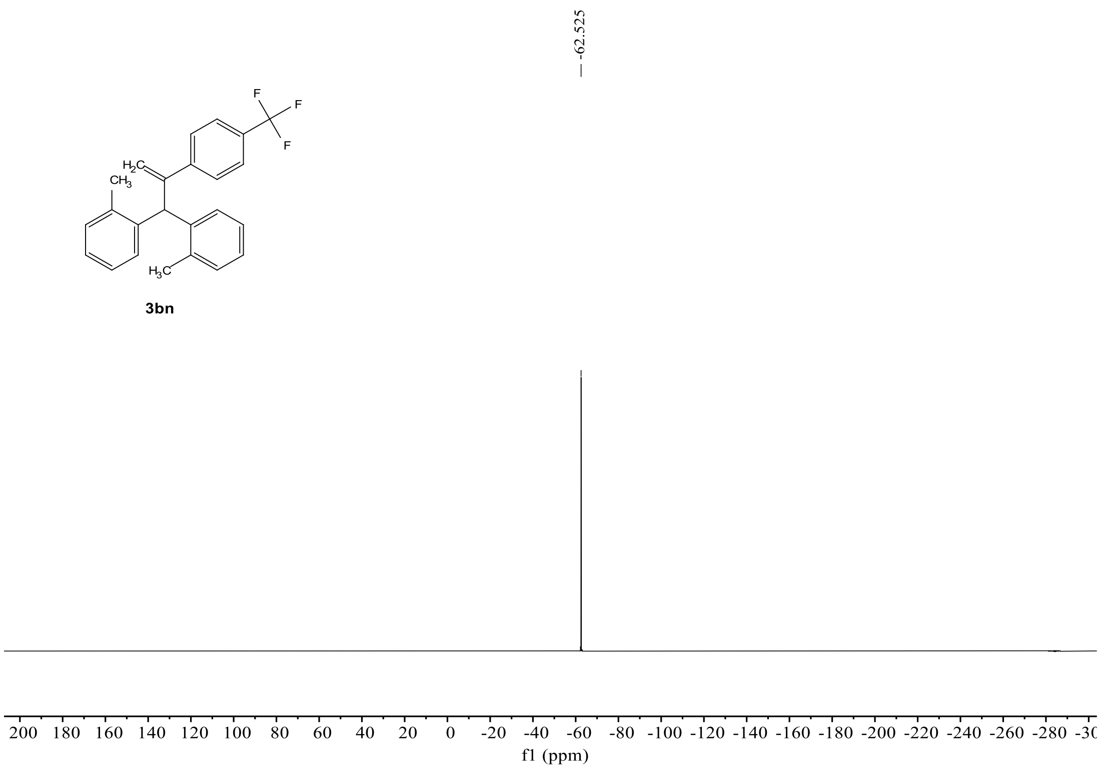

Figure S73. ^{1}H NMR ($CDCl_3$, 400 MHz), ^{13}C NMR ($CDCl_3$, 100 MHz), ^{19}F NMR ($CDCl_3$, 376 MHz) spectra of compound **3bn**

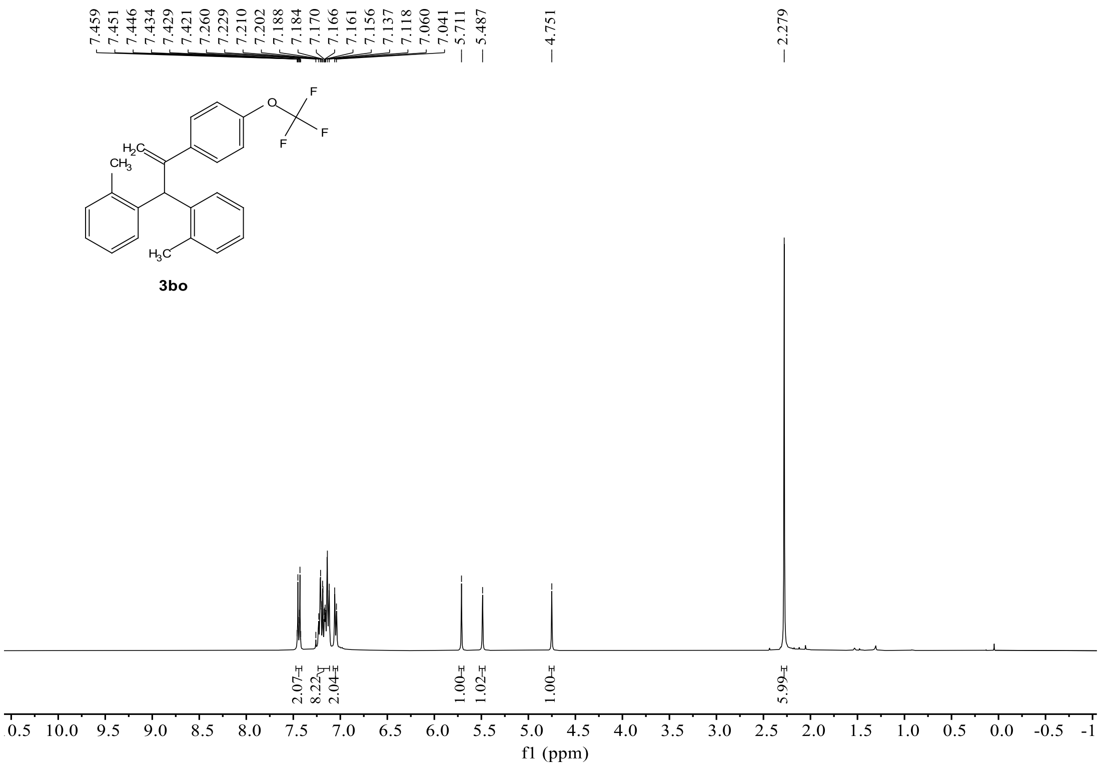

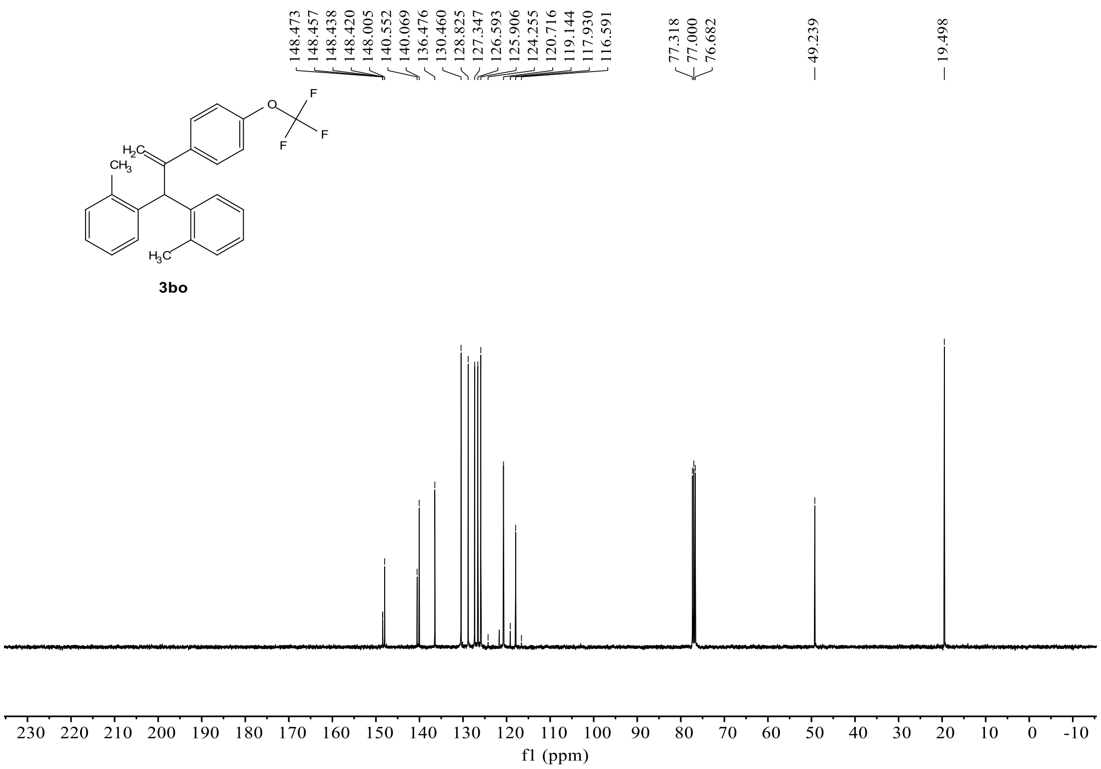

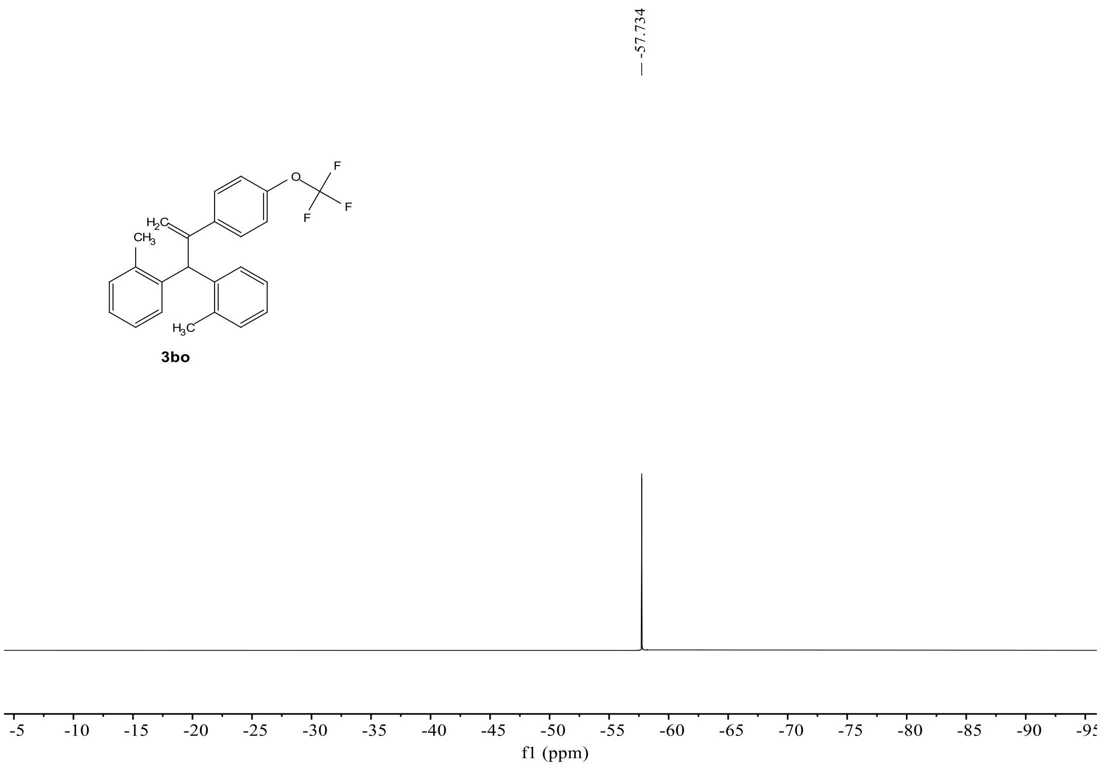

Figure S74. ^{1}H NMR ($CDCl_3$, 400 MHz), ^{13}C NMR ($CDCl_3$, 100 MHz), ^{19}F NMR ($CDCl_3$, 376 MHz) spectra of compound **3bo**

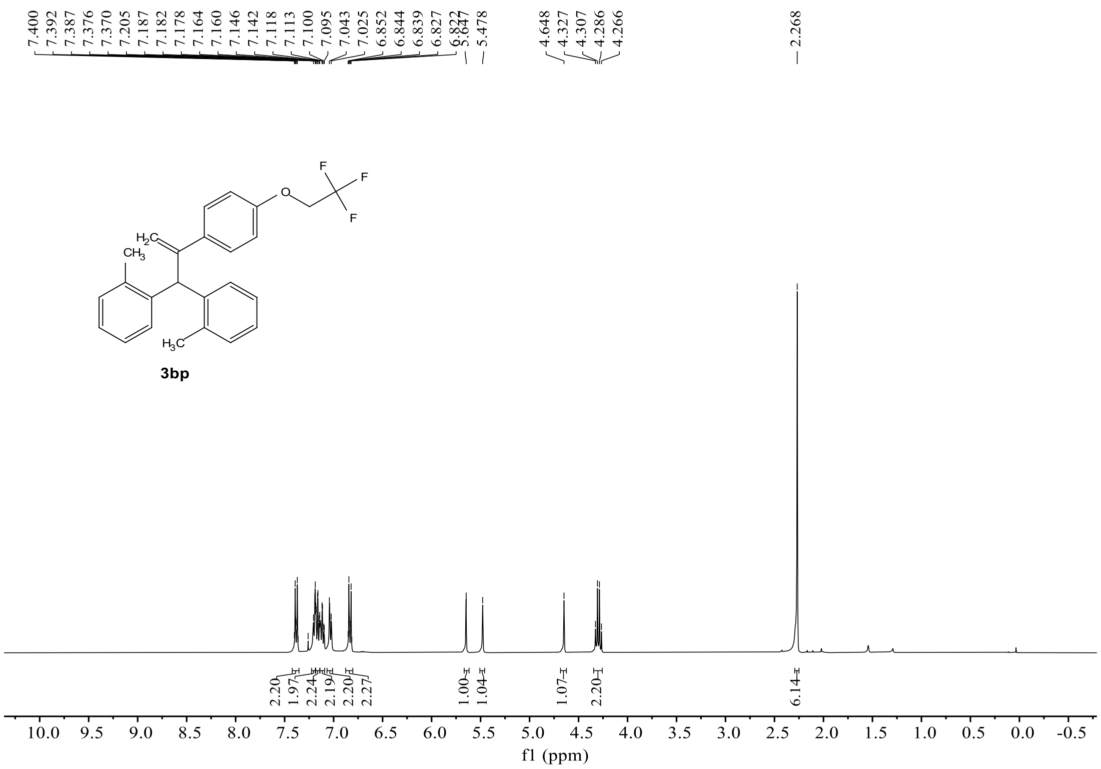

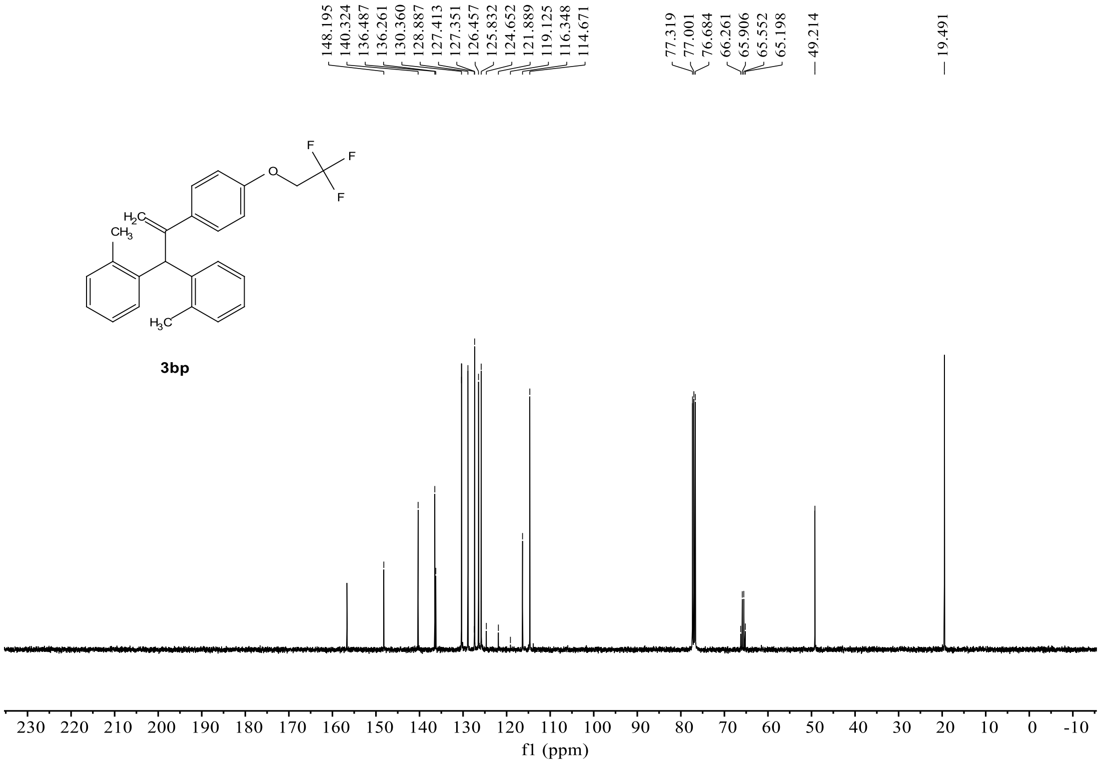

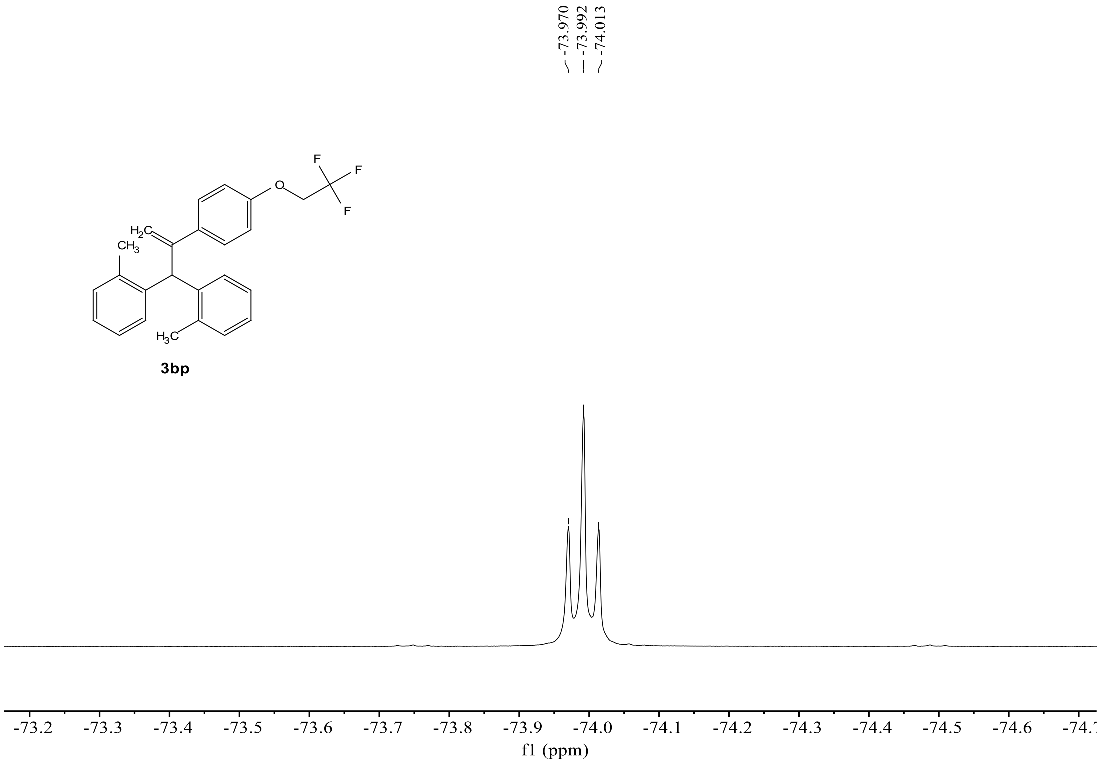

Figure S75. ^{1}H NMR ($CDCl_3$, 400 MHz), ^{13}C NMR ($CDCl_3$, 100 MHz), ^{19}F NMR ($CDCl_3$, 376 MHz) spectra of compound **3bp**

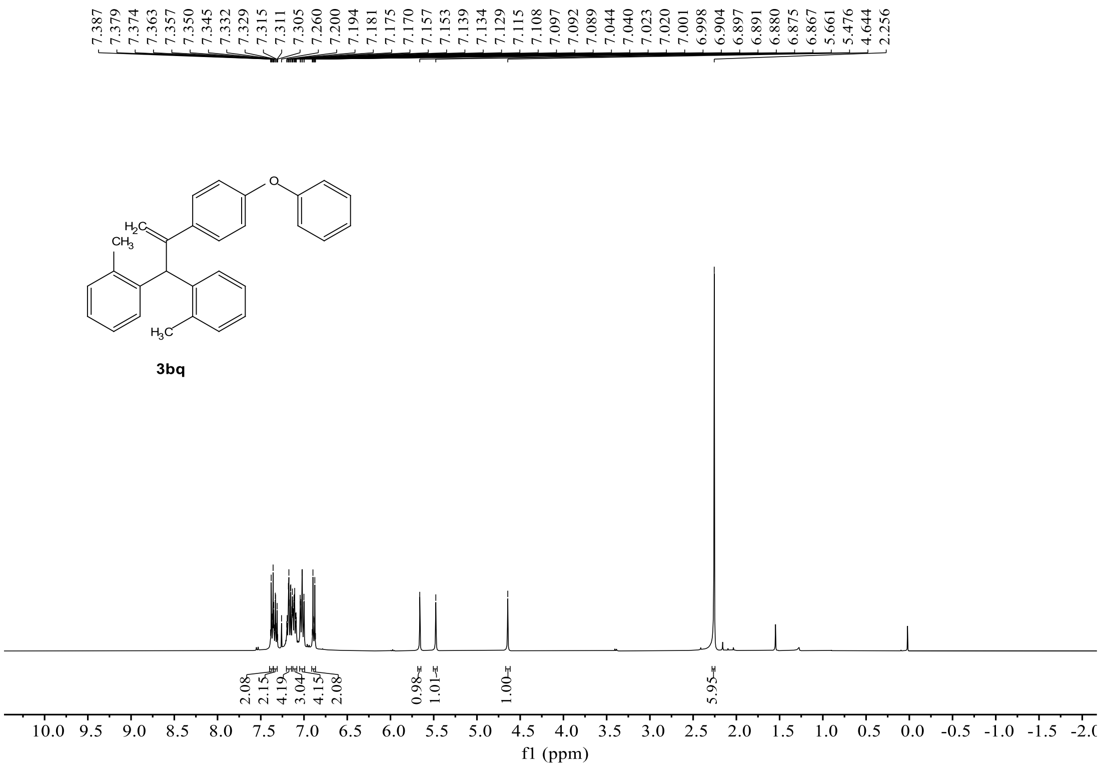

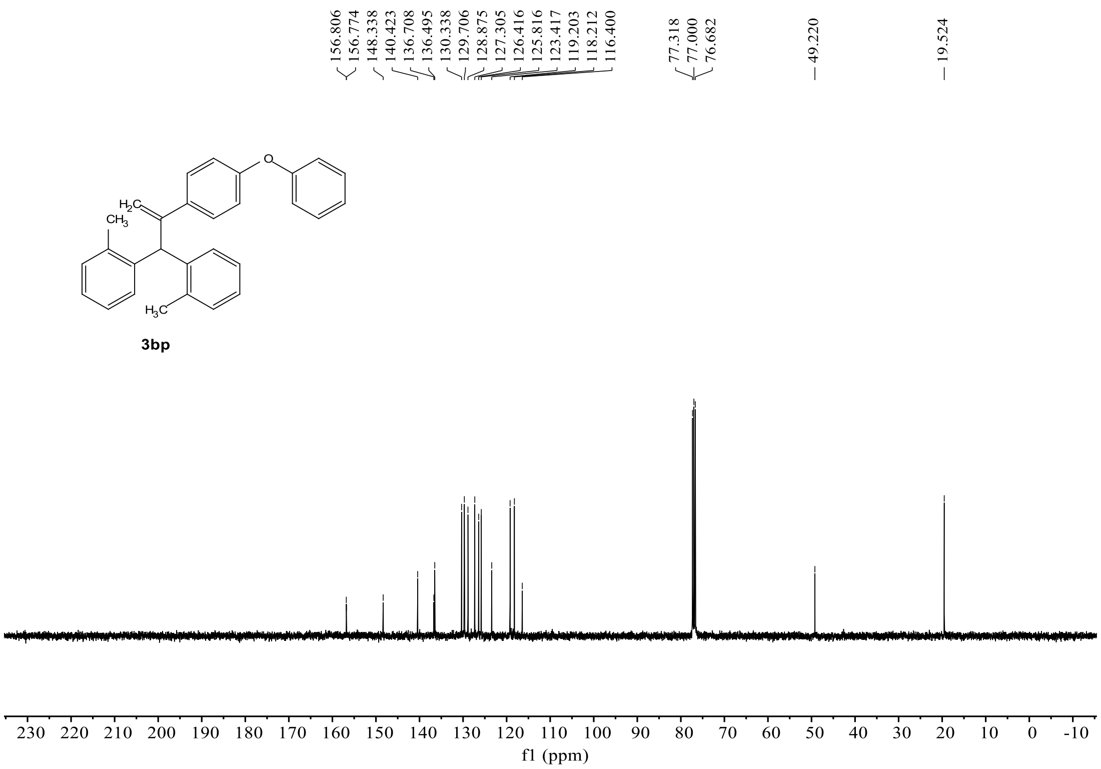

Figure S76. ^{1}H NMR ($CDCl_3$, 400 MHz) and ^{13}C NMR ($CDCl_3$, 100 MHz) spectra of compound **3bq**

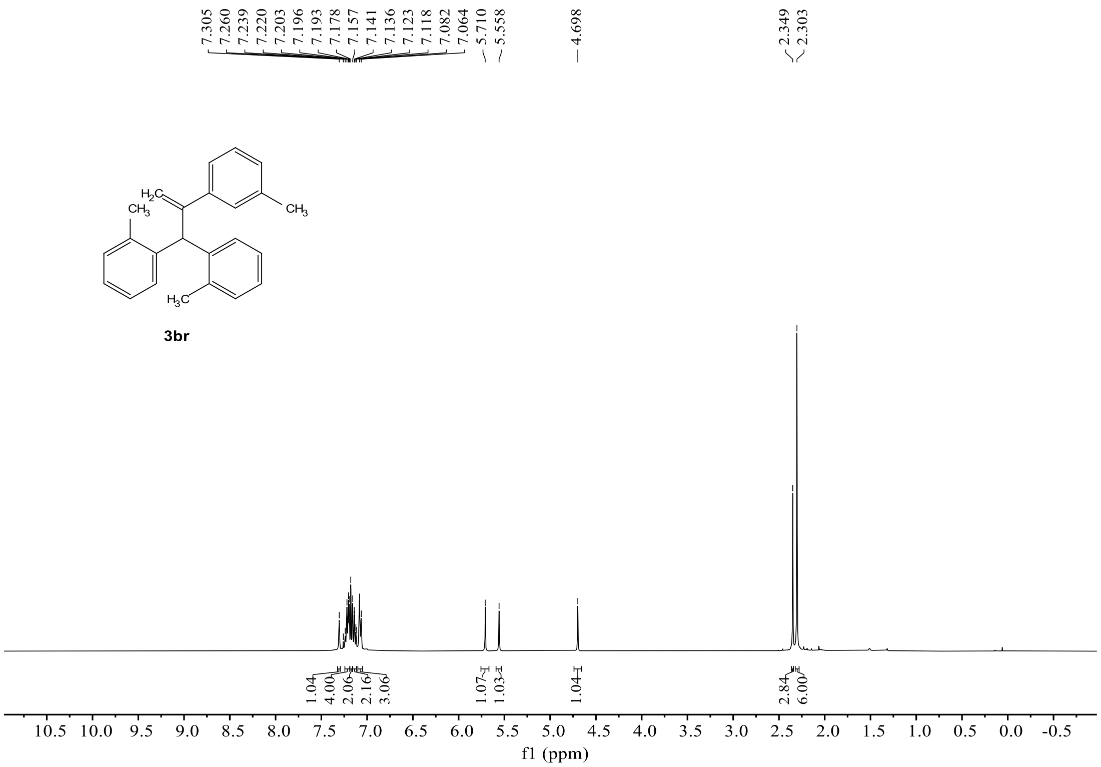

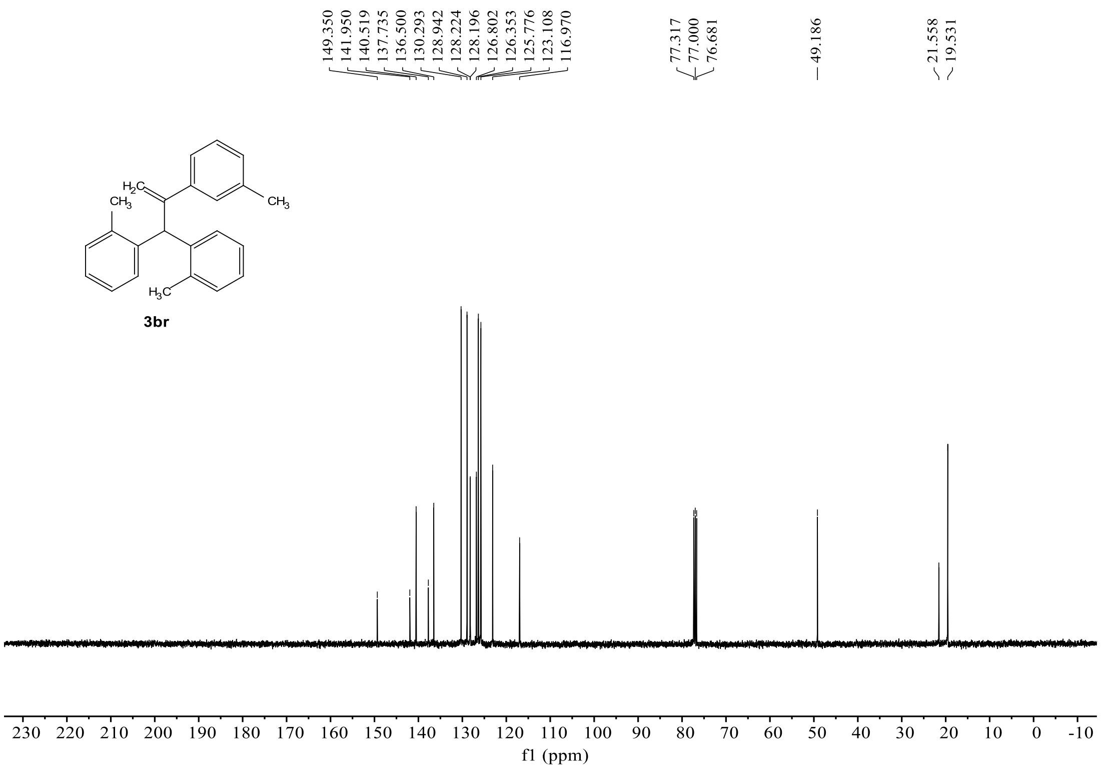

Figure S77. ^{1}H NMR ($CDCl_3$, 400 MHz) and ^{13}C NMR ($CDCl_3$, 100 MHz) spectra of compound **3br**

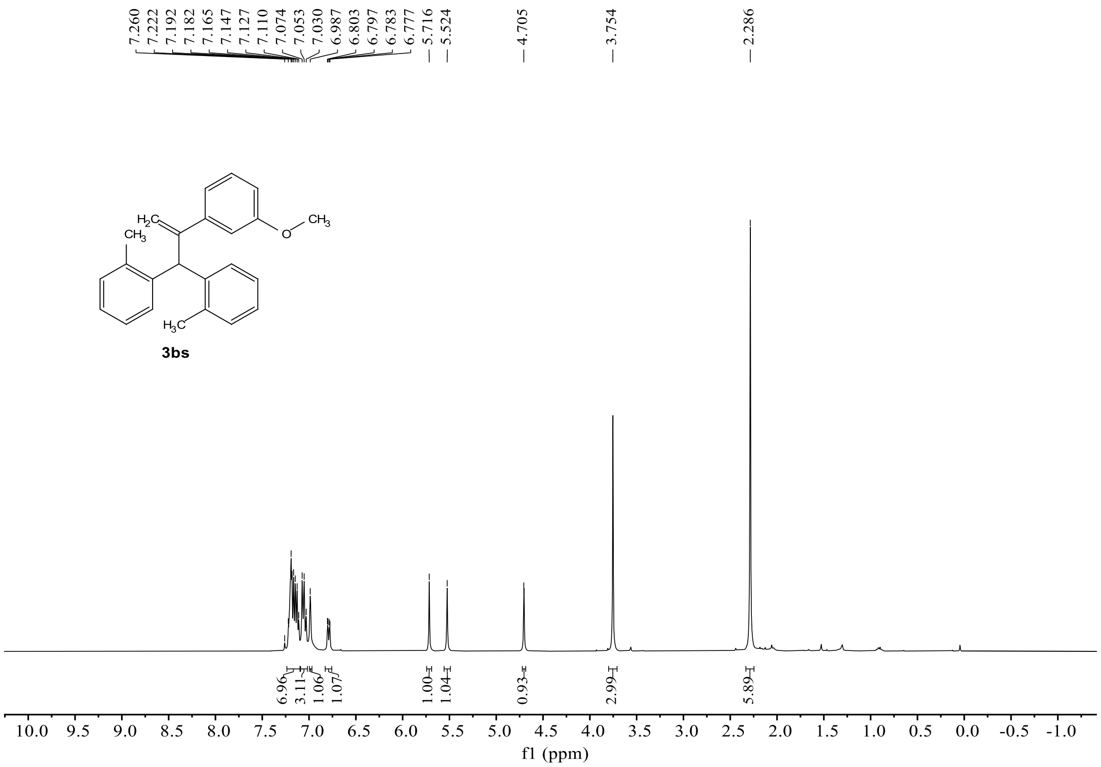

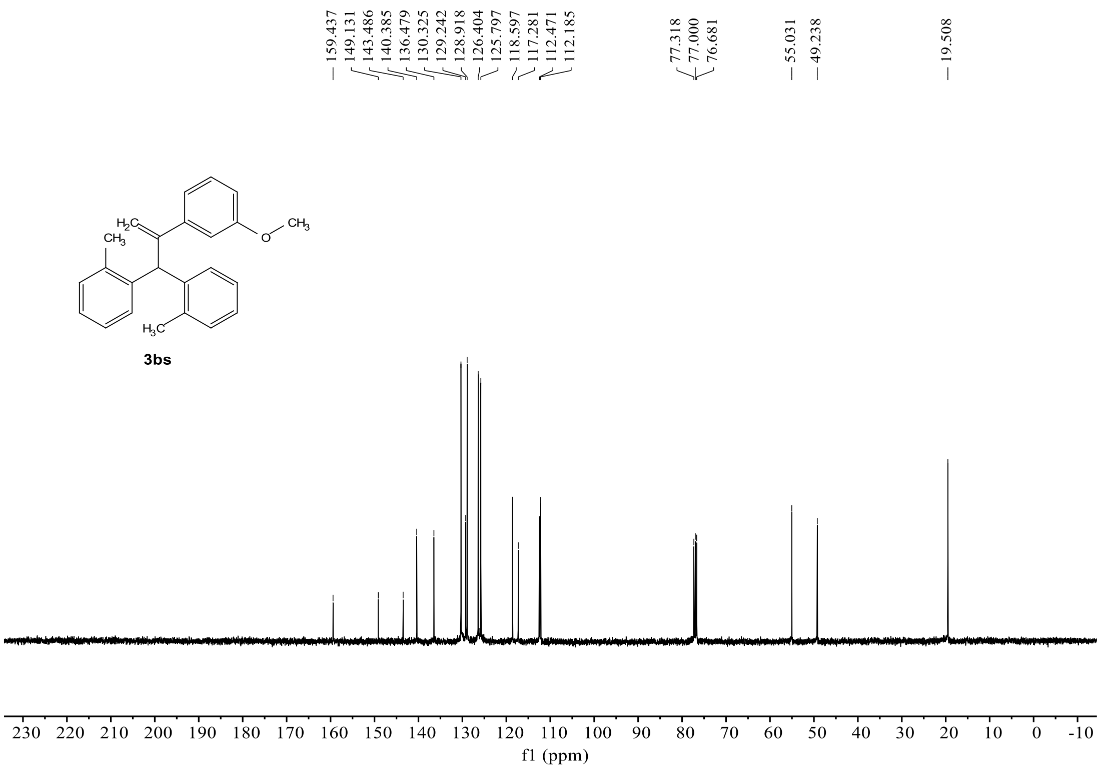

Figure S78. ^{1}H NMR ($CDCl_3$, 400 MHz) and ^{13}C NMR ($CDCl_3$, 100 MHz) spectra of compound **3bs**

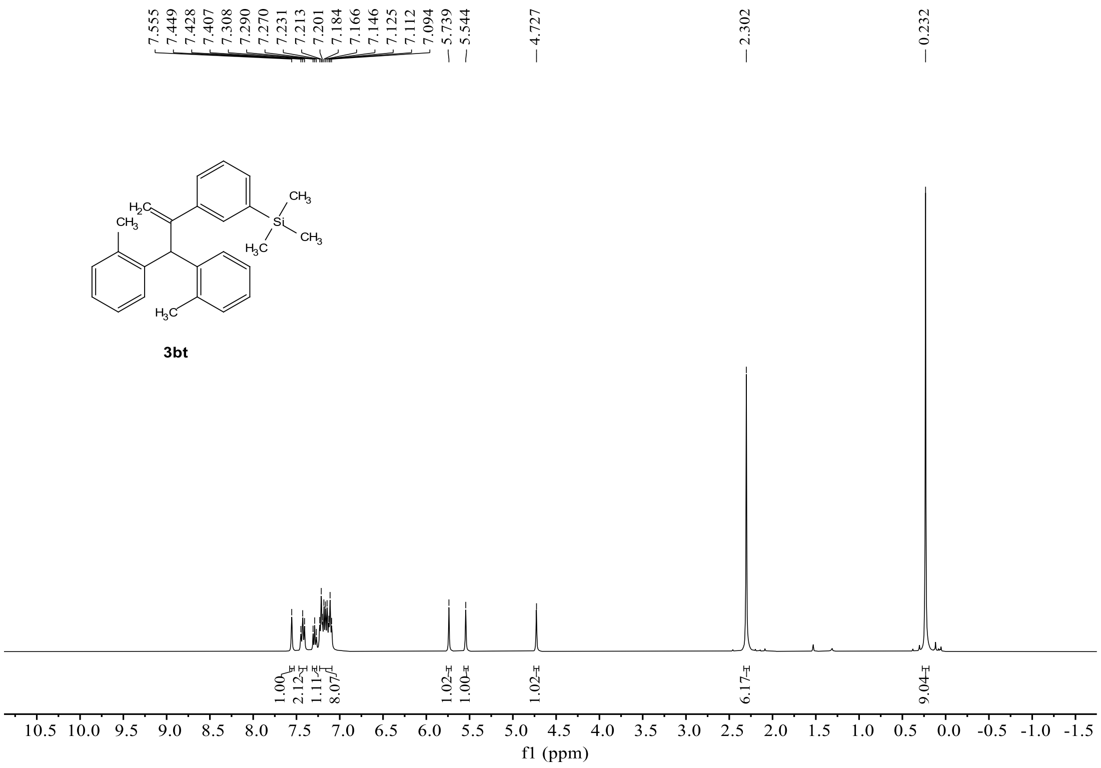

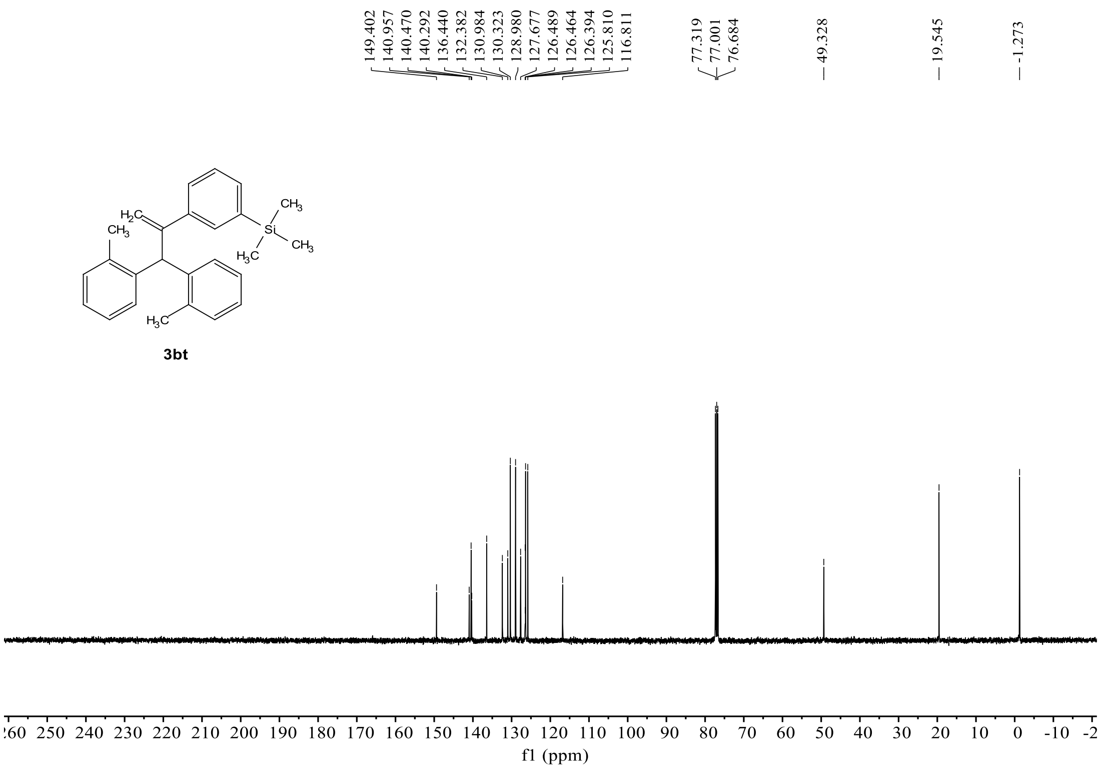

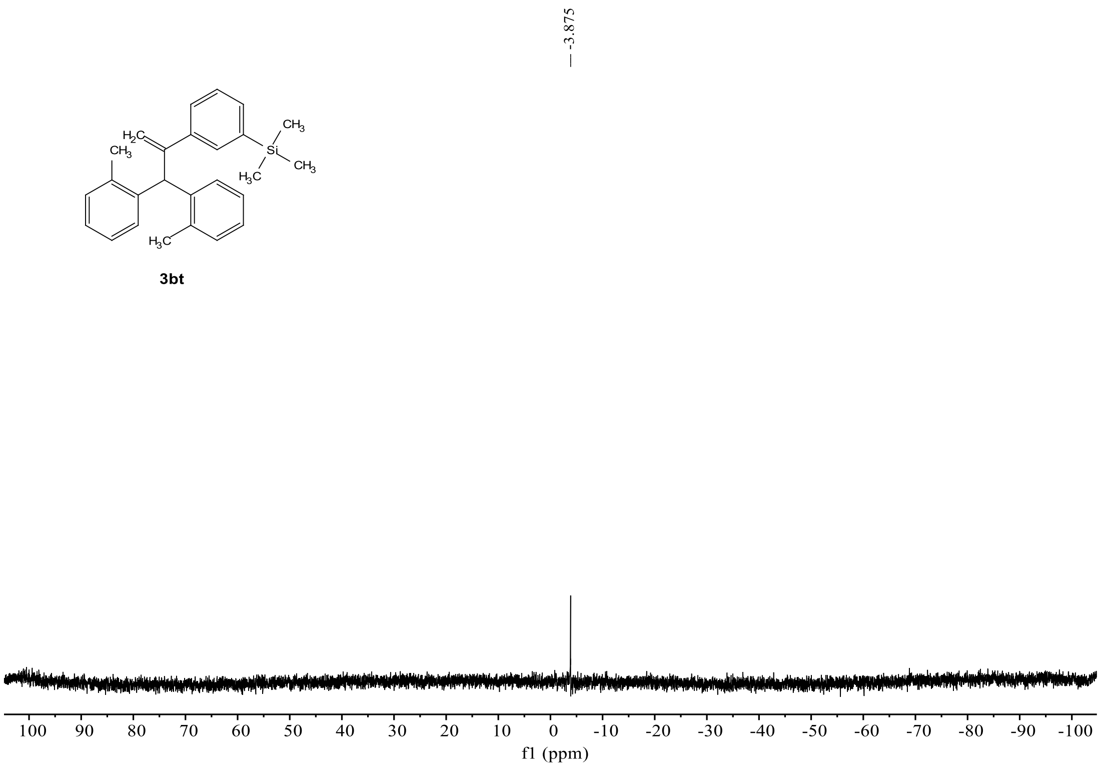

Figure S79. ^{1}H NMR ($CDCl_3$, 400 MHz), ^{13}C NMR ($CDCl_3$, 100 MHz), ^{29}Si NMR (80 MHz, $CDCl_3$) spectra of compound **3bt**

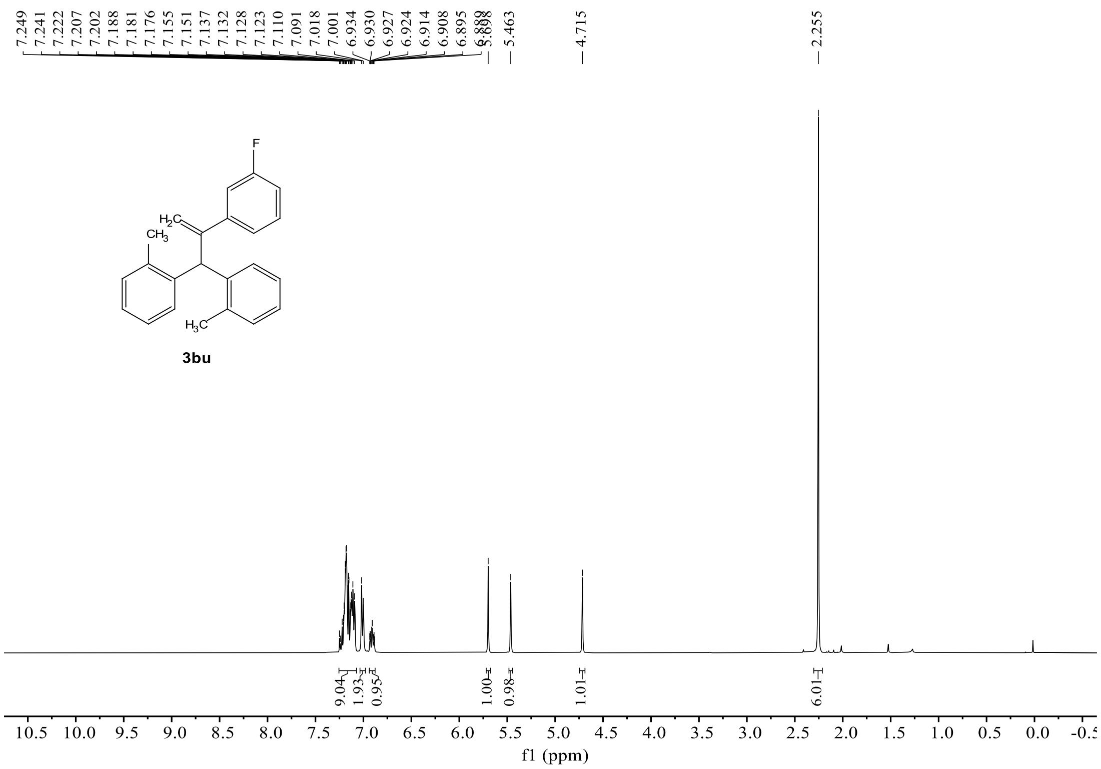

7.249
7.241
7.222
7.207
7.202
7.188
7.181
7.176
7.155
7.151
7.137
7.132
7.128
7.123
7.110
7.091
7.018
7.001
6.934
6.930
6.927
6.924
6.914
6.908
6.895
5.698
5.463
4.715
2.255
F
H_2C
CH_3
H_3C
3bu
9.04
1.93
0.95
1.00
0.98
1.01
6.01
10.5 10.0 9.5 9.0 8.5 8.0 7.5 7.0 6.5 6.0 5.5 5.0 4.5 4.0 3.5 3.0 2.5 2.0 1.5 1.0 0.5 0.0
f1 (ppm)

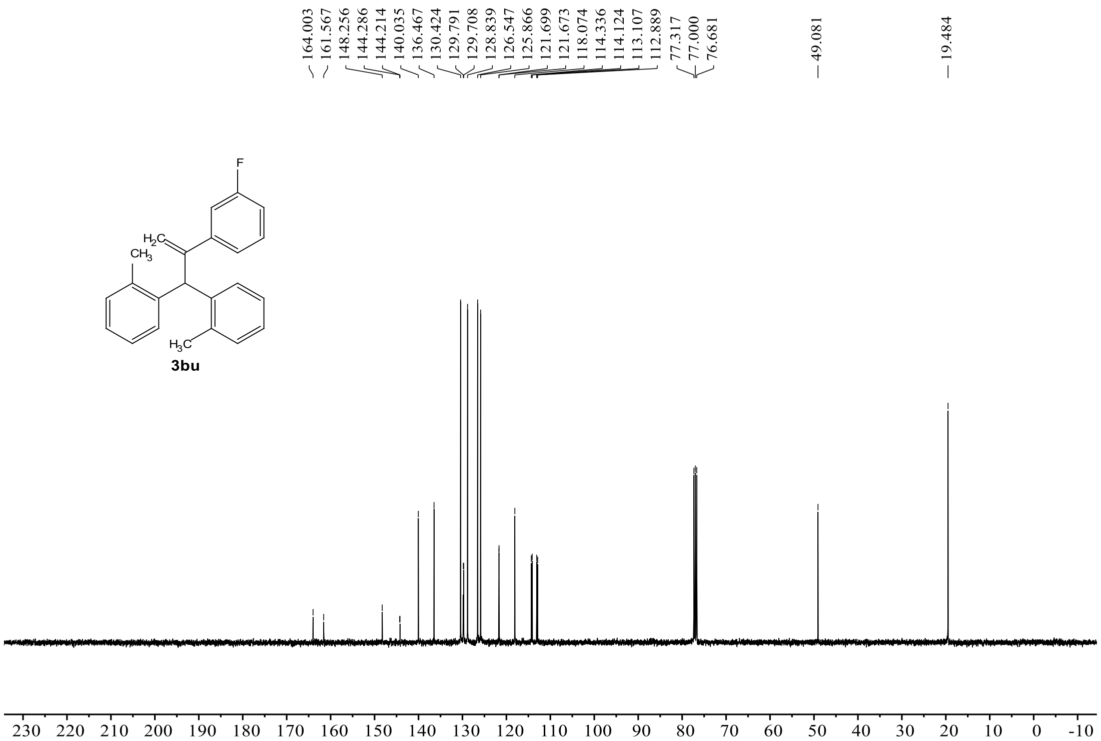

164.003
161.567
148.256
144.286
144.214
140.035
136.467
130.424
129.791
129.708
128.839
126.547
125.866
121.699
121.673
118.074
114.336
114.124
113.107
112.889
77.317
77.000
76.681
49.081
19.484
F
H_2C
CH_3
H_3C
3bu
230 220 210 200 190 180 170 160 150 140 130 120 110 100 90 80 70 60 50 40 30 20 10 0 -10
f1 (ppm)

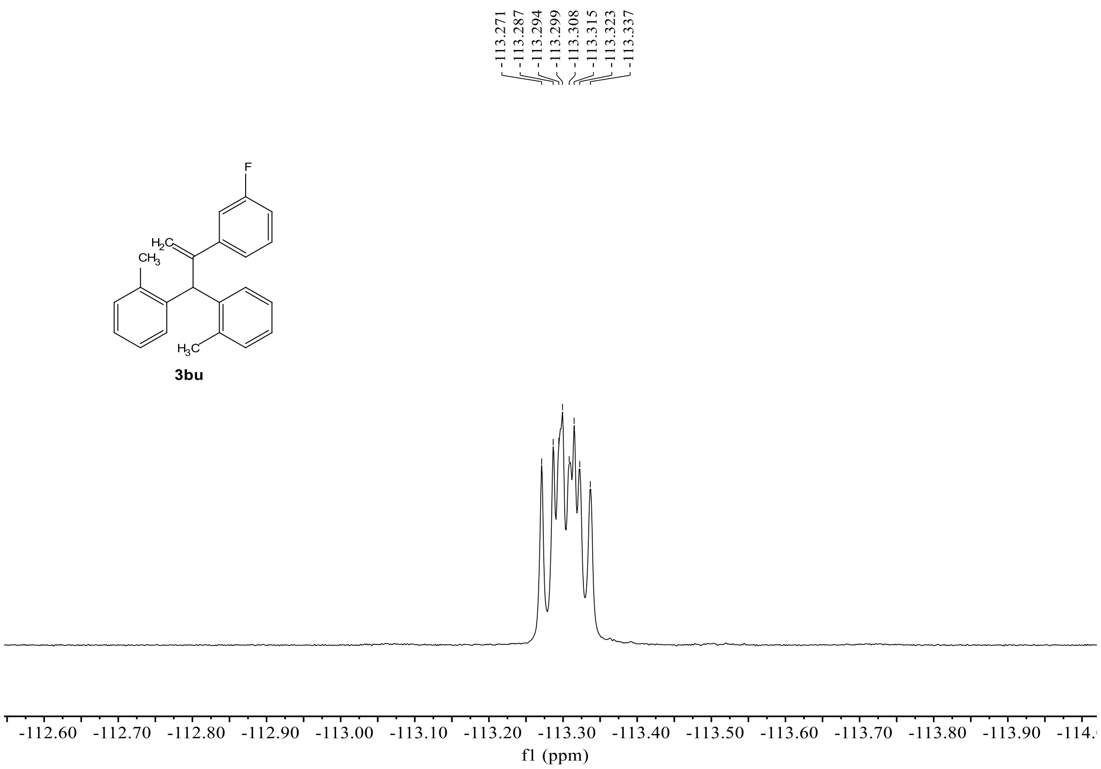

Figure S80. ^{1}H NMR ($CDCl_3$, 400 MHz), ^{13}C NMR ($CDCl_3$, 100 MHz), ^{19}F NMR ($CDCl_3$, 376 MHz) spectra of compound **3bu**

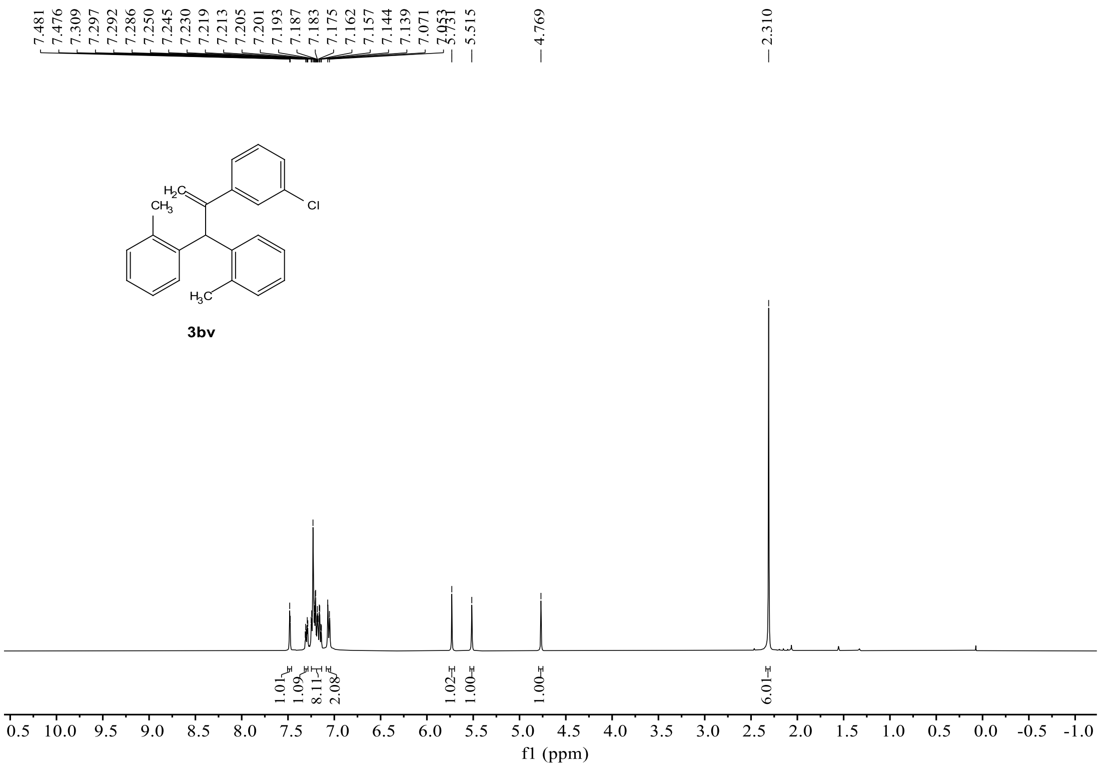

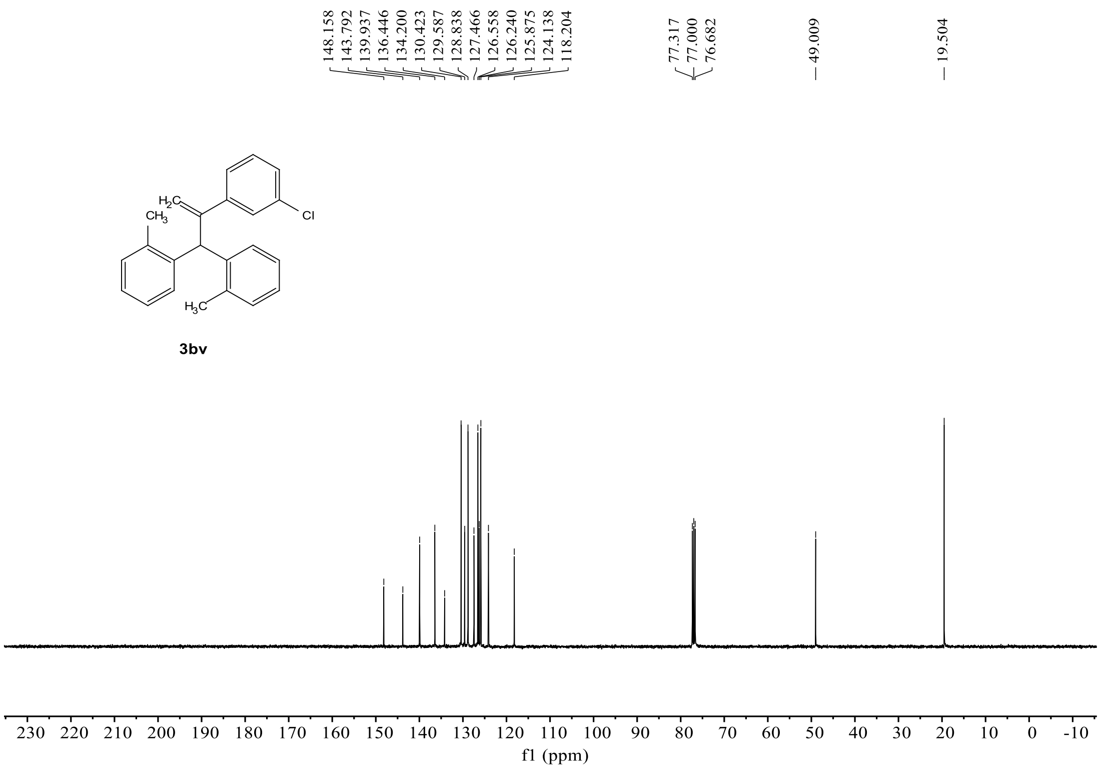

Figure S81. ^{1}H NMR ($CDCl_3$, 400 MHz) and ^{13}C NMR ($CDCl_3$, 100 MHz) spectra of compound **3bv**

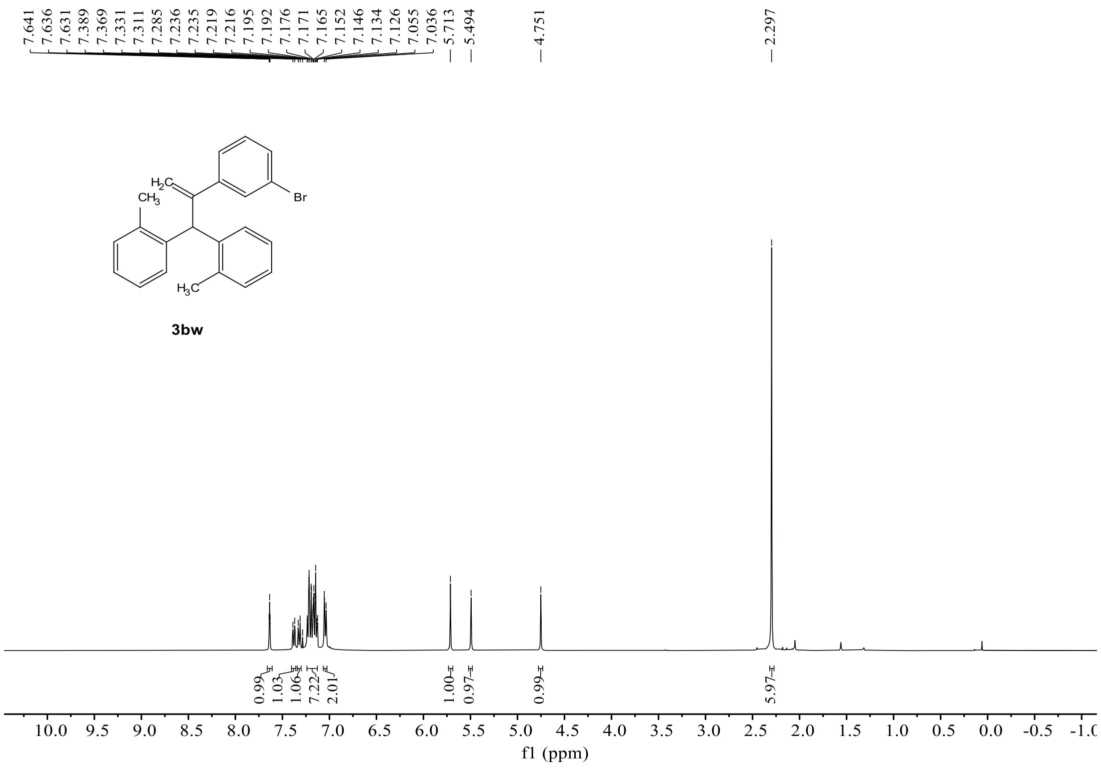

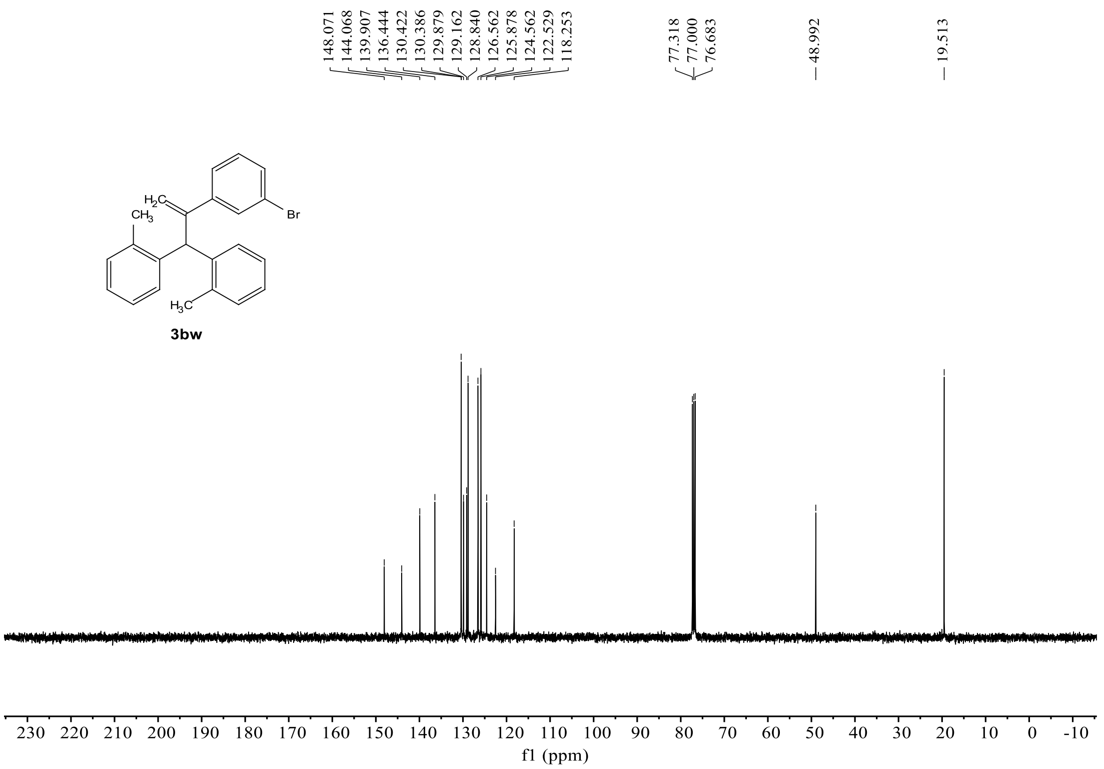

Figure S82. ^{1}H NMR ($CDCl_3$, 400 MHz) and ^{13}C NMR ($CDCl_3$, 100 MHz) spectra of compound **3bw**

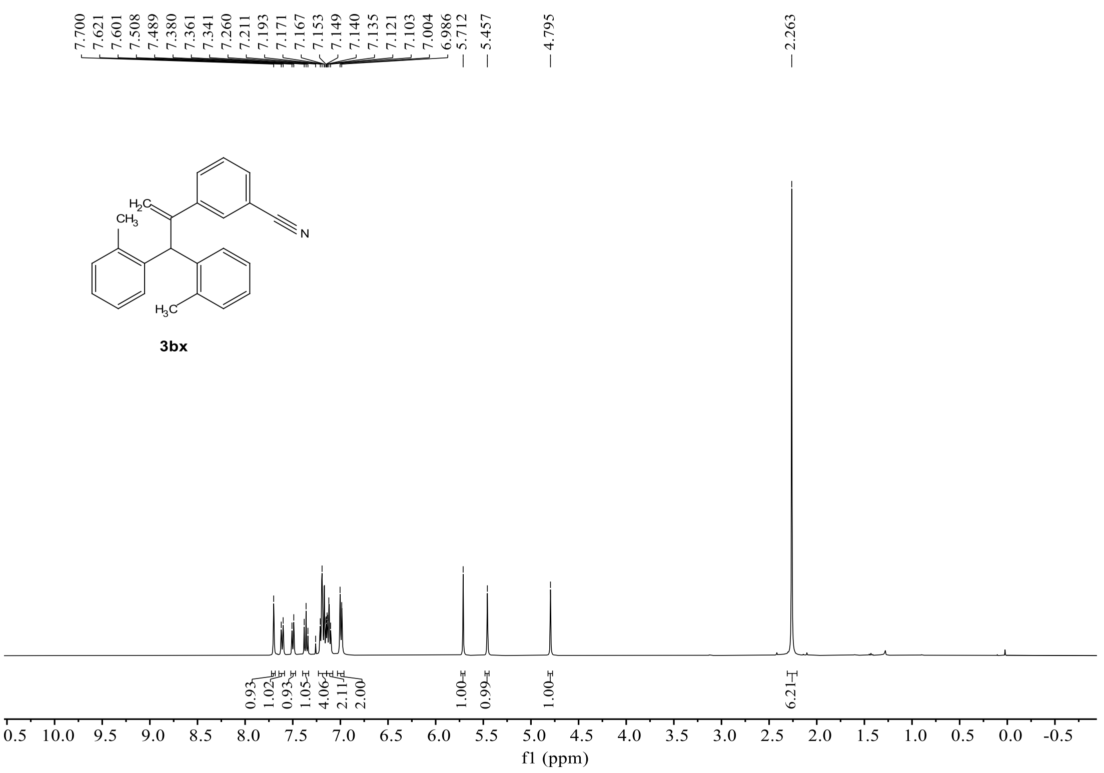

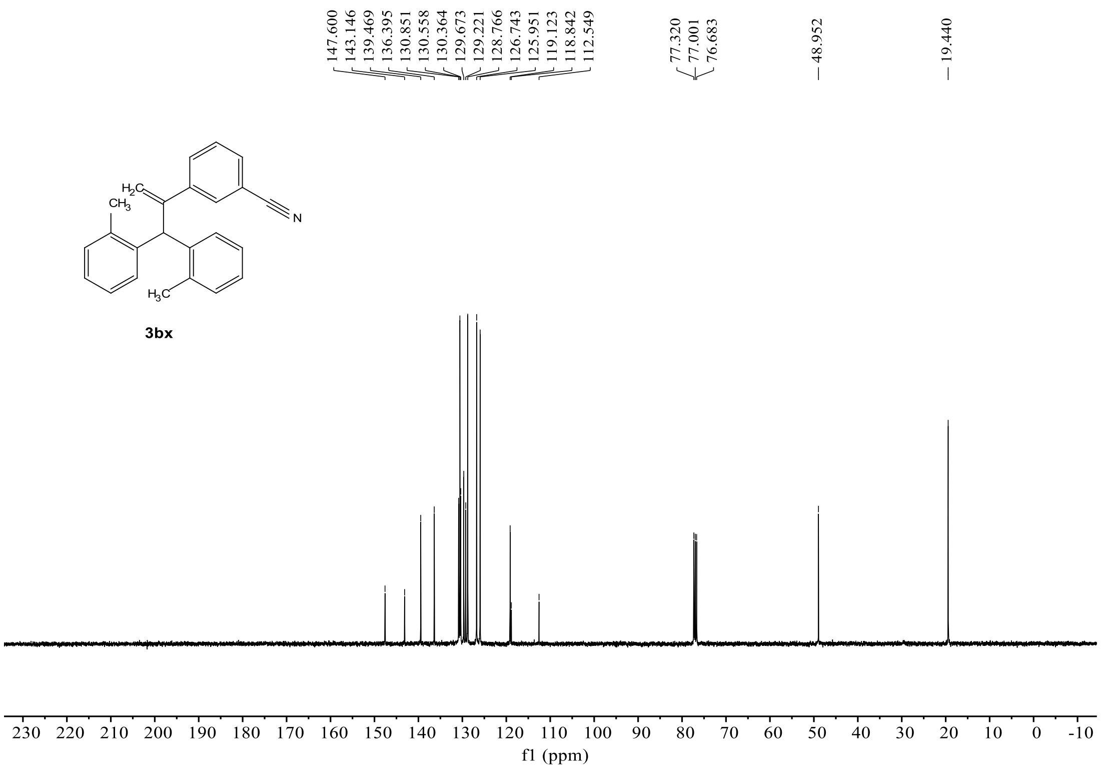

Figure S83. ^{1}H NMR ($CDCl_3$, 400 MHz) and ^{13}C NMR ($CDCl_3$, 100 MHz) spectra of compound **3bx**

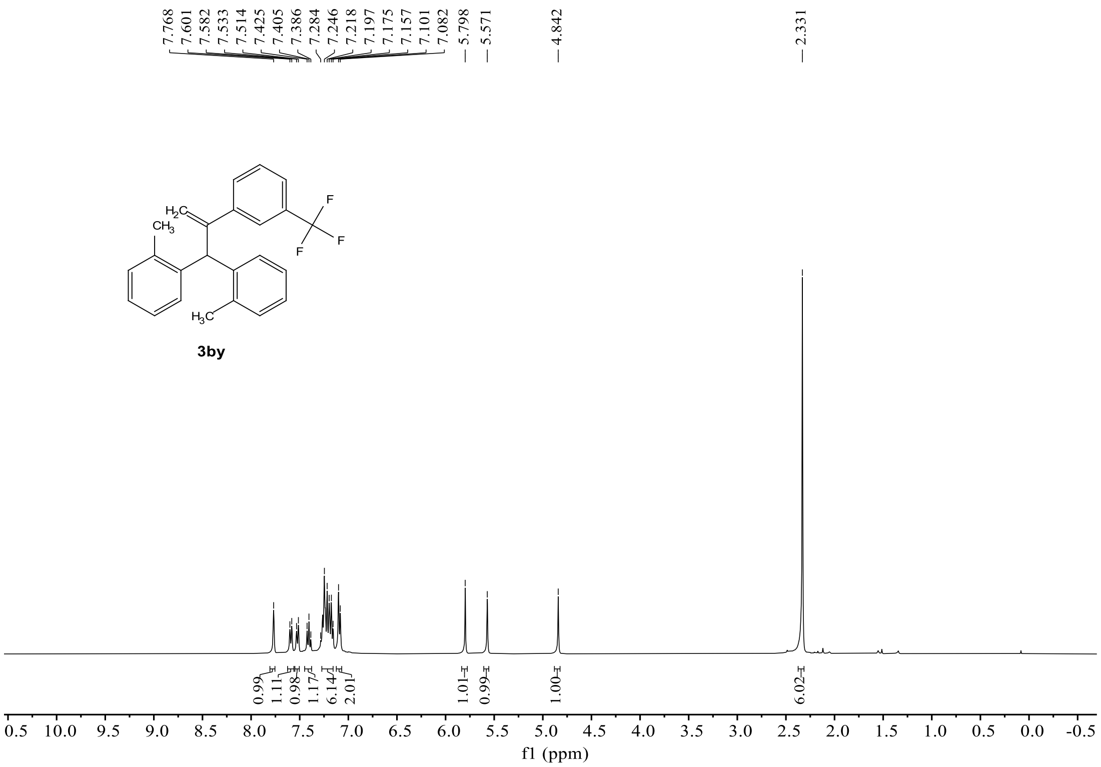

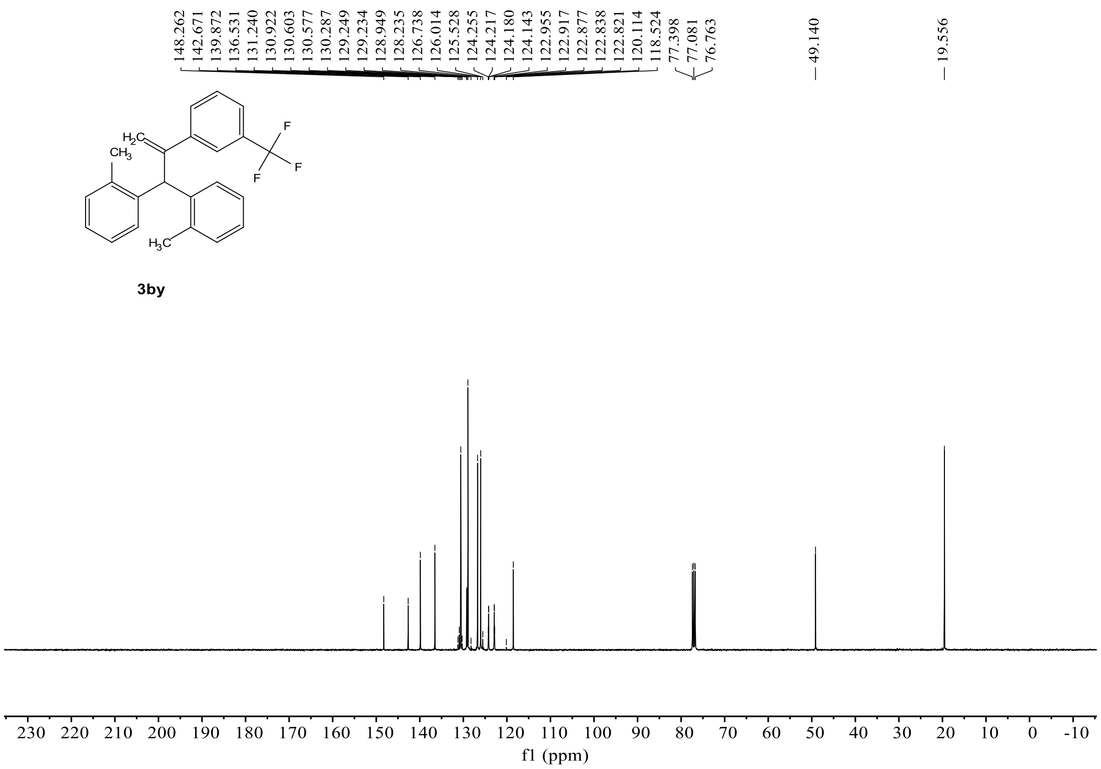

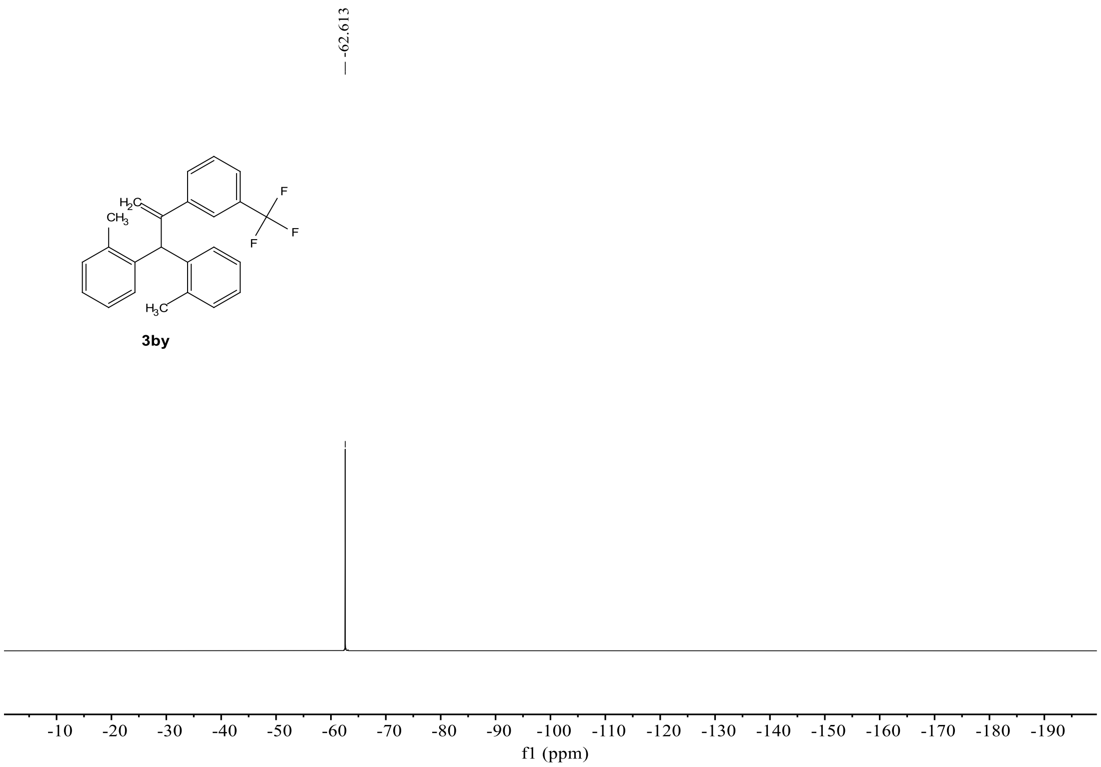

Figure S84. ^{1}H NMR ($CDCl_3$, 400 MHz), ^{13}C NMR ($CDCl_3$, 100 MHz), ^{19}F NMR ($CDCl_3$, 376 MHz) spectra of compound **3by**

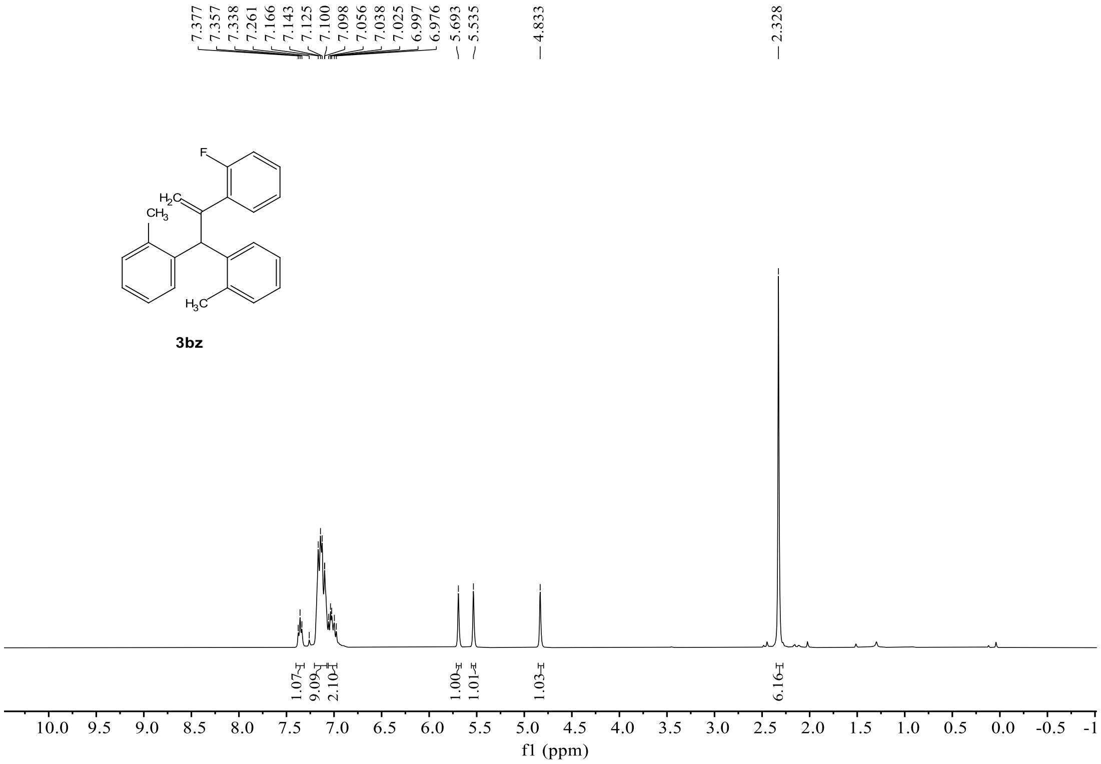
7.377
7.357
7.338
7.261
7.166
7.143
7.125
7.100
7.098
7.056
7.038
7.025
6.997
6.976
5.693
5.535
4.833
2.328
F
H_2C
CH_3
H_3C
3bz
1.07
9.09
2.10
1.00
1.01
1.03
6.16
10.0 9.5 9.0 8.5 8.0 7.5 7.0 6.5 6.0 5.5 5.0 4.5 4.0 3.5 3.0 2.5 2.0 1.5 1.0 0.5 0.0 -0.5 -1
f1 (ppm)

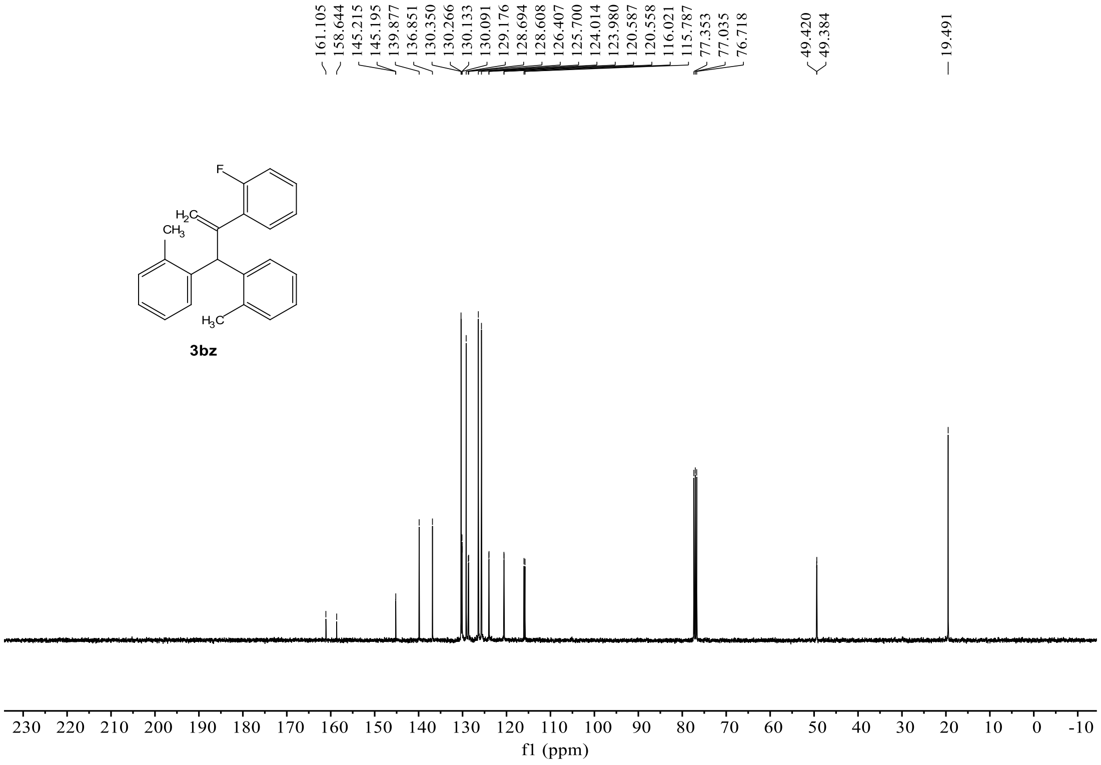
161.105
158.644
145.215
145.195
139.877
136.851
130.350
130.266
130.133
130.091
129.176
128.694
128.608
126.407
125.700
124.014
123.980
120.587
120.558
116.021
115.787
77.353
77.035
76.718
49.420
49.384
19.491
F
H_2C
CH_3
H_3C
3bz
230 220 210 200 190 180 170 160 150 140 130 120 110 100 90 80 70 60 50 40 30 20 10 0 -10
f1 (ppm)

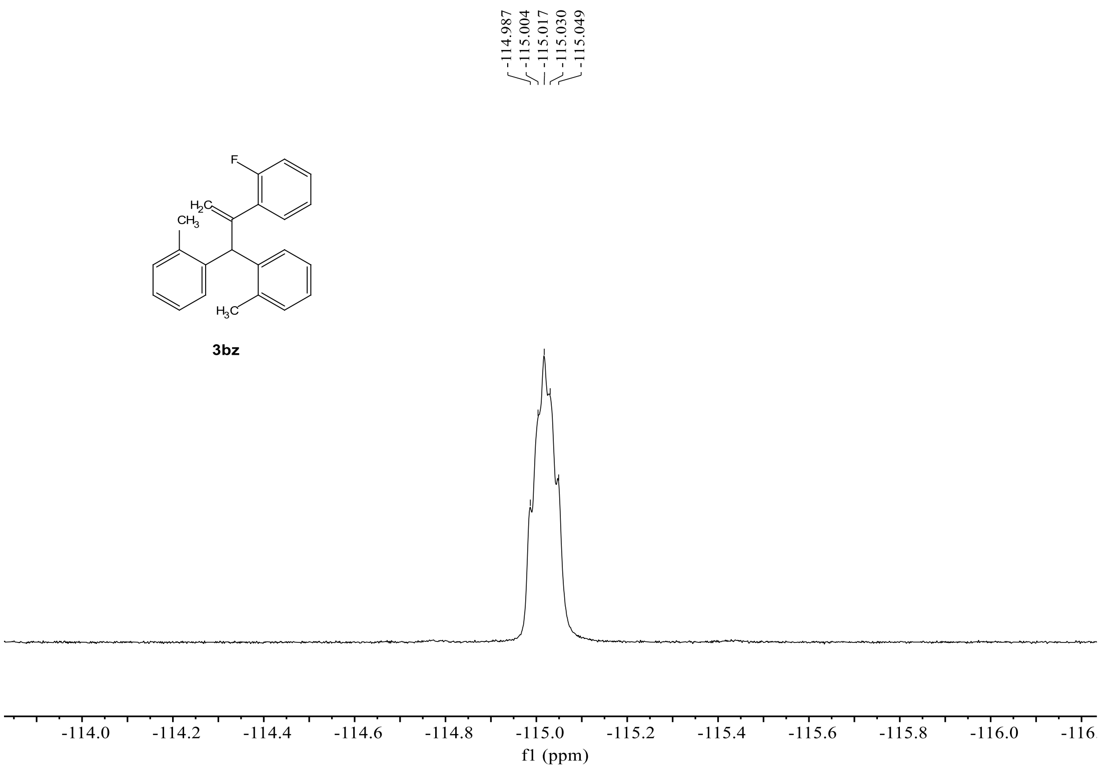

Figure S85. ^{1}H NMR ($CDCl_3$, 400 MHz), ^{13}C NMR ($CDCl_3$, 100 MHz), ^{19}F NMR ($CDCl_3$, 376 MHz) spectra of compound **3bz**

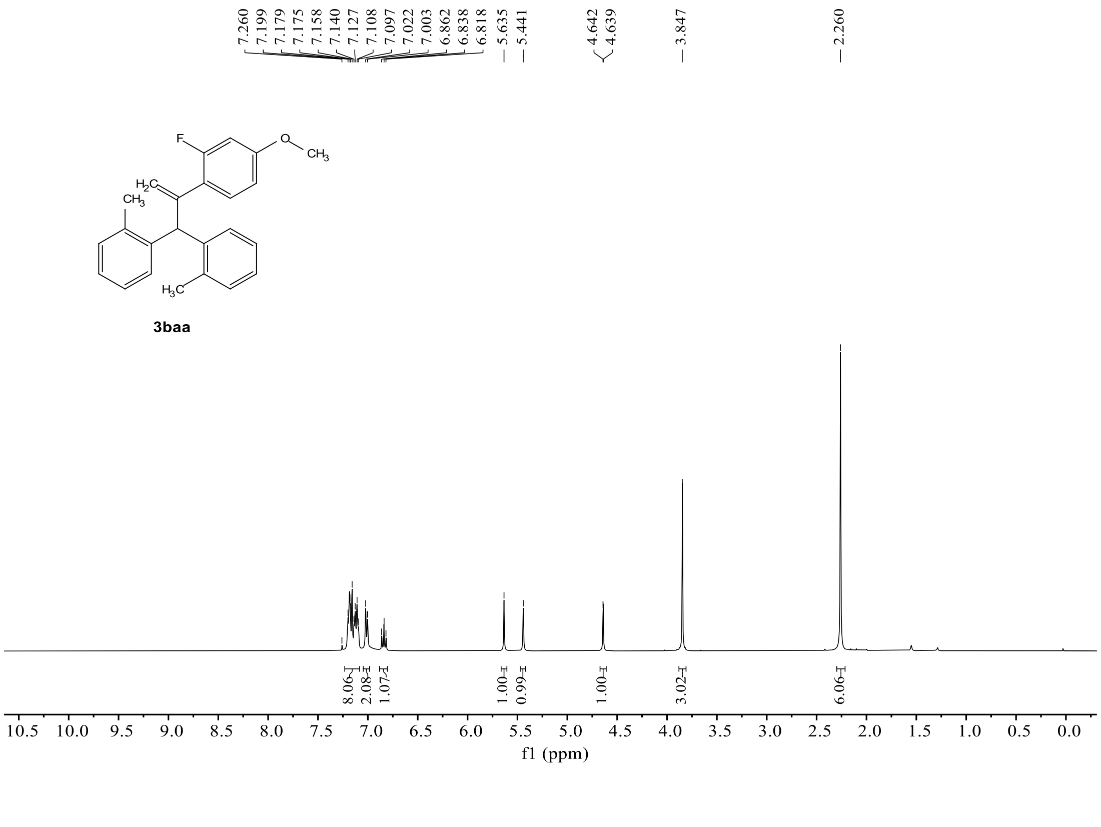

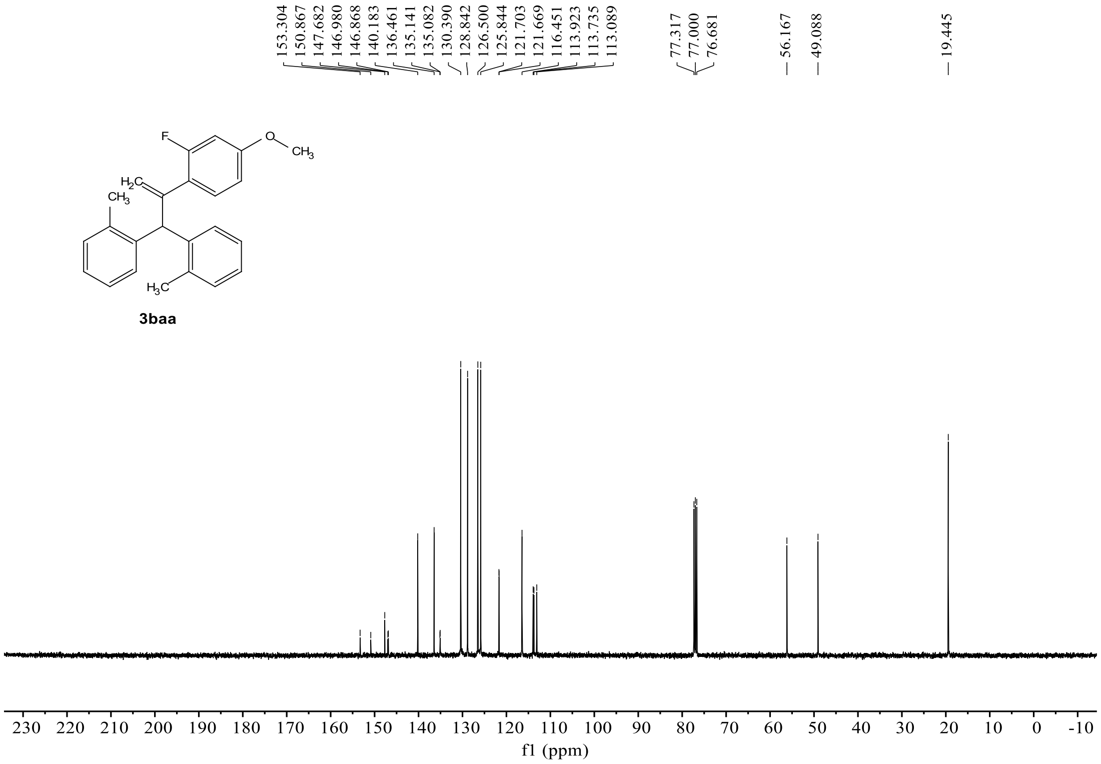

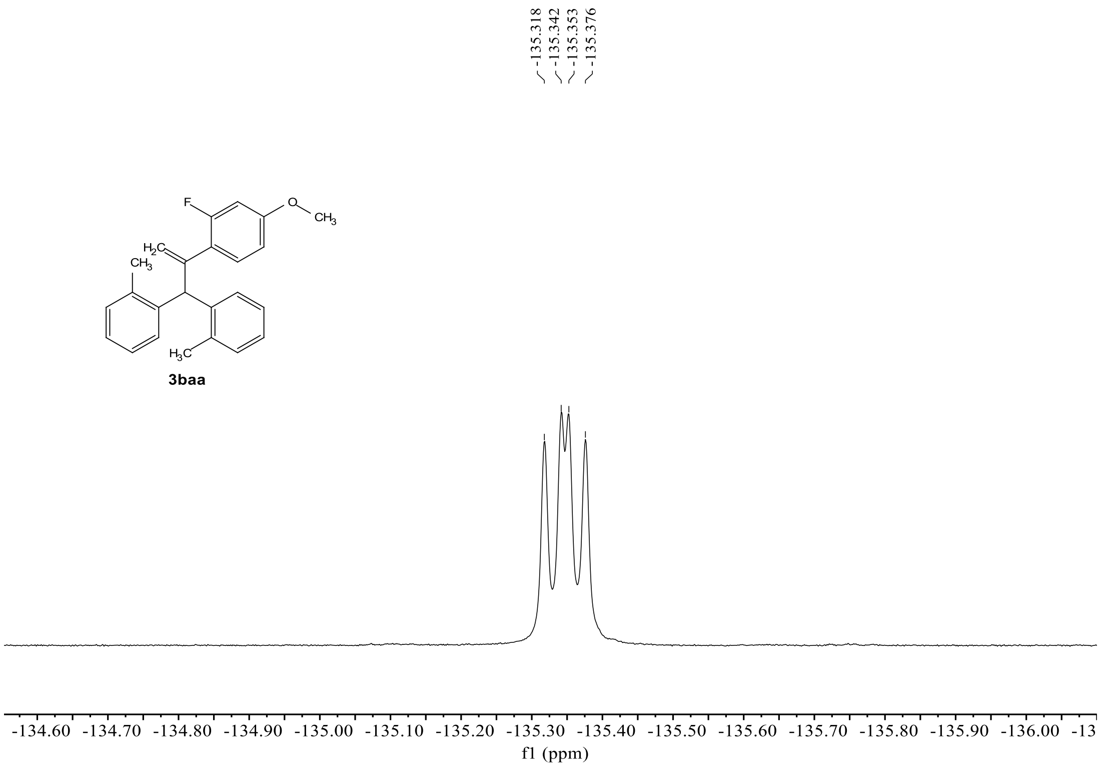

Figure S86. ^{1}H NMR ($CDCl_3$, 400 MHz), ^{13}C NMR ($CDCl_3$, 100 MHz), ^{19}F NMR ($CDCl_3$, 376 MHz) spectra of compound **3baa**

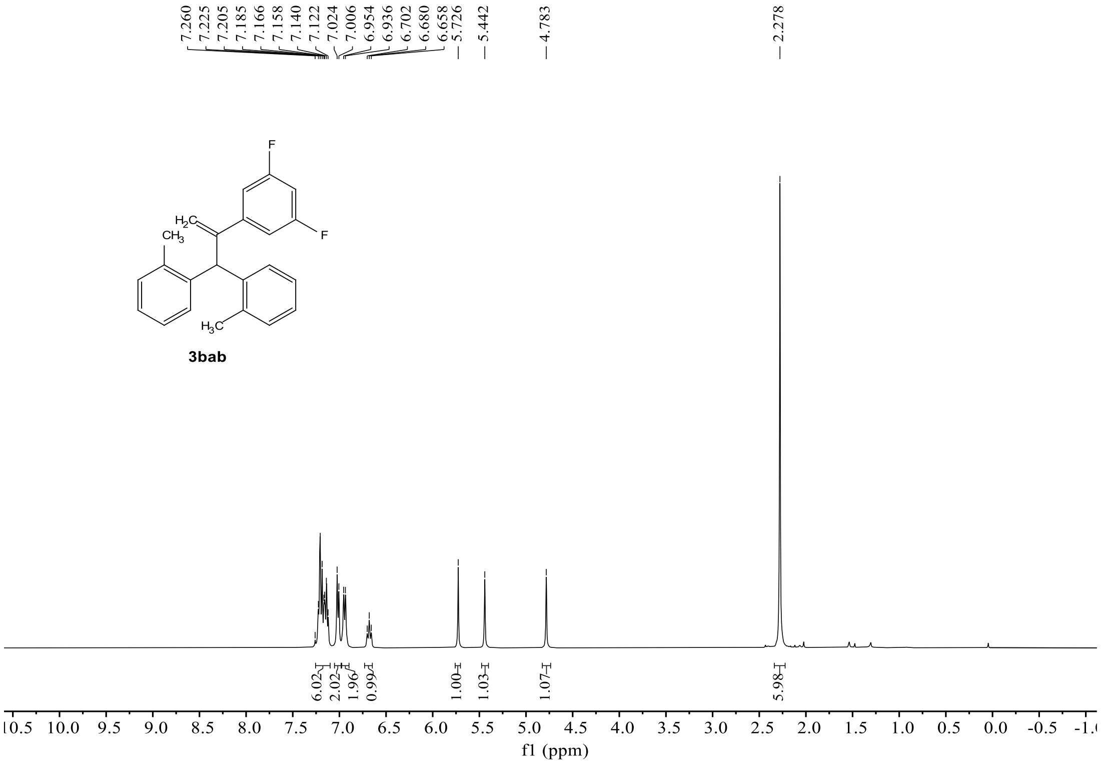
7.260
7.225
7.205
7.185
7.166
7.158
7.140
7.122
7.024
7.006
6.954
6.936
6.702
6.680
6.658
5.726
5.442
4.783
2.278
F
F
H2C
CH3
H3C
3bab
6.02
2.02
1.96
0.99
1.00
1.03
1.07
5.98
f1 (ppm)

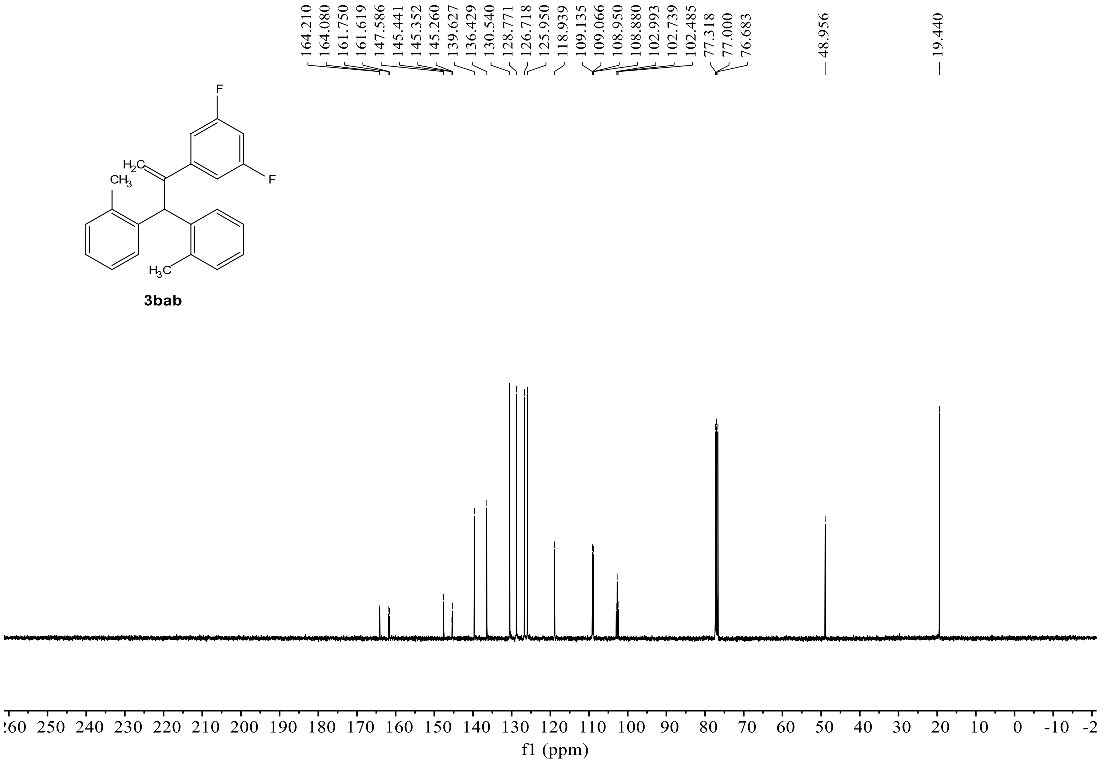
164.210
164.080
161.750
161.619
147.586
145.441
145.352
145.260
139.627
136.429
130.540
128.771
126.718
125.950
118.939
109.135
109.066
108.950
108.880
102.993
102.739
102.485
77.318
77.000
76.683
48.956
19.440
F
F
H2C
CH3
H3C
3bab
f1 (ppm)

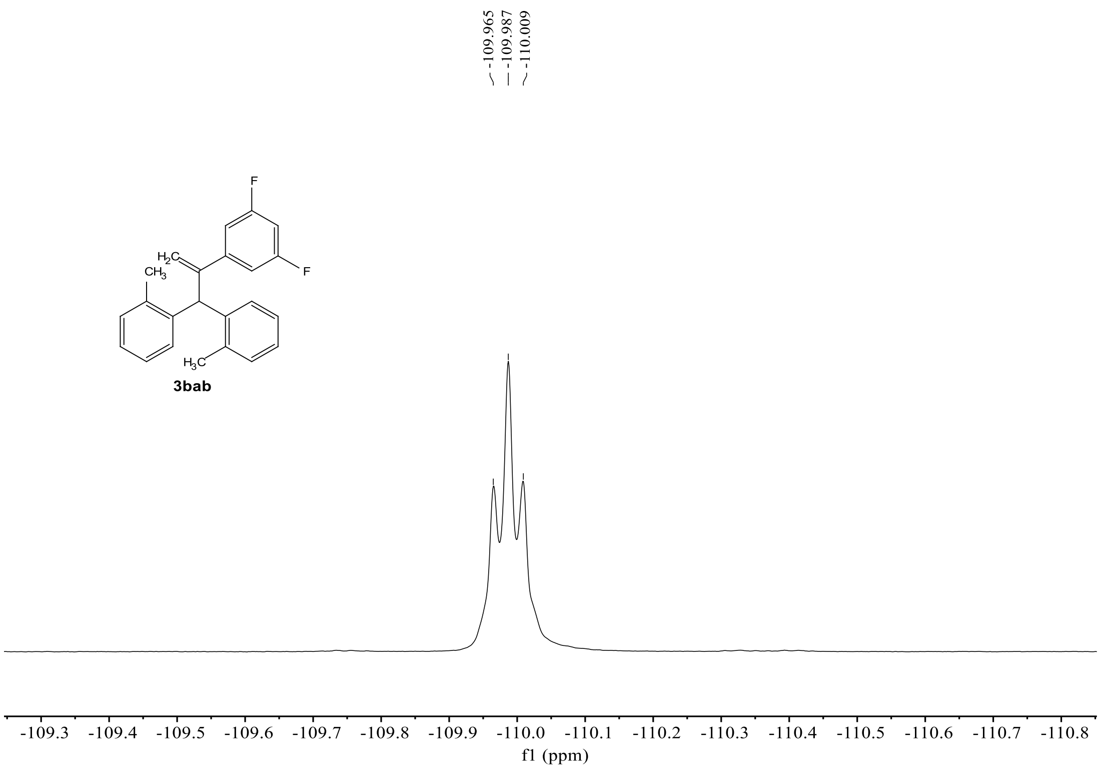

Figure S87. ^{1}H NMR ($CDCl_3$, 400 MHz), ^{13}C NMR ($CDCl_3$, 100 MHz), ^{19}F NMR ($CDCl_3$, 376 MHz) spectra of compound **3bab**

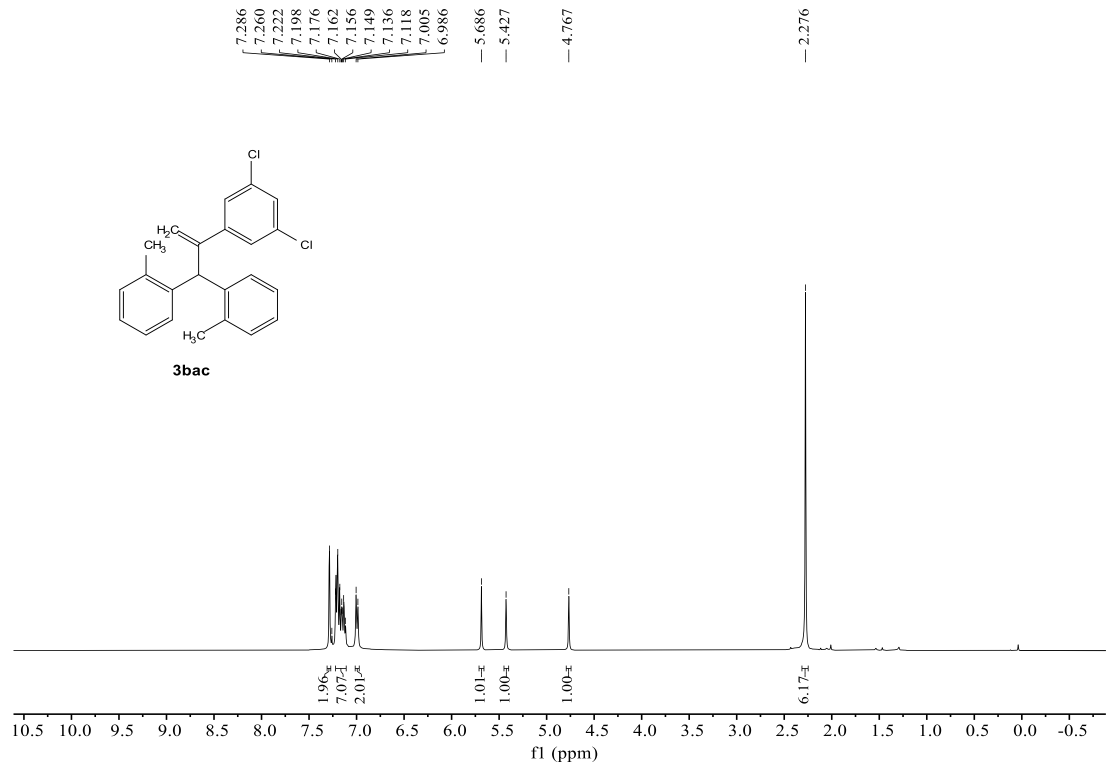

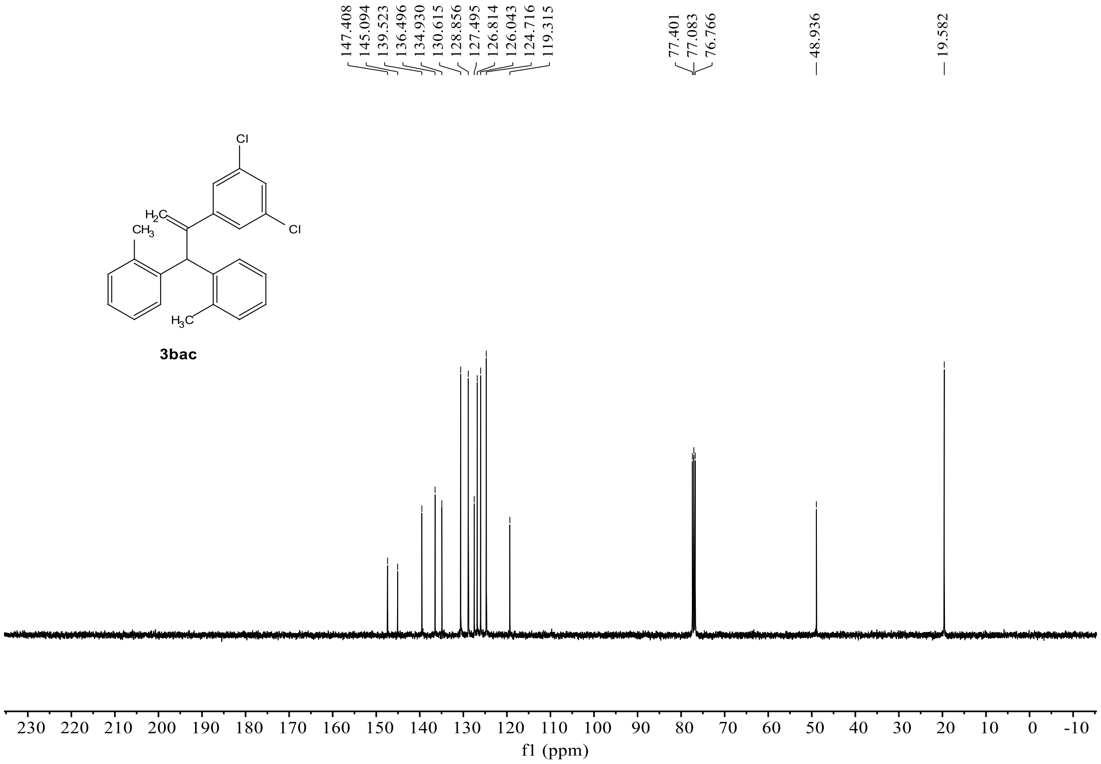

Figure S88. ^{1}H NMR ($CDCl_3$, 400 MHz) and ^{13}C NMR ($CDCl_3$, 100 MHz) spectra of compound **3bac**

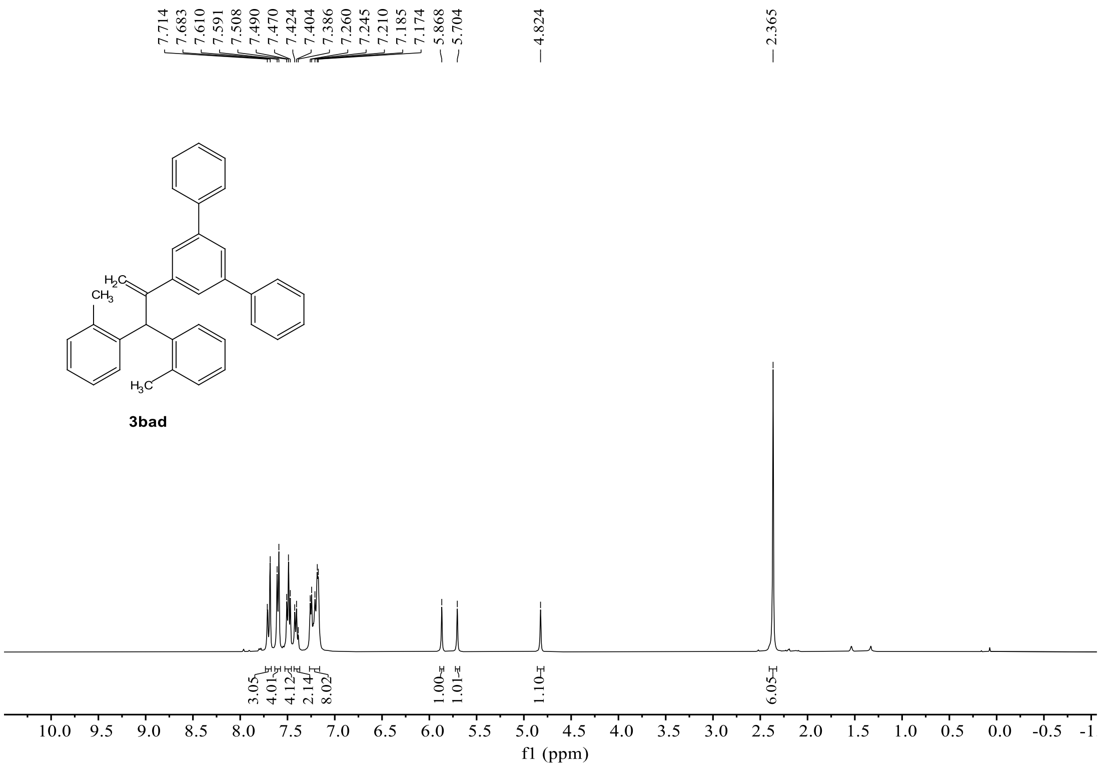

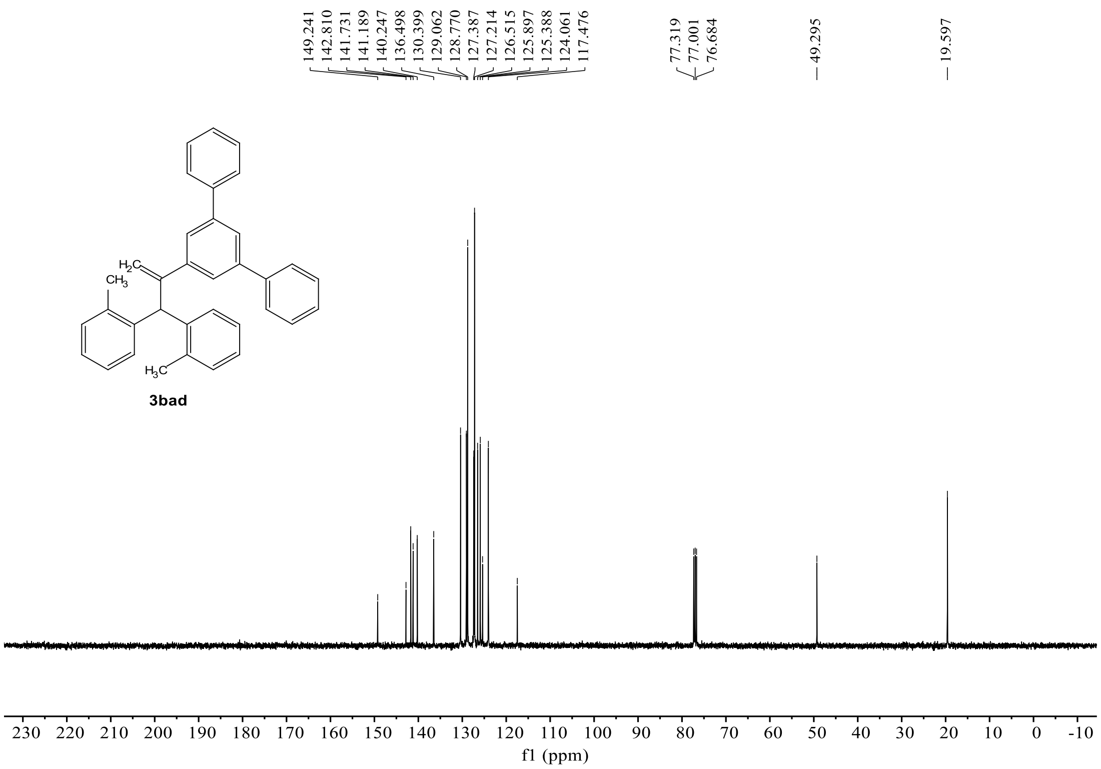

Figure S89. ¹H NMR ($CDCl_3$, 400 MHz) and ¹³C NMR ($CDCl_3$, 100 MHz) spectra of compound **3bad**

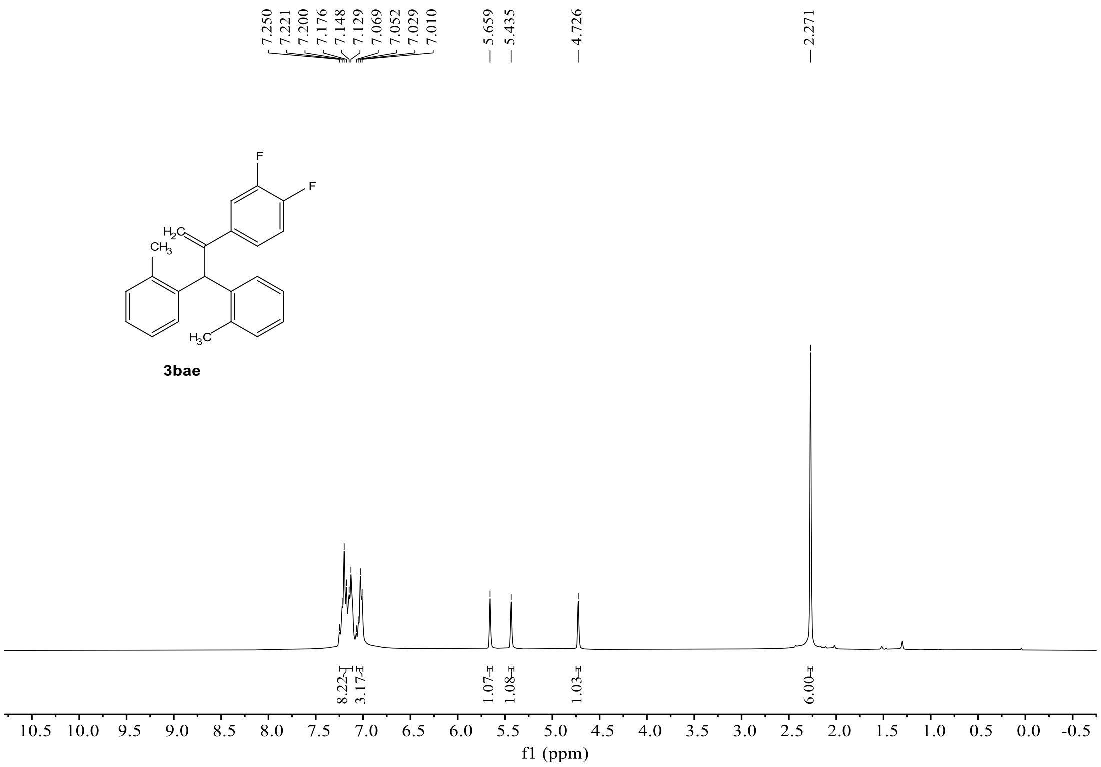

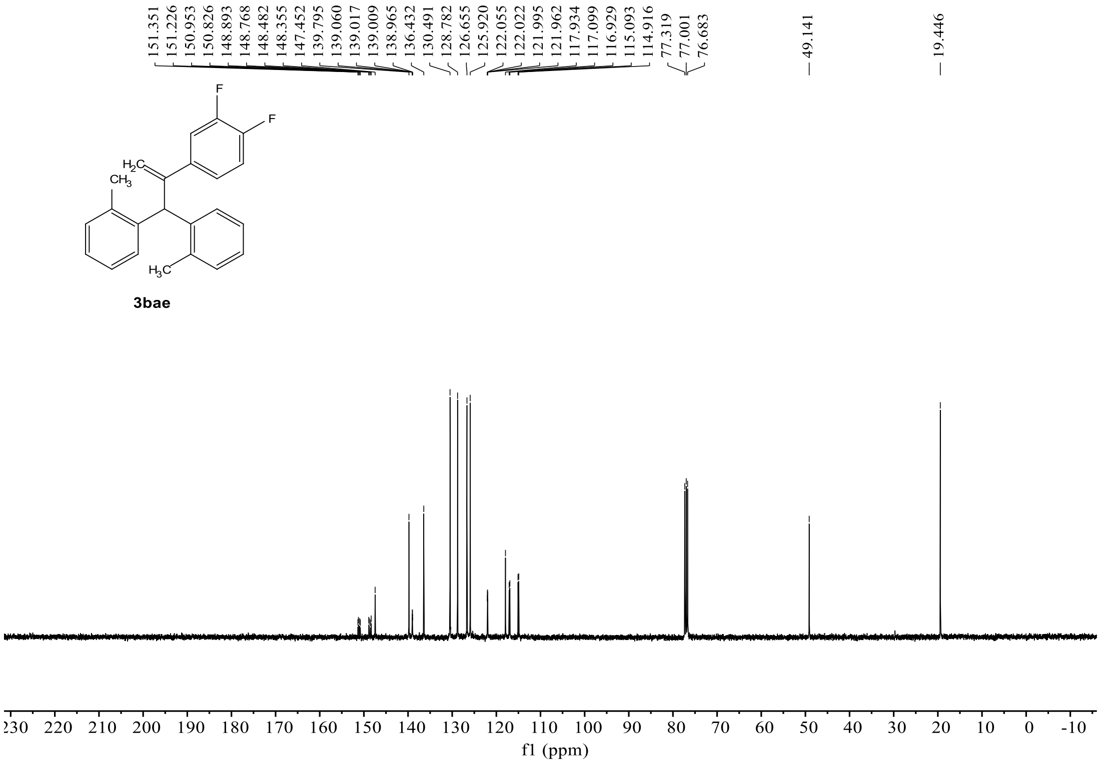

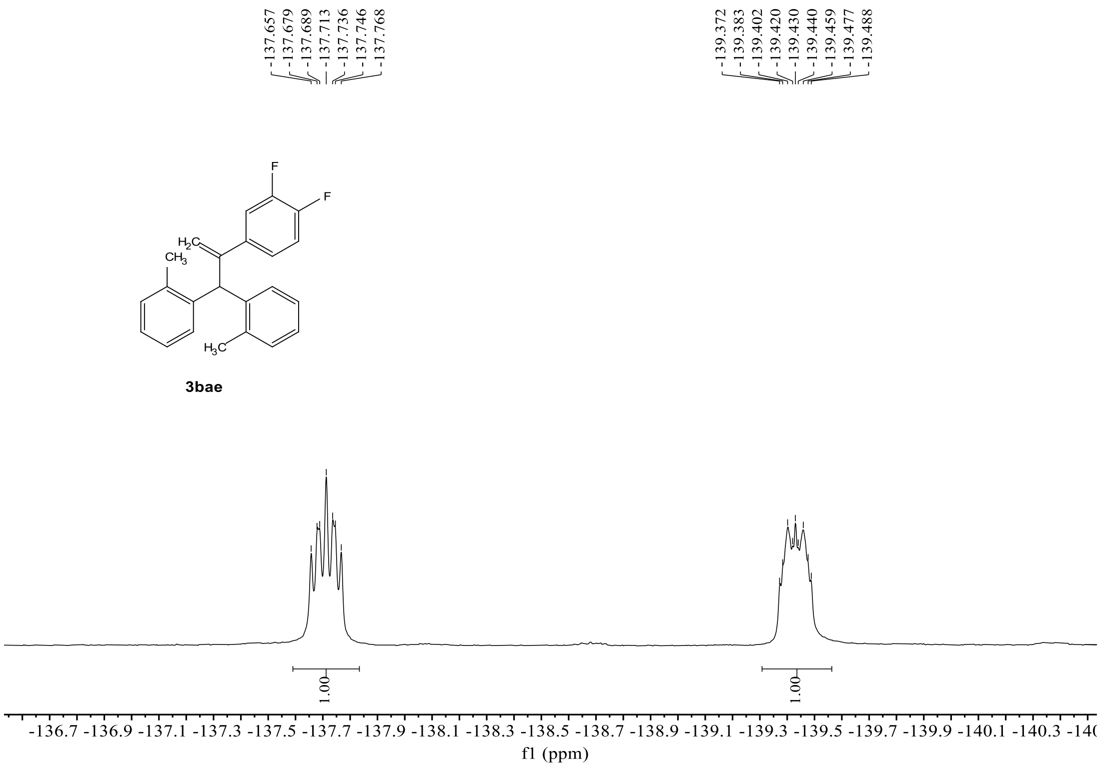

Figure S90. ^{1}H NMR ($CDCl_3$, 400 MHz), ^{13}C NMR ($CDCl_3$, 100 MHz), ^{19}F NMR ($CDCl_3$, 376 MHz) spectra of compound **3bae**

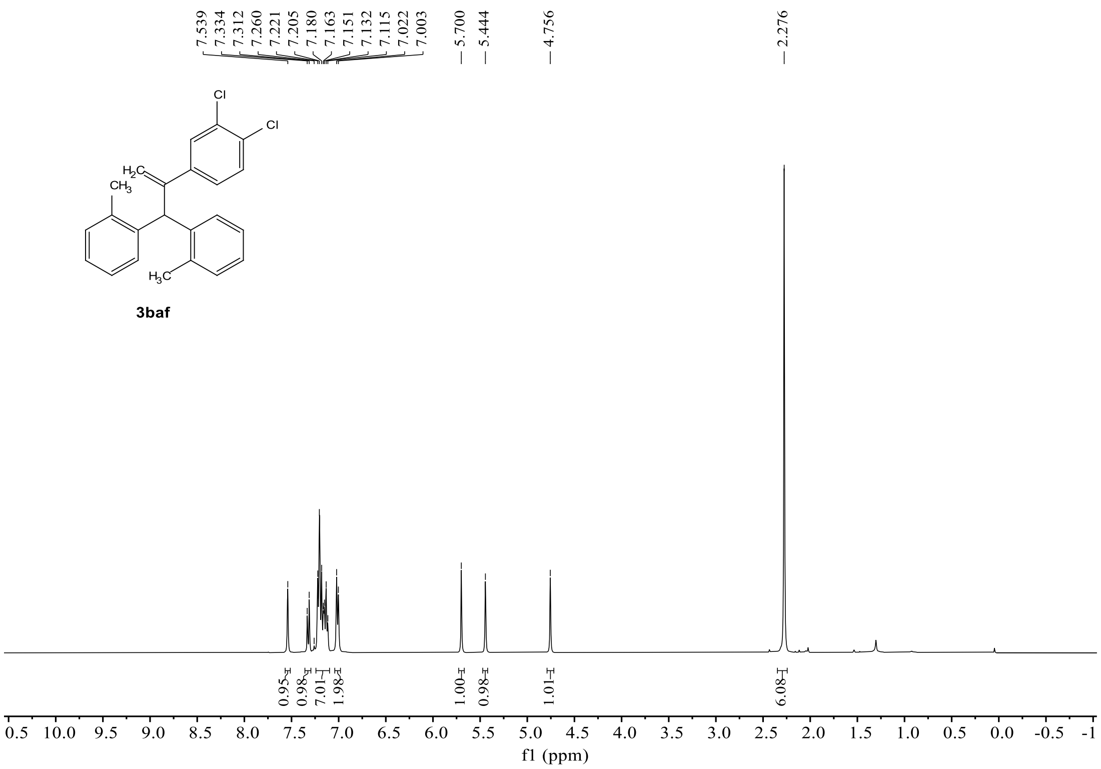

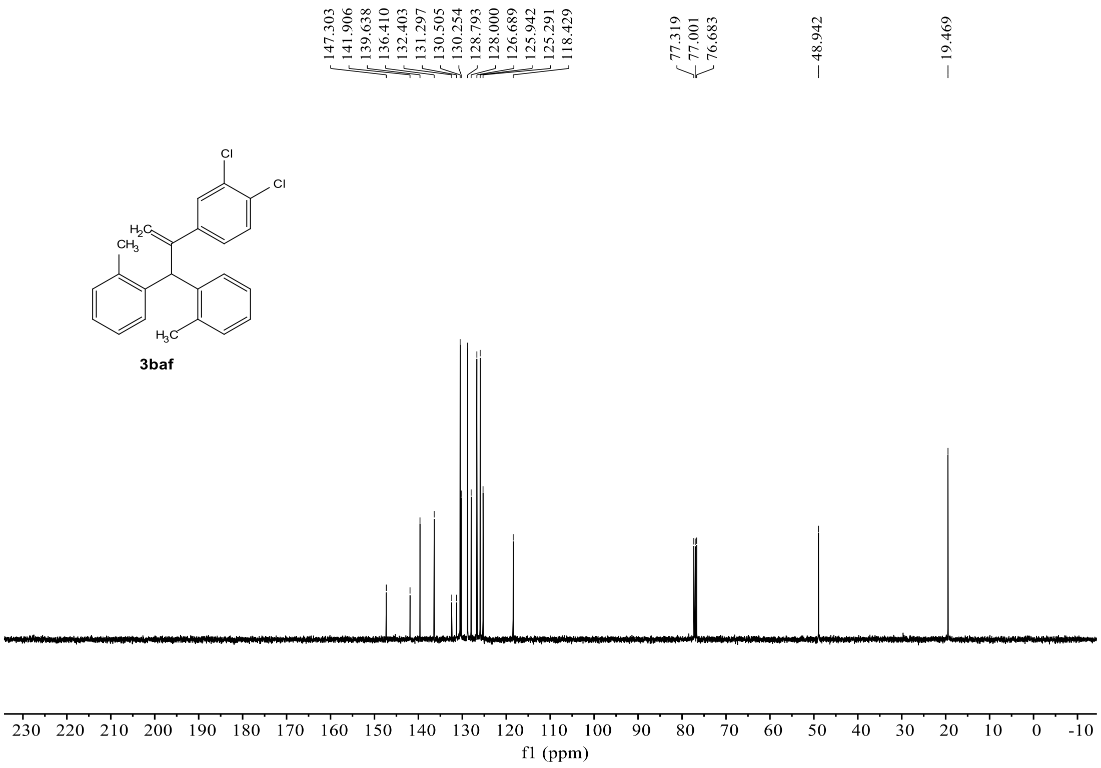

Figure S91. ^{1}H NMR ($CDCl_3$, 400 MHz) and ^{13}C NMR ($CDCl_3$, 100 MHz) spectra of compound **3baf**

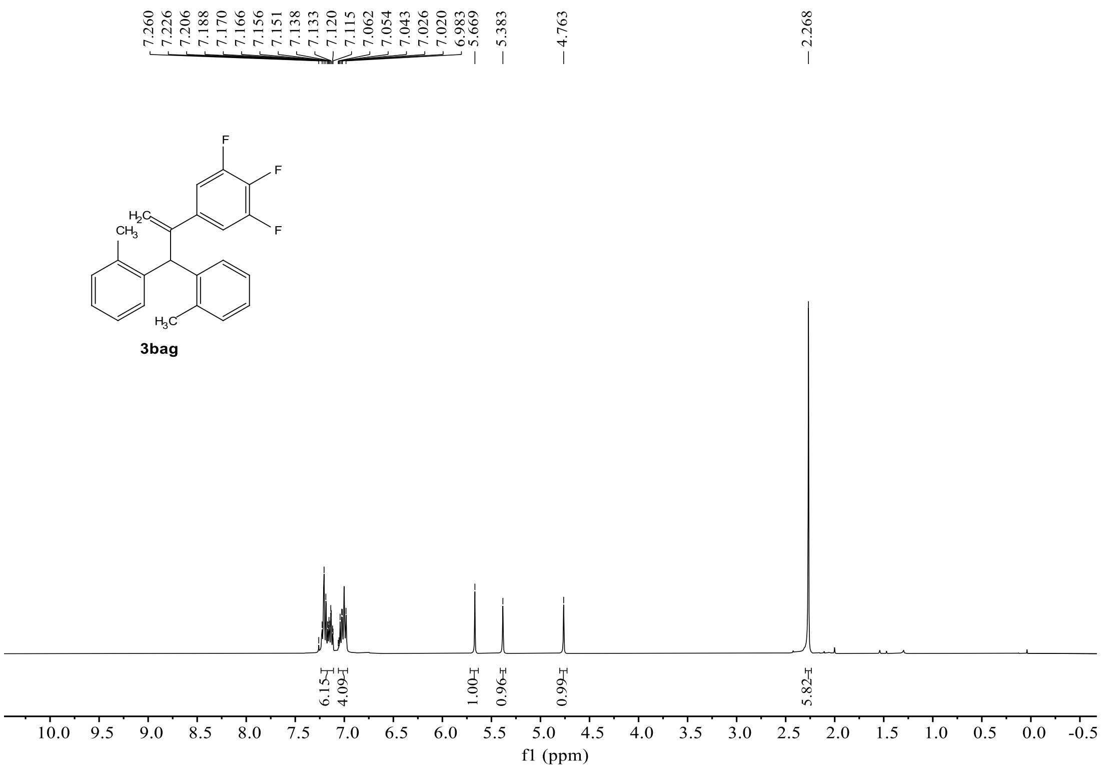
7.260
7.226
7.206
7.188
7.170
7.166
7.156
7.151
7.138
7.133
7.120
7.115
7.062
7.054
7.043
7.026
7.020
6.983
5.669
5.383
4.763
2.268
F
F
F
H_2C
CH_3
H_3C
3bag
6.15
4.09
1.00
0.96
0.99
5.82
10.0 9.5 9.0 8.5 8.0 7.5 7.0 6.5 6.0 5.5 5.0 4.5 4.0 3.5 3.0 2.5 2.0 1.5 1.0 0.5 0.0 -0.5
f1 (ppm)

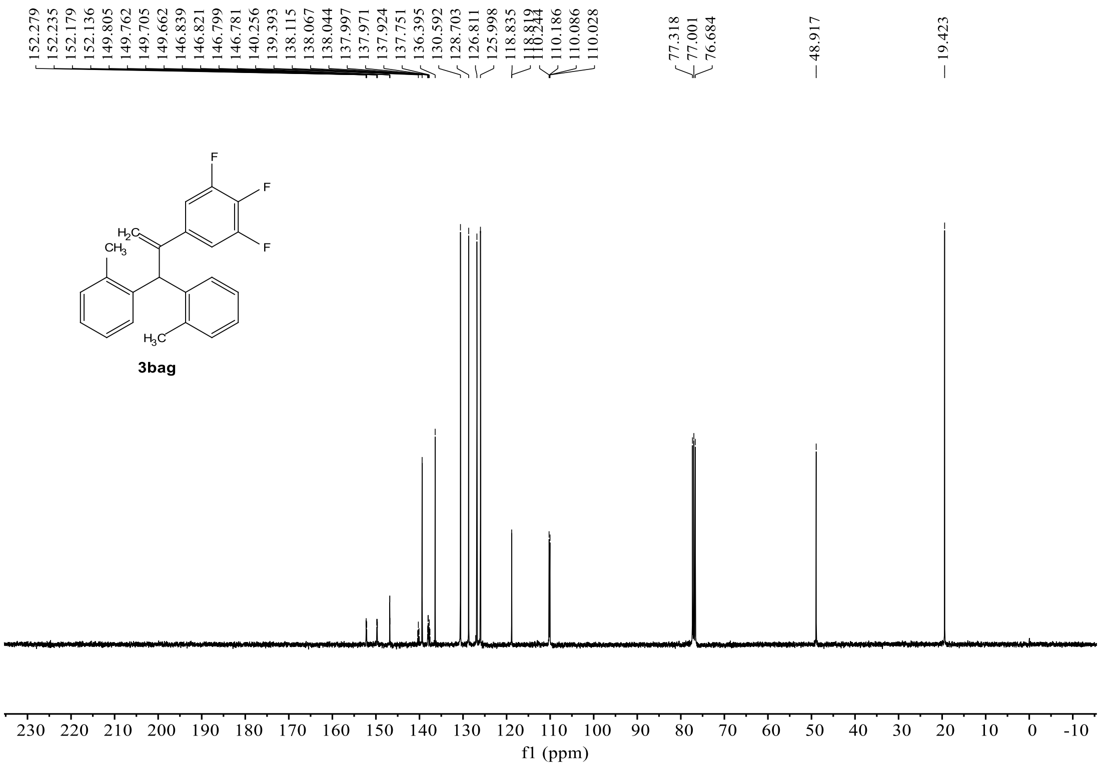
152.279
152.235
152.179
152.136
149.805
149.762
149.705
149.662
146.839
146.821
146.799
146.781
140.256
139.393
138.115
138.067
138.044
137.997
137.971
137.924
137.751
136.395
130.592
128.703
126.811
125.998
118.835
118.819
110.244
110.186
110.086
110.028
77.318
77.001
76.684
48.917
19.423
F
F
F
H_2C
CH_3
H_3C
3bag
230 220 210 200 190 180 170 160 150 140 130 120 110 100 90 80 70 60 50 40 30 20 10 0 -10
f1 (ppm)

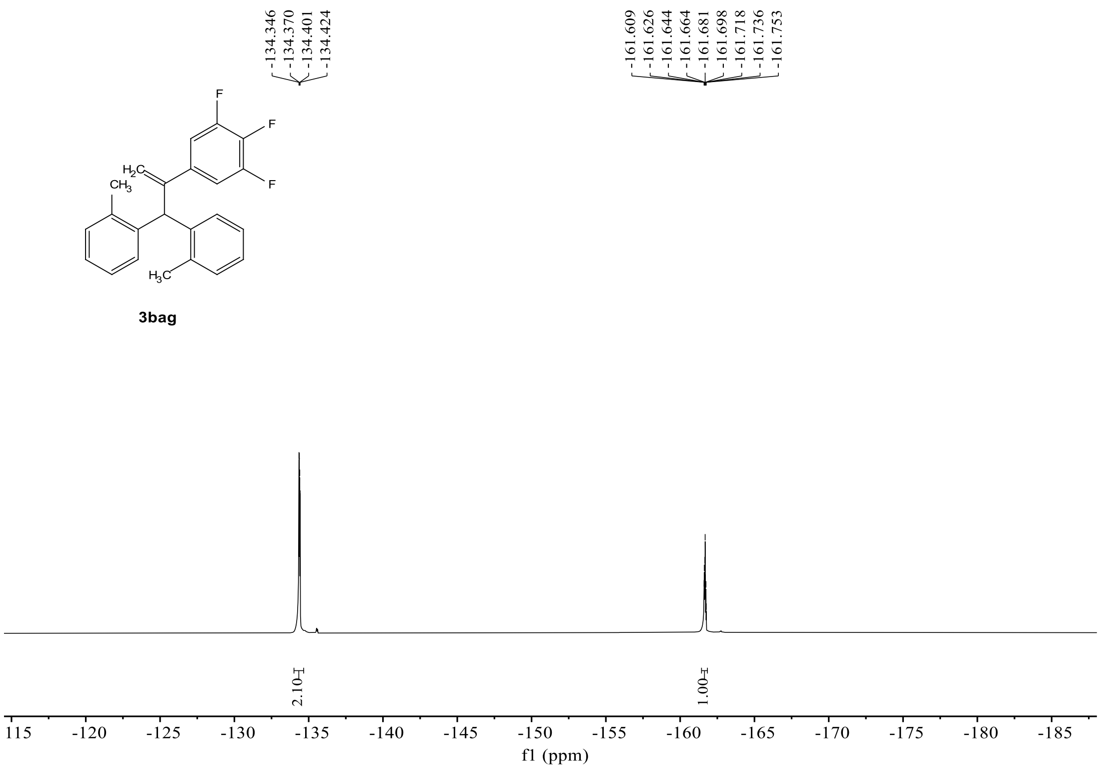

Figure S92. ^{1}H NMR ($CDCl_3$, 400 MHz), ^{13}C NMR ($CDCl_3$, 100 MHz), ^{19}F NMR ($CDCl_3$, 376 MHz) spectra of compound **3bag**

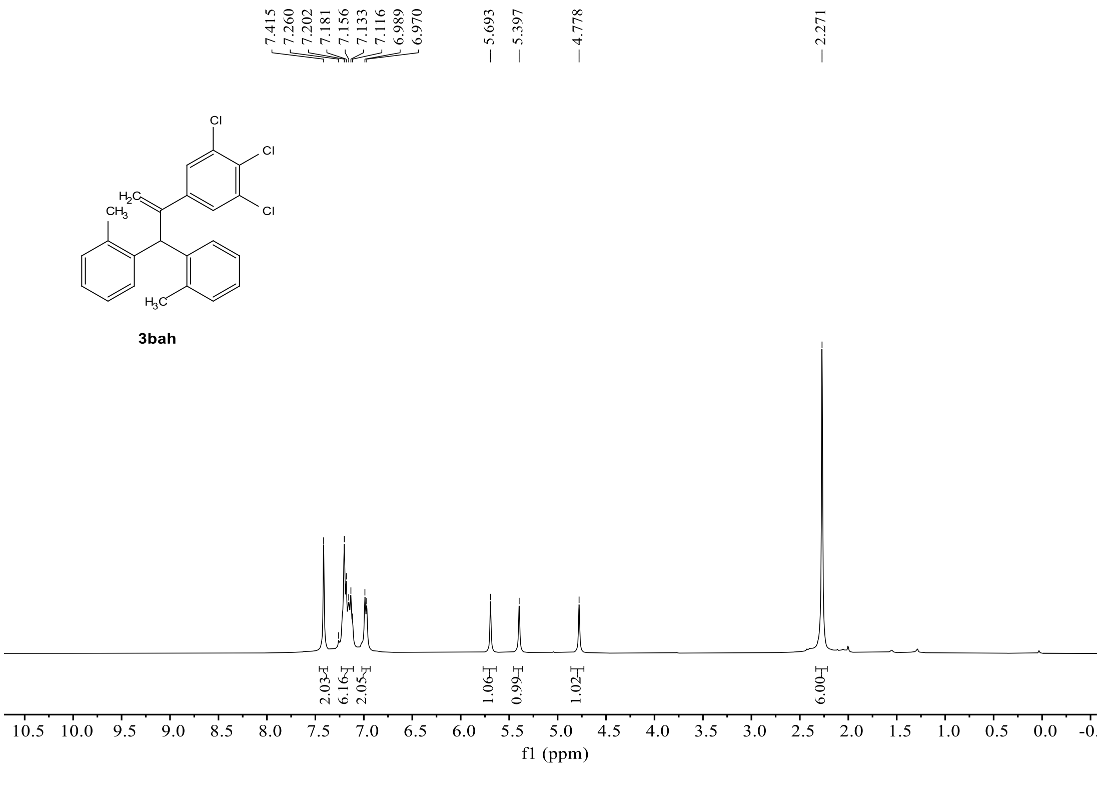

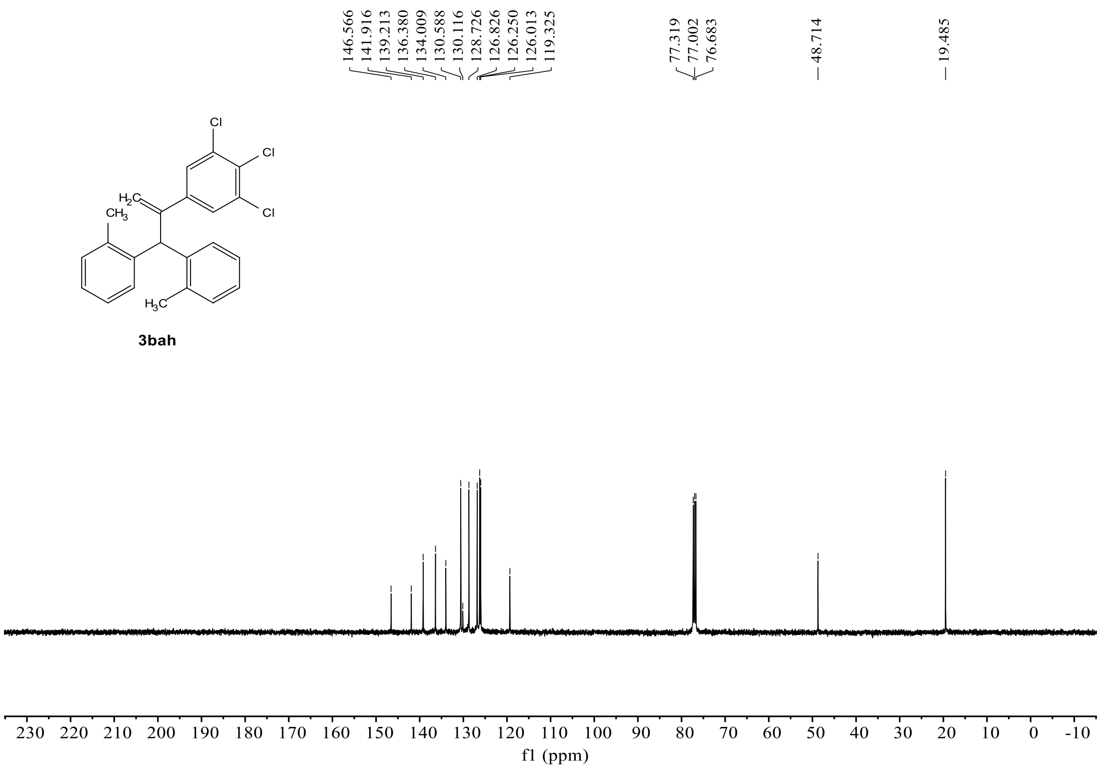

Figure S93. ^{1}H NMR ($CDCl_3$, 400 MHz) and ^{13}C NMR ($CDCl_3$, 100 MHz) spectra of compound **3bah**

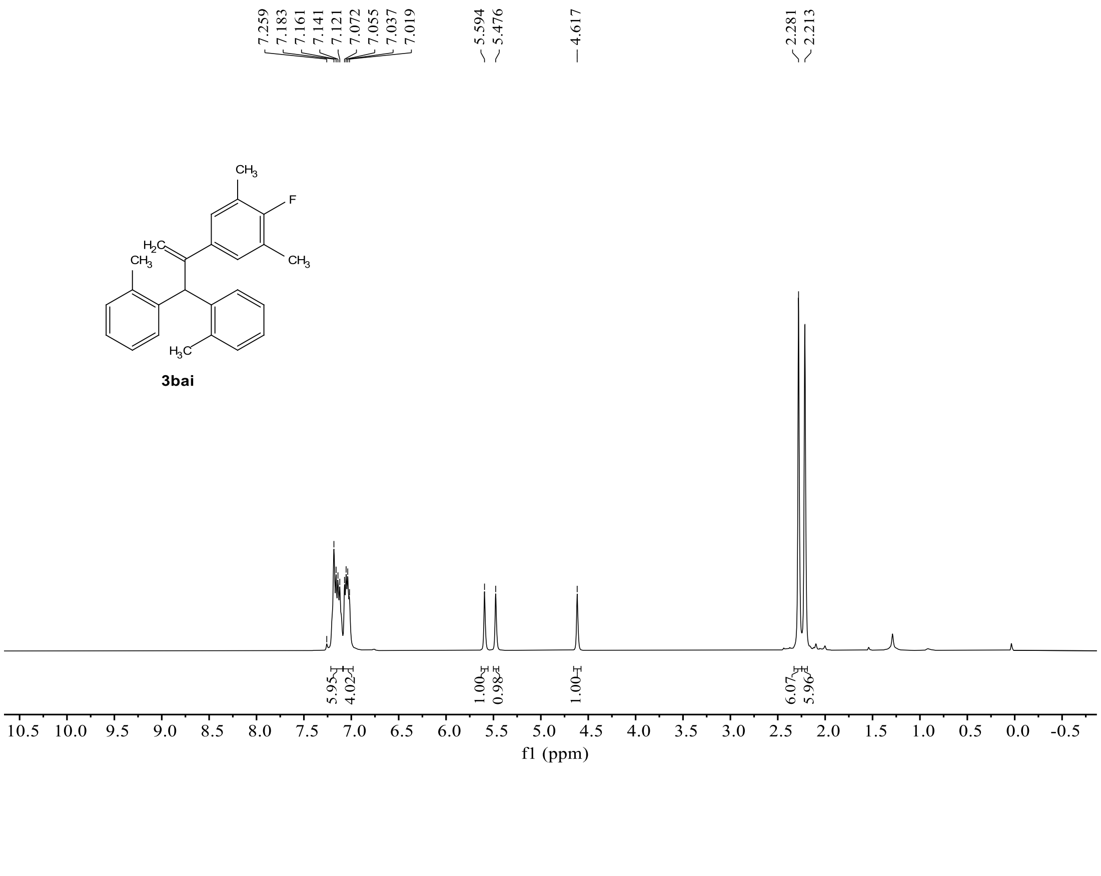

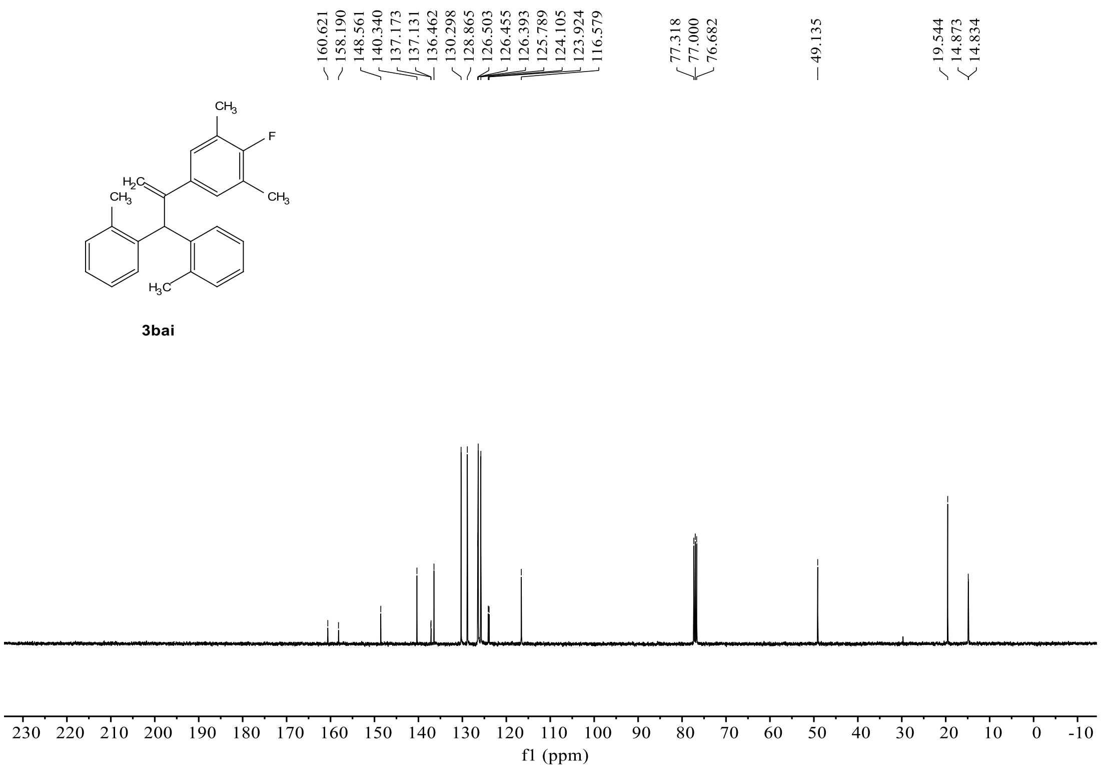

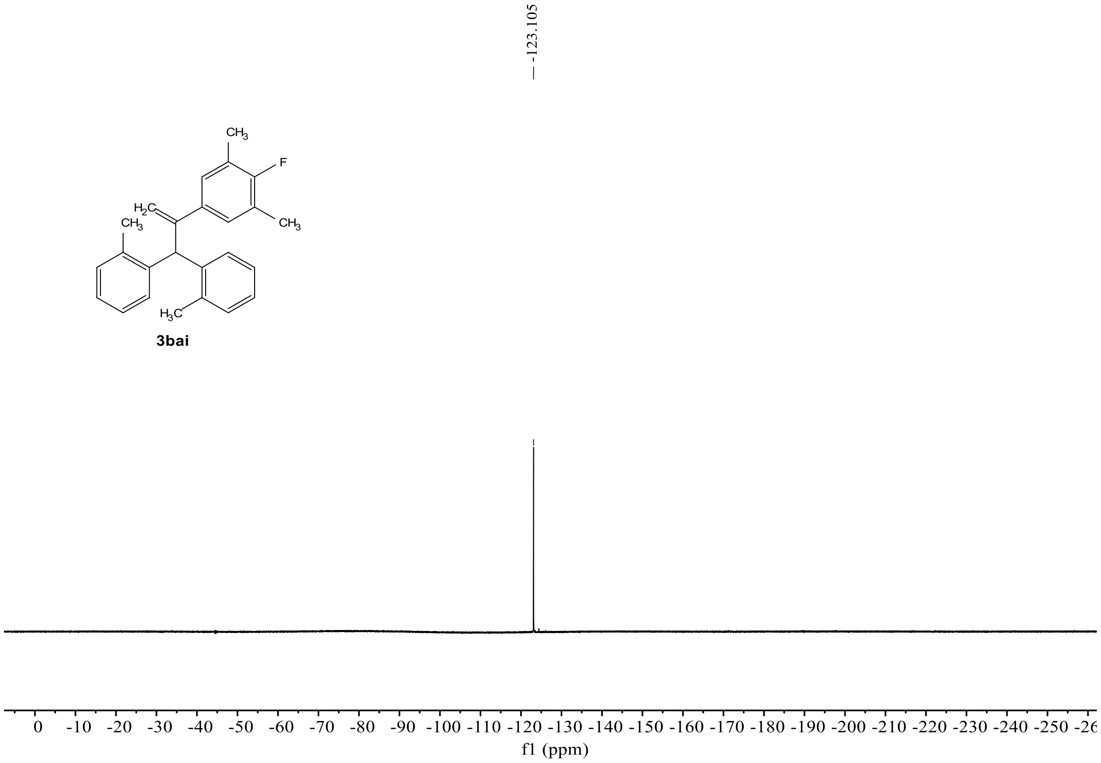

Figure S94. ^{1}H NMR ($CDCl_3$, 400 MHz), ^{13}C NMR ($CDCl_3$, 100 MHz), ^{19}F NMR ($CDCl_3$, 376 MHz) spectra of compound **3bai**

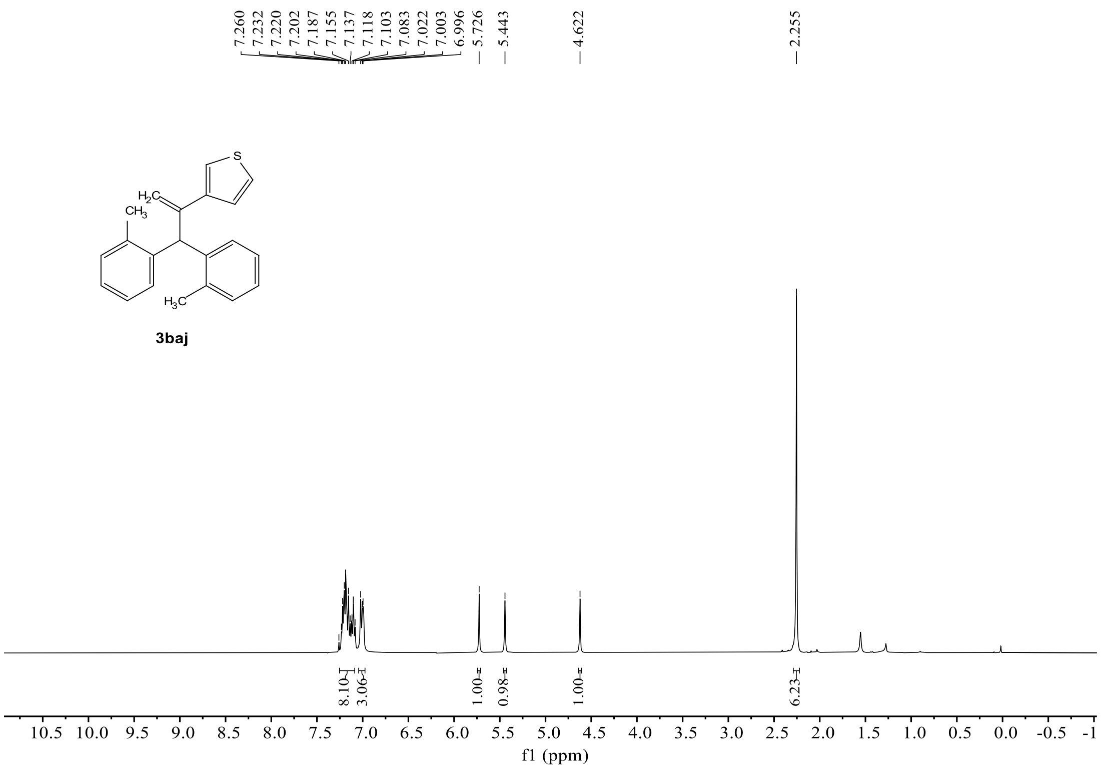

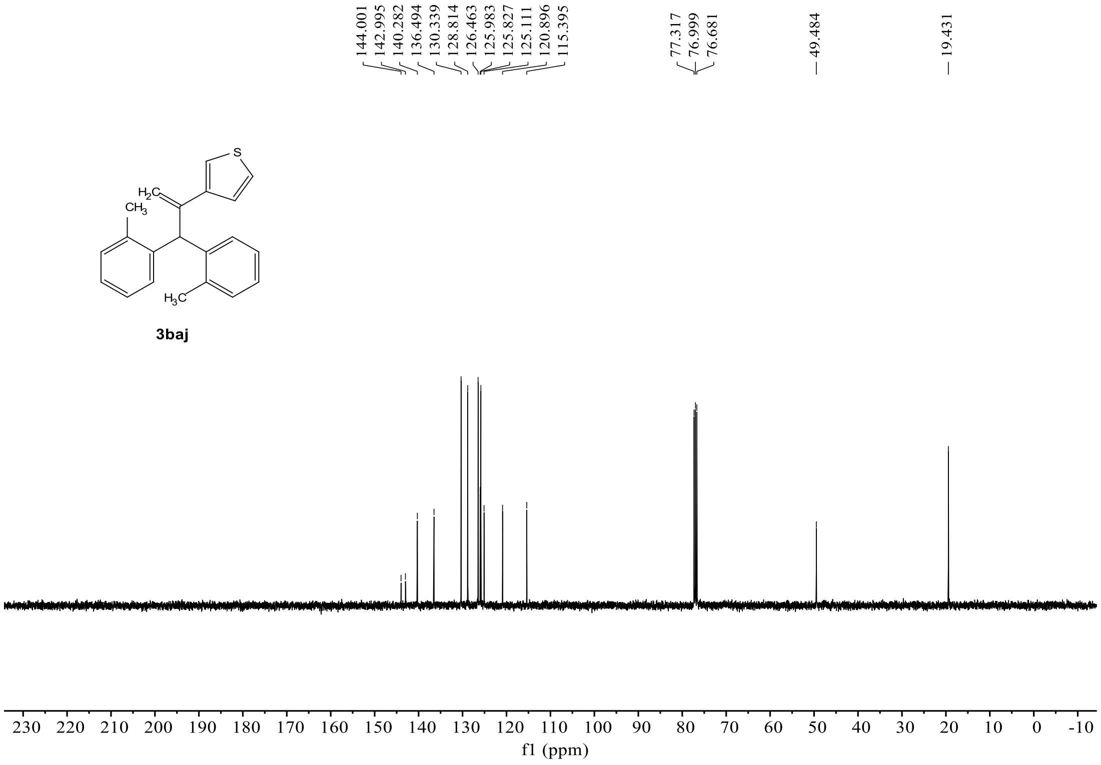

Figure S95. ^{1}H NMR ($CDCl_3$, 400 MHz) and ^{13}C NMR ($CDCl_3$, 100 MHz) spectra of compound **3baj**

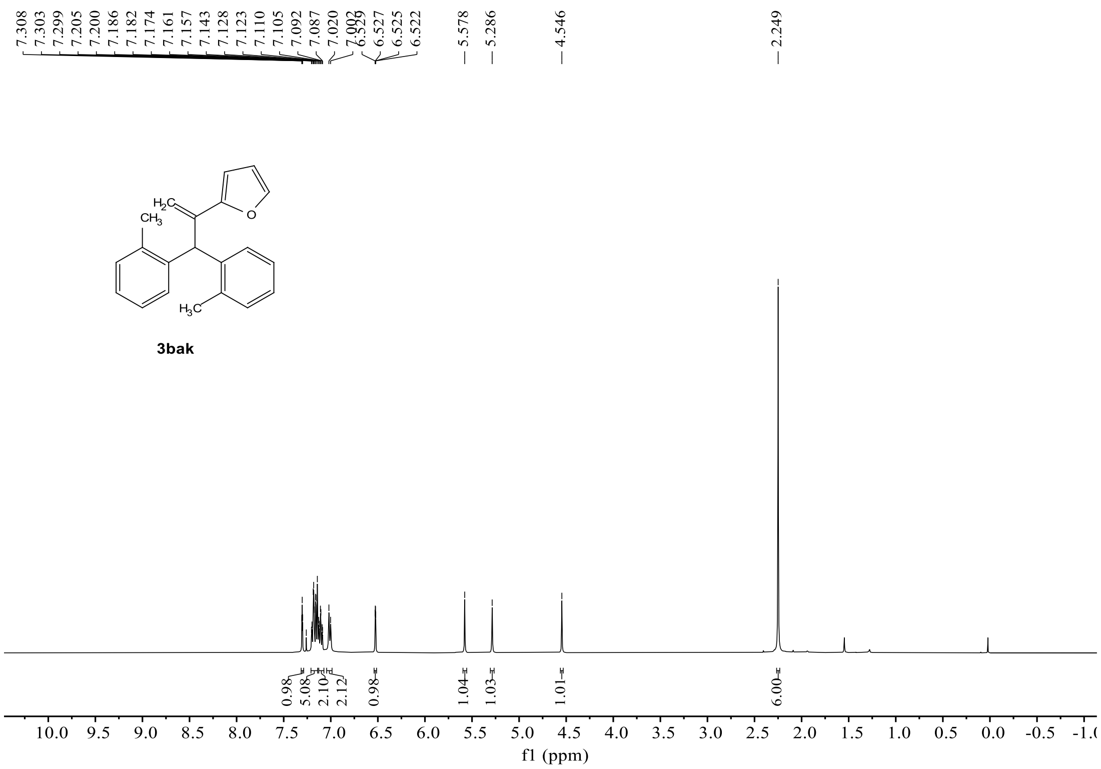

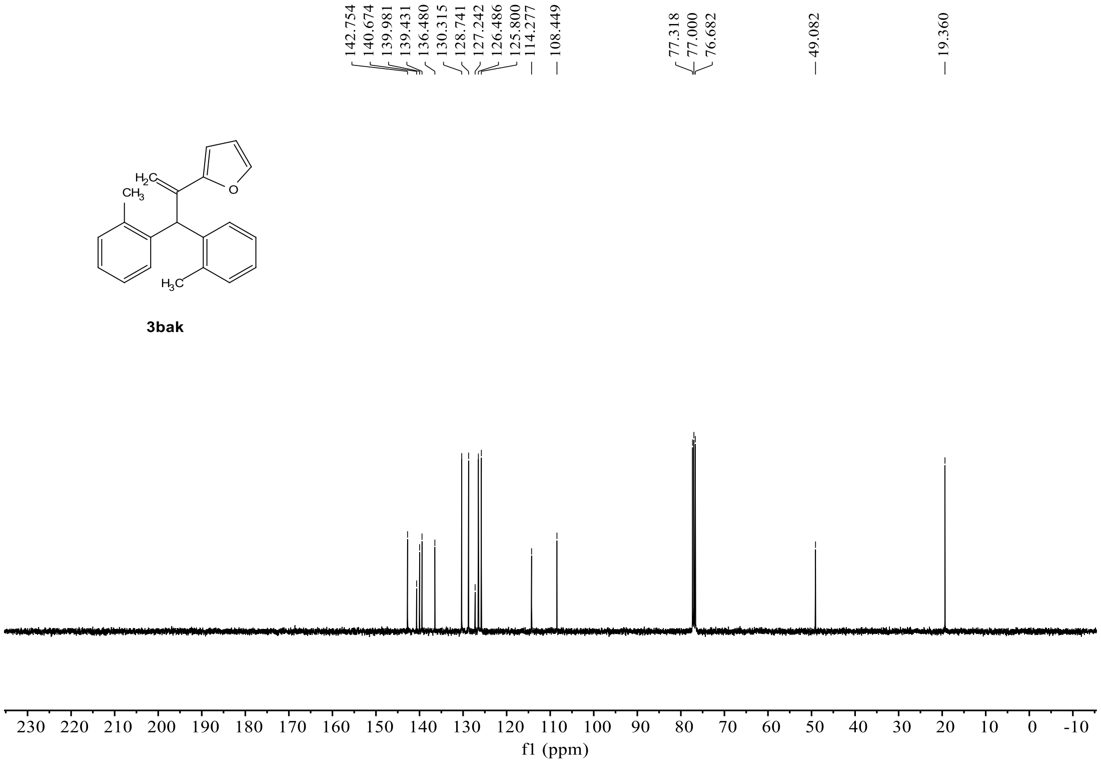

Figure S96. ^{1}H NMR ($CDCl_3$, 400 MHz) and ^{13}C NMR ($CDCl_3$, 100 MHz) spectra of compound **3bak**

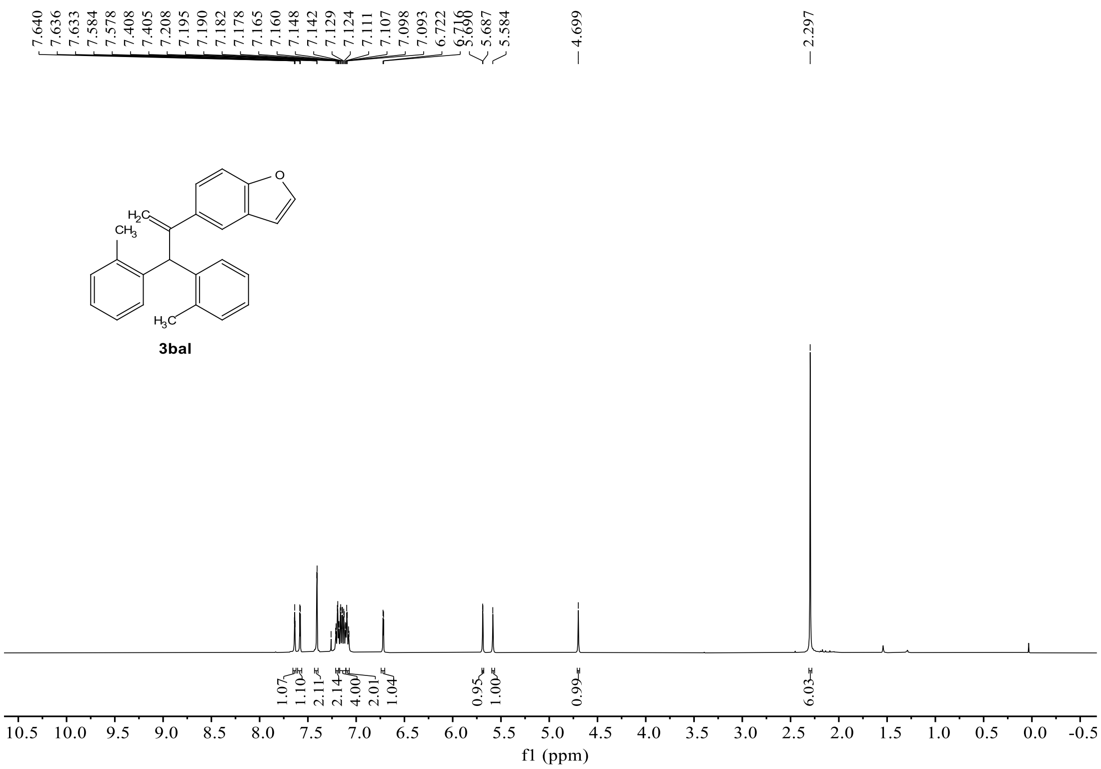

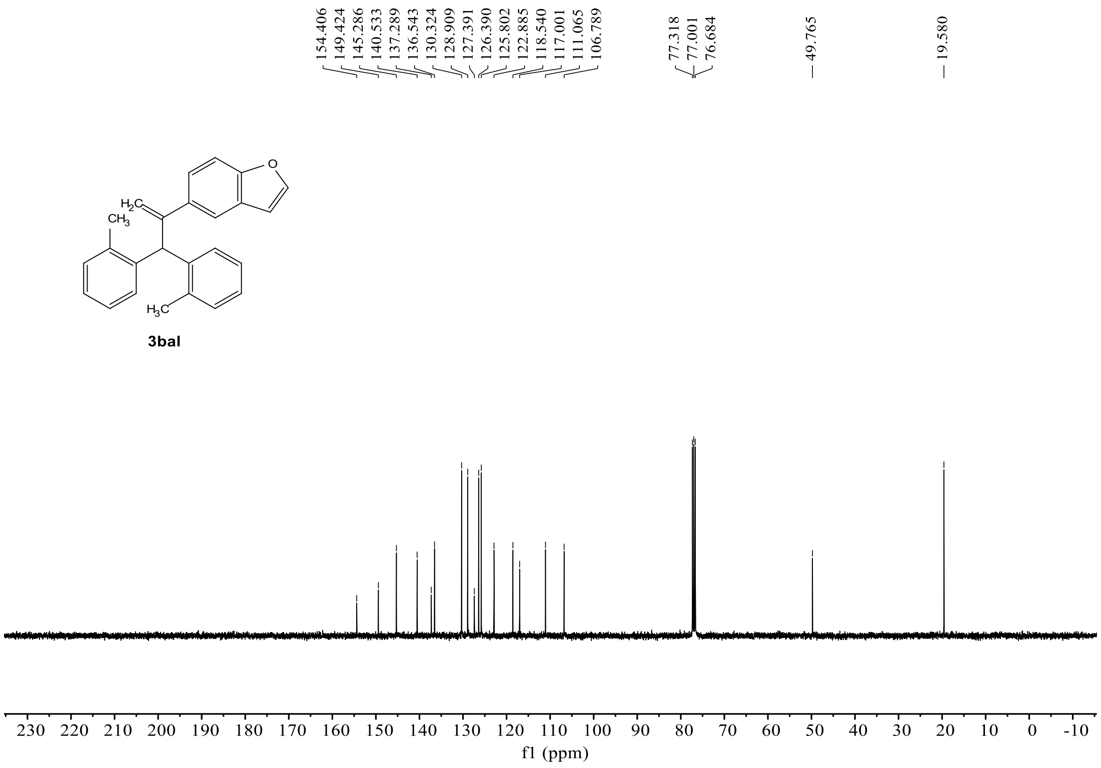

Figure S97. ^{1}H NMR ($CDCl_3$, 400 MHz) and ^{13}C NMR ($CDCl_3$, 100 MHz) spectra of compound **3bal**

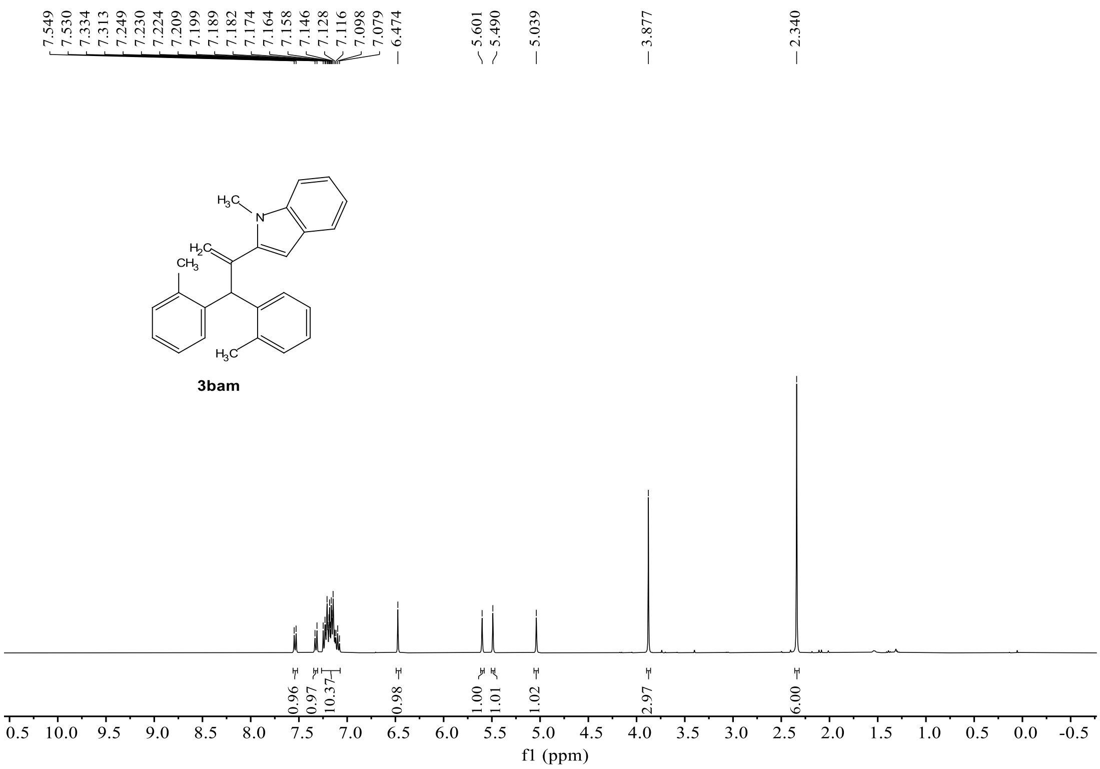

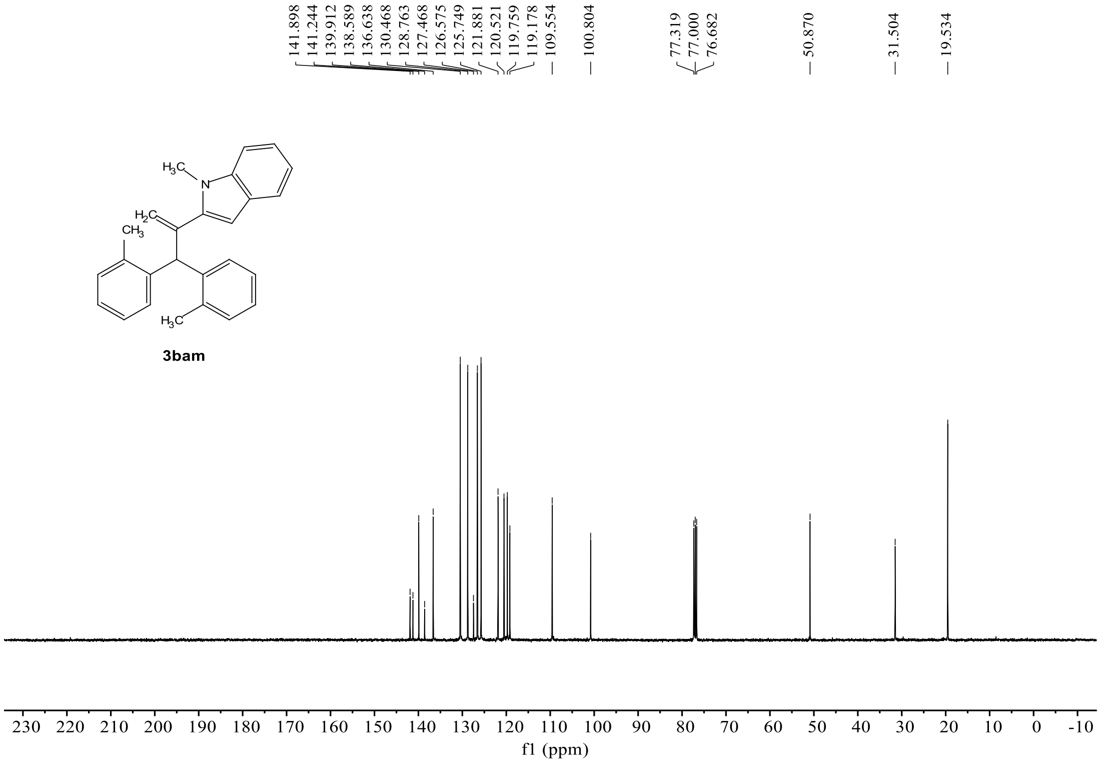

Figure S98. ^{1}H NMR ($CDCl_3$, 400 MHz) and ^{13}C NMR ($CDCl_3$, 100 MHz) spectra of compound **3bam**

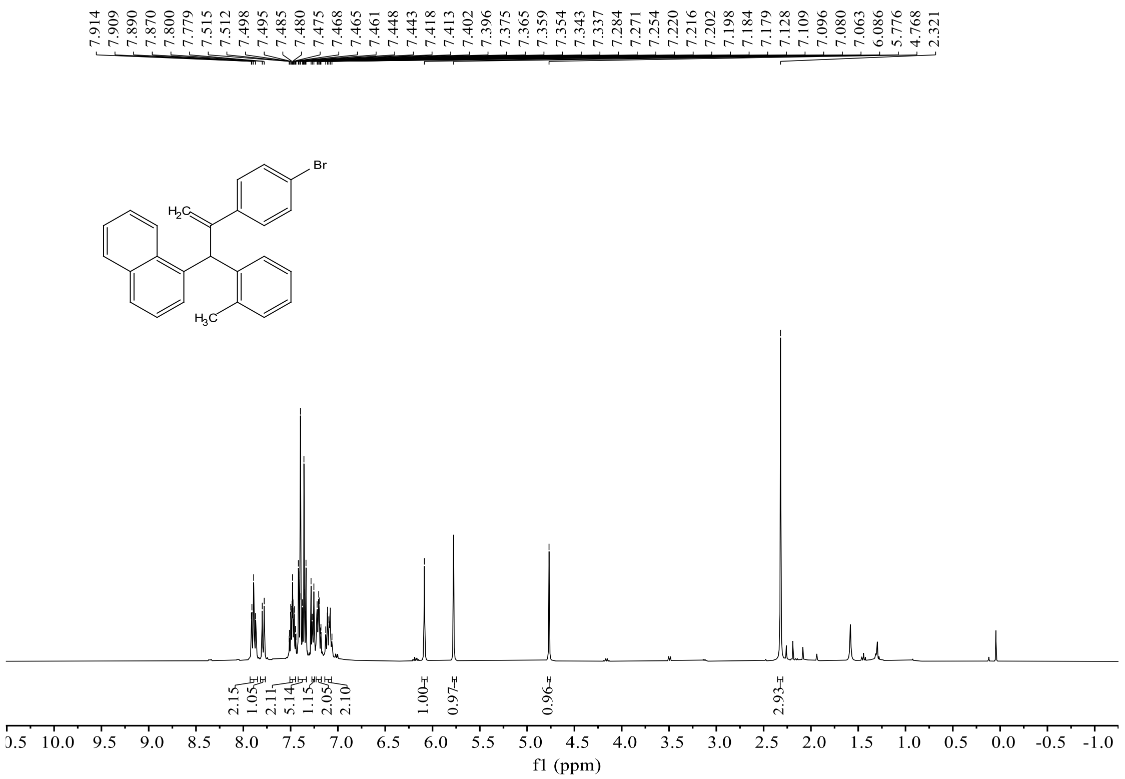

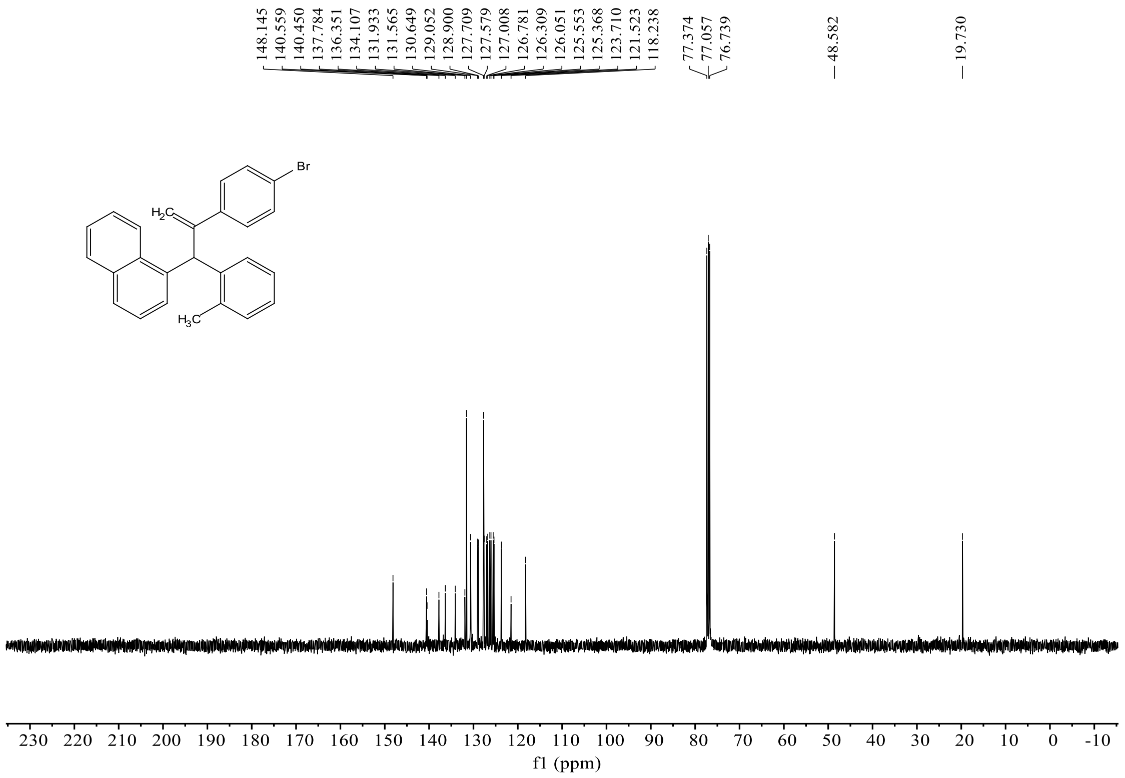

Figure S99. ^{1}H NMR ($CDCl_3$, 400 MHz) and ^{13}C NMR ($CDCl_3$, 100 MHz) spectra of compound **TBE-Br**

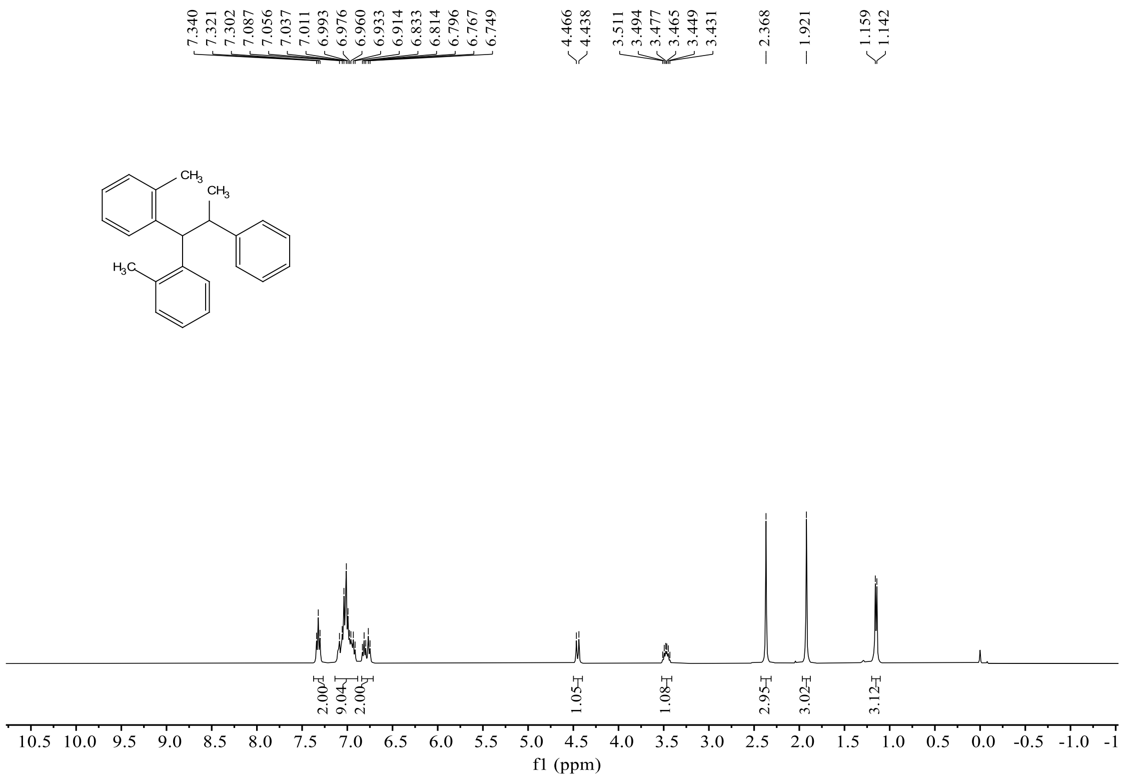

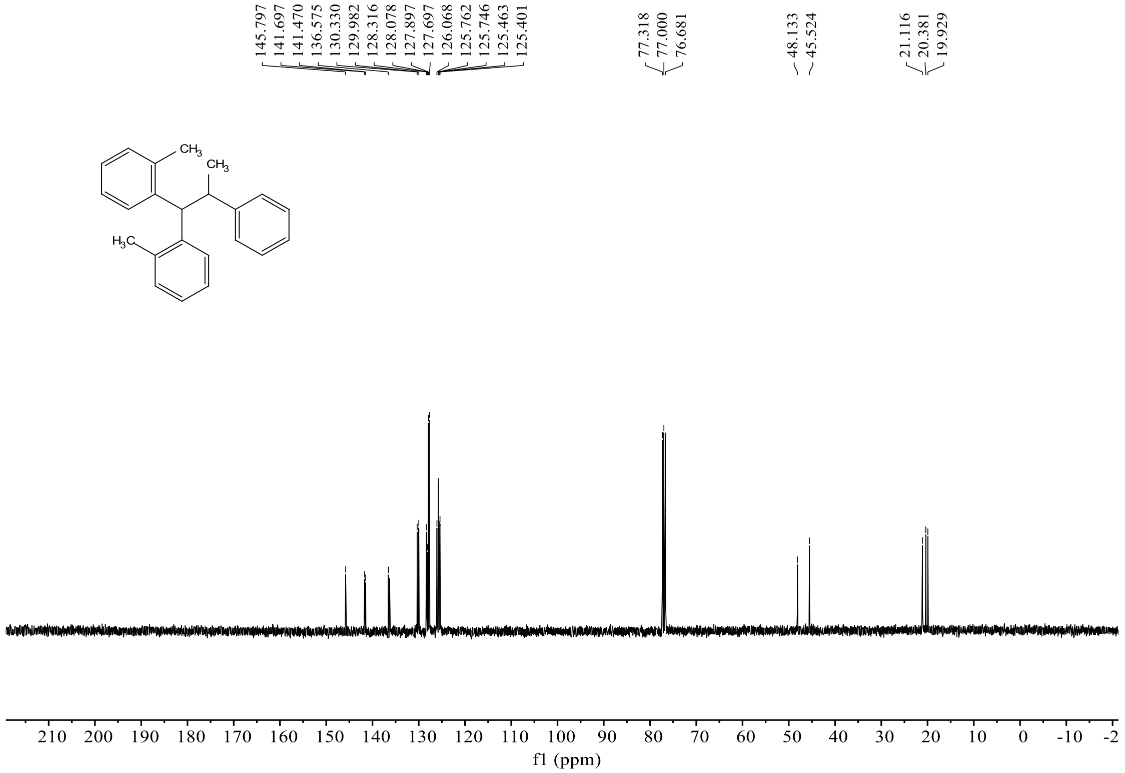

Figure S100. ^{1}H NMR ($CDCl_3$, 400 MHz) and ^{13}C NMR ($CDCl_3$, 100 MHz) spectra of compound **TBE-M-H**
